# A phase 1b/2 study of first-line anti-PD-L1/ TGF-βRII fusion protein SHR-1701 combined with nab-paclitaxel and gemcitabine for advanced pancreatic ductal adenocarcinoma

**DOI:** 10.1038/s41392-025-02530-2

**Published:** 2025-12-20

**Authors:** Ran Xue, Miaoyan Wei, Jiajia Yuan, Zhihua Li, Yuhong Zhou, Zeyun Xue, Yiwen Wu, Hongxia Han, Jun Zhou, Xianjun Yu, Lin Shen

**Affiliations:** 1 Key Laboratory of Carcinogenesis and Translational Research (Ministry of Education/Beijing), Department of Early Drug Development Centre, Peking University Cancer Hospital & Institute, Beijing, 100142 China; 2 Department of Pancreatic Surgery, Fudan University Shanghai Cancer Center, Shanghai, China; 3 Department of Oncology, Shanghai Medical College, Fudan University, Shanghai, China; 4 Shanghai Pancreatic Cancer Institute, Shanghai, China; 5 Pancreatic Cancer Institute, Fudan University, Shanghai, China; 6 Key Laboratory of Carcinogenesis and Translational Research (Ministry of Education/Beijing), Department of Gastrointestinal Oncology, Peking University Cancer Hospital & Institute, Beijing, 100142 China; 7 Department of Oncology, Sun Yat-sen Memorial Hospital, Sun Yat-sen University, Guangzhou, China; 8 Medical Oncology Department, Zhongshan Hospital Fudan University, Shanghai, China; 9 Jiangsu Hengrui Pharmaceuticals Co. Ltd., Shanghai, China; 10 State Key Laboratory of Holistic Integrative Management of Gastrointestinal Cancers, Beijing Key Laboratory of Cell & Gene Therapy for Solid Tumor, Department of GI Oncology, Peking University Cancer Hospital & Institute, Beijing, 100142 China

**Keywords:** Gastrointestinal cancer, Prognostic markers

## Abstract

Nab-paclitaxel plus gemcitabine (AG) is the standard first-line chemotherapy for advanced or metastatic pancreatic ductal adenocarcinoma and has limited efficacy. This phase 1b/2 study aimed to evaluate SHR-1701 (an anti-PD-L1/TGF-βRII fusion protein) plus AG in this population (NCT04624217). In phase 1b part, the recommended dose of SHR-1701 was identified as 30 mg/kg every 3 weeks, when combined with AG. In phase 2 part, the primary endpoint was objective response rate (ORR). As of Mar 31, 2023, 56 patients were enrolled. Median follow-up was 10.3 months (range, 0.2–24.7). ORR was 32.1% (95% CI, 20.3–46.0). Median progressive-free survival (PFS) was 5.6 months (95% CI, 4.3–6.6), and median overall survival (OS) was 10.3 months (95% CI, 8.8–12.3). Treatment-related adverse events of grade ≥3 were reported in 27 (48.2%) patients, with the most common being decreased neutrophil count. Patients with PD-L1 TPS ≥ 1% showed a higher ORR (66.7% vs. 25.0%), as well as extended median PFS (6.3 vs. 5.3 months) and median OS (18.8 vs. 9.9 months). Additionally, reduction of CA19-9 by at least 80% during treatment and pSMAD2/3 staining intensity of 1+ at baseline were potential monitoring tools and predictive biomarkers for better clinical outcomes, respectively. Tumor-specific T-cell infiltration and pancreatic cancer tumor subtypes were associated with anti-tumor response. The interactions within tumor microenvironment were involved disease progression. Overall, first-line SHR-1701 plus AG showed promising anti-tumor activity and controllable safety in advanced or metastatic pancreatic ductal adenocarcinoma, and features of patients more likely to benefit from the combination were drawn.

## Introduction

Pancreatic ductal adenocarcinoma is a highly aggressive disease and ranks as the third leading cause of cancer-related death.^1^ Its poor prognosis is characterized by a mortality rate that closely approaches its incidence, constituting a substantial global health challenge.^2^ Due to the deep-seated location of the pancreas and the asymptomatic nature of the disease at early stage, the majority of patients with pancreatic ductal adenocarcinoma present with advanced or metastatic disease at diagnosis, eliminating the possibility of curative resection.^2–6^ Chemotherapy remains the standard first-line treatment for advanced or metastatic pancreatic ductal adenocarcinoma, including nab-paclitaxel plus gemcitabine (AG), FOLFIRINOX (oxaliplatin, irinotecan, leucovorin, and fluorouracil), and NALIRIFOX (nanoliposomal irinotecan, 5-fluorouracil, leucovorin, and oxaliplatin) regimens.^7–9^ Nonetheless, these regimens provide limited survival benefits, and essentially all patients inevitably develop acquired resistance to chemotherapy. The mechanisms of resistance are complex and multifactorial, involving contributions from the dense fibrotic stroma, tumor cell heterogeneity, and adaptive cellular responses.^10^ Consequently, there is a pressing need to develop new clinical strategies to overcome resistance and improve the prognosis of pancreatic ductal adenocarcinoma.

Immunotherapy, particularly immune checkpoint inhibitors targeting programmed cell death receptor-1 (PD-1) or programmed cell death ligand-1 (PD-L1), have been developed as the potent and feasible therapeutic strategies and improved survival outcomes in many other cancer types, including melanoma, non-small cell lung cancer, and renal cell carcinoma. However, only limited or no activity was demonstrated in pancreatic ductal adenocarcinoma,^11–13^ due to the presence of multiple immunosuppressive entities within the tumor microenvironment (TME), such as transforming growth factor-beta (TGF-β). TGF-β contributes to immune evasion through multiple mechanisms: it can directly suppress the effector functions of CD8 + T cells and NK cells, promote the differentiation and recruitment of regulatory T cells and myeloid-derived suppressor cells, and inhibit T-cell trafficking into tumor islets. Beyond its immunomodulatory roles, TGF-β is a potent driver of the dense desmoplastic reaction characteristic of pancreatic ductal adenocarcinoma. This fibrotic stroma poses a physical barrier to drug penetration and fosters a hypoxic, nutrient-poor microenvironment that further compromises anti-tumor immunity.^14^ Given these challenges, dual inhibition of the PD-L1 pathway (to reinvigorate T cells) and the TGF-β pathway (to alleviate TME immunosuppression and stromal barriers) has emerged as a rationally designed and highly promising treatment strategy for pancreatic ductal adenocarcinoma, with the potential to overcome the limitations of single-agent checkpoint inhibitors.

SHR-1701 is a bifunctional fusion protein composed of an IgG4 monoclonal antibody against PD-L1 fused with the extracellular domain of TGF-β receptor II (TGF-βRII). This structure enables SHR-1701 to simultaneously block PD-1/PD-L1 interactions and neutralize TGF-β ligands in the tumor microenvironment. In vitro and in vivo pharmacological studies demonstrated that SHR-1701 had high affinity for PD-L1, TGF-β1, and TGF-β3, potent antitumor effect on xenograft in mice, and high PD-L1 target occupancy. In addition to targeting the PD-1/PD-L1 signaling pathway, SHR-1701 can also block the TGF-β/TGF-βR signaling axis. This dual action consequently inhibits multiple key steps in tumor progression, including tumor cell invasiveness, migration, and metastasis.^15^ Preclinical evidence suggests that neutralization of TGF-β can attenuate the epithelial-to-mesenchymal transition, a process associated with enhanced metastatic potential.^16^ Moreover, SHR-1701 exhibits the potential to suppress the immunosuppressive TME that is mediated by the TGF-β/TGF-βR signaling pathway.^17–19^ These mechanisms of action and preclinical findings support further clinical development of SHR-1701. It has been reported that SHR-1701 could overcome resistance to PD-1/PD-L1 inhibitors induced by disrupted lymphocyte recovery in lung cancer.^20^ SHR-1701 also exhibited encouraging antitumor activity and controllable safety in patients with recurrent or metastatic cervical cancer who had previously received platinum-based regimens.^21^ These collective findings from preclinical studies and early-phase clinical trials in various solid tumors provide a rationale for investigating SHR-1701 in pancreatic ductal adenocarcinoma, a malignancy characterized by an immunosuppressive tumor microenvironment and limited treatment options. However, until now, the clinical value of SHR-1701 in the treatment of pancreatic ductal adenocarcinoma has remained unclear.

This phase 1b/2 clinical trial was designed to evaluate the efficacy and safety of SHR-1701 combined with AG chemotherapy regimen in patients with previously untreated locally advanced and metastatic pancreatic cancer. Gemcitabine and nab-paclitaxel not only serve as the standard first-line cytotoxic backbone but have also been reported to induce immunogenic cell death, thereby enhancing tumor antigen release and presentation to antigen-presenting cells. This process can stimulate cytotoxic T-cell priming and activation, potentially sensitizing the tumor microenvironment to immunotherapy.^22^ In addition, chemotherapy can modulate immune cell composition by depleting myeloid-derived suppressor cells and tumor-associated macrophages, and by transiently reducing regulatory T-cell activity, thus alleviating immunosuppressive barriers.^23^ The combination aims to leverage this chemotherapy-induced immunogenic context in synergy with the dual immune and stromal modulation offered by SHR-1701. To our knowledge, this is the first clinical trial of an anti-PD-L1/TGF-βRII bifunctional fusion protein plus an established chemotherapy regimen in this challenging-to-treat patient population. This study seeks to assess whether this combination strategy can provide clinical benefit and address the unmet need in advanced pancreatic ductal adenocarcinoma.

## Results

### Tolerability and recommended phase 2 dose (RP2D)

In the phase 1b portion, 6 patients were enrolled to receive SHR-1701 at 30 mg/kg combined with AG regimen. No dose-limiting toxicities (DLTs) were observed during the 21-day observation period. Two (33.3%) patients achieved partial response (PR), 3 (50.0%) had stable disease, and 1 (16.7%) had progressive disease (PD). Combined with the tolerability, safety, anti-tumor activity, and pharmacokinetic data, 30 mg/kg every 3 weeks were determined to be the RP2D of SHR-1701, when combined with AG regimen. The 6 patients were entered in the phase 2 portion and continued to receive study treatment.

### Patients and treatment

**Fig. 1:**
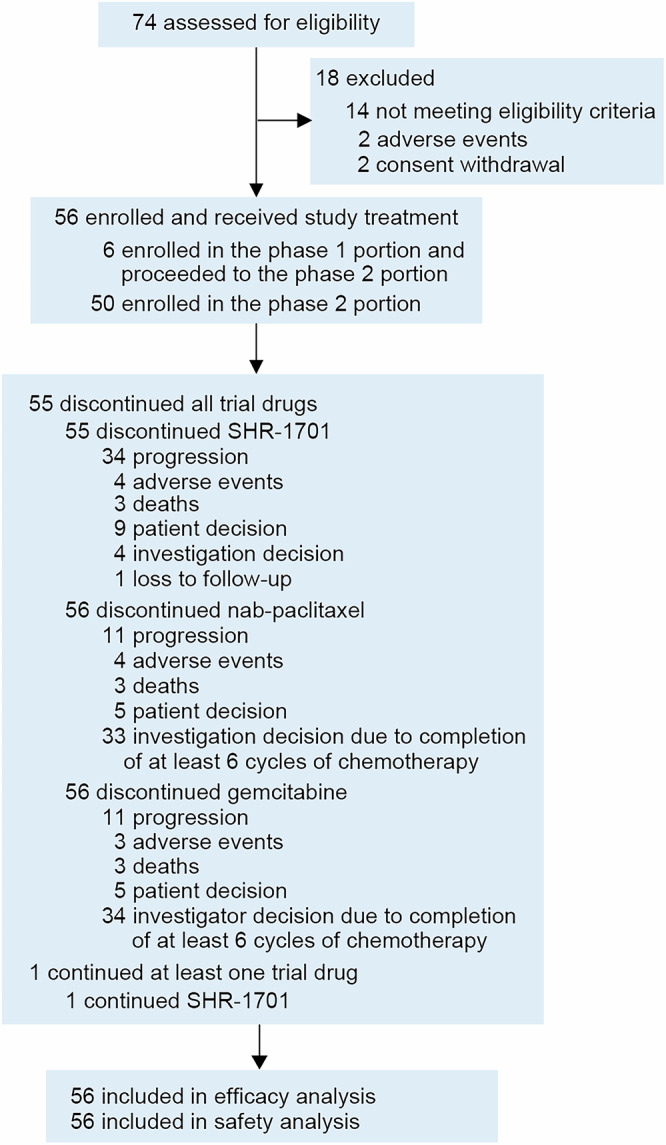
Trial profile

Between Dec 14, 2020 and Sep 16, 2021, a total of 56 patients were enrolled, and all received the study treatment at RP2D (Fig. 1). All patients had pancreatic ductal adenocarcinoma (Table 1). The median age was 60.5 years (range, 38.0–70.0), with males constituting 66.1% of the patients. The Eastern Cooperative Oncology Group (ECOG) performance status was 0 or 1 in 44.6% and 55.4% of patients, respectively. Additionally, 12.5% of patients had history of pancreatic tumor surgery. Only 1 (1.8%) patient with locally advanced pancreatic cancer was recruited, whereas the remaining 55 (98.2%) patients had distant metastatic disease. The most common distant metastatic sites were liver (71.4%) and peritoneum (53.6%).

**Table 1:** Patient demographics and clinical characteristics at baseline

	All patients (N = 56)
Age, years	60.5 (38.0–70.0)
Sex	
Male	37 (66.1%)
Female	19 (33.9%)
ECOG performance status	
0	25 (44.6%)
1	31 (55.4%)
Pathological type	
Pancreatic ductal adenocarcinoma	56 (100.0%)
Pancreatic tumor location	
Head	17 (30.4%)
Non-head	39 (69.6%)
Disease stage	
Locally advanced disease	1 (1.8%)
Distant metastatic disease	55 (98.2%)
Number of organs with metastases	
1	8 (14.3%)
2	28 (50.0%)
3 or more	20 (35.7%)
Metastatic sites (≥10%)	
Liver	40 (71.4%)
Lymph nodes	37 (66.1%)
Peritoneum	30 (53.6%)
Lung	12 (21.4%)
Stage at initial diagnosis	
I–III	7 (12.5%)
IV	49 (87.5%)
Stage before enrollment	
III	1 (1.8%)
IV	55 (98.2%)
Prior therapy	
Adjuvant chemotherapy	5 (8.9%)
Radical surgery	7 (12.5%)
PD-L1 CPS	
<1	35 (62.5%)
≥1	15 (26.8%)
<5	42 (75.0%)
≥5	8 (14.3%)
<10	45 (80.4%)
≥10	5 (8.9%)
Not evaluable	6 (10.7%)
PD-L1 TPS	
<1%	44 (78.6%)
1–49%	6 (10.7%)
Not evaluable	6 (10.7%)
CA19-9 level	
≤UNL	8 (14.3%)
>UNL and ≤1000 U/ml	22 (39.3%)
>1000 U/ml	26 (46.4%)

Data are median (range) or n (%)

*ECOG* Eastern Cooperative Oncology Group, *PD-L1* programmed cell death-ligand 1, *CPS* combined positive score, *TPS* tumor proportion score, *CA19-9* cancer antigen 19-9, *UNL* upper normal limit

As of Mar 31, 2023, with a median follow-up duration of 10.3 months (range, 0.2–24.7), 55 (98.2%) patients discontinued all study treatment compounds, and 1 (1.8%) patient remained on treatment with SHR-1701 alone. The most common reason for discontinuation of SHR-1701 was disease progression (60.7%), whereas 58.9% of patients discontinued AG chemotherapy at the discretion of the investigators due to completion of at least 6 treatment cycles (Fig. 1). The median exposure duration was 18.4 weeks (range, 3.0–85.3) for SHR-1701, 18.0 weeks (range, 1.0–35.6) for nab-paclitaxel, and 18.0 weeks (range, 1.0–35.6) for gemcitabine. After the end of study treatment, 42 (75.0%) patients received at least one subsequent anti-cancer therapy (Supplementary Table S1).

### Efficacy

**Fig. 2:**
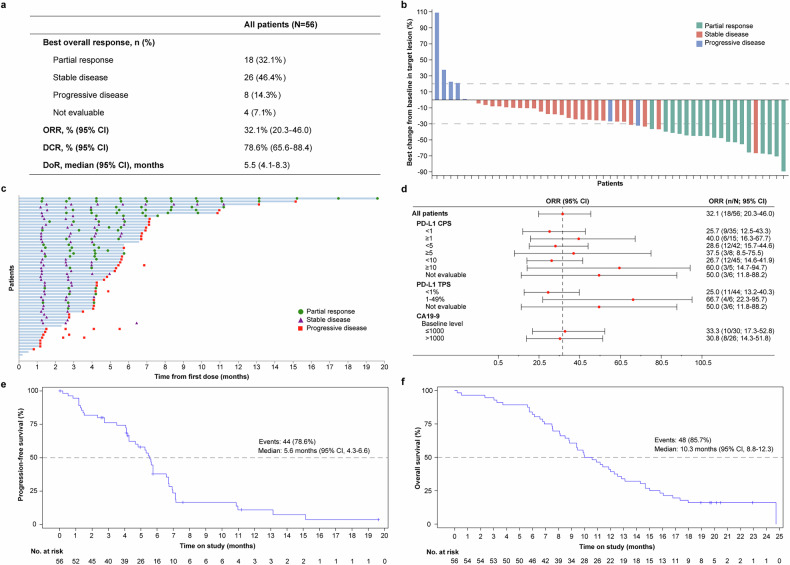
Efficacy assessments. **a** Anti-tumor responses. **b** Best percentage change from baseline in the sum of perpendicular diameters of target lesions. **c** Tumor responses per RECIST v1.1 over time. **d** Objective response rate in subgroups by PD-L1 and CA19-9 expression. **e** Kaplan–Meier estimates of progression-free survival. **f** Kaplan–Meier estimates of overall survival. ORR objective response rate, DCR disease control rate, DoR duration of response, PD-L1 programmed cell death-ligand 1, CPS combined positive score, TPS tumor proportion score, CA19-9 cancer antigen 19-9

All 56 patients received at least one dose of study treatment and were included for efficacy analyses (Fig. 2a). Of them, 18 patients achieved an objective response, resulting in a confirmed objective response rate (ORR) of 32.1% (95% CI, 20.3–46.0). The disease control rate (DCR) reached 78.6% (95% CI, 65.6–88.4). Tumor shrinkage was observed in 45 (88.2%) of 51 patients with pre-baseline and at least one post-baseline assessment for target lesions (Fig. 2b). The median duration of response (DoR) was 5.5 months (95% CI, 4.1–8.3; Fig. 2c).

In this study, 46 patients had primary tumors designated as target lesions, among whom 18 achieved an anti-tumor response (defined as a maximum reduction in target lesions from baseline exceeding 30%), resulting in a response rate of 39.1% (95% CI, 25.1–54.6). A total of 36 patients had liver metastases designated as target lesions, with 27 achieving an anti-tumor response, yielding a response rate of 75.0% (95% CI, 57.8–87.9).

As of the data cutoff, 44 (78.6%) patients had disease progression or died, and the median progression-free survival (PFS) was 5.6 months (95% CI, 4.3–6.6; Fig. 2e). The median time to progression (TTP) was 5.6 months (95% CI, 4.7–6.7). Totally, 48 (85.7%) deaths occurred, and the median overall survival (OS) was 10.3 months (95% CI, 8.8–12.3), with OS rates of 82.1% (95% CI, 69.4–90.0), 60.7% (95% CI, 46.7–72.1), and 39.3% (95% CI, 26.6–51.7) at 6, 9, and 12 months, respectively (Fig. 2f).

### Safety

Of the 56 patients, grade 3 or worse treatment-related adverse events (TRAEs) occurred in 27 (48.2%) patients (Supplementary Table S2), with the most common being decreased neutrophil count (14 [25.0%]), decreased white blood cell count (WBC) count (9 [16.1%]), increased gamma-glutamyltransferase (GGT) (3 [5.4%]), asthenia (3 [5.4%]), and neurotoxicity (3 [5.4%]; Table 2).

**Table 2:** Treatment-related adverse events

	All patients (N = 56)	
	Any grade	Grade 3 or 4
Anemia	48 (85.7%)	2 (3.6%)
Asthenia	44 (78.6%)	3 (5.4%)
White blood cell count decreased	40 (71.4%)	9 (16.1%)
Rash	40 (71.4%)	2 (3.6%)
Neutrophil count decreased	37 (66.1%)	14 (25.0%)
Alopecia	36 (64.3%)	0
Alanine aminotransferase increased	28 (50.0%)	1 (1.8%)
Aspartate aminotransferase increased	26 (46.4%)	1 (1.8%)
Platelet count decreased	19 (33.9%)	2 (3.6%)
Nausea	19 (33.9%)	0
Vomiting	18 (32.1%)	1 (1.8%)
Neurotoxicity	16 (28.6%)	3 (5.4%)
Pyrexia	16 (28.6%)	0
Gingival bleeding	16 (28.6%)	0
Constipation	14 (25.0%)	0
Hypoesthesia	14 (25.0%)	0
Diarrhea	13 (23.2%)	1 (1.8%)
Decreased appetite	12 (21.4%)	1 (1.8%)
Gamma-glutamyltransferase increased	11 (19.6%)	3 (5.4%)
Bilirubin conjugated increased	11 (19.6%)	1 (1.8%)
Abdominal pain upper	10 (17.9%)	0
Proteinuria	9 (16.1%)	0
Infusion related reaction	8 (14.3%)	1 (1.8%)
Blood bilirubin increased	7 (12.5%)	1 (1.8%)
Edema peripheral	7 (12.5%)	1 (1.8%)
Mouth hemorrhage	7 (12.5%)	0

Data are n (%). Table lists TRAEs of any grade that occurred in at least 10% of patients and the corresponding TRAEs of grade 3 or 4. Grade 5 TRAE occurred in 1 (1.8%) patient, which was upper gastrointestinal hemorrhage. TRAE, treatment-related adverse event

TRAEs led to the interruption of any study treatment compound in 24 (42.9%) patients, primarily due to decreased neutrophil count (5 patients [8.9%]), increased alanine aminotransferase (ALT) (4 [7.1%]), asthenia (4 [7.1%]), and decreased WBC count (3 [5.4%]). Ten (17.9%) patients had chemotherapy dose reductions owing to TRAEs, mainly including decreased neutrophil count (4 [7.1%]) and decreased WBC count (3 [5.4%]). All study treatment compounds had to discontinued because of TRAEs in 5 (8.9%) patients (immune-mediated hepatitis, neurotoxicity, immune-mediated lung disease, dermatomyositis, and edema peripheral; 1 [1.8%] for each).

Nine (16.1%) patients experienced serious TRAEs, with decreased WBC count reporting in 3 (5.4%) patients, decreased neutrophil count in 2 (3.6%), and upper gastrointestinal hemorrhage in 2 (3.6%) (Supplementary Table S3).

Treatment-related death occurred in 1 (1.8%) patient, which was upper gastrointestinal hemorrhage. The patient experienced chemotherapy-induced grade 4 decreased platelet count, and the death was deemed to be related to chemotherapy.

Any grade immune-related adverse events (irAEs) per investigator occurred in 25 (44.6%) patients, and grade 3 or worse irAEs were reported in 7 (12.5%) patients (Supplementary Table S4). The most common irAEs with an incidence of more than 10% were rash, gingival bleeding, increased ALT, increased aspartate aminotransferase (AST), increased GGT, diarrhea, and proteinuria. irAEs required systemic hormone therapies in 4 (7.1%) patients.

### PK

Semi-logarithm mean serum concentration–time profile of SHR-1701 at 30 mg/kg every 3 weeks, when combined with AG regimen, is shown in Supplementary Fig. S1. We found that coadministration of AG regimen had no apparent impact on the serum concentration of SHR-1701. The trough concentration was 91.2, 102.0, and 108.0 μg/mL at the end of cycle 1, 3, and 6, which were similar to the historical data of SHR-1701 single agent at the same dose.^24^

### Biomarker analyses

#### PD-L1 and cancer antigen 19-9 (CA19-9) expression

To investigate potential biomarkers of response, we evaluated the association of baseline PD-L1 expression and on-treatment CA19-9 dynamics with clinical efficacy. Baseline expression of PD-L1 and a reduction of CA19-9 by at least 80% during treatment tended to be positively correlated with clinical efficacy outcomes (Fig. 2d and Supplementary Table S5).

A high level of baseline PD-L1 was associated with a numerically elevated ORR (combined positive score [CPS] ≥1 vs. < 1: 40.0% vs. 25.7%; CPS ≥ 5 vs. < 5: 37.5% vs. 28.6%; CPS ≥ 10 vs. < 10: 60.0% vs. 26.7%; tumor proportion score [TPS] 1–49% vs. < 1%: 66.7% vs. 25.0%). Moreover, the improvement in ORR translated to prolonged PFS (median: 6.9 vs. 5.5 months for CPS ≥ 5 vs. < 5, 6.9 vs. 5.5 months for CPS ≥ 10 vs. < 10, and 6.3 vs. 5.3 months for TPS 1–49% vs. < 1%) and OS (median: 14.8 vs. 9.9, 12.8 vs. 10.0, and 18.8 vs. 9.9 months, respectively), except for CPS ≥ 1 vs. < 1. The contrast-enhanced CT images of a patient with high PD-L1 expression are presented in Supplementary Fig. S2. The patient had a PD-L1 CPS of 20 and a TPS of 10%. As shown in the images, the size of the target lesion was substantially reduced after 12 weeks of treatment (indicated by arrows). The patient achieved a best overall response of PR.

Compared to those with baseline CA19-9 > 1000 U/ml, patients with a level of ≤1000 showed similar ORR (33.3% vs. 30.8%) and median PFS (5.7 vs. 5.3 months), but had extended OS (median: 12.7 vs. 8.1 months). In addition, when CA19-9 was analyzed as a time-dependent variable, patients who achieved a maximum reduction of 80% or more from baseline to the end of treatment showed a 71% lower risk of progression or death (hazard ratio = 0.29; 95% CI, 0.14–0.58) and a 55% lower risk of death (hazard ratio = 0.45; 95% CI, 0.25–0.83), compared with those who did not. These findings provide mechanistic support for the efficacy of SHR-1701 combination therapy.

#### Signaling pathways, immunophenotype, and immune response biomarkers

To elucidate the molecular and immunological features underlying clinical responses, we compared the gene expression between patients who had a best overall response of either PR or SD lasting for at least 6 months (henceforth referred to as responders, n = 19) and those with SD lasting less than 6 months and PD (henceforth referred to as nonresponders, n = 33). Totally, 1078 differentially expressed genes (DEGs) were identified, including 131 higher-expressed and 947 lower-expressed genes (Supplementary Fig. S3a). Findings of Gene Ontology (GO) and Kyoto Encyclopedia of Genes and Genomes (KEGG) enrichment analyses for DEGs are shown in Supplementary Fig. S3b, c, respectively. Genes highly expressed in responders were enriched in the pathways involved in peroxisome proliferator-activated receptors signaling, cell adhesion molecules, extracellular matrix (ECM)-receptor interaction, and Th1 and Th2 cell differentiation, whereas lower-expressed genes were enriched in the pathway involved in antigen processing and presentation process.

The overall profile of the immune microenvironment in responders versus nonresponders is shown in Supplementary Fig. S4. The proportion of inactivated macrophage M0 was lower in responders compared with non-responders (p = 0.032). Conversely, there was a trend that the proportions of activated macrophages M1 and M2 were higher, despite no significant difference.

We further analyzed several potential biomarkers among responders versus nonresponders, previously reported to be linked with immune response. The bulk RNA-seq identified a cluster of T cells and 3 immune infiltration or fibrosis-associated signatures (such as interleukin-inflammatory CAF [IL-iCAF]) that were associated with responders (Supplementary Table S6). These biomarker analyses thereby provide supportive evidence for the clinical efficacy of SHR-1701 in combination therapy, highlighting patient subgroups that may derive particular benefit.

#### pSMAD2/3 and CD8+T cells

To assess the prognostic value of TGF-β pathway activity and immune contexture, we evaluated correlations of pSMAD2/3 and CD8 + T cell levels with PFS and OS were analyzed. The results showed that higher proportion of tumor cells exhibiting a pSMAD2/3 staining intensity of 1+ was associated with improved PFS (hazard ratio, 0.87 [95% CI, 0.77–0.99]; p = 0.028; Supplementary Table S7), providing clinical evidence that SHR-1701 effectively engages the TGF-β pathway.

#### Tumor specific T cell infiltration, subtype of pancreatic cancer, and immune microenvironment

**Fig. 3:**
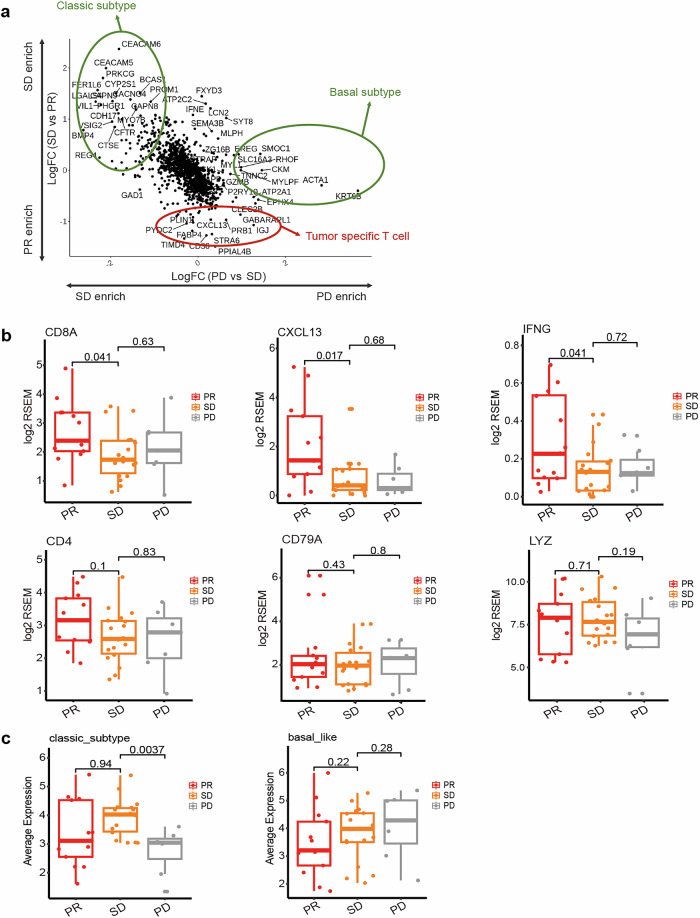
Associations of tumor-specific T cell infiltration and pancreatic cancer tumor subtype with the best overall response. **a** DEG plots between patients with PR versus SD and between patients with SD versus PD. **b** Association of genes involved in T cell-mediated immune responses with the best overall response. **c** Association of pancreatic cancer tumor subtype with the best overall response. Totally, 38 patients were included in these analyses, including 13 patients with PR, 18 patients with SD, and 7 with PD. In the boxplot, the bars represent whiskers, the range of the data. p values are nominal. DEG differentially expressed gene, PR partial response, SD stable disease, PD progressive response, FC fold change, RSEM RNA-Seq by expectation-maximization

To further validate the link between T cell infiltration and therapeutic efficacy of SHR-1701 plus AG regimen, differential gene analysis was performed between patients with PR versus SD and those with SD versus PD. Our findings revealed that the PR-enriched genes were associated with tumor specific T cells (Fig. 3a), and PR patients displayed higher abundance of tumor specific T cells, evidenced by elevated levels of *CD8A*, *CXCL13*, and *IFNG* genes (Fig. 3b). Moreover, PR patients were more likely to have non-basal-like subtype of pancreatic cancer; in contrast, SD patients predominantly had classic subtype, and PD patients were primarily characterized by the basal-like subtype (Fig. 3a, c).

**Fig. 4:**
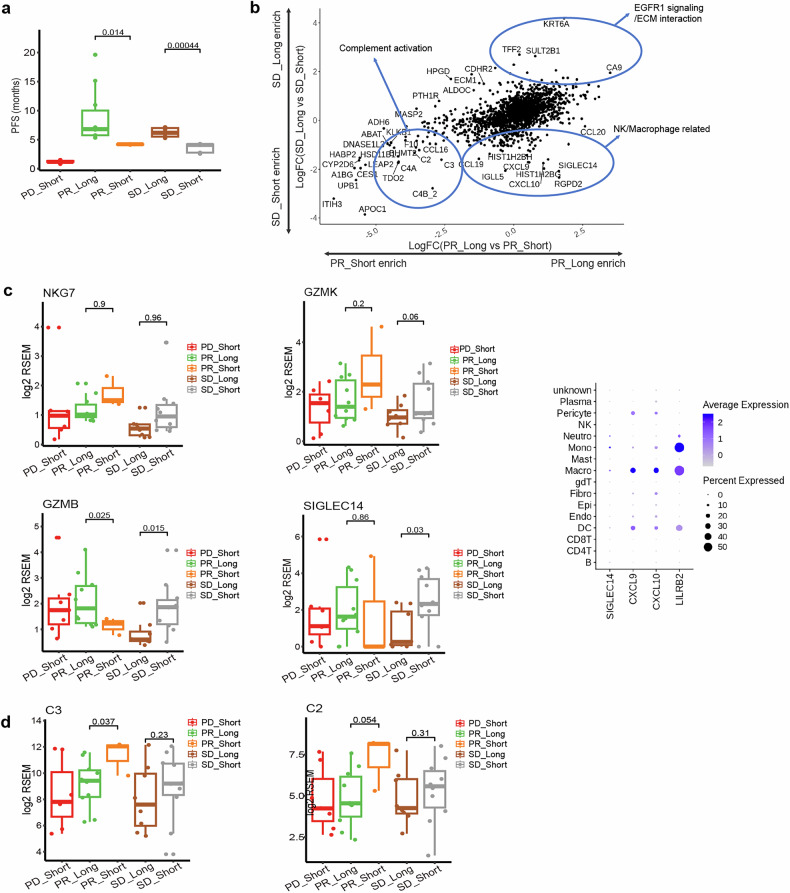
Associations of tumor microenvironment and complement activation with PFS. **a** Classification of long-survival and short-survival patients according to a PFS cut-off of 5 months. Patients with SD as the best overall response and a PFS of >5 months were defined as SD_Long; patients with SD and a PFS of <5 months were defined as SD_Short; patients with PR and a PFS of >5 months were defined as PR_Long; and patients with PR and a PFS of <5 months were defined as PR_Short. **b** DEG plots between patients with SD_Long versus SD_Short and between patients with PR_Long versus PR_Short. **c** SD_Short patients had more infiltration of NK and myeloid cells. **d** PR_Short patients showed complement activation. Totally, 38 patients were included in these analyses, including 7 patients with PD_Short, 10 with PR_Long, 3 with PR_Short, 8 with SD_Long, and 10 with SD_Short. In the boxplot, the bars represent whiskers, the range of the data. p values are nominal. DEG differentially expressed gene, PFS progression-free survival, PR partial response, SD stable disease, PD progressive response, FC fold change, RSEM RNA-Seq by expectation-maximization, NK natural killer

Based on the above analysis, since patients with SD and PR had their own characteristics, we hypothesized that the mechanisms responsible for long and short PFS in these patients might differ. Thus, the SD and PR groups were further divided into long-survival and short-survival patients according to a PFS cutoff of 5 months, generating four groups including SD_Long, SD_Short, PR_Long, and PR_Short (Fig. 4a). EGFR1 signaling/ECM interaction was enriched in patients with long PFS, either in those with PR or SD as the best overall response (Fig. 4b). Compared with SD_Long patients, SD_Short patients had relatively higher expression levels of *NKG7*, *GZMK*, *GZMB*, and *SIGLEC14*, demonstrating a more pronounced infiltration of natural killer (NK) and myeloid cells in patients with SD and short PFS (Fig. 4c). Compared with PR_Long patients, PR_Short patients showed increased expressions of genes encoding for the complement component 2 and 3 proteins, indicating an enhanced activation of the complement system in patients with PR and short PFS (Fig. 4d).

## Discussion

There is an urgent, unmet medical need to improve the prognosis of pancreatic ductal adenocarcinoma. In this phase 1b/2 trial, SHR-1701 combined with AG regimen showed an ORR of 32.1% and DCR of 78.6%. The median PFS was 5.6 months and the median OS was 10.3 months. The safety profile was similar with that of SHR-1701 monotherapy and chemotherapy. No unexpected TRAEs were observed. A total of 48.2% of patients experienced grade 3 or worse TRAEs, of which more than 10% were hematological toxicities (decreased neutrophil count and decreased WBC count). This study provides a novel and potentially effective treatment strategy with a bispecific agent targeting the PD-L1 and TGF-β pathways in combination with chemotherapy in this population.

Several clinical trials assessed the combination of immunotherapy targeting CTLA4 or PD-L1 with chemotherapeutic drugs as front-line treatment for pancreatic ductal adenocarcinoma. Tremelimumab plus gemcitabine showed a median OS of 7.4 months, with 2 of 28 (7.1%) evaluable patients achieving PR.^25^ The combination of pembrolizumab and AG regimen resulted in a median PFS and OS of 9.1 and 15.0 months, respectively, with 3 of 12 (25.0%) patients attaining PR.^26^ Inconsistent results were reported when combining nivolumab with AG regimen. In a phase 1b study, the ORR was 18% (9/50 patients), median PFS was 5.5 months, and median OS was 9.9 months.^27^ In the randomized phase 2 PRINCE trial of nivolumab and/or sotigalimab (CD40 agonistic antibody) combined with AG regimen, nivolumab combination arm showed an ORR of 35% (12/34 patients; unconfirmed), a median PFS of 6.4 months, and a median OS of 16.7 months.^28^ The investigators speculated that one possible explanation was the high proportion of patients with baseline PD-L1 > 1% (56%). In the sotigalimab plus AG regimen arm and the nivolumab and sotigalimab double immunotherapy plus AG regimen arm, the ORR was 33% (12/36) and 26% (9/35), median PFS was 7.3 and 6.7 months, and median OS was 11.4 and 10.1 months, respectively. Another dual immunotherapy with durvalumab and tremelimumab combined AG regimen demonstrated numerically improved ORR (30.3% vs. 23.0%), median PFS (5.5 vs. 5.4 months), and median OS (9.8 vs. 8.8 months) compared with AG regimen, but the differences were not significant.^29^ In summary, the combination of immunotherapy and chemotherapy for all-comers pancreatic ductal adenocarcinoma patients in the first-line setting demonstrated a trend toward better anti-tumor activity than AG alone (around 10% improvement in ORR), but has yet to yield substantial advancements in PFS. It is imperative to screen for biomarkers to identify the patient population that would derive the most benefit.

Baseline PD-L1 level tended to show positive correlation with antitumor response and survival benefit with SHR-1701 combined with AG regimen. The ORR was 37.5% in patients with PD-L1 CPS ≥ 5, and the median OS was 14.8 months. For population with PD-L1 TPS ≥ 1%, the ORR reached 66.7%, and the median OS extended 18.8 months, which were quite impressive. Perhaps PD-L1 positivity, represented by TPS, could serve as a reliable biomarker for SHR-1701 plus AG regimen in pancreatic ductal adenocarcinoma. However, it is essential to point out that only 10.7% of the patients included in this study were PD-L1 positive.

CA19-9 is a type of glycoprotein that is usually elevated in the blood of patients with pancreatic ductal adenocarcinoma. The ORR was similar between patients with baseline CA19-9≤ and >1000 U/ml. CA19-9 level can also be elevated in other conditions such as liver disease, inflammation of the gallbladder, cystic fibrosis, and diseases of the bile ducts, which might be an explanation of why its baseline level did not predict the response. But we found that patients with a ≥ 80% decrease in CA19-9 level from baseline to the end of treatment showed decreased risk of progression or death by 71% and decreased risk of death by 55%. CA19-9 decline had the potential to serves as a monitoring tool for the clinical outcomes of individual patient.

Additionally, positive correlation of baseline pSMAD2/3 level with PFS was noted. SMAD2 and 3 are pivotal downstream transcription factors in the TGF-β signaling pathway. Upon stimulation by TGF-β, these transcription factors undergo phosphorylation to be activated. The pSMAD2 and 3 form trimeric SMAD complexes with SMAD4, and subsequently regulate downstream genes involved in tumor progression and immunosuppressive microenvironment.^30,31^ Our findings further supported the mechanism of action of SHR-1701, and suggested that the baseline level of pSMAD2/3 might serve as a predictive biomarker for the efficacy of SHR-1701 plus AG regimen. It has been reported that the SMAD4 gene is deleted or mutated in over half of pancreatic ductal adenocarcinomas.^32,33^ Whether therapeutic responses differ depending on SMAD4 alterations at baseline warrants further investigation.

Following the treatment with the SHR-1701 plus AG regimen, DEGs between responders and non-responders were associated with Th1 and Th2 cell differentiation and antigen processing and presentation pathways. Concurrently, our analysis revealed that responders also had elevated levels of activated macrophages, both M1 and M2 subtypes, which play essential roles in the antigen presentation process. These observations suggest that responders might possess a more robust antigen processing and presentation capability, potentially triggering a stronger T cell mediated immune response. Studies have emphasized the pivotal role of CAFs in modulating the tumor immune microenvironment and directing the efficacy of cancer immunotherapies.^34,35^ We found that IL-iCAF was associated with anti-tumor activity following study treatment. IL-iCAF is not only involved in the inflammatory response within the tumor microenvironment but may also promote the proliferation, invasion, and metastasis of tumor cells.^36,37^

The above signaling pathways, immunophenotype, and immune response biomarkers analyses demonstrated the potential predictive role of tumor specific T cell infiltration for the efficacy of SHR-1701 plus AG regimen. To further validate the link, we identified the PR-, SD-, and PD-enriched genes. Compared with SD and PD patients, PR patients showed higher levels of *CD8A*, *CD4*, *CXCL13*, and *IFNG* genes. These genes are involved in T cell function and the immune response.^38–42^ Enrichment of these genes indicated higher abundance of tumor specific T cells in patients who achieved PR following SHR-1701 plus AG. Previous study identified two subtypes of pancreatic ductal adenocarcinoma, named classical and basal-like, and patients with basal-like disease had worse outcomes.^43^ Consistently, we also found that classic subtype was associated with SD, and basal-like subtype was associated with PD.

Patients who could achieved a long PFS exhibited genes enrichment in ECM interaction, consistent with the mechanism of action of SHR-1701. In the advanced stage of cancers, TGF-β can promote tumor growth, invasion, and metastasis by modulating the ECM composition and cell-ECM interactions.^44,45^ Therefore, by inhibiting TGF-β, SHR-1701 is capable of slowing down the progression of tumor. Furthermore, our analysis revealed that patients with a notable infiltration of NK and myeloid cells, as indicated by relatively higher expression levels of *NKG7*, *GZMK*, *GZMB*, and *SIGLEC14*, were prone to disease progression faster, even if they experienced a SD. Complement activation in the tumor microenvironment promotes tumorigenesis and progression.^46^ Our findings further indicated a correlation between an activated complement system and rapid disease progression in patients who achieved PR after study treatment. Combined, we successfully delineated the characteristics of patients who were likely to benefit from SHR-1701 plus AG (Supplementary Fig. S5).

In terms of safety, the most common grade 3 or worse TRAEs following SHR-1701 plus AG were hematological toxicities (decreased neutrophil count, 25.0%; decreased WBC count, 16.1%), which did not appear to obviously exacerbate the toxicity of AG. No new safety signals were found. Bintrafusp alfa (M7824) is the first-in-class anti-PD-L1/TGF-βRII bifunctional fusion molecule, but its development has been discontinued, partly due to poor safety.^47^ Unlike bintrafusp alfa, which uses an IgG1 monoclonal antibody, SHR-1701 employs an IgG4 monoclonal antibody against PD-L1. SHR-1701 has minimal to no effects of antibody-dependent cytotoxicity, complement-dependent cytotoxicity, and antibody-dependent cellular phagocytosis, thereby avoiding cytotoxicity against immune cells expressing PD-L1 and ensuring better safety. In this study, the only bleeding events occurring in at least 10% of patients were gingival bleeding and mouth hemorrhage, and these events were mostly grade 1, with the exception of one grade 2 gingival bleeding.

This study has several limitations. Firstly, this was a single-arm phase 1b/2 exploratory trial, with no statistical hypothesis test. Further investigations are required to address whether SHR-1701 in combination with chemotherapy is superior to standard-of-care treatment. Moreover, the associations between biomarkers and clinical outcomes were assessed solely in univariable models. These findings should be interpreted cautiously due to small simple size and risk of confounding, and must be validated in large-scale clinical trials. In addition, further analyses are warranted, such as analyzing the subtypes of metastatic lesions and their correlation with treatment response, exploring the dynamic changes of tumor subtypes and immune profiles during treatment, and analyzing other biomarkers like tumor mutational burden, microsatellite instability/mismatch repair status, and SMAD4 gene alterations at baseline. Last, this study enrolled patients up to 70 years of age. There is currently insufficient evidence for the efficacy and safety of SHR-1701 plus AG in elderly patients with pancreatic ductal adenocarcinoma.

In conclusion, the combination of SHR-1701 with AG regimen was safe and demonstrated clinical efficacy in untreated patients with advanced or metastatic pancreatic ductal adenocarcinoma. Several potential predictive biomarkers associated with improved clinical outcomes were identified, including baseline PD-L1 positive expression and pSMAD2/3 staining intensity of 1+ at baseline. CA19-9 reduction during treatment could serve as a monitoring tool, and a reduction by 80% was associated with better clinical outcomes. Tumor-specific T-cell infiltration and pancreatic ductal adenocarcinoma tumor subtypes were associated with anti-tumor response to SHR-1701 plus AG. The interactions within the tumor microenvironment might play a role in determining the disease progression. These findings provide valuable insights into the mechanisms of action of SHR-1701 and may guide the selection of patients who are most likely to benefit from this treatment combination.

## Materials and methods

### Patient selection

Eligible patients were aged 18 to 70 years and had histologically or cytologically confirmed ductal adenocarcinoma or acinar cell carcinoma of the pancreas with unresectable locally advanced or distant metastatic disease. Prior systemic anticancer therapies for pancreatic cancer were not permitted, with the exception of previous neoadjuvant or adjuvant chemotherapy completed at least 6 months before. All patients had an ECOG performance status of 0 or 1, at least one measurable lesion according to Response Evaluation Criteria in Solid Tumors (RECIST v1.1), life expectancy of at least 12 weeks, and adequate marrow and organ function (WBC ≥ 3 × 10^9^/L; absolute neutrophil count ≥1.5 × 10^9^/L; lymphocyte count ≥0.5×10^9^/L; platelet count ≥100 × 10^9^/L; hemoglobin ≥100 g/L; serum albumin ≥ 2.9 g/dL; serum creatinine ≤ 1.5 times the upper limit of normal value [ULN] or creatinine clearance rate ≥50 ml/min; total bilirubin ≤1.5 × ULN; ALT or AST level ≤3 × ULN for those without liver metastasis and ≤ 5 × ULN for those with liver metastasis; international normalized ratio ≤ 1.5 × ULN, prothrombin time and activated partial thromboplastin time ≤ 1.5 × ULN). All patients were required to provide fresh or archival tumor tissues after the last previous regimen before enrollment.

Key exclusion criteria included serious cardio-cerebrovascular disease, such as cerebral hemorrhage, cerebral infarction, or history of myocardial infarction, congestive heart failure ≥ Grade 2, within 6 months before enrollment; any inhibitors against PD-1, PD-L1, PD-L2, CD137, CTLA-4, TGF-β, or other drugs/antibodies that act on T cell costimulatory or checkpoint pathways; uncontrolled or symptomatic central nervous system metastases; active autoimmune disease or a history of autoimmune disease that might recur.

Written informed consent was obtained from all patients before study enrollment. The study was approved by the Ethics Committees of each participating site, including Beijing Cancer Hospital Medical Ethics Committee, Fudan University Shanghai Cancer Center Ethics Committee, Zhongshan Hospital Fudan University Ethics Committee, and Sun Yat-sen Memorial Hospital Ethics Committee. The study was conducted in accordance with the Declaration of Helsinki, Good Clinical Practice, and local laws and regulatory requirements.

### Study design and treatment

This study was a multicenter, open-label, phase 1b/2 study of SHR-1701 in combination with AG regimen in previously untreated patients with locally advanced or metastatic pancreatic cancer (ClinicalTrials.gov, number NCT04624217).

In our previous phase 1 study of SHR-1701 in advanced solid tumors, 30 mg/kg every 3 weeks was determined as the RP2D of SHR-1701 as monotherapy.^24^ In the phase 1b dose-finding portion of this study, SHR-1701 at 30 mg/kg on day 1 was initially tested in 6 patients, when combined with nab-paclitaxel at 125 mg/m^2^ and gemcitabine at 1000 mg/m^2^ on day 1 and 8. If more than one of the 6 patients experienced DLTs during the 21-day observation period, a reduced dose of SHR-1701 at 20 mg/kg would be tested; otherwise, the dose of 30 mg/kg every 3 weeks would be carried forward to the phase 2 clinical-expansion portion.

In all enrolled patients, SHR-1701 was given on day 1 and nab-paclitaxel and gemcitabine were administrated on day 1 and 8 of each 21-day cycle by intravenous infusion until disease progression, intolerable toxicity, investigator decision, or patient withdrawal, whichever occurred first. Given the cumulative toxicity of long-term use, no more than 6 cycles of chemotherapy were recommended. The patients who completed 6 cycles of chemotherapy plus SHR-1701 could be given SHR-1701 alone at the discretion of the investigators. In addition, SHR-1701 combination or monotherapy beyond the initial RECIST v1.1–defined PD was permitted, if the investigator judged that the patient could benefit from and tolerate to the continued treatment.

Dose modification of SHR-1701 was not allowed, but treatment interruptions were permitted to manage AEs until these events resolved to grade 0–1 or baseline level. Dose modification of nab-paclitaxel and gemcitabine was allowed; however, if the dose was reduced due to AEs, subsequent dose callback was not allowed.

### Study endpoints

The primary endpoint of the phase 1b portion was RP2D of SHR-1701, when combined with AG regimen. In the phase 2 portion, the primary endpoint was ORR per investigator according to RECIST v1.1. Secondary endpoints were investigator-assessed best overall response, DCR, DoR, PFS, and TTP according to RECIST v1.1; OS, 6-, 9-, and 12-month OS rate; and PK profile. Exploratory endpoints included relationship between biomarkers and clinical efficacy outcomes.

### Assessments

DLTs were assessed in all patients who were enrolled in the phase 1b portion, received at least one dose of the study treatment (all components, ≥90% of the prescribed dose), and either completed the 21-day observation period or experienced any DLT during the period. Definition for DLT is provided in the Supplementary Methods.

Radiographic assessments were done at baseline, every 6 weeks during the first 16 cycles, and every 9 weeks thereafter until PD, treatment discontinuation, start of new anticancer treatment, loss to follow-up, or death. Complete response and PR required confirmation by a subsequent repeat scan at least 4 weeks after initial response assessment. For patients who continued SHR-1701 combination or monotherapy beyond initial RECIST v1.1–defined PD, the disease progression needed to be confirmed through performing radiographic assessment at least 4 weeks later or at the next scheduled scan (i.e., no more than 9 weeks). Survival status was followed up monthly until death, loss to follow-up, or withdrawal of consent.

Safety was monitored from the time of informed consent to 90 days after the last administration of study medication. AEs were graded according to the National Cancer Institute Common Terminology Criteria for Adverse Events, version 5.0.

The PD-L1 expression in tumor samples was centrally determined by immunohistochemistry (IHC) assay (PD-L1 IHC 22C3 pharmDx test, Dako, Carpinteria) and expressed using CPS and TPS. CA19-9 levels were measured every 2 cycles. The phosphorylation of SMAD2 and 3 in tumor cells and interstitial immune cells were centrally detected by IHC (clone 138D4, Cell Signaling Technology). CD8 + T cells were identified by IHC (clone 4B11, Invitrogen). Total RNA was extracted from fresh or archival tumor tissues after the last previous regimen before patient enrollment. After RNA extraction and RNA library preparation, the samples were subjected to mRNA sequencing (MGISEQ-2000).

### Statistical analyses

The sample size for the phase 1b dose-finding portion was based on the tolerability of the study treatment, with a planned enrollment of 6–12 patients (if the initially tested dose was tolerated, the sample size was 6; if not, an additional 6 patients would be recruited to receive a reduced dose). In the phase 2 clinical-expansion portion, at a significance level of 0.05, a sample size of 49 could provide a 95% CI half-width of 14% for the ORR, when the ORR point estimate was expected to be 30%. Considering a dropout rate of 10%, approximately 54 patients were required. The 6 patients in the phase 1b portion who received the study treatment at RP2D could proceed to the phase 2 portion; therefore, the sample size of this study was approximately 54–60.

Efficacy and safety outcomes were assessed in all enrolled patients who received at least one dose of study treatment. ORR and DCR were reported with the corresponding 95% CIs calculated using the Clopper-Pearson method. Time-to-event outcomes including DoR, PFS, and OS were estimated using the Kaplan–Meier method, and their two-sided 95% CIs were calculated on the basis of the Brookmeyer-Crowley method. Survival at a given timepoint was estimated with the Kaplan–Meier method, with the 95% CIs calculated using normal approximation. Baseline characteristics and safety evaluations were summarized descriptively.

PK analysis were performed in patients who received at least one dose of study treatment and had at least one post-baseline assessment. Concentration-time profile of SHR-1701, when combined with AG regimen, was drawn with observed values.

For biomarker analyses, ORR, PFS, and OS were analyzed in subgroups by PD-L1 and CA19-9 expressions at baseline. In addition, DEGs in RNA-seq data between responders (patients who had a best overall response of either PR or SD lasting for at least 6 months) and non-responders (patients with SD lasting less than 6 months and PD) were analyzed by using the wald test, with |log2FoldChange (FC)| > 1 and p < 0.05 as the threshold. GO and KEGG enrichment analyses were performed to highlight the most relevant GO terms and signaling pathways associated with DEGs. CIBERSORT was used to deconvolute bulk RNA-seq profiles into cell-type scores,^48^ and the 22 immune cell abundance was estimated between responders and non-responders. For the analysis on pSMAD2/3 and CD8 + T cell, separate Cox regression models were constructed for each biomarker (pSMAD2/3 and CD8⁺ T cell levels), adjusting for responder/non-responder status as a covariate. The hazard ratio of each biomarker for PFS and OS was calculated according to the models. Furthermore, differential gene analysis was performed between patients with PR versus SD and between those with SD versus PD. The logFC values calculated for genes were plotted on a two-dimensional coordinate axis. Subsequently, genes concentrated in different regions of the coordinate axis were subjected to enrichment analysis. The gene set was defined as follows: genes with logFC(SD vs. PR) > 1 and logFC(PD vs. SD) < −1 were considered as the SD-enriched gene set; those with logFC(PD vs. SD) > 1 and |logFC(SD vs. PR)| < 1 were classified as the PD-enriched gene set; and genes with LogFC(SD vs. PD) < −1 and |logFC(PD vs. SD)| < 1 were designated as the PR-enriched gene set. The selected genes had a p < 0.05 in any group. These different gene sets were then analyzed for enrichment on the website https://maayanlab.cloud/Enrichr/. We also explored the mechanisms underlying the differences in length of PFS among PR and SD patients, by comparing genes between patients with SD_Long (SD as the best overall response and a PFS of >5 months) versus those SD_Short (SD and a PFS of <5 months) and between patients with PR_Long (PR and a PFS of >5 months) versus those with PR_Short (PR and a PFS of <5 months). The logFC values of genes were plotted on a two-dimensional coordinate axis, and genes concentrated in different regions were subjected to enrichment analysis. Genes with logFC(SD_Long *vs*. SD_Short) > 1 and logFC(PR_Long vs. PR_Short) > 1 were regarded as common features of long PFS patients, while genes with logFC(SD_Long *vs*. SD_Short) < −1 and logFC(PR_Long *vs*. PR_Short) < −1 were considered as common features of short PFS patients.

## Supplementary information

Revised Supplementary Data_highlighted version

Protocol

Statistical Analysis Plan

## Data Availability

The raw sequencing data reported in this paper have been deposited in the Genome Sequence Archive (GSA) for Human at the China National Center for Bioinformation (CNCB) under accession number HRA014726 (https://ngdc.cncb.ac.cn/gsa-human/). Individual deidentified participant data that underlie the results reported in this article can be requested 24 months after study completion. Researchers should submit a proposal to the corresponding author outlining the reasons for requiring the data. The leading clinical site and sponsor will check whether the request is subject to any intellectual property or confidentiality obligations. A signed data access agreement with the sponsor is required before accessing shared data.
