# Supplementary material for: A phase 1b/2 study of first-line anti-PD-L1/ TGF-βRII fusion protein SHR-1701 combined with nab-paclitaxel and gemcitabine for advanced pancreatic ductal adenocarcinoma
Source: Signal Transduct Target Ther. 2025 Dec 20;10:415. doi: 10.1038/s41392-025-02530-2 (PMC12718313; doi:10.1038/s41392-025-02530-2)
Supplement: Supplementary file 1 — Revised Supplementary Data_highlighted version [file 41392_2025_2530_MOESM1_ESM.docx]

Supplementary Materials for

**A phase 1b/2 study of first-line SHR-1701 combined with nab-paclitaxel and gemcitabine for advanced pancreatic ductal adenocarcinoma**

Ran Xue, Miaoyan Wei, Jiajia Yuan, Zhihua Li, Yuhong Zhou, Zeyun Xue, Yiwen Wu, Hongxia Han, Jun Zhou, Xianjun Yu, Lin Shen

Correspondence to: linshenpku@163.com, yuxianjun@fudanpci.org, or 13366152815@126.com

**This PDF file includes:**

Materials and Methods

Figure S1 to S5

Tables S1 to S7

# Materials and Methods

Definition for dose-limiting toxicities

The following toxicities were considered as dose-limiting toxicities if they are possibly or definitely related to SHR-1701:

1) Non-hematological toxicity of Grade ≥3, with the exception of the following:

a) Nausea, vomiting, diarrhea, or asthenia of Grade ≥3, which ameliorates to Grade ≤2 within 7 days of supportive treatment;

b) Grade 3 ALT/AST increased, which ameliorates to Grade ≤2 within 7 days of treatment;

c) Grade 3 infusion reaction or Grade 3 pyrexia lasting for NMT 6h after supportive treatment;

d) Grade 3 skin toxicity, which ameliorates to Grade ≤2 within 7 days of symptomatic/supportive treatment;

e) Any symptomatic lab abnormality of Grade ≥ 3, which ameliorates to Grade ≤2 within 7 days of symptomatic/supportive treatment;

f) Any blood amylase or lipase laboratory abnormality of Grade ≥ 3 that has no clinical manifestation, with the exception of pancreatitis.

2) Hematotoxicities of Grade ≥ 3, including any of the following:

a) Platelet count decreased of Grade 3, lasting for ≥7 days or with significant clinical hemorrhage symptom(s);

b) Neutropenia of Grade 3 or febrile neutropenia of Grade 3 with infection;

c) Grade 4 neutrophils reduced, lasting for ≥3 days;

d) Any other hematotoxicity of Grade ≥4.

3) Other unexpected, persistent, intolerable toxicities of Grade ≥2, which, in the opinion of SMC, justify the discontinuation of the SHR-1701 treatment.


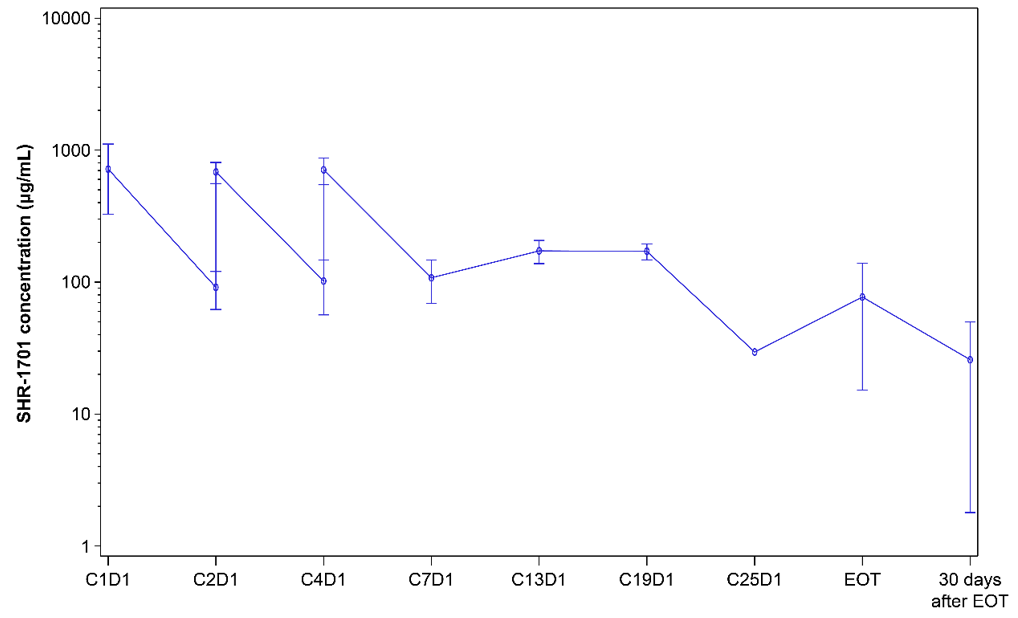


# Figure. S1. Concentration-time profile of SHR-1701, when combined with nab-paclitaxel and gemcitabine

Semi-logarithm mean serum concentration-time profile of SHR-1701 at 30 mg/kg every 3 weeks, when combined with nab-paclitaxel at 125 mg/m^2^ and gemcitabine at 1000 mg/m^2^ on day 1 and 8 of each 3-week cycles.

Error bars represent standard deviation. C, cycle; D, day; EOT, end of treatment.


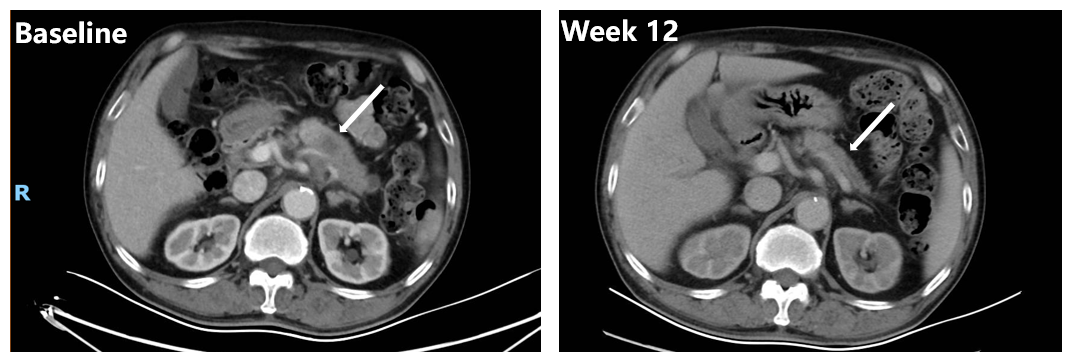


# Figure. S2. Contrast-enhanced CT images of a patient with high PD-L1 expression before and after 12 weeks of treatment

The patient had a PD-L1 CPS of 20 and a TPS of 10%. As shown in the images, the size of the target lesion was substantially reduced after 12 weeks of treatment (indicated by arrows). The patient achieved a best overall response of PR.


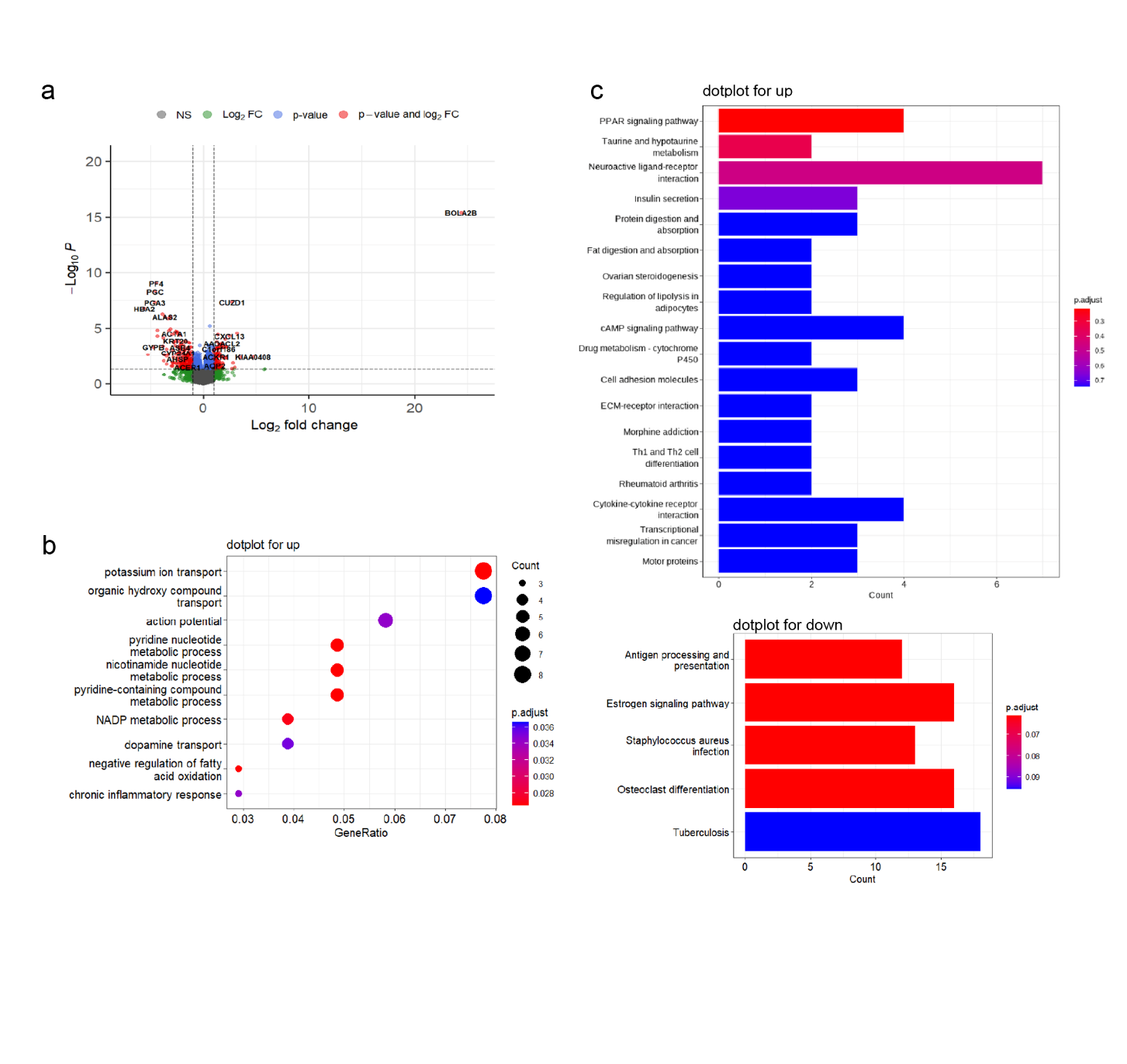


# Figure. S3. Gene and pathway alterations

**a,** Volcano plot of differential expression analysis, with x-axis as log_2_FoldChange and y-axis as -log_10_p-value. Red dots highlighted genes with fold change ≥2 and adjusted p value ≤0.05, green dots highlighted genes with fold change ≥2, blue dots highlighted genes with adjusted p value ≤0.05, and gray dots presented as genes were not significantly expressed between responders (including patients who had a best overall response of either PR or SD lasting for at least 6 months, n=19) and non-responders (including those with SD lasting less than 6 months and PD, n=33). The top 10 DEGs are listed.

**b,** Gene Ontology functional enrichment analysis for DEGs.

**c,** Kyoto Encyclopedia of Genes and Genomes enrichment analysis for DEGs.

DEG, differentially expressed gene, with |log_2_FoldChange| > 1 and p value < 0.05 as the threshold. PR, partial response; SD, stable disease; PD, progressive disease.


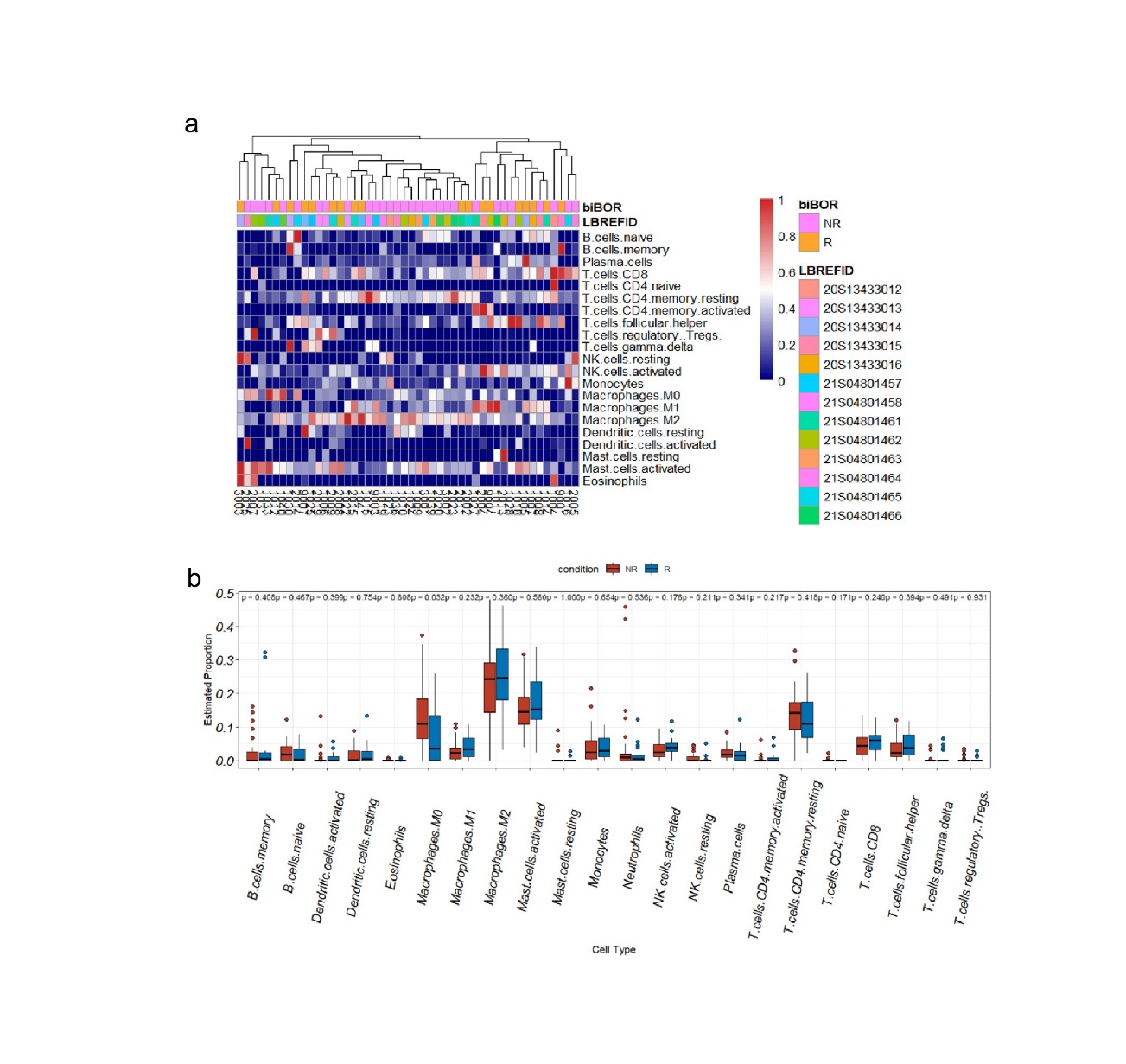


# Figure. S4. Immunophenotype analysis

**a,** Heatmap of all 22 immune cell abundance.

**b,** Comparisons in proportion of different immune cell abundance.

Responders (including patients who had a best overall response of either PR or SD lasting for at least 6 months, n=19) and non-responders (including those with SD lasting less than 6 months and PD, n=33) were included for analysis. The 22 immune cell abundance was estimated, by extracting genes representing these immune cells and activating status. The cell type signature score based on the representing genes in each functional annotation was calculated.

PR, partial response; SD, stable disease; PD, progressive disease.


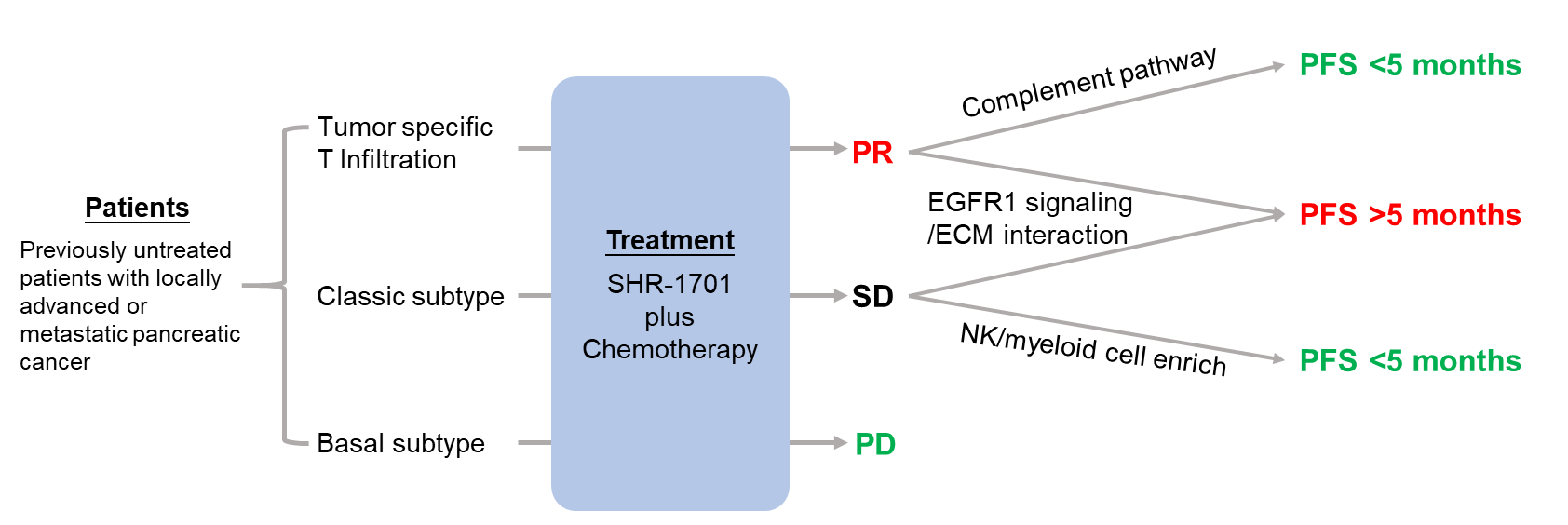


# Figure. S5. Features of patients who could benefit from SHR-1701 plus chemotherapy

PFS, progression-free survival; PR, partial response; SD, stable disease; PD, progressive response.

# Table S1. Subsequent anti-cancer systemic therapy after the end of study treatment

|  | **All patients (N=56)** |
| --- | --- |
| Any | 42 (75.0%) |
| Chemotherapy | 26 (46.4%) |
| Traditional Chinese Patent Medicine | 11 (19.6%) |
| Targeted Therapy | 6 (10.7%) |
| Immunotherapy | 3 (5.4%) |
| Others | 2 (3.6%) |

# Table S2. Safety summary

|  | **All patients (N=56)** |
| --- | --- |
| TEAE of any grade | 56 (100.0%) |
| TEAE of grade 3 or worse | 35 (62.5%) |
| TRAE of any grade | 56 (100.0%) |
| TRAE of grade 3 or worse | 27 (48.2%) |
| SAE | 22 (39.3%) |
| TRSAE | 9 (16.1%) |
| AE leading to death | 8 (14.3%) |
| TRAE leading to death | 1 (1.8%) |
| TRAE leading to treatment discontinuation | 5 (8.9%) |
| TRAE leading to chemotherapy dose reduction | 10 (17.9%) |
| TRAE leading to treatment interruption | 24 (42.9%) |

Data are n (%). TEAE, treatment-emergent adverse event; TRAE, treatment-related adverse event; SAE, serious adverse event; TRSAE, treatment-related serious adverse event.

# Table S3. Treatment-related serious adverse events

|  | **All patients (N=56)** |
| --- | --- |
| Any | 9 (16.1%) |
| White blood cell count decreased | 3 (5.4%) |
| Neutrophil count decreased | 2 (3.6%) |
| Upper gastrointestinal hemorrhage | 2 (3.6%) |
| Alanine aminotransferase increased | 1 (1.8%) |
| Aspartate aminotransferase increased | 1 (1.8%) |
| Platelet count decreased | 1 (1.8%) |
| Diarrhea | 1 (1.8%) |
| Decreased appetite | 1 (1.8%) |
| Immune-mediated hepatitis | 1 (1.8%) |
| Pneumonia | 1 (1.8%) |
| Cerebral hemorrhage | 1 (1.8%) |
| Immune-mediated lung disease | 1 (1.8%) |
| Cardiac failure | 1 (1.8%) |
| Deep vein thrombosis | 1 (1.8%) |

Data are n (%).

# Table S4. Immune-related adverse events

|  | **All patients (N=56)** | |
| --- | --- | --- |
|  | **Any grade** | **Grade 3/4** |
| Any | 25 (44.6) | 7 (12.5) |
| Rash | 17 (30.4) | 1 (1.8) |
| Gingival bleeding | 11 (19.6) | 0 |
| Alanine aminotransferase increased | 7 (12.5) | 1 (1.8) |
| Aspartate aminotransferase increased | 7 (12.5) | 1 (1.8) |
| Gamma-glutamyltransferase increased | 6 (10.7) | 1 (1.8) |
| Diarrhea | 6 (10.7) | 0 |
| Proteinuria | 6 (10.7) | 0 |
| Infusion related reaction | 4 (7.1) | 1 (1.8) |
| Immune-mediated dermatitis | 3 (5.4) | 0 |
| Blood alkaline phosphatase increased | 3 (5.4) | 0 |
| Bilirubin conjugated increased | 2 (3.6) | 0 |
| Immune-mediated enterocolitis | 1 (1.8) | 1 (1.8) |
| Immune-mediated lung disease | 1 (1.8) | 1 (1.8) |
| Immune-mediated hepatitis | 1 (1.8) | 1 (1.8) |
| Cardiac failure | 1 (1.8) | 1 (1.8) |
| Blood bilirubin increased | 1 (1.8) | 0 |
| Blood bilirubin unconjugated increased | 1 (1.8) | 0 |
| Hyperthyroidism | 1 (1.8) | 0 |

# Table S5. PFS and OS in subgroups by PD-L1 and CA19-9 expression

|  | **n** | **PFS, months** | **OS, months** |
| --- | --- | --- | --- |
| **PD-L1 CPS** |  |  |  |
| <1 | 35 | 5.5 (4.2-6.6) | 10.0 (8.8-12.3) |
| ≥1 | 15 | 5.5 (1.2-7.0) | 9.4 (3.3-16.8) |
| <5 | 42 | 5.5 (4.2-5.8) | 9.9 (8.5-11.9) |
| ≥5 | 8 | 6.9 (0.2-15.2) | 14.8 (0.2-24.7) |
| <10 | 45 | 5.5 (4.3-5.8) | 10.0 (8.5-12.0) |
| ≥10 | 5 | 6.9 (0.2-15.2) | 12.8 (0.2-24.7) |
| Not evaluable | 6 | 5.7 (1.5-NE) | 13.6 (5.8-NE) |
| **PD-L1 TPS** |  |  |  |
| <1% | 44 | 5.3 (4.2-6.6) | 9.9 (8.1-12.0) |
| 1-49% | 6 | 6.3 (0.2-15.2) | 18.8 (0.2-24.7) |
| Not evaluable | 6 | 5.7 (1.5-NE) | 13.6 (5.8-NE) |
| **CA19-9** |  |  |  |
| Baseline level |  |  |  |
| ≤1000 U/ml | 30 | 5.7 (4.1-7.1) | 12.7 (9.5-15.8) |
| >1000 U/ml | 26 | 5.3 (4.1-5.8) | 8.1 (6.8-10.9) |

Data are n or median (95% CI).

PFS, progression-free survival; OS, overall survival; PD-L1, programmed cell death-ligand 1; CPS, combined positive score; TPS, tumor proportion score; CA19-9, cancer antigen 19-9; NE, not estimable.

# Table S6. Signature analysis of immune cell abundance and immune infiltration or fibrosis-related signatures between responders versus non-responders

|  | **AUC** | **p value** |
| --- | --- | --- |
| **Immune cell abundance** | | |
| B.cells.naive | 0.57 | 0.234 |
| B.cells.memory | 0.53 | 0.529 |
| Plasma.cells | 0.51 | 0.198 |
| T.cells.CD8 | 0.49 | 0.304 |
| T.cells.CD4.naive | 0.50 | 0.327 |
| T.cells.CD4.memory.resting | 0.47 | 0.451 |
| T.cells.CD4.memory.activated | 0.60 | 0.042 |
| T.cells.follicular.helper | 0.64 | 0.038 |
| T.cells.regulatory..Tregs. | 0.61 | 0.011 |
| T.cells.gamma.delta | 0.56 | 8.79E-03 |
| NK.cells.resting | 0.35 | 0.555 |
| NK.cells.activated | 0.62 | 0.076 |
| Monocytes | 0.47 | 0.404 |
| Macrophages.M0 | 0.36 | 0.555 |
| Macrophages.M1 | 0.47 | 0.081 |
| Macrophages.M2 | 0.52 | 0.238 |
| Dendritic.cells.resting | 0.50 | 0.769 |
| Dendritic.cells.activated | 0.61 | 0.056 |
| Mast.cells.resting | 0.46 | 0.555 |
| Mast.cells.activated | 0.60 | 0.011 |
| Eosinophils | 0.47 | 1 |
| Neutrophils | 0.46 | 1 |
| **I****mmune infiltration or fibrosis-associated signatures** | | |
| pS02Fibromatrix.stemcellniche_score | 0.57 | 0.014 |
| pS15Perictye_score | 0.53 | 0.029 |
| IL.iCAF_score | 0.57 | 0.031 |

Responders (including patients who had a best overall response of either PR or SD lasting for at least 6 months, n=19) and non-responders (including those with SD lasting less than 6 months and PD, n=33) were included for analysis.

Signature scores were calculated: the average expression of genes in the module subtracted by the average expression of a randomly selected set of control genes with similar expression across the samples.^1^

AUC, areas under the curve; PR, partial response; SD, stable disease; PD, progressive disease.

**Reference:**

1. Kieffer Y, Hocine HR, Gentric G, et al. Single-Cell Analysis Reveals Fibroblast Clusters Linked to Immunotherapy Resistance in Cancer. Cancer Discov 2020;10:1330-1351.

# Table S7. Association of pSMAD2/3 and CD8+ T cell with survival.

|  | **PFS** | | **OS** | |
| --- | --- | --- | --- | --- |
|  | **HR (95% CI)** | **p value** | **HR (95% CI)** | **p value** |
| **pSMAD2/3** |  |  |  |  |
| TCP | 1 (0.98-1) | 0.67 | 1 (0.96-1.1) | 0.74 |
| TCSIPER1 | 0.98 (0.93-1) | 0.31 | 1 (0.95-1.1) | 0.35 |
| TCHSCO | 1 (0.99-1) | 0.69 | 1 (0.99-1) | 0.63 |
| ICP | 1 (0.99-1) | 0.17 | 1 (0.99-1) | 0.31 |
| ICSIPER1 | 0.87 (0.77-0.99) | 0.028 | 1.1 (0.97-1.3) | 0.13 |
| ICHSCO | 1 (1-1) | 0.11 | 1 (0.99-1) | 0.45 |
| **CD8+ T cell** |  |  |  |  |
| CD8_total | 1.2 (0.77-1.8) | 0.45 | 1.9 (1-3.5) | 0.052 |

The reference group was non-responder group, including patients who had a best overall response of either SD lasting less than 6 months and PD (n=19). In contrast, the responder group included patients who had a best overall response of either PR or SD lasting for at least 6 months (n=33). Separate Cox regression models were constructed for each biomarker (pSMAD2/3 and CD8+ T cell levels), adjusting for responder/non-responder status as a covariate. The risk of each biomarker for PFS and OS was calculated according to the models.

TCP, proportion of tumor cells exhibiting pSMAD2/3 positivity; TCSIPER1, proportion of tumor cells exhibiting a pSMAD2/3 staining intensity of 1+; TCHSCO, pSMAD H-score in tumor cells; ICSIPER1, proportion of immune cells with a pSMAD2/3 staining intensity of 1+; ICHSCO, pSMAD H-score in immune cells; H-score, histochemical score (defined and calculated as the product of the intensity score and proportion); CD8_total, proportion of CD8-positive cells in total cells; PFS, progression-free survival; OS, overall survival; PR, partial response; SD, stable disease; PD, progressive disease; HR, hazard ratio.
