# Supplementary material for: A phase 1b/2 study of first-line anti-PD-L1/ TGF-βRII fusion protein SHR-1701 combined with nab-paclitaxel and gemcitabine for advanced pancreatic ductal adenocarcinoma
Source: Signal Transduct Target Ther. 2025 Dec 20;10:415. doi: 10.1038/s41392-025-02530-2 (PMC12718313; doi:10.1038/s41392-025-02530-2)
Supplement: Supplementary file 2 — Protocol [file 41392_2025_2530_MOESM2_ESM.pdf]

A Phase Ib/II Clinical Study to Assess the Combination of PD-L1/TGF-βRII antibody (SHR-1701) and Gemcitabine and Albumin-bound paclitaxel as First-line Therapy for advanced/metastatic pancreatic cancer

Protocol No.:	SHR-1701- II-204
Study Phase:	1b/2
Compound No.:	SHR-1701
[redacted]	[redacted]
Version No.:	1.0
Version Date:	May 26, 2020

Sponsor: Jiangsu Hengrui Pharmaceutical Co., Ltd.

38 Huanghe Road, Lianyungang Economic and Technological Development Zone, Lianyugang, Jiangsu 222047

Confidential Statement

The information contained in this protocol is confidential and is for clinical investigator use only, and it may not be disclosed unless required by current laws or regulations. Its copyright is owned by Jiangsu Hengrui Pharmaceutical Co., Ltd. or its subsidiaries and may not be copied or distributed to any person not involved in this clinical study except to those who have signed a confidentiality agreement with Jiangsu Hengrui Pharmaceutical Co., Ltd. or its subsidiaries.

Version History/Revision History

Version No.:	Version Date:	Reason for Amendment and Summary of Changes
1.0	May 26, 2020	NA

Sponsor Protocol Signature Page

I have read and confirm this clinical trial protocol (protocol No.: SHR-1701-II-204, version No.: 1.0, version date: Tuesday, May 26, 2020). I agree to perform my duties in accordance with the laws of China, the Declaration of Helsinki, ICH-GCP, and this protocol.

Sponsor: Jiangsu Hengrui Pharmaceutical Co., Ltd.

Medical Director (Print)	Medical Director (Signature)	Signature Date (MM/DD/YY)

Protocol Signature Page of Principal Investigator (Leading Site)

Protocol Title: A Phase Ib/II Clinical Study to Assess the Combination of PD-L1/TGF-βRII antibody (SHR-1701) and Gemcitabine and Albumin-bound paclitaxel as First-line Therapy for advanced/metastatic pancreatic cancer

Protocol No.: SHR-1701-II-204

Version Number and Date:1.0, May 26, 2020

By signing this protocol signature page, the investigator acknowledges and agrees that:

I have read the above study protocol and its attachments.

I have fully discussed the contents of this protocol with the sponsor Jiangsu Hengrui Pharmaceutical Co., Ltd.

I agree to conduct the study in accordance with this protocol and to perform my duties in accordance with ICH-GCP, local regulations, and other applicable regulations.

This document contains confidential information, which will not be disclosed without written authorization from the sponsor, except to persons directly involved in the conduct of the study or ethical/regulatory review.

I agree to ensure that all staff involved in this study have understood their obligations in fulfilling the above commitments.

Study Sites: ____

______________________	______________________	______________________
Principal Investigator (Printed name)	Principal Investigator (Signature)	Signature Date (MM/DD/YY)

Protocol Signature Page of Principal Investigator (Participating Site)

Protocol Title:A Phase Ib/II Clinical Study to Assess the Combination of PD-L1/TGF-βRII antibody (SHR-1701) and Gemcitabine and Albumin-bound paclitaxel as First-line Therapy for advanced/metastatic pancreatic cancer

Protocol No.: SHR-1701-II-204

Version Number and Date:1.0, May 26, 2020

By signing this protocol signature page, the investigator acknowledges and agrees that:

I have read the above study protocol and its attachments.

I have fully discussed the contents of this protocol with the sponsor Jiangsu Hengrui Pharmaceutical Co., Ltd.

I agree to conduct the study in accordance with this protocol and to perform my duties in accordance with ICH-GCP, local regulations, and other applicable regulations.

This document contains confidential information, which will not be disclosed without written authorization from the sponsor, except to persons directly involved in the conduct of the study or ethical/regulatory review.

I agree to ensure that all staff involved in this study have understood their obligations in fulfilling the above commitments.

Study Sites: ____

______________________	______________________	______________________
Principal Investigator (Printed name)	Principal Investigator (Signature)	Signature Date (MM/DD/YY)

Table of Contents

Table of Contents 6
List of Tables 10
List of Figures 10
Synopsis 12
Trial flow chart 22
Abbreviations 27
1. Introduction: Background and Scientific Rationale of the Study 29
1.1. Study Background 29
1.1.1. Epidemiology and Clinical Treatment of PC 29
1.1.2. Exploratory study of immune checkpoint inhibitors in advanced/metastatic PC 30
1.1.3. Exploration of the roles of PD-L1/TGFβ in advanced tumors 30
1.2. Information of the drug SHR-1701 32
1.2.1. Type of the drug's pharmacologic action and the mechanism of action 32
1.2.2. Pharmacodynamic study 33
1.2.3. Safety pharmacology study 38
1.2.4. Toxicological studies 38
1.2.5. Immunogenicity/immunotoxicity studies 39
1.2.6. Preclinical pharmacokinetic studies 40
1.2.7. Pre-clinical study of receptor occupancy 41
1.3. Progress in clinical studies of SHR-1701 42
1.4. Potential risks and benefits 45
1.4.1. Safety findings in pre-clinical studies 45
1.4.2. Known potential risks 45
1.4.3. Risk control plan 46
1.4.4. Known potential benefits 47
2. Study Objectives and Endpoints 47
2.1. Study Objectives 47
2.2. Study Endpoints 48
3. Study Design 49
3.1. Overall design of the clinical trial 49
3.2. Clinically significant toxicity 51
3.3. Safety Monitoring Committee 52
3.4. PK, immunogenicity and biomarker studies 52

3.4.1. PK blood sampling time points 52
3.4.2. Immunogenicity blood sampling time points 52
3.4.3. Collection of tumor tissue samples 52
4. Selection and withdrawal of subjects 53
4.1. Enrollment Criteria 53
4.2. Exclusion Criteria 54
4.3. Criteria for re-screening 55
4.4. Withdrawal or discontinuation by subjects 56
4.4.1. Criteria for study withdrawal 56
4.4.2. Criteria for discontinuation of study treatment 56
4.4.3. Procedures for withdrawal or discontinuation 57
4.5. Criteria for remain on treatment after disease progression 57
4.6. Early Termination or Suspension of the Study 58
4.7. Definition of End of Study 58
5. study treatment 59
5.1. Overview of the investigational drug 59
5.1.1. Acquisition of the drug 59
5.1.2. Pharmaceutical information 59
5.1.3. Preservation of drugs 59
5.1.4. Mode of drug administration 60
5.2. Dose modification of study drugs 61
5.2.1. Dose modification of SHR-1701 61
5.2.2. Discontinuation and delay of SHR-1701 dose 61
5.2.3. Criteria for dose modification of AG regimen 62
5.3. Duration of treatment with the investigational drug 63
5.4. Management, dispensing, recovery and destruction of drugs 64
5.5. Concomitant medications and concomitant therapies 64
5.5.1. Other antitumor therapies or investigational drugs 64
5.5.2. Treatment of diarrhea and vomiting 65
5.5.3. Vaccine 65
5.5.4. Immunomodulators and corticosteroids 65
5.5.5. Hematopoietic growth factors and blood transfusion 66
5.5.6. Anti-inflammatory therapy 66
5.5.7. Surgery 66
5.5.8. Supportive care 66

5.6. Recommended symptomatic treatment of common AEs 67
5.6.1. Rules for Safety Management of Immuno-oncology Agents 67
5.6.2. Management of infusion reactions 69
5.6.3. Rules for treatment of common AEs of the AG chemotherapy regimen 71
6. Study Procedures 71
6.1. Screening Phase 71
6.2. Treatment period 73
6.3. End-of-treatment/Withdrawal visit 75
6.4. Follow-up period 75
6.4.1. Safety follow-up period 75
6.4.2. Survival follow-up period 76
6.5. Unscheduled Visits 77
7. Study evaluation: 77
7.1. Safety Evaluation 77
7.1.1. Safety parameter 77
7.1.2. Definition of AE 77
7.1.3. Definition of serious adverse events(SAEs) 77
7.1.4. Disease progression and death 78
7.1.5. Immune-mediated AEs 79
7.1.6. Adverse events of special interest 79
7.2. Classification of AEs and SAEs 80
7.2.1. Criteria for judging the severity of adverse events 80
7.2.2. Determination of the correlation between an AE and the investigational drug 80
7.3. Follow-up and report of AEs 81
7.3.1. Follow-up of AEs/SAEs/SIEs 81
7.3.2. Report of serious adverse events 81
7.3.3. Pregnancy reporting 82
7.4. Assessment of Biomarker(s) 82
7.5. Efficacy Evaluation 82
7.5.1. Efficacy parameters 82
7.5.2. Criteria for efficacy assessment 83
7.6. Pharmacokinetic Evaluation 83
7.7. Immunogenicity assessment 84
8. Data analysis/statistical method 84

8.1. Statistical Analysis Plan 84
8.2. Sample Size 84
8.3. Populations for analysis 84
8.4. Statistical Methods 85
8.4.1. General Analysis 85
8.4.2. Safety analysis 85
8.4.3. Pharmacokinetic analysis 86
8.4.4. Efficacy analysis 86
8.4.5. Immunogenicity study 86
8.4.6. Other analyses 86
9. Data Management Method 86
9.1. Data acquisition 87
9.1.1. Completion of Electronic Case Report Form (eCRF) 87
9.1.2. Use of Electronic Data Capture (EDC) system 87
9.2. Data Management 87
9.2.1. eCRF data review 87
9.2.2. Data review meeting and database lock 87
9.2.3. Data archiving 87
9.3. Protocol deviations 87
10. Original documents and acquisition of source data/files. 88
11. Quality Assurance and Quality Control 88
12. Regulations, ethics, informed consent and subject protection 89
12.1. Regulatory considerations 89
12.2. Ethical Norms 89
12.3. institutional review board/independent ethics committee 90
12.4. Informed Consent 90
12.4.1. Informed consent form and other written information required by subjects 90
12.4.2. Informed consent process and records 90
12.5. Confidentiality of Subject Information 90
13. Publication of Study Results 91
14. Reference 92
Annex 1 ECOG PS score 94
Annex2 Cockroft-Gault equation (for calculation of creatinine clearance) 95
Annex 3 New York Heart Association (NYHA) Functional Classification 96
Annex 4 Response Evaluation Criteria in Solid Tumors 97

Annex 5 Prohibited TCM Preparations during the Study 108
Annex6 Bone marrow content(%) of human skeleton 109

List of Tables

Table 1 List of dose levels 14
Table 2 Preliminary study results of the combination therapy of Pembrolizumab and Nivolumab with AG regimen 30
Table 3 Affinity of SHR-1701 and M7824 for PD-L1 protein and TGFβ 33
Table 4 Affinity of SHR-1701 and M7824 for FcRn in a variety of animal species 34
Table 5 Affinity of SHR-1701 and M7824 for human FcγR or C1q 34
Table 6 Binding capacity of SHR-1701 to PD-L1 of a variety of animal species and other human B7 family proteins 35
Table 7 SHR-1701's efficacy in MC38/H-11 mouse colon carcinoma cell xenograft model 37
Table 8 Summary of PK parameters of SHR-1701 administered to cynomolgus monkeys in a single intravenous dose 41
Table 9 Summary of PK parameters of SHR-1701 administered to cynomolgus monkeys in multiple intravenous doses 41
Table 10 Receptor occupancy on cynomolgus monkey T cells after a single intravenous dose of SHR-1701 42
Table 11 Summary of single-dose PK parameters (Mean±SD) of SHR-1701 after the first dose 43
Table 12 Definition of re-confirmed PD after first disease progression 58
Table 13 Dose levels of AG regimen available for dose modification 62
Table 14 Rules for dose modification of the AG regimen on D1 of each cycle 62
Table 15 Rules for dose modification of the AG regimen on D8 of each cycle 63
Table 16 Glucocorticoid induced ARs and recommended preventive and therapeutic measures 69
Table 17 Recommended treatment for SHR-1701 infusion reaction 70
Table 18 PTs included in IMAE analysis for supporting warnings and precautions 79
Table 19 Evaluation of abnormal liver function tests 80
Table 20 Severity criteria of AEs 80
Table 21 Principles for AEs/SAEs/SIEs collection and follow-up period 81

List of Figures

Figure 1 Evaluation of response to M7824 in a phase I clinical study 32
Figure 2 Binding capacity of SHR-1701 with PD-L1 of a variety of animal species and human B7 family protein 35

Figure 3 SHR-1701's binding capacity with MC38/H-11 cell 36
Figure 4 SHR-1701's blocking capability of PD-L1/PD-1 binding........ 36
Figure 5 SHR-1701's effect on body weight of MC38/H-11 mouse colon carcinoma cell tumor-bearing mice 37
Figure 6 SHR-1701 's efficacy in MC38/H-11 mouse colon carcinoma cell xenograft model (by RTV) 38
Figure 7 SHR-1701's efficacy in MC38/H-11 mouse colon carcinoma cell xenograft model(tumor photos)........ 38
Figure 8 T cell receptor occupancy in cynomolgus monkeys administered a single dose of SHR-1701 intravenously 42
Figure 9 Mean drug concentration-time curve (semi-logarithmic scale) of SHR-1701 after the first dose........ 44
Figure 10 PD-L1 RO in a phase I study of SHR-1701 44
Figure 11 TGFβ suppression in a phase I study of SHR-1701 45

Synopsis

**Study Title**	A Phase Ib/II Clinical Study to Assess the Combination of PD-L1/TGF-βRII antibody (SHR-1701) and Gemcitabine and Albumin-bound paclitaxel as First-line Therapy for advanced/metastatic pancreatic cancer
**Protocol No.**	SHR-1701-II-204
**Version No. and Date**	Version 1.0; 2020/5/26
**Study Phase**	1b/2
**Investigational Drug**	SHR-1701
**Sponsor**	Jiangsu Hengrui Pharmaceutical Co., Ltd.
[redacted]	[redacted]
[redacted]	[redacted]
**Study Objectives**	This study consists of 2 phases, Phase 1 (Phase Ib) is for dose-finding of the combination therapy, Phase 2 (Phase II) is for efficacy extension.<br>Primary Objective of Phase Ib:<br>• To evaluate the safety and tolerability of the combination of SHR-1701 and gemcitabine and albumin-bound paclitaxel as first-line therapy for advanced/metastatic pancreatic cancer (PC) patients and determine the recommended phase 2 dose (RP2D) of SHR-1701 in the combination therapy regimen.<br>Secondary Objectives of Phase Ib:<br>• To preliminarily evaluate the efficacy of the combination of SHR-1701 and gemcitabine and albumin-bound paclitaxel as first-line therapy for advanced/metastatic PC patients.<br>• To evaluate the pharmacokinetics (PK) properties of SHR-1701;<br><br>Primary Objective of Phase II:<br>• To evaluate the efficacy of the combination of SHR-1701 and gemcitabine and albumin-bound paclitaxel as first-line therapy for advanced/metastatic PC patients by objective response rate (ORR).<br>Secondary Objectives of Phase II:<br>• Key secondary study objective: To evaluate the efficacy of the combination of SHR-1701 and gemcitabine and albumin-bound paclitaxel as first-line therapy for advanced/metastatic PC patients by OS rates at 9-month.<br>• To evaluate the efficacy of the combination of SHR-1701 and gemcitabine and albumin-bound paclitaxel as first-line therapy for advanced/metastatic PC patients by other efficacy indicators.<br>• To further evaluate the safety and tolerability of the combination of SHR-1701 and gemcitabine and albumin-bound paclitaxel as first-line therapy for advanced/metastatic PC patients;<br>• To evaluate the pharmacokinetics (PK) properties of SHR-1701;<br><br>Exploratory Objectives for Phase Ib and Phase II:<br>• To evaluate the immunogenicity of SHR-1701;<br>• To explore the relationship between biomarkers and clinical efficacy by collecting tumor tissue samples for analysis of biomarkers, including PD-L1 expression of tumor tissue, tumor infiltrating lymphocytes (TILs), TGFβ/pSmad pathway related protein level, tumor micro-environment gene expression, etc.
Study Endpoints	Primary Endpoint of Phase Ib:<br>• RP2D of SHR-1701 in the combination therapy regimen.<br>Secondary Endpoints of Phase Ib:<br>• Occurrence of clinically significant toxicity events, incidence and severity of

	adverse events (AEs) and serious adverse events (SAEs) evaluated per NCI-CTC AE 5.0, vital signs, electrocardiograms (ECG) and lab abnormalities and other safety indicators;<br>• Preliminary efficacy endpoints: effectiveness indicators such as ORR, diseases control rate (DCR), best overall response (BOR), progression-free survival (PFS), time to progression (TTP), duration of response (DoR), OS rates at 6-month, OS rates at 9-month, OS rates at 12-month, overall survival (OS), assessed by investigator per RECIST 1.1;<br>• PK parameters, including C_{trough} and C_{max}, of SHR-1701<br><br>Primary Endpoint of Phase II:<br>• ORR, assessed by investigator per RECIST 1.1.<br>Secondary Endpoints of Phase II:<br>• Key secondary endpoint: OS rates at 9-month;<br>• Other secondary efficacy endpoints: Effectiveness indicators such as DCR, BOR, PFS, TTP, DoR, OS rates at 6-month, OS rates at 12-month, OS, etc., assessed by investigator per RECIST 1.1;<br>• PK parameters, including C_{trough} and C_{max}, of SHR-1701<br><br>Endpoints for Phase Ib and Phase II Exploratory Study:<br>• Evaluation of the immunogenicity of SHR-1701: The positive status of anti-SHR-1701 antibodies (including ADA and Nab) formed during the study period in relation to the baseline will be analyzed in light of the plasma concentration, safety and effectiveness data of SHR-1701.<br>• The relationship between clinical efficacy and biomarkers, including PD-L1 expression of tumor tissue, tumor infiltrating lymphocytes (TIL), TGFβ/pSmad pathway related protein level, tumor micro-environment gene expression, etc., will be explored.
Study Population	Patients naive to systemic therapies for advanced/metastatic PC.
Study Design	This is an open-label, multi-center Phase Ib/II clinical study consisting of two phases: Phase Ib for dose-finding of the combination therapy regimen, and Phase II for efficacy extension. Planned enrollment: A total of 54-60 patients naive to systemic therapies for advanced/metastatic PC will be enrolled.<br>**Phase Ib: Dose-finding stage**<br>This stage is intended to observe and evaluate the safety and tolerability of the combination of SHR-1701 and gemcitabine and albumin-bound paclitaxel as first-line therapy for advanced/metastatic PC patients, and determine RP2D of SHR-1701 in the combination therapy regimen.<br>Dose-finding will be carried out according to a modified “3+3” scheme; the predefined doses of SHR-1701 are 30mg/kg, D1, q3w (i.e., every 3 weeks/21 days), and 20mg/kg, D1, q3w. The dose level 30mg/kg is based on the RP2D of SHR-1701 determined in a phase I clinical trial of SHR-1701 monotherapy. In this study, dose finding for SHR-1701 will start from 30mg/kg, D1, q3w; if the dose is not tolerated, then the dose level of SHR-1701 will be lowered to 20mg/kg, D1, q3w to continue the exploration.<br>The first 6 enrolled subjects will be treated with combination chemotherapy regimen a(SHR-1701 30mg/kg, D1, q3w; gemcitabine 1000mg/m^2, D1, D8, q3w; albumin-bound paclitaxel 125mg/m^2, D1, D8, q3w); if NMT 1(proportion$<$0.33) of the 6 subjects experiences clinically significant toxicity, then the efficacy extension phase (phase II) of the study will be carried out with this dose level;<br>If NLT 2 (proportion$\geq$0.33) of the 6 subjects at the dose level experience clinically significant toxicity, then 6 additional subjects will be enrolled for treatment with combination chemotherapy regimen b (SHR-1701 20mg/kg, D1, q3w; gemcitabine 1000mg/m^2, D1, D8, q3w; albumin-bound paclitaxel 125mg/m^2, D1, D8, q3w). If the number of patients who experience clinically significant toxicity is still NLT 2 (proportion$\geq$0.33), then it is up to the Safety Monitoring Committee (SMC) to determine the dose level/mode of administration for subsequent study after discussion.<br>For details of the dose-finding procedures in Phase Ib study, refer toTable 1.

Table 1 List of dose levels

Dose level	SHR-1701	Combination therapy regimen	Enrollment, n
a	30mg/kg, D1, q3w	Gemcitabine,1000mg/m^2, D1, D8, q3w; Albumin-bound paclitaxel, 125mg/m^2, D1, D8, q3w	n=6
b (If regimen a is not tolerated)	20mg/kg, D1, q3w	Gemcitabine,1000mg/m^2, D1, D8, q3w; Albumin-bound paclitaxel, 125mg/m^2, D1, D8, q3w	n=6

Cycle 1 (21 days) of the co-medication will clinically significant toxicity observation period, in which those subjects who complete 1 cycle of the co-medication will receive safety assessment.
If a subject is administered a total dose of the investigational drug less than 90% of the specified value in Cycle 1 (due to, for example, discontinuation of dosing because of infusion reaction) and experiences no clinically significant toxicity, the subject will be excluded from the calculation of the occurrence of clinically significant toxicity at the specified dose level of the investigational drug; moreover, the subject will be replaced by an additionally recruited subject who will be administered the drug at the current dose level.
If a subject discontinues his/her study treatment during the clinically significant toxicity observation period after the first dose for reason(s) other than clinically significant toxicity, then the subject will be replaced by an additionally recruited subject. During the study period, no upregulation or downregulation of the dose level of SHR-1701 is permitted. Likewise, no regulation of the dose level of any component of the co-medication is permitted during the clinically significant toxicity observation period.
All subjects (n=6) on treatment with current dose level have to complete the clinically significant toxicity observation period prior to the start of the study at the next dose level.
Phase II: Efficacy extension stage
Once the RP2D for SHR-1701 in the combination chemotherapy regimen is determined on the basis of the preliminary safety, PK and efficacy data from phase Ib, the study will proceed to efficacy extension stage and enrollment of subjects will continue until 54 patients are included for treatment at the same dose level. The efficacy and safety of the therapy for the treatment of advanced/metastatic PC will be further explored.

The study consists of a screening period, a treatment period, and a follow-up period.
Screening period: This period is NLT 28 days long, starting from the signing of ICF by subjects to the first dose; it is acceptable to have the subjects to receive imaging examinations within the 28 days prior to the first dose.
Treatment period: Subjects who complete and pass the screening period examinations and evaluations will proceed to treatment period of the study. Each subject will receive treatment with SHR-1701 in combination with chemotherapy (gemcitabine+albumin-bound paclitaxel) in 21-day cycle(s). The treatment continues until disease progression, intolerable toxicity, subject's discontinuation with the study treatment or withdrawal from the study, or, ineligibility of subject to continue the study treatment in the opinion of the investigator, which ever occurs first, or until termination of the study by the sponsor.
During the study period, no upregulation or downregulation of the dose level of SHR-1701 is permitted. Throughout the study treatment process with the exception of the clinically significant toxicity observation period in phase Ib, if the investigator

	determines that a subject cannot tolerate one or two component drugs of the combination therapy regimen, the investigator may, after thorough assessment of the subject's clinical benefits from the study treatment, retain the subject in the study for receiving treatment with another tolerable drug therapy.<br>In the first 12 months of the study treatment period (the first 16 treatment cycles), subjects will receive an imaging evaluation of their tumors every 6 weeks (±7 days); afterwards, subjects will receive an imaging evaluation of their tumors every 3 cycles (i.e., (9 weeks (±7 days)).<br>During the study treatment, if the investigator determines that a subject who shows disease progression for the first time can still benefit from the treatment (without: any symptom and sign of clinical progression, performance status decreased, fast progression of disease, especially at important anatomical parts, such as spinal cord compression), and the subject can tolerate the study treatment and voluntarily wish to stay in the study, the treatment with SHR-1701 alone or in combination with chemotherpay can be continued (for more details, refer to 4.5) until another imageologically-confirmed progression of the disease or the disappearance of clinical benefits in the subject in the opinion of the investigator(whichever occurs first).<br>Follow-up period: The follow-up period can be divided into safety follow-up period and survival follow-up period. The safety follow-up period starts from the last study treatment and ends 90 days after the last dose; during this period, the subjects are followed up once every 30 days (±7 days). At Safety Follow-up Visit 1, the subjects should go to the study site for assessment; Safety Follow-up Visits 2 and 3 can be done by telephone. At the end of the safety follow-up period, the subjects proceed to the survival follow-up period. The survival follow-up period lasts until subject's death, lost to follow-up, withdrawal of ICF or termination of the study by the sponsor. During the period, subjects are followed up once per month by telephone other valid method for collecting survival information and subsequent treatment information. Subjects without radiologically-confirmed disease progression should receive imaging evaluation at the efficacy evaluation frequency specified in the study protocol until disease progression, death, lost to follow-up, withdrawal of ICF, the start of other antitumor therapy or termination of the study by the sponsor.<br>During the study, safety data will be collected continually. Safety and tolerability assessment: The occurrence and severity of AEs will be determined according to CTC AE v5.0. During the study, subjects will be continually evaluated by laboratory tests, vital signs measurements, ECOG score, physical examination, ECG examinations and AEs.<br>All subjects will be collected PK, immunogenicity, and tumor tissue samples for PK and immunogenicity assessment of SHR-1701 and analysis of biomarkers.
Clinically significant toxicity	Severity of adverse events will be graded according to NCI CTCAE Ver. 5.0. At Phase Ib dose-finding stage, the occurrence of any of the following toxic and side reactions in the clinically significant toxicity observation period (Cycle 1 (21days) of the co-medication) that is possibly or definitely related to SHR-1701 will be deemed as clinically significant toxicity:<br>1) Non-hematological toxicity of Grade ≥3, with the exception of the following:<br>a) Nausea, vomiting, diarrhea, or asthenia of Grade ≥3, which ameliorates to Grade ≤2 within 7 days of supportive treatment;<br>b) Grade 3 ALT/AST increased, which ameliorates to Grade ≤2 within 7 days of treatment;<br>c) Grade 3 infusion reaction or Grade 3 pyrexia lasting for NMT 6h after supportive treatment;<br>d) Grade 3 skin toxicity, which ameliorates to Grade ≤2 within 7 days of symptomatic/supportive treatment;<br>e) Any symptomatic lab abnormality of Grade ≥ 3, which ameliorates to Grade ≤2 within 7 days of symptomatic/supportive treatment;<br>f) Any blood amylase or lipase laboratory abnormality of Grade ≥ 3 that has no clinical manifestation, with the exception of pancreatitis.

	2) Hematotoxicities of Grade ≥ 3, including any of the following:<br>g) Platelet count decreased of Grade 3, lasting for ≥7 days or with significant clinical hemorrhage symptom(s);<br>h) Neutropenia of Grade 3 or febrile neutropenia of Grade 3 with infection;<br>i) Grade 4 neutrophils reduced, lasting for ≥3 days;<br>j) Any other hematotoxicity of Grade ≥4.<br><br>3) Other unexpected, persistent, intolerable toxicities of Grade ≥2, which, in the opinion of SMC, justify the discontinuation of the SHR-1701 treatment.
Study drugs and administration methods	On D1 of each cycle, SHR-1701 is administered intravenously first and, after an interval of at least 30min, albumin-bound paclitaxel is administered, finally gemcitabine is administered.<br>• SHR-1701 is administered intravenously at 30 mg/kg/dose or 20 mg/kg/dose (depending on the stage of the Phase Ib study) in 30-60min, q3w.<br>• Gemcitabine is administered intravenously at 1000 mg/m^2/dose, in 3-week cycles (on D1 and D8 of each cycle) by following the IFU of the drug.<br>• Albumin-bound paclitaxel is administered intravenously at 125mg/m^2/dose, in 3-week cycles (on D1 and D8 of each cycle), by following the IFU of the drug.<br>The combination therapy of SHR-1701 and gemcitabine and albumin-bound paclitaxel continues until disease progression, intolerable toxicity, subject's discontinuation with the study treatment or withdrawal from the study, or, ineligibility of subject to continue the study treatment in the opinion of the investigator, which ever occurs first, or until termination of the study by the sponsor.
Enrollment Criteria	Subjects have to meet all of the following criteria for inclusion<br>1. Histologically or cytologically diagnosed with PC, with evidence(s) of unresectable locally advanced or distant metastasis disease, which is histologically or cytologically verified to be duct adenoma or acinic cell carcinoma.<br>2. Naive to systemic treatment for advanced/metastatic PC; disease progression after more than 6 months of past neoadjuvant treatment or postoperative adjuvant treatment. Palliative radiotherapy is permitted; however, the study medication cannot be started in less than 2 weeks of the completion of the palliative radiotherapy. Previous treatment with anti-tumor TCM preparation(s) is permitted; however, such treatment has to be discontinued at least 2 weeks prior to the start of the study medication.<br>3. 18 - 70 year-old, male or female.<br>4. ECOG PS score 0 - 1.<br>5. Life expectancy ≥ 12 weeks.<br>6. All toxicities from previous anti-tumor therapy or surgical procedures have ameliorated to Grade 0 - Grade 1 (per NCI CTCAE 5.0) or to levels stipulated in eligibility criteria. Excluding other toxicities (e.g., alopecia, lassitude, hearing injury, etc.) which, in the opinion of investigator, pose no safety risks to the patient.<br>7. With at least 1 measurable disease that meets RECIST 1.1 criteria.<br>8. Adequate organ and bone marrow functions, meeting the following criteria (patients need to avoid corrective treatment within the 14 days prior to routine hematology):<br>a) WBC≥ 3,000/mm^3 (3 × 10^9/L);<br>b) ANC≥ 1,500/mm^3 (1.5 × 10^9/L);<br>c) Lymphocyte count ≥ 500/mm^3 (0.5 × 10^9/L);<br>d) PLT ≥ 100,000/mm^3(100 × 10^9/L);<br>e) Hb ≥ 10 g/dL (100 g/L);<br>f) Serum albumin ≥ 2.9 g/dL;<br>g) Serum creatinine ≤ 1.5×ULN or creatinine clearance ≥ 50 ml/min;<br>h) TBIL ≤ 1.5×ULN;<br>i) AST/SGOT or ALT/SGPT ≤ 3×ULN, or ≤ 5×ULN for patients with

	metastases to liver;<br>j) INR ≤ 1.5, PT and APTT ≤ 1.5×ULN;<br>k) Protein urine <2+; if protein urine is ≥2+, then the patient's 24-h urine protein has to be ≤1g to be eligible.<br>9. Availability of fresh or archived tumor tissue samples, preferably fresh ones. Formalin-fixed, paraffin-embedded tumor tissue blocks or unstained tumor specimens, 5-10 sections.<br>Note: if the above-mentioned tumor tissue specimens are unavailable, the sponsor's medical monitor has to be consulted to determine the patient's eligibility. For the harvesting/collection and disposal procedures of tumor specimens, refer to Laboratory Manual.<br>10. Female subjects of childbearing age have to be tested negative in a serum pregnancy test within 7 days prior to the start of study medications and willing to take a medically recognized effective contraceptive measure (e.g., intra-uterine device, contraceptives or condom) during the study and within 3 months after the last dose of the study medications to be eligible for the study; male subjects who have female partners of childbearing age should be surgically sterilized or willing to take effective contraceptive measure(s) during the study and within 3 months after the last dose of the study medications.<br>11. A signed ICF, stating that the subject is willing and able to abide by the scheduled follow-up visits, study treatment, laboratory investigations and other study procedures.
Exclusion Criteria	A subject will be excluded from the study if he/she meets any of the following criteria:<br>1. Previous use of TGFβ inhibitor, anti-PD-1/PD-L1 antibody, anti-PD-L2 antibody, anti-CD137 antibody, anti-CTLA-4 antibody or other drug/antibody that acts on T-cell costimulatory signaling or checkpoint pathway.<br>2. Previous allergy to study medications or any excipient of them, or previous serious anaphylactic reaction to other mAb.<br>3. Any of the following circumstances:<br>a) Any major operations (with the exception of diagnostic biopsy) within 28 days prior to the first dose of the study medications.<br>b) Use of any immunosuppressants (with the exception of corticosteroids nasal spray and inhaler, or systemic steroids at physiological dose (i.e., prednisone at NMT 10 mg/d, or other corticosteroids at equivalent physiological dose)) within 7 days prior to the fist dose of the study medications.<br>c) Use of any immunomodulators within 14 days prior to the first dose of the study medications.<br>d) Inoculation with any live attenuated vaccines within 28 days prior to the first dose of the study medications or within 60 days of the end of treatment with the study medications.<br>4. Previous or present metastasis to CNS.<br>5. Risk of any symptomatic, life-threatening complications (including uncontrolled effusion of moderate or above amount into pleural space, pericardium, abdominal cavity) of advanced disease that have disseminated to viscera.<br>6. Presence of any active autoimmune diseases or expected relapse of such diseases (including but not limited to: autoimmune hepatitis, interstitial pneumonitis, uveitis, enteritis, hepatitis, hypophysitis, vasculitis, nephritis, hyperthyroidism, hypothyroidism[with the exception of those whose condition can be controlled by hormone replacement therapy]; patients who have dermatoses(e.g., vitiligo, psoriasis, etc.) alopecia, type I diabetes mellitus that require no systemic treatment, or in their childhood suffered from asthma which has completely resolved in their adulthood and requires no intervention can be included; however, patients who have asthma that

	requires bronchodilator for medical intervention should not be included).<br>7. Presence/history of other active malignant tumors within 5 years prior to the study, with the exception of cutaneous basal cell carcinoma or squamous cell carcinoma that have been cured by topical therapy, superficial bladder cancer, carcinoma in situ of cervix, carcinoma in situ of breast ductal and thyroid papillary carcinoma.<br>8. HIV infection or AIDS, treatment naïve active hepatitis (hepatitis B, defined as: HBsAg+, HBV-DNA ≥ 500 IU/ml, plus hepatic function abnormal; hepatitis C, defined as: HCV-Ab +, HCV-RNA > LLOD of analytical method, plus hepatic function abnormal) or coinfection of HBV and HCV concurrently.<br>9. Presence of clinically significant acute or chronic pancreatitis.<br>10. Presence of other clinically significant acute or chronic infections (determined by the investigator according to patient's history of exposure to source of infection, etiology test positive results, clinical symptom(s), sign(s), imaging finding(s), and the need for clinical intervention).<br>11. Presence/history of any of the following clinically significant cardio-/cerebro-vascular diseases within 6 months prior to the study: myocardial infarction/brain infarction/hematencephalon, serious/unstable angina, congestive heart failure (cardiac dysfunction of severity ≥ NYHA Class 2) and clinically significant supraventricular or ventricular arrhythmia that requires clinical intervention.<br>12. Systemic use of any antibiotics for ≥ 7 days within 4 weeks prior to the first dose; pyrexia >38.5°C of unknown cause during the screening period/prior to the first dose (patients with pyrexia attributable to tumor may be included at the investigator's discretion).<br>13. Known history of homologous organ transplant or history of homologous hematopoiesis stem cell transplant.<br>14. Participation in clinical trial of any other drug within 4 weeks or 5 times the half-life of the drug of the previous clinical trial after the last dose of the said drug, whichever is longer, prior to the fist dose of the current study.<br>15. Female patients in lactation period. Breast feeding have to be discontinued after the administration of SHR-1701 for treatment due to the unknown risk of the drug to baby.<br>16. Known history of abuse of psychotropics abuse or doping.<br>17. Other serious physical or psychiatric disorders or lab abnormalities that may increase the risk of the patient's participation in the study, or interfere with the study results, and other conditions which, in the opinion of the investigator, render the patient unsuitable for the study.
Sampling for PK study	Below are the blood sampling time points for PK study of all subjects:<br>One (1) sampling is to be performed within 0.5h before dosing and within 10min after dosing of SHR-1701 on C1D1, C2D1, C4D1; within 0.5h before dosing of SHR-1701 on C7D1 and on D1 after every 6 cycles beyond, on the date of the subject's end of treatment, and after 30 days (±7 days) of the last dose. The blood sampling time windows after the end of dosing do not include the time needed for tube cleaning. At each time point, 3.5 mL of venous blood will be collected into a serum separator tube for PK study of SHR-1701. PK blood sampling should be carried out per the study schedule; if necessary, however, it is acceptable to collect unscheduled PK blood samples for PK analysis.
Sampling for immunogenicity study	Below are the blood sampling time points for immunogenicity study of all subjects:<br>One (1) sampling is to be performed within 0.5h before dosing of SHR-1701 on C1D1, C2D1, C4D1, C7D1, and on D1 of cycles beyond C7 at an interval of 6 cycles, on the subject's end of treatment day, and after 30 days (±7 days) of the last dose. At each time point, 5 mL of venous blood will be collected into a serum separator tube for ADA and Nab detection. Immunogenicity blood sampling should be carried out per the study schedule; if necessary, however, it is acceptable to collect unscheduled immunogenicity blood samples for immunogenicity analysis.

Tumor Tissue Biomarkers	Tumor tissue samples will be collected from subjects prior to the first dose for biomarkers (including PD-L1 expression, TIL, TGFβ/pSmad pathway-related protein, tumor micro-environment gene expression, etc.) studies to explore their relationship with the drug's efficacy.<br>For sample harvesting/collection, treatment, storage, and transportation procedures, refer to Laboratory Manual.
Criteria for study withdrawal	Reasons for withdrawal from the study may include:<br>• Withdrawal of ICF and/or refusal to receive further follow-up visits by subject;<br>• Other conditions which, in the opinion of the investigator, justify the withdrawal of the subject from the study; for example, the subject is imprisoned, quarantined, or incapacitated from free expressing his/her will;<br>• Lost to follow-up;<br>• Death of subject;<br>• The study is terminated by the sponsor.
Criteria for discontinuation of study treatment	Discontinuation of study treatment does not mean withdrawal from the study. Subjects who discontinue study treatment must continue to complete the remaining study visits as required by the protocol. Study medication has to be discontinued in subject in any of the following cases:<br>• The subject asks to discontinue his/her treatment with study medication;<br>• The subject's response evaluation results meet the criteria for disease progression, and the subject fails the criteria for remain on treatment after disease progression;<br>• Pregnancy in female subjects during the study;<br>• Intolerable toxicity in subject despite dose modification, or any AE, laboratory abnormality or other medical condition that makes the investigator believe further treatment is not in line with subject's best interest;<br>• General deterioration of health status that prevents continued participation in the study;<br>• Any significant protocol deviation (e.g., enrollment of ineligible subject) found during the study and confirmed by the sponsor;<br>• Termination of the study by the sponsor;<br>• Other reasons which, in the opinion of th investigator, render it impossible to carry on with the study treatment.
Determination of Sample Size	**Phase Ib: Dose-finding stage**<br>Sample size: about 6-12 subjects.<br>**Phase II: Efficacy extension stage**<br>When sample size is 49 subjects, exact test at the significance level(α) of 0.05 will be able to provide a 95% CI [16%,44%] for the ORR of the Combination Therapy Group (SHR-1701 + gemcitabine + albumin-bound paclitaxel); the accuracy of ORR (half width of the CI is 14%) is 30%±14%, taken into consideration a dropout rate of 10%, it is estimated that 54 subjects will be enrolled.<br>For the key secondary endpoint (OS rates at 9-month), this sample size is also able to detect a difference of 20% with 80% power, i.e. 50% vs. 70%.<br>Through the use of a dose level identical to that at Stage 2 and in consideration of that the 6 subjects enrolled at Stage 1 can be included in the sample size of Stage 2, the total sample size of this study is about 54-60 subjects.
Data analysis/statistical method	■ Populations for analysis<br>The following analysis sets will be involved in this study:<br>▪ Full analysis set (FAS): All subjects who are enrolled and administered at least a dose of the investigational drug are included. This set will be used for effectiveness analysis.<br>▪ Safety set (SS): the same with FAS. This set is the primary analysis population for safety analysis of this study.<br>▪ Clinically significant toxicity analysis set: All subjects who receive the

	co-medication to the end of Cycle 1(21 days in total) or discontinue their treatment because of the occurrence of clinically significant toxicity are included in this set.<br>▪ Per protocol set (PPS): PPS is a subset of FAS; all subjects with important protocol deviations that have been determined to have significant impact on efficacy will be excluded from this set. The list of subjects to be included in or excluded from PPS should be reviewed and determined by the sponsor and investigator before locking of database.<br>▪ Evaluable Set (ES): ES is a subset of FAS; by definition, it includes all enrolled subjects who have been administered at least 1 dose of the investigational drug and subjected to at least 1 post-baseline tumor assessment.<br>▪ PK analysis set (PKAS): All enrolled subjects who have been administered at least 1 dose of the investigational drug and have post-dose PK evaluation data constitute the PKAS of this study.<br>▪ Immunogenicity Analysis Set: All subjects who are enrolled in the study and receive the investigational drug at least once, and have baseline and at least one post-baseline immunogenicity evaluation data.<br><br>■ General Analysis<br>In this study, unless otherwise specified, the data will be summarized using descriptive statistics according to the following general principles.<br>Measurement data are summarized by mean, standard deviation (SD), median, maximum, and minimum; count data are summarized by frequency number and percentage; time-event data are subjected to Kaplan-Meier estimation of survival rate and plotting of survival curve; plasma concentration data are summarized by geometric mean, geometric SD, geometric coefficient of variation (CV), mean, SD, CV, median, maximum, and minimum. If necessary, corresponding 95% CIs are provided for the above-mentioned analyses.<br>■ Safety analysis<br>Safety analysis will be based on SS (by actual dose group); clinically significant toxicity analysis is based on clinically significant toxicity analysis set. According to Henrui's reporting SOPs, safety analysis is limited to summary of descriptive statistics, including but not limited to the following:<br>◆ Analysis of patient discontinuation, dose reduction or suspension due to AEs;<br>◆ Summary of (all-cause and treatment-related) adverse events (AEs);<br>◆ Occurrence and severity of (all-cause and treatment-related) AEs.<br>◆ Summary of details of SAEs;<br>◆ Analysis of relatedness of AEs;<br>◆ Occurrences of abnormal laboratory indexes, vital signs, ECG data.<br>■ Pharmacokinetic analysis<br>Pharmacokinetic analysis is based on PK analysis set. PK parameters such as C_{trough} and C_{max} will be subjected to statistical description (number of subjects, mean, SD, median, minimum and maximum) by dose group and scheduled blood sampling time point. Drug concentration-time profiles will be plotted for individual subjects based on their actual sampling time; mean and/or median drug concentration-time curve (linear scale and logarithmic scale) will be plotted for all subjects by dose group and scheduled sampling time.<br>■ Efficacy analysis<br>Analysis of effectiveness will be based o FAS, PPS and ES.<br>ORR, DCR and clinical benefit rate (CBR) will be estimated by Clopper-Pearson method and their 95% CIs will be listed.<br>For analysis of time-event data (PFS, TTP, DoR and OS), survival curves will be plotted by Kaplan-Meier method and median survival time will be estimated and, if necessary, two-sided 95% CI of overall median time will be estimated.<br>■ Immunogenicity study<br>Immunogenicity analysis will be based on immunogenicity analysis set. Positive rate of

	anti-SHR-1701 antibodies, proportion of anti-SHR-1701 antibodies with neutralizing activity, and correlation between anti-SHR-1701 antibody and the drug's Ctrough, safety and effectiveness will be summarized and analyzed.<br>■ Other analyses<br>The relation between SHR-1701 related solid tumor markers, such as PD-L1 expression level, TIL, TGFβ/pSmad pathway-related protein, tumor micro-environment gene expression in tumor tissue samples and efficacy will be analyzed.

Trial flow chart

Study Procedures	Screening Phase D-28 ~ D-1	Screening Phase D-7 ~ D-1	Cycle 1 D1 ± 3	Cycle 1 D8	Cycle 2 and beyond D1 ± 3	Cycle 2 and beyond D8	End of Treatment Visit[24]	Follow-up period (after end of treatment) Safety follow-up[25]	Follow-up period (after end of treatment) Survival follow-up[26]
Signing of ICF[1]	×								
Demographic data	×								
Tumor diagnosis/previous anti-tumor therapy/prior medical history and treatment history[2]	×								
Height		×							
Body weight[3]		×	×		×		×	×	
ECOG PS score[4]		×	×	×	×	×	×	×	
Vital signs[4]		×	×	×	×	×	×	×	
Physical examination[5]		×	×	×	×	×	×	×	
Virology test[6]	×								
Routine hematology[7]		×	×	×	×	×	×	×	
Routine urinalysis[8]		×	×	×	×	×	×	×	
Fecal occult blood[9]		×	×		×		×	×	
Blood chemistry panel[[10]		×	×	×	×	×	×	×	
Coagulation function[11]		×	×		×		×	×	
Thyroid function[12]		×	×		×		×	×	
Blood amylase and lipase [13]		×							
12-lead ECG[14]		×	×		×		×	×	
Echocardiography[15]		×					×		
Pregnancy test[16]		×					×		
Serum CA19-9 [17]		×	×				×	×	
Tumor imaging[18]	×				×		×		
Study treatment[19]			× ----------------	×	× ----------------	×			

Study Procedures	Screening Phase		Cycle 1		Cycle 2 and beyond		End of Treatment Visit[24]	Follow-up period (after end of treatment)	
	D-28 ~ D-1	D-7 ~ D-1	D1 ± 3	D8	D1 ± 3	D8		Safety follow-up[25]	Survival follow-up[26]
Collection of immunogenicity and PK blood samples[20]			×		×		×	×	
Collection of tumor tissue samples[21]	×								
Adverse events[22]	× --				------- ×			×	
Concomitant medications/concomitant therapies[23]	× --				------- ×			×	

Note:

[1] Save for the tumor imaging examination(s) and tumor tissue biopsies available in the specified time limit prior to the first dose, no procedures of the clinical trial should be carried out without the signed ICF from the subject. Subjects who have failed previous screening are allowed to be re-screened in this study; to take part in the re-screening, the subjects must re-sign the ICF and be re-assigned a new Subject Number in advance.

[2] Prior medical history and treatment history: including tumor history (history of tumor diagnosis, surgical procedures, radiotherapy, chemotherapy, etc.) and history of other comorbidities. Tumor diagnosis should include: available histological diagnosis results, pathological typing, histological grading, clinical staging, initial diagnosis time and gene mutation detection results (if applicable), etc., before enrollment.

[3] Body weight: Body weight will be taken within 7 days prior to the first dose, before dosing on D1 of each treatment cycle, at the end of treatment/withdrawal from the study, and at Visit 1 in the safety follow-up period.

[4] ECOG score and vital signs (pulse rate, respiratory rate, body temperature and blood pressure): These will be recorded within 7 days prior to the first dose, before dosing on D1 and D8 of each treatment cycle, at the end of treatment/withdrawal from the trial, and at Visit 1 in the safety follow-up period.

[5] Physical examination: A thorough physical examination (covering general conditions, head and face, skin, lymph nodes, eyes, ears, nose and throat, oral cavity, respiratory system, cardiovascular system, abdomen, reproductive-urinary system, musculoskeletal system, nervous system and mental state, etc.) will be carried out within 7 days prior to the first dose, at the end of treatment/withdrawal from the trial, before dosing on D1 and D8 of each cycle, and at Visit 1 in the safety follow-up period, and at times of clinically indicated.

[6] Virology tests: HBsAg, HBsAb, HBeAg, HBeAb, HBcAb, HBV DNA (if "test of (five) hepatitis B serologic markers" suggests HBV infection [HBsAg+] or history of prior HBV infection, then HBV DNA must be determined quantitatively), HCV-Ab (if the test result is positive or suggesting history of previous HCV infection, then HCV-RNA must be determined quantitatively), and HIV-Ab. These tests should be conducted within 28 days prior to the first study treatment.

[7] Routine hematology: RBC, hemoglobin (Hb), platelet count (PLT), WBC, ANC and lymphocyte count; routine hematology should be examined within 7 days prior to the first dose, before dosing on D1 and D8 of each treatment cycle (if corresponding baseline examination has been conducted within 7 days prior to the first dose, then no re-test has to be conducted), at the end of treatment/withdrawal from the study, and at Visit 1 in safety follow-up period.

[8] Routine urinalysis: WBC, RBC, protein urine. Routine urinalyses should be carried out within 7 days prior to the first dose (if protein urine ≥2＋,it is necessary to

perform additional quantitative determination of 24-h urine protein), before dosing on D1 and D8 of each treatment cycle (if corresponding baseline analysis had been performed within 7 days prior to the first dose, it is not necessary to repeat), at the end of treatment/withdrawal from the study, and at Visit 1 in safety follow-up period.

[9] Fecal occult blood: Fecal occult blood detections should be performed within 7 days prior to the first dose (if fecal occult blood result is +, a retest should be conducted; if the result is still + in the retest, the subject might have active hemorrhage of digestive tract and should receive gastrointestinal endoscopy at the investigator's discretion), before dosing on D1 of each treatment cycle (if corresponding baseline test had been conducted within 7 days prior to the first dose, then no retest has to be conducted), at the end of treatment/withdrawal from the study, and at Visit 1 in the safety follow-up period.

[10] Blood chemistry panel: ALT, AST, γ-GT, total bilirubin (TBIL), direct bilirubin (DBIL), alkaline phosphatase (AKP), blood urea nitrogen (BUN) or urea (preferably BUN), total protein (TP), albumin (ALB), creatinine (Cr), blood sugar (GLU), K^+, Na^+, Ca^{2+}, Mg^{2+}, Cl^-; these indicators should be determined within 7 days prior to the first dose, before dosing on D1 and D8 of each treatment cycle (if corresponding baseline examinations have been done within 7 days prior to the first dose, then no re-examination has to be conducted), at the end of treatment/withdrawal from the trial, and at Visit 1 in the safety follow-up period.

[11] Coagulation function: activated partial thromboplastin time (APTT), prothrombin time (PT), thrombin time (TT), fibrinogen (FIB), International Normalized Ratio (INR); coagulation function should be tested within 7 days prior to the first dose, before dosing on D1 of each treatment cycle (if corresponding baseline tests had been conducted within 7 days prior to the first dose, it would be unnecessary to repeat the tests scheduled to be performed at this time point), at the end of treatment/withdrawal from the trial, and at Visit 1 in the safety follow-up period.

[12] Thyroid function: Thyroid function tests should cover serum thyroid-stimulating hormone (TSH), free triiodothyronine (FT3), free thyroxine (FT4); if FT3, FT4 are not available, it is acceptable to use (T)T3, (T)T4 in place of them; the test should be conducted within 7 days prior to the first dose, before dosing on D1 of each treatment cycle (if corresponding baseline tests had been conducted within 7 days prior to the first dose, it would be unnecessary to repeat the tests scheduled to be performed at this time point), at the end of treatment/withdrawal from the trial, and at Visit 1 in the safety follow-up period.

[13] Blood amylase and lipase: Blood amylase and lipase test should be performed once within 7 days prior to the first dose, and at times of clinically indicated during the study.

[14] 12-lead ECG: QT, QTc and P-R interval should be scrutinized during ECG examinations. ECG examinations should be performed within 7 days prior to the first dose, before dosing on D1 of each treatment cycle (if ECG examination had been done in the screening period within 7 days prior to the first dose, then it would be unnecessary to perform the examination scheduled to be done at this time point), at the end of treatment/withdrawal from the trial, and at Visit 1 in the safety follow-up period.

[15] Echocardiography: Echocardiographic examinations should be done within 7 days prior to the first dose and at the end of treatment/withdrawal from the trial and at times of clinically indicated during the study.

[16] Pregnancy test: A serum pregnancy test is scheduled for female subjects of childbearing age. It should be done within 7 days prior to the first dose.

[17] Serum CA19-9 tests: Tests should be performed before dosing on C1D1 (if corresponding baseline test had been conducted within 7 days prior to the first dose, it would be unnecessary to repeat the test scheduled to be performed at this time point), in every other treatment cycle (C1D1, C3D1, C5D1…), at the end of treatment/withdrawal from the trial, and at Visit 1 in the safety follow-up period.

[18] Imaging examination of tumor: The examination should be done by chest, abdominal, and pelvic CT or MRI. Subjects with suspected or diagnosed metastasis to brain should receive brain MRI (or CT if MRI is impossible); bone scan is to be done only when clinically indicated and within 42 days prior to the first dose.

- ✓ In the screening period, tumor assessment can be done with imaging examination results obtained within 4 weeks prior to the first dose of the study medications even before the subject's signing of ICF and the results are usable so long as they are up to the requirements in RECIST 1.1.
- ✓ Imaging examinations are performed once every 6 weeks (±7 days) in the first 12 months and once every 9 weeks (±7 days) afterwards in the study treatment period and as appropriate in case a new lesion is suspected; if a subject withdraws/is withdrawn from the study for any reason, he/she needs to receive imaging

examination in a timely manner (in ±4 weeks of his/her withdrawal; if the subject's previous examination is no more than 4 weeks away from his/her discontinuation of treatment, then no re-examination is required at his/her withdrawal). The conditions for the imaging examinations should be the same with those for baseline examination (including scan slice thickness, contrast agent, etc.). The acceptable time window for an imaging examination is the scheduled date±7 days; it is acceptable to perform unscheduled imaging examination at times of suspected disease progression (e.g., symptomatic deterioration).

- ✓ For subjects in whom no radiographic progression is observed during the safety follow-up and survival follow-up periods, imaging examinations should be conducted at the same frequency if possible until disease progression or the start of other antitumor therapy.
- ✓ Except for those who have radiologically confirmed disease progression, all subjects who discontinue the study treatment for other reasons should receive imaging examinations at protocol specified frequency until disease progression, the start of new antitumor therapy, or death is registered.
- ✓ For patients who remain in the study after disease progression, imaging examinations should be continued at the above-mentioned frequency until their discontinuation of study treatment, the start of new antitumor therapy, lost to follow-up, or death (whichever occurs first).

[19] Study treatment: Study treatment is administered once on D1 of each 21-day cycles (for detailed administration method, refer to 5.1.4):

[20] Collection of immunogenicity and PK blood samples:

Collection of PK blood samples: One (1) sampling is to be performed within 0.5h before dosing and within 10min after dosing of SHR-1701 on C1D1, C2D1, C4D1; within 0.5h before dosing of SHR-1701 on C7D1 and on D1 after every 6 cycles beyond, on the date of the subject's end of treatment, and after 30 days (±7 days) of the last dose. The blood sampling time windows after the end of dosing do not include the time needed for tube cleaning. At each time point, 3.5 mL of venous blood will be collected into a serum separator tube for PK study of SHR-1701.

Collection of immunogenic blood samples: One (1) sampling is to be performed within 0.5h before dosing of SHR-1701 on C1D1, C2D1, C4D1, C7D1, and on D1 of cycles beyond C7 at an interval of 6 cycles, on the subject's end of treatment day, and after 30 days (±7 days) of the last dose. At each time point, 5 mL of venous blood will be collected into a serum separator tube for ADA and Nab detection.

[21] Collection of tumor tissue samples: Subjects should provide fresh specimens (preferred) or archived formalin-fixed, paraffin-embedded tumor tissue blocks or 5-10 unstained tumor sections (at least 5 sections of tissue sample, at least 10 aspiration samples) of 3~5 μm in thickness. For the harvesting/collection and disposal procedures of tumor specimens, refer to Laboratory Manual. Tissue samples need to be sent to central laboratory for biomarkers studies. For a few subjects who are unable to provide adequate tissue samples, it is up to the investigator and the sponsor to determine the eligibility of subject patients for the study by discussion. This should be done within 28 days prior to the first dose.

[22] Adverse events: AEs should be collected from the signing of ICF to the end of the safety follow-up period; for the specific sample collection and subject follow-up principles, refer to 7.3.1 Follow-up of AEs/SAEs/SIEs. AEs should be followed up until resolution and the regression of relevant laboratory investigation results to baseline level, the degradation to Grade ≤1, the attainment of stable state, or the availability of a reasonable explanation (e.g., lost to follow-up, death); or final determination of that they are not related to the investigational drug/study process at the end of the safety follow-up period; if possible, best outcome and definite determination of relatedness of AEs to the investigational drug should be achieved for the subjects.

[23] Concomitant medications/concomitant therapies: Concomitant medications/concomitant therapies should be recorded from 30 days prior to the first dose of study medication to 30 days after the last study treatment (concomitant medications/concomitant therapies for the treatment of investigation drug related AEs/SAEs should be collected beyond 30 days after the last study treatment).

[24] End of treatment (EOT) visit: EOT visit has to be done at the decision of treatment withdrawal and/or study withdrawal ±3days. If imaging examination for efficacy assessment has been completed within 4 weeks, and safety assessment related examinations have been completed within 7 days prior to the discontinuation of study treatment, then none of such examinations has to be repeated at this stage.

[25] Safety follow-up: Safety follow-up starts from the last study treatment and ends 90 days after the last study treatment; during this period, the subjects are followed up

once every 30 days (±7 days). Safety Follow-up Visit 1 (30 days±7 days after the last dose) should be done at the study site for completing protocol specified evaluations; Safety Follow-up Visit 2 (60 days±7 days after the last dose) and Visit 3 (90 days±7 days) can be done by telephone or other valid method to collect survival information, concomitant medications/concomitant therapies and record AE(s).

[26] Survival follow-up: Subjects will proceed to survival follow-up at the end of the safety follow-up period until their death, lost to follow-up, withdrawal of ICF, or termination of the study by the sponsor. During this period, 1 follow-up visit will be carried out every 1 month for collection of survival information and subsequent treatment information (if the subject has started a new antitumor therapy, the regimen and start/end date of the treatment should be recorded).

Abbreviations

Abbreviations	Full name in English	Full name in Chinese
12-Lead ECG	12-Lead electrocardiogram	12-导联心电图
ADA	Anti-drug antibody	抗药抗体
ADRs	Adverse drug reactions	药物不良反应
AE	Adverse event	不良事件
AKP	Alkaline phosphatase	碱性磷酸酶
ALT	Alanine aminotransferase	谷氨酸丙氨酸氨基转移酶
ANC	Absolute Neutrophil Count	中性粒细胞计数
ANOVA	Analysis of variance	方差分析
APTT	Activeated Partial Thromboplastin Time	活化部分凝血活酶时间
AST	Aspartate aminotransferase	谷氨酸天门冬氨酸氨基转移酶
BUN	Blood urea nitrogen	尿素氮
CTC	Circulating Tumor Cell	循环肿瘤细胞
Cl-	Blood chlorine	血氯
Cr	Creatinine	肌酐
CR	Complete Response	完全缓解
CRF	Case report form	病例报告表
CRO	Contract research organization	合同研究组织
CTLA-4	Cytotoxic T Lymphocyte Antigen 4	细胞毒性 T 淋巴细胞抗原 4
D	Day	天
DC	Dendritic Cell	树突状细胞
DCR	Disease Control Rate	疾病控制率
DoR	Duration of Response	缓解持续时间
EC	Ethics committee	伦理委员会
ECG	Electrocardiogram	心电图
ER	Estrogen Receptor	雌激素受体
ES	Evaluable Set	可评估分析集
FAS	Full Analysis Set	全分析集
FT3	free triiodothyronine	游离三碘甲状腺原氨酸
FT4	free thyroxine	游离甲状腺素
GCP	Good Clinical Practice	药物临床试验质量管理规范
Hb	Hemoglobin	血红蛋白
HR	Hazard Ratio	风险比
HUVEC	Human umbilical vein endothelial cells	人血管内皮细胞
IB	Investigator’s brochure	研究者手册
IC_{50}	Half maximal inhibitory concentration	50%抑制浓度
irAE	Immune-related Adverse Event	免疫相关不良事件
IU	International unit	国际单位
K^+	Serum potassium	血钾
LDH	Lactate dehydrogenase	乳酸脱氢酶
MTD	Maximum tolerated dose	最大耐受剂量

Abbreviations	Full name in English	Full name in Chinese
Na+	Plasma sodium	血钠
NEUT	Neutrophil	中性粒细胞
HBV	Hepatitis B Virus	乙型肝炎病毒
HCV	Hepatitis C Virus	丙型肝炎病毒
HIV	Human Immunodeficiency Virus	人类免疫缺陷病毒
iRECIST	modified RECIST 1.1 for immune-based therapeutics	免疫治疗疗效评价标准
NOAEL	No Observed Adverse Effect Level	无可见有害作用水平
NYHA	New York Heart Association	纽约心脏病学会
ORR	Objective Response Rate	客观缓解率
OS	Overall Survival	总生存期
PD	Progressive Disease	疾病进展
PD-1	Programmed Death-1	程序性死亡分子 1
PD-L1	Programmed death-ligand 1	程序性死亡受体-配体 1
PDGFR	Platelet-derived growth factor receptors	血小板衍生生长因子受体
PFS	Progression Free Survival	无进展生存期
PPS	Per-Protocol Set	符合方案集
PR	Partial Response	部分缓解
PLT	Blood platelet	血小板
RBC	Red blood cell count	红细胞计数
RECIST	Response Evaluation Criteria In Solid Tumors	实体瘤疗效评价标准
SAE	Serious adverse event	严重不良事件
SAP	Statistical analysis plan	统计分析计划
SD	Stable Disease	疾病稳定
SS	Safety Set	安全性分析集
sUA	Serum uric acid	血尿酸
T-BIL	Total bilirubin	总胆红素
TEAE	Treatment Emergent Adverse Event	治疗期间出现的不良事件
TMB	Tumor Mutation Burden	肿瘤突变负荷
TTP	Time to Progress	疾病进展时间
UA	Uric acid	尿酸
URBC	Urine red blood cell	尿红细胞
VEGF	Vascular endothelial growth factor	血管内皮生长因子
WBC	White blood cell count	白细胞计数

1. Introduction: Background and Scientific Rationale of the Study

1.1. Study Background

1.1.1. Epidemiology and Clinical Treatment of PC

Pancreatic cancer (PC) is a malignant tumor of the digestive system in clinical, the incidence of which has been on the rise worldwide. Worldwide, the number of new diagnosed PC patients in 2017 was 448,000, 1.1 times the number in 1990; the number of deaths from PC was 441,000, up 30% from that in 1990 (1); According to the statistics from American Cancer Society (ACS), PC is the 4th leading cause of cancer death in the United States. It was estimated that about 23,800 males and 21,950 females died from PC in 2019(2). According to the malignant tumor statistics released by National Cancer Center(3), 48,000 Chinese men and 36,000 Chinese women died from PC in 2015. In China, the death toll of PC increased by 9% in the last 10 years; as a result of the change in life style and eating habit and the acceleration of population aging, the death toll of PC is increasing drastically (4).

PC generally develops occultly; its early manifestations include epigastric distress, lumbodorsal pain, dyspepsia, diarrhea, and other atypical symptoms; the prognosis of PC patients is poor since the disease is locally advanced or metastatic in 80% of patients at times of diagnosis and unresectable. The 5-year survival rate of PC patients is less than 8%(5) globally and only 2-4% (6-7) in China. It is thus evident more effective therapies are clinically in urgent need.

Systemic chemotherapy is the primary therapy for unresectable locally advanced or metastatic PC. However, its development has been very slow in recent 30 years. The earliest chemotherapy agent for advanced/metastatic PC is 5-fluorouracil (5-Fu). Later, gemcitabine became the first-line standard of care for advanced/metastatic PC due to its more superior control of tumor-related symptoms and more effectively prolongation of patient survival than 5-Fu (ORR: 5.4% vs. 0%; OS rates at 12-month: 18% vs. 2%; Median survival: 5.65 months vs. 4.41 months)(8). Afterwards, multiple phase III clinical studies of gemcitabine monotherapy vs. gemcitabine combination therapies with other cytotoxic drugs or targeted drugs in advanced/metastatic PC patients failed to produce survival benefits.

Results from a study of gemcitabine combination therapy with erlotinib: The combination therapy produced slightly longer survival of patients than gemcitabine monotherapy did (Median survival: 6.24months vs. 5.91 months; OS rates at 12-month: 23% vs. 17%;); however, no difference in ORR was observed between the two groups of patients (9). FOLFIRINOX (5-fluorouracil+calcium folinate+irinotecan+oxaliplatin) currently is the first-line chemotherapy regimen recommended in NCCN guideline. Primary rationale: In a phase III clinical study (10), FOLFIRINOX produced significantly longer survival and higher response rate than gemcitabine monotherapy in advanced/metastatic PC patients (ORR: 31.6% vs. 9.4%; Median PFS: 6.4 months vs. 3.3 months; Median survival: 11.1 months vs. 6.8 months). However, FOLFIRINOX is a 4-agent based chemotherapy featuring considerable toxic and side effects, its application in Chinese patients is limited only to patients with good ECOG PS score (0-1). The combination therapy of gemcitabine with albumin-bound paclitaxel (AG regimen) is another first-line standard chemotherapy regimen recommended in NCCN guidelines. The data from an international phase III clinical study (11) showed the combination treatment regimen produced better efficacy results than gemcitabine monotherapy (ORR: 23% vs. 7%; Median PFS: 5.5 months vs. 3.7 months; Median survival: 8.5 months vs. 6.7 months; OS rates at 12-month: 35% vs. 22%;); compared with FOLFIRINOX, this regimen has better medication safety and is tolerated better by patients. Its efficacy and safety in Chinese patients had been verified (12), therefore, this regimen has been extensively applied in clinical.

In Study POLO, olaparib, a PARP inhibitor, was used for maintenance treatment of metastatic

PC patients who had germline BRCA (gBRCA) mutations and showed no disease progression after treatment with first-line platinum-based chemotherapy. In the study, olaparib produced longer PFS than placebo control (mPFS: 7.4 months vs. 3.8 months), and lowered the risk of disease progression by 47%. After 1-year follow-up, 33.7% of the patients in Olaparib Treatment Group achieved PFS; by contrast, only 14.5% of the patients in the Placebo Group achieved that; after two years of follow-up, PFS was 22.1% in Olaparib Group patients and only 9.6% in Placebo Group patients. The drug retarded the disease progress in patients without adverse impact on their quality of life(13). It is recommended NCCN Guidelines for Pancreatic Adenocarcinoma (3rd Edition, 2019) that all patients receive gene sequencing to determine their BRCA1/2 mutational status. For patients with relevant mutations, olaparib maintenance therapy may be considered. However, only 4-7% of PC patients have BRCA1/2 mutations(14); therefore, the number of patients that can benefit from this therapy is very limited.

1.1.2. Exploratory study of immune checkpoint inhibitors in advanced/metastatic PC

Immunotherapeutic drugs, represented by immune checkpoint inhibitors, are developed and applied rapidly, achieving good results with controllable overall safety. However, immunotherapeutic drugs failed to achieve desired results in early exploratory studies for the treatment of PC(15). In current CCN guidelines, pembrolizumab is recommended as category 2 regimen for second-line treatment of patients with MSI-H or dMMR advanced/metastatic PC. However, generally PC has very low tumor mutation load and its incidence of dMMR is less than 1%. In light of this, the combination of immune checkpoint inhibitor with chemotherapy has opened up a new avenue for exploration. The theoretical basis of this combination therapy lies in that cytotoxic chemotherapy drugs such as gemcitabine and paclitaxel have immunomodulatory action(16); in addition to killing tumor cells, chemotherapy drugs can increase the exposure and presentation of tumor antigen, improve the activity of immune killer cells and T cells, thereby achieving synergism. Pembrolizumab (17) and Nivolumab (18) have been exploratorily been used in combination with AG regimen. Preliminary study results （Table 2) initially demonstrated the safety and feasibility of this co-medication; however, this combination therapy has to be further demonstrated in future studies.

Table 2 Preliminary study results of the combination therapy of Pembrolizumab and Nivolumab with AG regimen

Chemotherapy regimen (sample size)	mOS (95% CI)	mPFS (95% CI)	ORR (95% CI)
Pembro+AG[2] n=12	15.0 months (6.8-22.6)	9.1 months (4.9-15.3)	25%
Nivo+AG[3] N=50	9.9 months	5.5 months	18%

1.1.3. Exploration of the roles of PD-L1/TGFβ in advanced tumors

In microenvironment of tumor, PD-L1/PD-1 pathway and TGF-β signaling pathway play very important roles in the immune escape of tumors. PD-1/PD-L1 are a pair of negative immune costimulatory signaling molecules; PD-L1, also known as Cluster of Differentiation 274 (CD274) or B7 homolog 1 (B7-H1), is a member of the B7 family; the ligand of PD-1 is a type I 40 kDa transmembrane protein that can negatively regulate immune response. PD-L1 is mainly expressed in T-cells, B-cells, macrophages and dendritic cells (DCs); when PD-L1 is activated, its expression in cells can be upregulated. The PD-L1 expressed on tumor cell surface inhibits the functioning of lymphocytes by binding to the PD-1 molecules on the surface of TILs, and this inhibition is an important cause of immune escape of tumors. In light of this, studies of the interaction between PD-1 and PD-L1 hopefully will provide experiment basis for exploration of

immune escape and target therapy of tumors.

TGF-β is a disome (molecular weight: 12.5 kDa) formed by the binding of two structurally identical or similar subunits through disulfide bond. TGF-β signaling pathway has two-way regulation of the genesis and development of tumors: it inhibits the growth of early tumors but promotes the growth of advanced tumors. At early stage of tumorigenesis, the TGF-β signaling pathway is weakened due to the negative feedback caused by the high expression of inhibitory Smad7 and other two negative regulatory factors (SKI-like factor and TGF-β inducing factor); the latter two are co-inhibitory factors of Smad2/3 dependent transcription complex; at advanced stage of tumorigenesis, the highly active state of that pathway plays important roles in tumor infiltration and metastasis by triggering the changes of tumor cell skeleton, cell adhesion and matrix remodeling and migration related gene expressions and participating in the regulation of the epithelial-mesenchymal transition (EMT) of tumors. TGF-βRII can phosphorylate the downstream matrix Smad protein by directly binding to free ligands TGF-β1 or TGF-β3, initiating the entire signaling pathway, connecting the bound ligand of TGF-βRII, and phosphorylating type I receptor TGF-βRI. Therefore, TGF-βRII as the key link for TGF-β to play its regulatory action is essential to the above-mentioned signaling pathway.

TGF-β can upregulate the PD-L1 on antigen presenting cells (APCs), which release soluble PD-L1 into the microenvironment of tumor and, through the interaction between PD-L1 and its receptor, directly inhibit cytotoxic T lymphocytes, or induce the cytotoxic T lymphocytes to release soluble inhibitory molecules. As a result, targeted neutralizing the TGF-β in tumor micro-environment on the basis of the suppression of PD-1/PD-L1 pathway can restore the activity of T cells, enhance immune response, and more effectively improve the suppression of tumorigenesis and development. This has become a new regimen for the treatment of tumors.

Bifunctional fusion proteins are fusion proteins that have two functional structure domains. They have become a hotspot in the study field of bioengineering therapeutics and show promising prospects for the immunotherapy of tumors and the treatment of autoimmune diseases for their specificity and bifunctional nature. MSB0011359C (M-7824), a PD-L1 and TGF-β bifunctional fusion protein co-developed by EMD Serono Inc and Merck KGaA, has displayed considerable efficacy in the treatment of advanced solid tumors.

In an open-label, dose-escalation phase I clinical study (NCT02517398) in patients with advanced solid tumors, the safety and effectiveness of M7824 had been preliminarily investigated(19) . A total of 19 subjects took part in the study from September 2015 to March 2017. The subjects received treatment with M7824 at 0.3 ~ 20 mg/kg: the drug was administered to 3 subjects at each of the dose levels of 0.3 mg/kg, 1 mg/kg, 3 mg/kg and 10 mg/kg, and to 7 subjects at the dose level of 20 mg/kg. The subjects' median treatment duration was 11.9 weeks (range: 4.0 ~ 41.9 weeks). 47% of the subjects reported drug-related AEs of Grade ≥ 1. For (4) subjects reported 1 occurrence of each of the following drug-related AEs of Grade ≥ 3: skin infection secondary to localized bullous pemphigoid (3 mg/kg), asymptomatic lipase increased (20 mg/kg), colitis with anemia (20 mg/kg) and gastroparesis with hypopotassemia (10 mg/kg). MLT had not been reached in the study at dose level up to 20 mg/kg. No subject died from AE during the study.

Efficacy analysis results: the drug showed antitumor effect at all study dose levels. Five (5) PC patients were included, 1 achieved PR, 3 had SD, 1 had PD; presently, a phase Ib/II study is underway in which the drug is used in combination with gemcitabine for second-line therapy of PC;

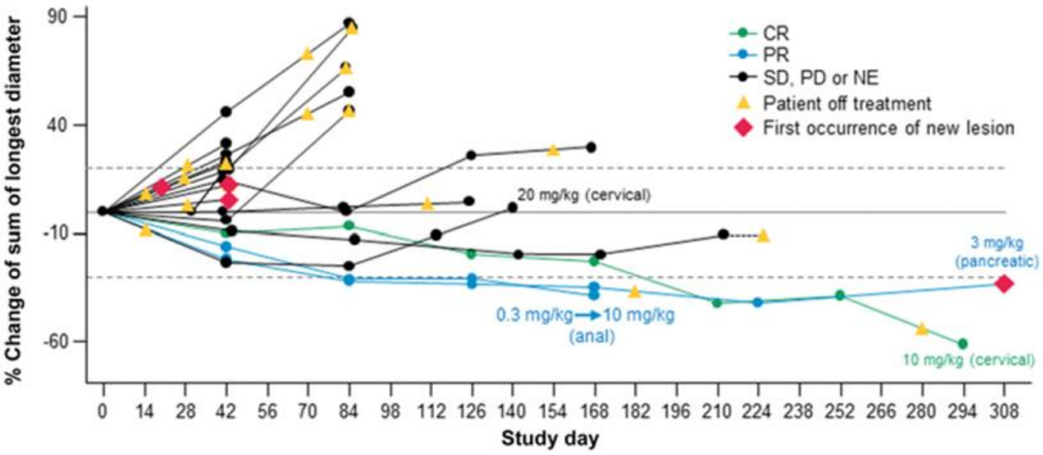

Figure 1 Evaluation of response to M7824 in a phase I clinical study

SHR-1701 is an anti-PD-L1/TGF-βRII bifunctional fusion protein developed by Henrui. Its molecule consists of two parts, an anti-PD-L1 mAb and a truncated TGF-βRII extracellular structure domain. To be specific, the protein is formed by connecting the C-terminal amino acid of the heavy chain of the anti-PD-L1 antibody to the N-terminal amino acid of the TGF-βRII extracellular structure domain by a junction protein fragment (G4S) 4G. SHR-1701 can block the PD-1/PD-L1 pathway, and neutralize TGFβ in tumor microenvironment. Compared with the suppression of any single pathway, the cosuppression of PD-1 and TGF-β negative signal can bring about more effective antitumor immune response, thereby improving antitumor efficacy.

1.2. Information of the drug SHR-1701

[Generic Name]: SHR-1701 Injection

[English Name]: SHR-1701 Injection

1.2.1. Type of the drug's pharmacologic action and the mechanism of action

Programmed death 1 ligand 1 (PD-L1), also known as B7 homologue 1 (B7-H1), is a type I transmembrane protein in B7 family. Generally expressed on antigen presenting cells (APCs), it mainly binds to programmed cell death 1 (PD-1; CD279) for releasing inhibitory signal to limit the activation and proliferation of T cells, thereby playing an important role in downregulating autoimmunity. In recent years, it is found that multiple tumor cells on their surface have high expression of PD-L1, which when binding to PD-1 on the surface of TILs inhibits the functioning of lymphocytes and mediate the immune escape of tumors. PD-1/PD-L1 pathway specific inhibitors can relieve PD-L1 mediated immunosuppression and improve the functioning of killer T cells to mobilize the immune system to eliminate tumor cells in the body.

Transforming growth factorβ (TGF-β) is a cytokine that has a variety of bioactivities. It takes part in the regulating of many life activities, including the proliferation, differentiation, development and apoptosis of cells. TGF-β has two-way regulating functions on tumor cells at different stages. TGF-β can promote cycle arrest and trigger apoptosis of tumor cells at early stage of tumorigenesis and promote the migration and infiltration of tumor cells at late stage of tumorigenesis, thereby promoting the development and metastasis of cancers. There are 3 subtypes of TGF-β, i.e., TGF-β1, TGF-β2 and TGF-β3; all of which can bind to receptors on cell surface for playing their regulatory actions. TGF-βRII can phosphorylate the downstream matrix Smad protein by directly binding to free ligands TGF-β1 or TGF-β3, initiating the entire signaling pathway, connecting the bound ligand of TGF-βRII, and phosphorylating type I receptor TGF-βRI. Therefore, TGF-βRII as the key link for TGF-β to play its regulatory action is essential to the above-mentioned signaling pathway.

TGF-β can upregulate the PD-L1 on antigen presenting cells (APCs), which release soluble PD-L1 into the microenvironment of tumor and, through the interaction between PD-L1 and its receptor, directly inhibit cytotoxic T lymphocytes, or induce the cytotoxic T lymphocytes to release soluble inhibitory molecules. As a result, targeted neutralizing the TGF-β in tumor micro-environment on the basis of the suppression of PD-1/PD-L1 pathway can restore the activity of T cells, enhance immune response, and more effectively improve the suppression of tumorigenesis and development. This has become a new option for the treatment of tumors.

1.2.2. Pharmacodynamic study

1.2.2.1. In vitro affinity assay

The affinity of SHR-1701 for PD-L1 and TGF-β was analyzed by SPR technique with Biacore system; also analyzed was the affinity of SHR-1701 for Fc receptor and C1q protein; throughout the assay process, SHR-1701 was compared with the control antibody M7824. Results: SHR-1701 showed potent affinity activity(10^{-10}M) for human PD-L1 protein and comparable and potent affinity activities for human and mouse TGF-β1 (10^{-12} ~ 10^{-13}M). The antibody showed certain selectivity for 3 subtypes of human TGF-β; it has high affinity (10^{-11} ~ 10^{-13} M) for TGF-β1 and TGF-β3 and relatively low affinity for TGF-β2(10^{-9}M). This suggests the antibody's selectivity is good and in agreement with the control antibody M7824's and the reported value in literature. In slightly acidic environment (pH≤6.5), SHR-1701 showed good affinity for FcRn of 4 animal species (rat, mouse, monkey, human), with KD values of 9.311×10^{-8}, 1.047×10^{-7}, 8.571×10^{-7} and 1.431×10^{-6} M, respectively; its affinity was comparable to the control antibody M7824's. Being an IgG4 subtype product, SHR-1701 has low affinity for FcγR of various subtypes and does not bind to C1q protein; these findings suggest it has no ADCC and CDC. For detailed results refer to Table 3, Table 4 and Table 5.

Table 3 Affinity of SHR-1701 and M7824 for PD-L1 protein and TGFβ

Investigational drug	Antigen	k_a ($M^{-1}s^{-1}$)	k_d (s^{-1})	K_D (M)
SHR-1701	Recombinant human PD-L1 protein	1.153E+6	1.510E-4	1.309E-10
	Recombinant human TGF-β1 protein	7.491E+7	2.549E-5	3.403E-13
	Recombinant monkey TGF-β1 protein	Non-binding		
	Recombinant mouse TGF-β1 protein	1.486E+8	0.001191	8.012E-12
	Recombinant human TGF-β2 protein	8.854E+5	0.004338	4.900E-9
	Recombinant human TGF-β3 protein	8.492E+7	0.002455	2.891E-11
M7824	Recombinant human PD-L1 protein	1.665E+5	1.011E-4	6.069E-10
	Recombinant human TGF-β1 protein	1.314E+8	0.001747	1.329E-11
	Recombinant monkey TGF-β1 protein	Non-binding		
	Recombinant mouse TGF-β1 protein	1.155E+12	27.20	2.354E-11
	Recombinant human TGF-β2 protein	4.242E+6	0.005787	1.364E-9
	Recombinant human TGF-β3 protein	6.524E+7	0.002826	4.332E-11

Table 4 Affinity of SHR-1701 and M7824 for FcRn in a variety of animal species

Investigational drug	FcRn protein	k_a ($M^{-1}s^{-1}$)	k_d (s^{-1})	K_D (M)
SHR-1701	Recombinant rat FcRn protein	2.755E+4	0.002565	9.311E-8
	Recombinant mouse FcRn protein	6.773E+4	0.007090	1.047E-7
	Recombinant monkey FcRn protein	2.166E+5	0.1856	8.571E-7
	Recombinant human FcRn protein	1.219E+5	0.1745	1.431E-6
M7824	Recombinant rat FcRn protein	8.868E+4	0.002299	2.592E-8
	Recombinant mouse FcRn protein	9.763E+4	0.003011	3.084E-8
	Recombinant monkey FcRn protein	2.491E+5	0.08881	3.566E-7
	Recombinant human FcRn protein	2.410E+5	0.1156	4.796E-7

Table 5 Affinity of SHR-1701 and M7824 for human FcγR or C1q

Investigational drug	FcR/C1q protein	k_a ($M^{-1}s^{-1}$)	k_d (s^{-1})	K_D (M)
SHR-1701	Recombinant human FcγRI (Human CD64)	2.204E+5	0.001091	4.951E-9
	Recombinant human FcγRIIB (Human CD32b)	N/A	N/A	9.641E-6
	Recombinant human FcγRIIIA (Human CD16a)	1.648E+4	1.016	6.164E-5
	Recombinant human C1q	Non-binding		
M7824	Recombinant human FcγRI (Human CD64)	4.119E+5	1.804E-4	4.379E-10
	Recombinant human FcγRIIB (Human CD32b)	N/A	N/A	6.688E-7
	Recombinant human FcγRIIIA (Human CD16a)	9.462E+4	0.06238	6.592E-7
	Recombinant human C1q	5.022E+5	0.1699	3.383E-7

1.2.2.2. Binding assay with PD-L1 protein of a variety of animal species and humanized B7 family protein

The binding of SHR-1701 with PD-L1 of different animal species (human, mouse, monkey) or with other human B7 family proteins (B7-1/CD80, B7-H3/CD276, B7-DC/PD-L2/CD273) was assayed by ELISA. The corresponding antigens were coated in 96-well plate, sealed and left at 4 °C overnight; the plate was washed 3 times on the next day and blocked with blocking buffer (containing 0.5% BSA) for 2h, washed another 3 times, then added the test antibody for incubation at 37 °C for 2h; at the end of the incubation, the plate was washed 3 times and added TMB solution for chromgenesis, then incubated at room temperature away from light for 10min, then added stop solution and the OD values of wells were read at 450nm for calculation of EC_{50}.

SHR-1701 could bind with human and monkey PD-L1 protein, with EC_{50} values in the ranges of 0.28 ± 0.02 nM and 0.04 ± 0.01 nM, respectively; its binding capacity was comparable to the control antibody SHR-1316(humanized anti-PD-L1mAb)'s. SHR-1701 showed no notable binding with murine PD-L1 and other human B7 family proteins (B7-1/CD80, B7-H3/CD276, B7-DC/PD-L2/CD273), suggesting that it had good target selectivity. For detailed results, refer to Table 6 and Figure 2.

Table 6 Binding capacity of SHR-1701 to PD-L1 of a variety of animal species and other human B7 family proteins

Protein	EC_{50} (nM)	
	**SHR-1701**	**SHR-1316**
PD-L1 (Human)	0.28 ± 0.02	1.48 ± 0.25
PD-L1 (Monkey)	0.04 ± 0.01	0.11 ± 0.78
PD-L1 (Mouse)	N/A	N/D
PD-L2 (human)	N/A	N/D
B7-1 (human)	N/A	N/D
B7-H3 (human)	N/A	N/D

(Mean±SD, n=3); N/A, No binding; N/D, Not detected.

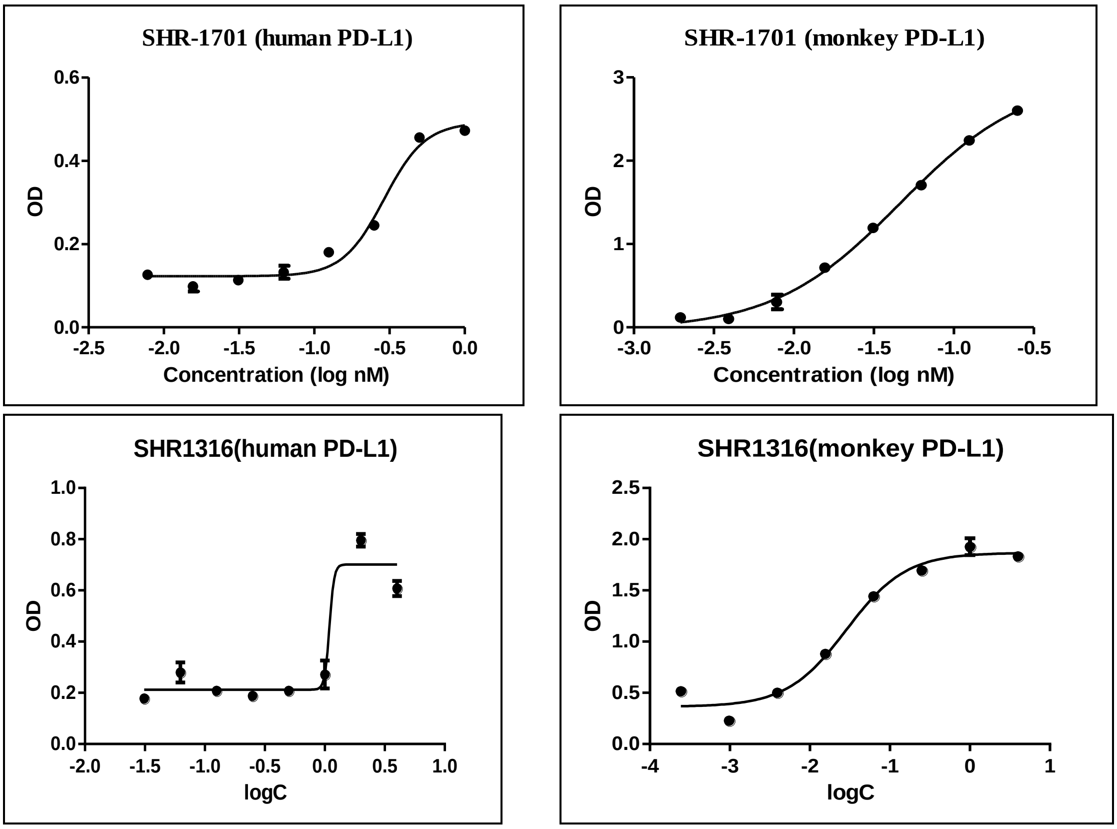

Figure 2 Binding capacity of SHR-1701 with PD-L1 of a variety of animal species and human B7 family protein

1.2.2.3. Binding assay of SHR-1701 at cell level

SHR-1701 or the control antibody were labeled with FITC and diluted to a variety of concentration gradients for incubation with MC38/H-11 mouse colon carcinoma cells (the murine PD-L1 was knocked out and human PD-L1 was transfected), washed, and fluorescence intensity was determined with flow cytometer and EC_{50} was calculated. Results: SHR-1701 can bind to mouse colon carcinoma cell MC38/H-11 (in which murine PD-L1 is knocked out and human PD-L1 is transfected), with EC_{50} of 0.69 ± 0.619 nM; by contrast, the EC_{50} of control antibodies SHR-1316 and M7824 was 0.90 ± 0.96 nM and 1.78 ± 1.53 nM, respectively. Results: The binding activity of SHR-1701 at cell level was comparable to those of SHR-1316 and M7824; for detailed results, refer to Figure 3.

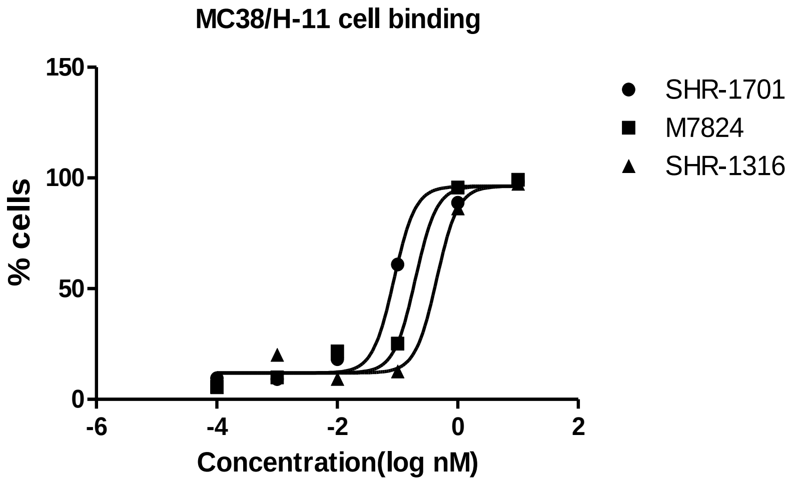

Figure 3 SHR-1701's binding capacity with MC38/H-11 cell

1.2.2.4. PD-L1/PD-1 binding blockage test of SHR-1701

First, PD-L1-CHO/K1 cells were added into 96-well plate, incubated at 37 °C for 18h; then, SHR-1701 antibody and the control antibody were diluted to a series of concentrations and added into the corresponding wells of the 96-well plate; Jurkat-PD1 cells were added, and incubated at 37 °C for 6 h; finally, to each the wells, prepared Bio-GbTM reagent was added, and the plate was incubated at room temperature for 10min; the spontaneous luminescence value was determined with microplate reader. Results: SHR-1701 could specifically block the interaction between PD-L1 and PD-1 of the surface of two kinds of cells, with EC_{50} values of 1.67 ± 0.30 nM; SHR-1316's and M7824's EC_{50} values were 1.07 ± 0.71 nM and 1.87 ± 0.66 nM, respectively; SHR-1701's blocking capacity was comparable to SHR-1316's and M7824's; for detailed results, refer to Figure 4.

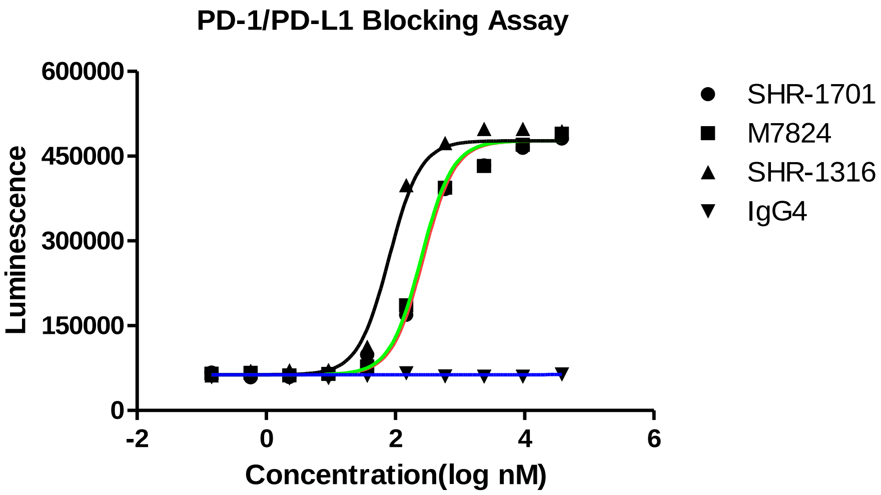

Figure 4 SHR-1701's blocking capability of PD-L1/PD-1 binding

1.2.2.5. SHR-1701's antitumor efficacy on MC-38/H-11 xenograft in mice

8 week-old female C57BL/6 mice were inoculated 3.5×10^5 MC38/H-11 cells to their left axilla. The tumors were allowed to grow until the mean tumor volume in each group reached about 50 mm^3; then the animals were randomized into 6 groups and administered SHR-1701 (3, 10, 30 mg/kg, t.i.w., ×8) by intracaudate injection; animals in the vehicle control group were

administered IgG4 or SHR-1316 PBS of the same volume by intracaudate injection; animals in IgG4 and SHR-1316 groups were administered 30 mg/kg IgG4 or SHR-1316, respectively. The animals were weighed and their tumor volume was measured twice a week; their relative tumor volume (RTV), relative tumor growth rate (T/C), and tumor growth inhibition ratio (TGI) were calculated and subjected to statistical analysis by One-Way ANOVA (SPSS 19.0).

On postdose D19, RTVs (in relation to Vehicle Control Group (PBS)) for IgG4 30 mg/kg Group, SHR-1701: 3, 10 and 30 mg/kg Groups, SHR-1316 30 mg/kg Group were 41.31 ± 5.98, 26.57 ± 4.92, 18.88 ± 2.12 (P<0.01), 17.02 ± 2.55 (P<0.01) and 26.80 ± 3.83, respectively; T/C values were 111.47%, 71.70%, 50.96%, 45.92%, 72.32%, respectively; TGI ratios were -11.47%, 28.30%, 49.04%, 54.08%, 27.68%, respectively. The tumor bearing mice could tolerate the above drugs well. For detailed results, refer to Table 7 SHR-1701's efficacy in MC38/H-11 mouse colon carcinoma cell xenograft model and Figure 5, Figure 6and Figure 7.

Table 7 SHR-1701's efficacy in MC38/H-11 mouse colon carcinoma cell xenograft model

Group	Dose level (mg/kg)	Tumor volume (mm^3)		RTV	T/C (%)	TGI (%)
		D1	D19			
PBS	--	46 ± 3	1721 ± 324	37.06 ± 5.44	—	—
IgG4	30	47 ± 3	1842 ± 196	41.31 ± 5.98	111.47	-11.47
SHR-1701	3	45 ± 3	1202 ± 214	26.57 ± 4.92	71.70	28.30
SHR-1701	10	46 ± 3	913 ± 138*	18.88 ± 2.12**	50.96	49.04
SHR-1701	30	46 ± 3	762 ± 114**#	17.02 ± 2.55**#	45.92	54.08
SHR-1316	30	48 ± 2	1252 ± 177	26.80 ± 3.83	72.32	27.68

(Mean±SEM, n=10)

One-Way ANOVA, vs. PBS, *:P<0.05**:P<0.01, ***:P<0.001; vs. SHR-1316 30 mg/kg, #:P<0.05.

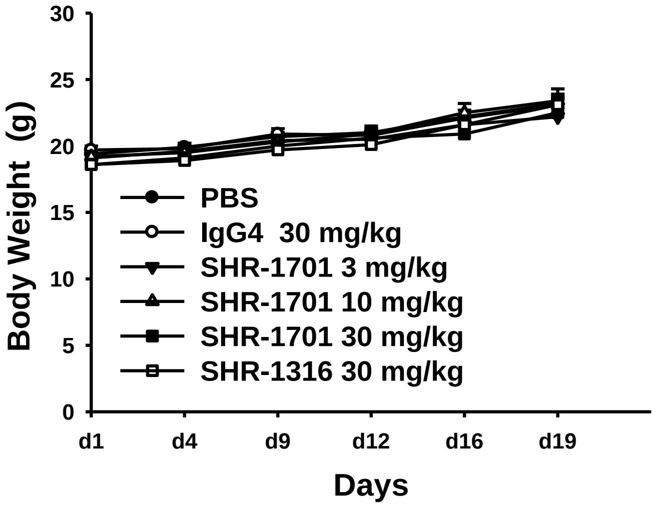

Figure 5 SHR-1701's effect on body weight of MC38/H-11 mouse colon carcinoma cell tumor-bearing mice

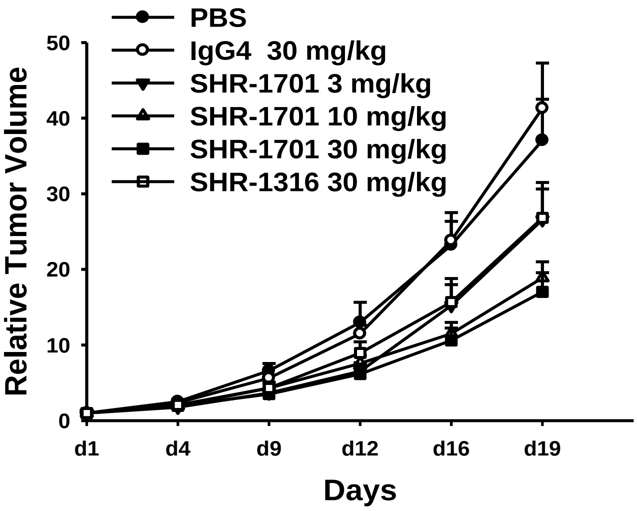

Figure 6 SHR-1701 's efficacy in MC38/H-11 mouse colon carcinoma cell xenograft model (by RTV)

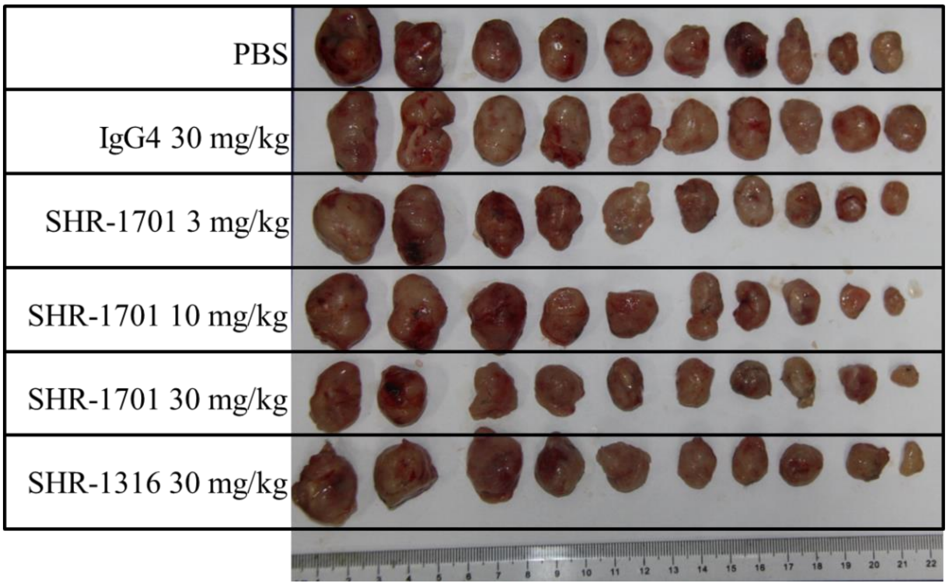

Figure 7 SHR-1701's efficacy in MC38/H-11 mouse colon carcinoma cell xenograft model(tumor photos)

For more detailed pre-clinical PD data, refer to Investigator's Brochure (IB).

1.2.3. Safety pharmacology study

A single intravenous dose of 30, 100, 300 mg/kg SHR-1701 showed no notable effect on the CNS of SD rats; a single intravenous dose of 100 mg/kg SHR-1701 showed no notable effect on the cardiovascular, respiratory functions and body temperature of conscious, unrestrained cynomolgus monkeys. Tissue cross-reactivity assay was conducted with fresh frozen normal human tissues, cynomolgus monkey tissues and rat tissues. Results: SHR-1701 had tissue cross-reactivity with human placenta, tonsil, cynomolgus monkey tonsil, mammary gland, parotid salivary gland, striated muscle and rat choroid plexus, parotid salivary gland and no tissue cross-reactivity with other tissues of the three species.

1.2.4. Toxicological studies

A single intravenous dose of 30, 100, 300 mg/kg SHR-1701 showed no notable effect on the CNS of SD rats; a single intravenous dose of 100 mg/kg SHR-1701 showed no notable effect on

the cardiovascular, respiratory functions and body temperature of conscious, unrestrained cynomolgus monkeys. Tissue cross-reactivity assay was conducted with fresh frozen normal human tissues, cynomolgus monkey tissues and rat tissues. Results: SHR-1701 had tissue cross-reactivity with human placenta, tonsil, cynomolgus monkey tonsil, mammary gland, parotid salivary gland, striated muscle and rat choroid plexus, parotid salivary gland and no tissue cross-reactivity with other tissues of the three species. No abnormal reaction was observed in SD rats administered a single intravenous dose of 30 mg/kg or 750 mg/kg SHR-1701; the antibody's maximum tolerated dose (MTD) was ≥ 750 mg/kg; in cynomolgus monkeys that had been administered a single intravenous dose of 150 mg/kg or 479 mg/kg SHR-1701, only 1 animal in high-dose group showed transient WBC, Neut, Retic increases, which were considered to be related to the test substance. The antibody's MTD was ≥ 479 mg/kg in cynomolgus monkeys. Repeat-dose toxicity studies were conducted in rats and cynomolgus monkeys, respectively, in order to evaluate the toxicity manifestation of SHR-1701 after the administration of multiple doses. A total of 5 doses of SHR-1701 was administered intravenously to SD rats at 15, 50, 150 mg/kg/dose, q.w., in 4 consecutive weeks. In all dose groups, pharmacologic action related TGF-β1 decrease was observed in the animals; also observed in the animals was thymus weight decreased; histological examination revealed slight or mild cortical lymphocytes decreased. SHR-1701 had certain immunogenicity in rats; the proportion of ADA positive animals in low-, mid-, high-dose groups was 25% (2/8), 12.5% (1/8) and 12.5% (1/8), respectively. Under the condition of the study, SHR-1701 had an STD_{10} > 150 mg/kg, at which dose level, the mean C_{max} and $AUC_{(0-144h)}$ of the drug on D29 were 6465.397 μg/mL and 344536.8 μg×hr/mL, respectively, in male animals, and 6465.397 μg/mL and 344536.8 μg×hr/mL, respectively, in female animals. A total of 5 doses of SHR-1701 was administered intravenously to cynomolgus monkeys at 30, 75, 150 mg/kg/dose, q.w., in 4 consecutive weeks. In all dose groups, pharmacologic action related transient HGB slightly decreased was observed in the animals; save for the afore-mentioned, no notable abnormal change was observed in the cynomolgus monkeys with regard to general status, body weight, food consumption, body temperature, II-lead ECG, blood pressure, respiratory rate, ophthalmic examination, hematology, blood chemistry panel, routine urinalysis, bone marrow examination, complement, circulating immunocomplex, lymphocyte subset, cytokines, organ weight and coefficicent, gross anatomical observation and histopathological examination. SHR-1701 showed potent immunogenicity in cynomolgus monkeys. The proportions of ADA positive animals in low-, mid-, high-dose groups were 100% (10/10), 80% (8/10) and 100% (10/10), respectively. Under the condition of the study, the drug's NOAEL was 150 mg/kg, at which level, the drug's mean C_{max} and $AUC_{(0-144h)}$ on D29 were 6327.359 μg/mL and 498550.820 μg×hr/mL, in male animals, and 5502.563 μg/mL and 416606.480 μg×hr/mL, in female animals. In vitro hemolysis and local irritation tests were conducted concomitantly with the long-term toxicity study; the results were all negative, suggesting that SHR-1701 is neither hemolytic nor irritating to the injection site.

1.2.5. Immunogenicity/immunotoxicity studies

1.2.5.1. Four-week repeat-dose immunogenicity and immunotoxicity study in rats

This experiment was conducted concomitantly with the 4-week repeat-dose toxicity study in rats; the animals were observed for any immunogenicity or immunotoxicity potentially caused by the drug. SD rats were intravenously administered 5 repeat doses of SHR-1701 at 15, 50 and 150 mg/kg,q.w., in 4 consecutive weeks; during the study, the animals were subjected to blood cell counting, blood chemistry panel, lymphocyte subset (CD3+, CD4+, CD8+ and CD4+/CD8), cytokines (IL-2, IL-4, IL-6, TNF-α, IFN-γ and TGF-β1), anti-drug antibody, organ weight, gross anatomy and histopathology examinations. In all groups, serum TGF-β1 decreased, thymus weight decreased were observed in the animals after dosing; histological examination revealed slight or mild cortical lymphocytes decreased. The afore-mentioned changes were considered to

be related to the test substance's pharmacologic actions. Save for the above-mentioned, no abnormality was observed in any group of animals with regard to hematology, blood chemistry panel, lymphocytes subtype, cytokines, organ weight, gross anatomy and histopathology. SHR-1701 had certain immunogenicity in rats; the proportion of ADA positive animals in low-, mid-, high-dose groups was 25% (2/8), 12.5% (1/8) and 12.5% (1/8), respectively.

1.2.5.2. Four-week repeat-dose immunogenicity and immunotoxicity study in cynomolgus monkeys

This experiment was conducted concomitantly with the 4-week repeat-dose toxicity study in cynomolgus monkeys; the animals were observed for any immunogenicity or immunotoxicity potentially caused by the drug. Cynomolgus monkeys were intravenously administered 5 repeat doses of SHR-1701 at 30, 75 and 150 mg/kg, q.w., in 4 consecutive weeks. No marked abnormality was observed in any of the animal groups during the clinical pathology (blood cell counting, blood chemistry indicators), T lymphocyte subset, cytokines (IL-2, IL-4, IL-5, IL-6, TNF-α, IFN-γ, TGF-β1), immunoglobulins (IgG, IgM, IgA), serum complements (C3, C4), anti-drug antibody and neutralizing antibody, organ weight, gross anatomy and histopathology examinations. SHR-1701 showed potent immunogenicity in cynomolgus monkeys. The proportions of ADA positive animals in low-, mid-, high-dose groups were 100% (10/10), 80% (8/10) and 100% (10/10), respectively. Anti-drug (PD-L1 part) neutralizing antibodies were observed in 30 mg/kg group and 75 mg/kg group; anti-drug (TGFβRII part) neutralizing antibodies were observed in all dose groups.

1.2.6. Preclinical pharmacokinetic studies

In SD rats that had been administered a single intravenous dose of SHR-1701 at 3, 10 and 30 mg/kg, no marked gender variability was observed in any of the dose groups with regard to SHR-1701 exposure, which increased with dose; the drug's C_{max} and AUC increased linearly with dose. SHR-1701 had certain immunogenicity in rats; the proportion of ADA positive animals in low-, mid-, high-dose groups was 16.7% (1/6), 16.7% (1/6) and 66.7% (4/6), respectively. In cynomolgus monkeys that had been administered a single intravenous dose of SHR-1701 at 3, 10, 30 mg/kg, no significant gender variability was observed in any of the groups with regard to SHR-1701 exposure, which increased with dose; the drug's C_{max} and AUC increased linearly with dose. No significant accumulation of SHR-1701 was observed in any cynomolgus monkeys that had been administered repeat weekly doses of SHR-1701 at 10 mg/kg for 4 consecutive weeks. SHR-1701 showed potent immunogenicity in cynomolgus monkeys. Postdose ADA test results were positive in all groups of animals. In cynomolgus monkeys that had been administered a single intravenous dose of SHR-1701, it was observed that the occupancy level of receptors on T cell surface rapidly became saturated. In 3, 10 mg/kg dose groups, receptor occupancy rate showed a gradually decreasing trend over time. In tumor bearing mice that had been intravenously injected ^{125}I-labeled SHR-1701, radioactivity was high in tissues and organs that have profusive blood perfusion (e.g., heart, lungs, kidney, etc.); the high radioactivity in tumor tissue suggests that the drug distribution is tumor-targeted; the distribution of radioactivity was scarce in organs where blood perfusion is less profusive. SHR-1701 is mainly excreted via urine in the form of small-molecular degradation products. No unchanged drug was present in the urine. A few of the drug was excreted via feces. About 56.21% ± 7.37% and 29.43% ± 5.57% of the injected amount of radioactivity were excreted into urine and feces, respectively. For the in vivo PK parameters of SHR-1701 in cynomolgus monkeys, refer to Table 8 and Table 9.

Table 8 Summary of PK parameters of SHR-1701 administered to cynomolgus monkeys in a single intravenous dose

Parameter	Unit	3 mg/kg	10 mg/kg	30 mg/kg
AUC_{INF_obs}	h×μg/ml	4560±856	20700±3810	60800±14300
AUC_{last}	h×μg/ml	4190±541	20000±3530	59300±14200
$Cl_{\_obs}$	ml/h/kg	0.674±0.107	0.496±0.083	0.516±0.119
C_{max}	μg/ml	80.1±11.7	307±45.2	711±153
MRT_{last}	h	63.6±7.25	80.9±13.5	126±33.8
$T_{1/2z}$	h	53.4±20.2	48.2±18.2	81.3±28.4
$T_{max}^{\$}$	h	0.250 [0.25,0.25]	0.250 [0.25,0.25]	0.250 [0.25,0.25]
V_{ss_obs}	ml/kg	52.9±8.22	43.1±5.21	68.3±17.2
V_{z_obs}	ml/kg	50.4±15.1	33.4±11.9	58.5±16.0

(Mean±SD, n=6)
$: medium [min, max]

Table 9 Summary of PK parameters of SHR-1701 administered to cynomolgus monkeys in multiple intravenous doses

Parameter	Unit	First Dose	Last dose	Last/First
AUC_{0_168}	h×μg/ml	12600±2840	2720±2460	0.22
AUC_{INF_obs}	h×μg/ml	16000±4810	2740±2480	0.17
AUC_{last}	h×μg/ml	12600±2840	1980±2250	0.16
$Cl_{\_obs}$	ml/h/kg	0.673±0.206	9.72±9.29	14.44
C_{max}	μg/ml	241±24.9	152±50.4	
MRT_{last}	h	57.5±5.19	13.8±12.4	
$T_{1/2z}$	h	77.3±19.1	16.8±12.0	
$T_{max}^{\$}$	h	0.250 [0.25, 0.25]	0.250 [0.25, 0.25]	
V_{ss_obs}	ml/kg	65.8±10.5	99.0±56.8	
V_{z_obs}	ml/kg	71.3±12.7	124±67.0	

(Mean±SD, n=6)
$: medium [min, max]

1.2.7. Pre-clinical study of receptor occupancy

From cynomolgus monkeys administered a single intravenous dose of SHR-1701 at 3, 10, 30 mg/kg, heparin anticoagulated whole blood samples were collected before and after dosing at a variety of time points in duplicate (aliquot A and aliquot B); aliquot B was subjected to in vitro spiking to create saturated receptor occupancy, stained with Human TGF-beta RII Biotinylated antibody, APC Streptavidin and fluorescein-labeled anti-CD3 antibody, and then assayed with flow cytometer. Aliquots A and B were gated with FSC/SSC and CD3+, T lymphocytes were circled, and fluorescence intensity of antibodies bound to T cell surface was assayed for calculating receptor occupancy (RO).

In cynomolgus monkeys that had been intravenously administered SHR-1701 at 3, 10 and 30 mg/kg, it was observed that the RO level on T cell surface rapidly became saturated and RO showed a gradually decreasing trend over time; for detailed results, refer to Table 10 and Figure 8.

Table 10 Receptor occupancy on cynomolgus monkey T cells after a single intravenous dose of SHR-1701

Time (hr)	SHR-1701, 3 mg/kg	SHR-1701, 10 mg/kg	SHR-1701, 30 mg/kg
0	0.00 ± 0.00	0.00 ± 0.00	0.00 ± 0.00
2	109.27 ± 14.41	90.35 ± 11.82	120.96 ± 10.54
24	109.71 ± 10.03	99.76 ± 6.82	105.69 ± 5.99
168 (7d)	94.62 ± 33.68	85.85 ± 20.42	141.73 ± 27.28
336 (14d)	9.92 ± 9.72	51.64 ± 17.34	157.67 ± 30.59
504 (21d)	2.84 ± 1.93	28.59 ± 18.35	65.32 ± 21.66
720 (30d)	6.47 ± 2.46	10.59 ± 4.74	50.19 ± 18.78
1008 (42d)	9.72 ± 2.51	21.45 ± 9.08	54.14 ± 13.65

(%, Mean ± SE, n=6)

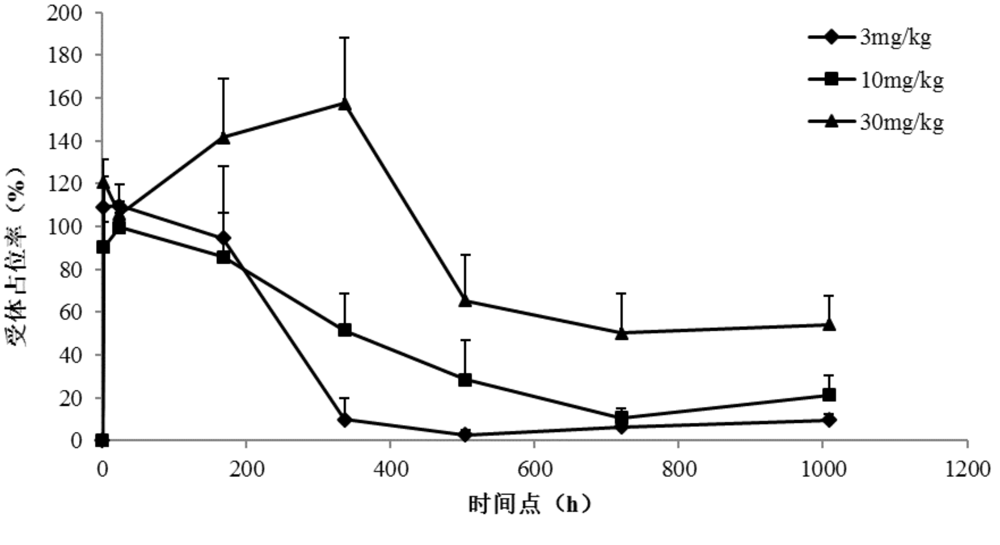

Figure 8 T cell receptor occupancy in cynomolgus monkeys administered a single dose of SHR-1701 intravenously

1.3. Progress in clinical studies of SHR-1701

So far, two phase I studies (SHR-1701-I-101 and SHR-1701-I-102) of SHR-1701 for the treatment of have been conducted in advanced malignant solid tumor patients who have failed/showed intolerance to multiple lines of systemic treatment in the past. By November 13, 2019, a total of 65 subjects were enrolled in the two studies (n=41 for Study SHR-1701-I-101, n=24 for Study SHR-1701-I-102). During the dose escalation study, maximum tolerated dose (MTD) had not been achieved with the regimen 30 mg/kg q2w, suggesting that the drug has good safety profile. Results from the two phase I studies were summarized; a total of 43(67%) subjects experienced treatment-related adverse events (TRAEs), mostly of Grade 1-Grade 2 in severity.

The most common TRAEs were: ALT increased (16%), AST increased (16%), bilirubin increased (16%), anemia (14%) and proteinuria (12%). There were 8 occurrences of Grade 3 TRAEs, including 2 occurrences of anemia, 2 occurrences of DBIL increased, 1 occurrence of ALT increased, 1 occurrence of AST increased, 1 occurrence of immune-related pneumonia and 1 occurrence of Lichen planus. No TRAE of severity ≥ Grade 4 occurred. One (1) occurrence of DLT was observed in the 20 mg/kg, q2w Dose Group; the DLT was Grade 3 immune-related pneumonia, which had resolved after active treatment. No obvious correlation was identified between dose level and overall incidence of TRAEs/incidence of TRAEs of Grade ≥3.

In the two studies, the drug's efficacy was evaluable in 47 subjects, 8 of whom had achieved PR, 15 had SD, 4 had PD but continued to be on study treatment and remained stable. Of the 8 subjects who had PR, 1 was in 3 mg/kg q3w Group, 2 in 10 mg/kg q3w Group, 2 in 20 mg/kg q3w Group, and 3 in 30 mg/kg q3w Group. Of the 4 PC patients enrolled in this study, 1 (pancreasacinous cancer) patient had achieved PR, 1 patient had SD, 2 had PD; the PC patient who had achieved PR in the study showed disease progression in the past after treatment with 3rd line therapy. In this study, the patient achieved PR in 3 consecutive efficacy assessment and had a PFS of 175 days.

By data cut, a total of 59 subjects in the two studies had completed determination of SHR-1701 concentration in their PK blood samples; the data were used for calculating single-dose PK parameters by noncompartmental analysis (NCA) after the first dose. Summarized in Table 11 are the single-dose PK parameters of SHR-1701 after the first dose. Presented in Figure 9 is the average concentration-time curve (semi-logarithmic scale) of the drug.

Table 11 Summary of single-dose PK parameters (Mean±SD) of SHR-1701 after the first dose

PK Parameter	Unit	1 mg/kg (N=1)	3 mg/kg (N=6)	10 mg/kg (N=14)	20 mg/kg* (N=25)	30 mg/kg* (N=13)
$AUC_{0\text{-}t}$	h*μg/mL	3322	11590±4513	41756±8883	66225±23374	144783±33521
AUC_{INF}	h*μg/mL	3604	14038±2998	49325±11025	89268±35037	195440±37974
$T_{1/2}$	h	142	148±22	172±41	186±64	234±145
Cl	mL/h	15.82	14.70±2.96	13.37±2.55	16.04±8.50	10.78±3.79
Vss	mL	3224	3037±319	3239±628	3911±1323	3203±1277

* Since the calculation was for determining the PK parameters after the first dose, subjects who had been administered at 20 mg/kg and 30 mg/kg dose levels at different dosing frequencies (q2w and q3w) were pooled for the analysis.

After the first dose, the elimination half-life of SHR-1701 was 5 - 10 days, and showed a trend of extending in high-dose group. After the first dose (dose range: 1 mg/kg - 30 mg/kg), the drug's in vivo exposure increases with dose in a somewhat non-linear manner. The drug's clearance rate showed a decreasing trend, and the elimination half-life was extended in the 30 mg/kg dose group. The drug's exposure (AUC_{inf}) in 30 mg/kg group was about 2.2 time that in the 20 mg/kg dose group; and the increase of exposure was non-linear, and similar to that of the drug M7824 (C_{max} and AUC in 30 mg/kg q2w group were 1.8~2 times those in the 1200 mg q2w dose group); this might be caused by target-mediated drug disposition (TMDD). When RO is saturated, the clearance rate of drug decreases.

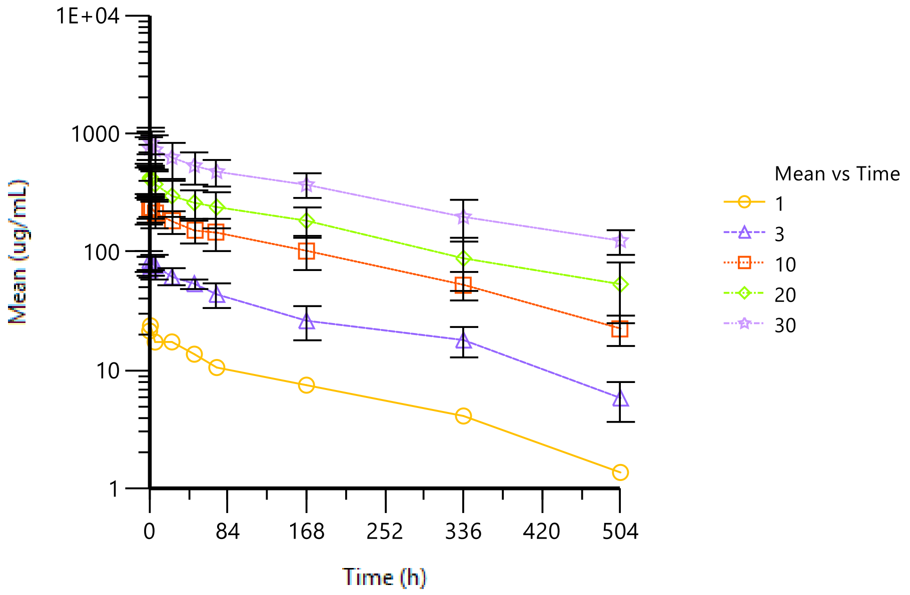

Figure 9 Mean drug concentration-time curve (semi-logarithmic scale) of SHR-1701 after the first dose

RO samples had been collected from every subject as scheduled for determining the RO of PD-L1 on surface of CD3+ cells in peripheral blood. By data cut, assay of RO samples had been completed for 53 subjects. For information on the RO of PD-L1 in subject peripheral blood by dose group, refer to Figure 10

In all dose groups, RO of PD-L1 in peripheral blood became saturated at postdose 72h and remained saturated in spite of the metabolism of the drug. Except for 1 subject in the 10 mg/kg q3w dose group, whose PD-L1 RO began to decrease continuously starting from Cycle 2, All of the rest subjects' PD-L1 RO remained saturated after multiple doses.

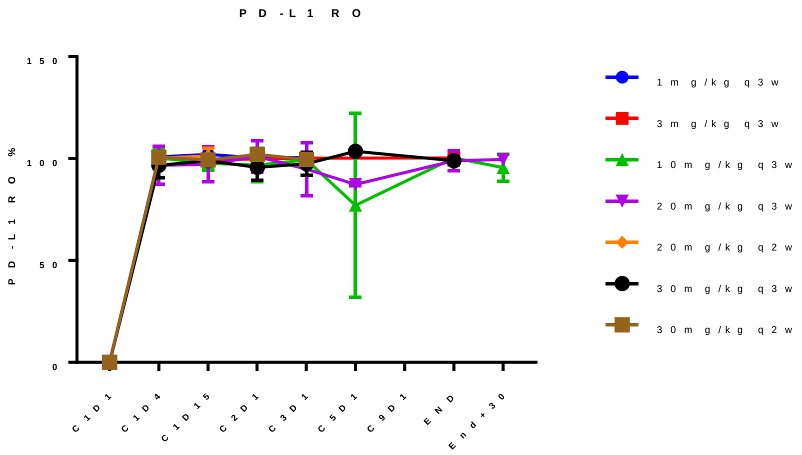

Figure 10 PD-L1 RO in a phase I study of SHR-1701

Serum samples had been collected from every subject as scheduled for determination of the level of free TGFβ1 in peripheral blood. By data cut, determination of free TGFβ1 level in peripheral blood had been completed for 55 subjects. For information on the drug's inhibition of free TGFβ1 in peripheral blood of subjects by dose group, refer to Figure 11.

In all dose groups, SHR-1701 potently suppressed the TGFβ1 level in peripheral blood, and its suppression remained at high level in spite of the metabolism of the drug. Its suppression of TGFβ1 in peripheral blood remain at high level after multiple doses.

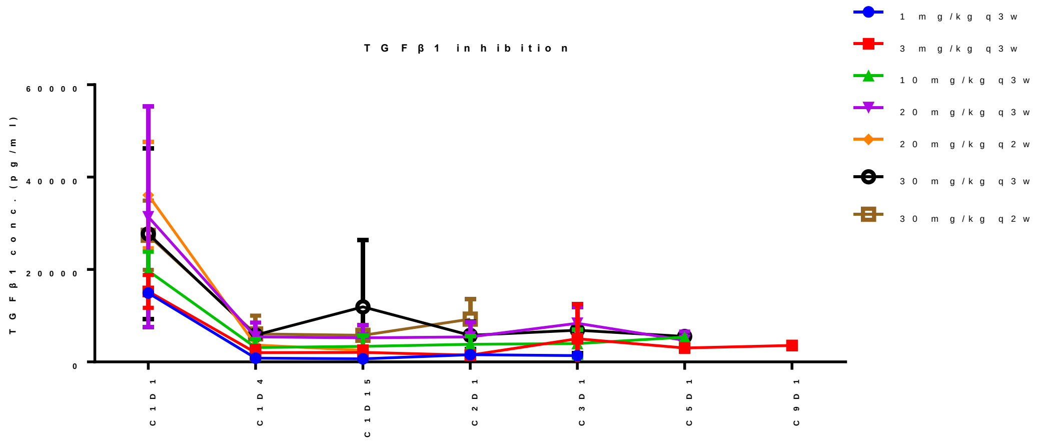

Figure 11 TGFβ suppression in a phase I study of SHR-1701

Early PK data revealed that the drug's exposure (AUC_{inf}) increased non-linearly with dose (from 20 mg/kg to 30 mg/kg); however, in early tolerability observations, no DLT event was observed. Therefore, the use of 30 mg/kg, q3w as RP2D for subsequent exploratory studies is acceptable from the perspective of tolerability since available early tolerability data revealed no potential risk.

After a comprehensive analysis of existing safety, effectiveness, PK and PD data from the two phase I studies, it was recommended that 30 mg/kg, q3w be used as RP2D for subsequent clinical studies. The RP2D hopefully will further improve the drug's efficacy and currently available safety data reveal no potential risk.

1.4. Potential risks and benefits

1.4.1. Safety findings in pre-clinical studies

SHR-1701 is an anti-PD-L1/TGF-βRII bifunctional fusion protein that can specifically block the interactions between PD-1/PD-L1 and neutralize the TGFβ in tumor micro-environment for breaking the immunotolerance of tumors and restoring the tumor cell killing function of tumor-specific T cells in order to eliminate tumor.

Results of toxicity studies of SHR-1701 in SD rats and cynomolgus monkeys: the major toxicity target organs of SHR-1701 were the hematopoietic/immune system (histological findings: thymus weight decreased, slight to mild cortical lymphocyte decreased, and slight HGB decreased). In SD rats and cynomolgus monkeys administered repeat doses, SHR-1701 was found to be somewhat immunogenic in rats and potently immunogenic in cynomolgus monkeys.

1.4.2. Known potential risks

The administration of any drug may give rise to unexpected or even serious AEs. Presently, SHR-1701 is at phase I early clinical development stage. By November 13, 2019, the most common TRAEs (incidence ≥10%) included: ALT increased (16%), AST increased (16%), bilirubin increased (16%), anemia (14%) and proteinuria (12%).

According to the reported AEs in clinical trials of similar foreign products, the AEs of anti-PD-L1 mAbs targeting PD-L1 are generally milder than those of mAbs targeting PD-1; moreover, mAbs targeting PD-L1 are generally better tolerated by patients. The most common AEs (affecting ≥ 20% of patients) included: fatigue, appetite decreased, nausea, urinary tract

infection, pyrexia, and constipation, etc.; immune-related adverse reactions (irAEs) mainly included: inflammatory colitis, thyroid function decreased, pneumonitis, etc.; most of the AEs were mild. Therefore, the drug has good overall safety. For anti-TGFβ mAb, the AEs mainly included gingival bleeding, nasal hemorrhage, headache, asthenia and skin damage; skin toxicity was the most common TRAE. It can be learnt from the disclosed AEs of the anti-PD-L1/TGF-βRII bifunctional fusion protein MSB0011359C (M-7824), only 1 subject experienced DLT (colitis) in dose-escalation study, and the drug's MTD had not been reached at its maximum dose (20 mg/kg) in the dose-escalation study.

Gemcitabine and albumin-bound paclitaxel are marked common cytotoxic chemotherapeutic drugs and have quite clear AE spectrum.

Common AEs of gemcitabine mainly involve the hemic and lymphatic system (anemia, WBC decreased, blood platelet decreased, febrile neutropenia, etc.), the digestive system (hepatic function abnormal, nausea and vomiting, diarrhea, stomatitis, etc.), the reproductive-urinary system (mild proteinuria and hematuria), skin and appendages toxicity (skin rash, itching, alopecia, etc.), the respiratory system (dyspnea, bronchial spasm, interstitial pneumonitis, etc.), systemic (influenza-like symptoms, pyrexia, headache, chills, myalgia, asthenia, anorexic, coughing, nasopharyngeal, asthenia, malaise and sweating, somnolence, allergic reactions and radiotoxicity, etc.), the cardiovascular system (edema, hypotension, myocardial infarction), etc.

Common AEs of albumin-bound paclitaxel include: myelosuppression (e.g., neutropenia, blood platelet decreased, anemia; peripheral neuropathy; arthromyalgia; allergic reactions; nausea, vomiting; catarrh; motor neurotoxicity, sensory neurotoxicity; cardiac events; alopecia; injection site reactions; diarrhea, etc.

In light of the safety findings from non-clinical studies and early phase I clinical studies and the known safety risks of similar drugs, close attention should be paid to the following AEs during this clinical trial:

- Hematopoietic/immune system reactions
- Infusion reaction
- Skin toxicity events
- Reproductive toxicity events

1.4.3. Risk control plan

The following risk control measures have been proposed in the study protocol in light of the above-mentioned potential risks in order to minimize subject’s risk with the administration of the investigational drug.

- Hematopoietic/immune system reactions

Applicable tests including hematology, immunology will be carried out in the screening period; inclusion/exclusion criteria will be established(for more details, refer to 4 Selection and withdrawal of subjects); relevant indexes will be monitored during the study treatment period; in case of the occurrence of hematopoietic/immune system AEs, it is recommended that the AEs be treated in accordance with the guidelines provided in the protocol (for more details, refer to 5.5 Concomitant medications and concomitant therapies and 5.6 Recommended symptomatic treatment of common AEs).

- Infusion reaction

During the study, investigators need to pay attention to subjects’ infusion/allergic reactions, especially acute immune-mediated adverse reactions (including cytokine storm), and train the

medical staff involved in the study on emergency treatment of relevant allergic reactions; if relevant infusion reactions occur during the study treatment period, the corresponding treatment guidelines specified in the protocol should be followed to administer corresponding treatment(for more details, refer to 5.6.2 Management).

- Skin toxicity events

Prior to the start of the study, investigators should inform subjects of potential skin adverse reactions(ARs) so that subjects know the risk and how to contact the investigators in case of the occurrence of ARs; during the study treatment period, investigators should regularly monitor and perform relevant examinations/tests of subjects; if a subject experiences skin related AEs, the corresponding treatment guidelines specified in the protocol should be followed to administer corresponding treatment (refer to 5.6.1 Rules for Safety Management of Immuno-oncology Agents).

- Reproductive system toxicity events

It is expressly prohibited that pregnant and breast-feeding women and women who plan to have a baby be included in the study. During the screening period, female subjects should receive blood pregnancy test to determine their pregnancy state; in case of pregnancy during the study treatment period, the affected subject must discontinue her study treatment. Investigators need to follow up the pregnancy outcome and report it to the sponsor.

1.4.4. Known potential benefits

Immediate potential benefits: Subjects will receive better care and attention, free disease monitoring and treatment with the investigational drug; after treatment, subjects' disease condition may be controlled; investigators will have the opportunity to use the latest medication on the subjects.

Long-term potential benefits: The study will be helpful for investigators to improve their academic level and increase their academic influence and good for China's medical innovation and R&D; moreover, it will improve the international competitiveness of China's medical cause and eventually improve the treatment and survival of tumor subjects.

2. Study Objectives and Endpoints

2.1. Study Objectives

This study consists of 2 phases, Phase 1 (Phase Ib) is for dose-finding of the combination therapy, Phase 2 (Phase II) is for efficacy extension.

Primary Objective of Phase Ib:

- To evaluate the safety and tolerability of the combination of SHR-1701 and gemcitabine and albumin-bound paclitaxel as first-line therapy for advanced/metastatic pancreatic cancer (PC) patients and determine the recommended phase 2 dose (RP2D) of SHR-1701 in the combination therapy regimen.

Secondary Objectives of Phase Ib:

- To preliminarily evaluate the efficacy of the combination of SHR-1701 and gemcitabine and albumin-bound paclitaxel as first-line therapy for advanced/metastatic PC patients.
- To evaluate the pharmacokinetics (PK) properties of SHR-1701;

Primary Objective of Phase II:

- To evaluate the efficacy of the combination of SHR-1701 and gemcitabine and albumin-bound paclitaxel as first-line therapy for advanced/metastatic PC patients by objective response rate (ORR).

Secondary Objectives of Phase II:

- Key secondary study objective: To evaluate the efficacy of the combination of SHR-1701 and gemcitabine and albumin-bound paclitaxel as first-line therapy for advanced/metastatic PC patients by OS rates at 9-month.
- To evaluate the efficacy of the combination of SHR-1701 and gemcitabine and albumin-bound paclitaxel as first-line therapy for advanced/metastatic PC patients by other efficacy indicators.
- To further evaluate the safety and tolerability of the combination of SHR-1701 and gemcitabine and albumin-bound paclitaxel as first-line therapy for advanced/metastatic PC patients;
- To evaluate the pharmacokinetics (PK) properties of SHR-1701;

Exploratory Objectives for Phase Ib and Phase II:

- To evaluate the immunogenicity of SHR-1701;
- To explore the relationship between biomarkers and clinical efficacy by collecting tumor tissue samples for analysis of biomarkers, including PD-L1 expression of tumor tissue, tumor infiltrating lymphocytes (TILs), TGFβ/pSmad pathway related protein level, tumor micro-environment gene expression, etc.

2.2. Study Endpoints

Primary Endpoint of Phase Ib:

- RP2D of SHR-1701 in the combination therapy regimen.

Secondary Endpoints of Phase Ib:

- Occurrence of clinically significant toxicity events, incidence and severity of adverse events (AEs) and serious adverse events (SAEs) evaluated per NCI-CTC AE 5.0, vital signs, electrocardiograms (ECG) and lab abnormalities and other safety indicators;
- Preliminary efficacy endpoints: effectiveness indicators such as ORR, diseases control rate (DCR), best overall response (BOR), progression-free survival (PFS), time to progression (TTP), duration of response (DoR), OS rates at 6-month, OS rates at 9-month, OS rates at 12-month, overall survival (OS), assessed by investigator per RECIST 1.1;
- PK parameters, including C_{trough} and C_{max}, of SHR-1701

Primary Endpoint of Phase II:

- ORR, assessed by investigator per RECIST 1.1.

Secondary Endpoints of Phase II:

- Key secondary endpoint: OS rates at 9-month;

- Other secondary efficacy endpoints: Effectiveness indicators such as DCR, BOR, PFS, TTP, DoR, OS rates at 6-month, OS rates at 12-month, OS, etc., assessed by investigator per RECIST 1.1;
- PK parameters, including C_{trough} and C_{max}, of SHR-1701

Endpoints for Phase Ib and Phase II Exploratory Study:

- Evaluation of the immunogenicity of SHR-1701: The positive status of anti-SHR-1701 antibodies (including ADA and Nab) formed during the study period in relation to the baseline will be analyzed in light of the plasma concentration, safety and effectiveness data of SHR-1701.
- The relationship between clinical efficacy and biomarkers, including PD-L1 expression of tumor tissue, tumor infiltrating lymphocytes (TIL), TGFβ/pSmad pathway related protein level, tumor micro-environment gene expression, etc., will be explored.

3. Study Design

3.1. Overall design of the clinical trial

This is an open-label, multi-center Phase Ib/II clinical study consisting of two phases: Phase Ib for dose-finding of the combination therapy regimen, and Phase II for efficacy extension. Planned enrollment: A total of 54-60 patients naive to systemic therapies for advanced/metastatic PC will be enrolled.

Phase Ib: Dose-finding stage

This stage is intended to observe and evaluate the safety and tolerability of the combination of SHR-1701 and gemcitabine and albumin-bound paclitaxel as first-line therapy for advanced/metastatic PC patients, and determine RP2D of SHR-1701 in the combination therapy regimen.

Dose-finding will be carried out according to a modified “3+3” scheme; the predefined doses of SHR-1701 are 30mg/kg, q3w (i.e., every 3 weeks/21 days), and 20mg/kg, D1, q3w. The dose level 30mg/kg is based on the RP2D of SHR-1701 determined in a phase I clinical trial of SHR-1701 monotherapy. In this study, dose finding for SHR-1701 will start from 30mg/kg, D1, q3w; if the dose is not tolerated, then the dose level of SHR-1701 will be lowered to 20mg/kg, D1, q3w to continue the exploration.

The first 6 enrolled subjects will be treated with combination chemotherapy regimen a(SHR-1701 30mg/kg, D1, q3w; gemcitabine 1000mg/m^2, D1, D8, q3w; albumin-bound paclitaxel 125mg/m^2, D1, D8, q3w); if NMT 1(proportion<0.33) of the 6 subjects experiences clinically significant toxicity, then the efficacy extension phase (phase II) of the study will be carried out with this dose level;

If NLT 2 (proportion≥0.33) of the 6 subjects at the dose level experience clinically significant toxicity, then 6 additional subjects will be enrolled for treatment with combination chemotherapy regimen b (SHR-1701 20mg/kg, D1, q3w; gemcitabine 1000mg/m^2, D1, D8, q3w; albumin-bound paclitaxel 125mg/m^2, D1, D8, q3w). If the number of patients who experience clinically significant toxicity is still NLT 2 (proportion≥0.33), then it is up to the SMC to determine the dose level/mode of administration for subsequent study after discussion.

For details of the dose-finding procedures in Phase Ib study, refer to Table 1 List of dose levels

Cycle 1 (21 days) of the co-medication will clinically significant toxicity observation period, in which those subjects who complete 1 cycle of the co-medication will receive safety assessment.

If a subject is administered a total dose of the investigational drug less than 90% of the specified value in Cycle 1 (due to, for example, discontinuation of dosing because of infusion reaction) and experiences no clinically significant toxicity, the subject will be excluded from the calculation of the occurrence of clinically significant toxicity at the specified dose level of the investigational drug; moreover, the subject will be replaced by an additionally recruited subject who will be administered the drug at the current dose level.

If a subject discontinues his/her study treatment during the clinically significant toxicity observation period after the first dose for reason(s) other than clinically significant toxicity, then the subject will be replaced by an additionally recruited subject. During the study period, no upregulation or downregulation of the dose level of SHR-1701 is permitted. Likewise, no regulation of the dose level of any component of the co-medication is permitted during the clinically significant toxicity observation period.

All subjects (n=6) on treatment with current dose level have to complete the clinically significant toxicity observation period prior to the start of the study at the next dose level. At the same dose level, the interval for enrollment of subject in the study will be decided by SMC according to available safety/tolerability data.

Phase II: Efficacy extension stage

Once the RP2D for SHR-1701 in the combination chemotherapy regimen is determined on the basis of the preliminary safety, PK and efficacy data from phase Ib, the study will proceed to efficacy extension stage and enrollment of subjects will continue until 54 patients are included for treatment at the same dose level. The efficacy and safety of the therapy for the treatment of advanced/metastatic PC will be further explored.

The study consists of a screening period, a treatment period, and a follow-up period.

Screening period: This period is NLT 28 days long, starting from the signing of ICF by subjects to the first dose; it is acceptable to have the subjects to receive imaging examinations within the 28 days prior to the first dose.

Treatment period: Subjects who complete and pass the screening period examinations and evaluations will proceed to treatment period of the study. Each subject will receive treatment with SHR-1701 in combination with chemotherapy ((gemcitabine+albumin-bound paclitaxel) in 21-day cycle(s). The treatment continues until disease progression, intolerable toxicity, subject’s discontinuation with the study treatment or withdrawal from the study, or, ineligibility of subject to continue the study treatment in the opinion of the investigator, which ever occurs first, or until termination of the study by the sponsor.

During the treatment process, if the investigator determines that a subject cannot tolerate one or two component drugs of the combination therapy regimen, the investigator may, after thorough assessment of the subject's clinical benefits from the study treatment, retain the subject in the study for receiving treatment with other tolerable drug therapy.

In the first 12 months of the study treatment period (the first 16 treatment cycles), subjects will receive an imaging evaluation of their tumors every 6 weeks (±7 days); afterwards, subjects will receive an imaging evaluation of their tumors every 3 cycles (i.e., (9 weeks (±7 days)).

During the study treatment, if the investigator determines that a subject who shows disease progression for the first time can still benefit from the treatment (without: any symptom and sign of clinical progression, performance status decreased, fast progression of disease, especially at important anatomical parts, such as spinal cord compression), and the subject can tolerate the study treatment and voluntarily wish to stay in the study, the treatment with SHR-1701 alone or in combination with chemotherpay can be continued (for more details, refer to 4.5) until another

imageologically-confirmed progression of the disease or the disappearance of clinical benefits in the subject in the opinion of the investigator(whichever occurs first).

Follow-up period: The follow-up period can be divided into safety follow-up period and survival follow-up period. The safety follow-up period starts from the last study treatment and ends 90 days after the last study treatment; during this period, the subjects are followed up once every 30 days (±7 days). At Safety Follow-up Visit 1, the subjects should go to the study site for assessment; Safety Follow-up Visits 2 and 3 can be done by telephone. At the end of the safety follow-up period, the subjects proceed to the survival follow-up period. The survival follow-up period lasts until subject's death, lost to follow-up, withdrawal of ICF or termination of the study by the sponsor. During the period, subjects are followed up once per month by telephone other valid method for collecting survival information and subsequent treatment information. Subjects without radiologically-confirmed disease progression should receive imaging evaluation at the efficacy evaluation frequency specified in the study protocol until disease progression, death, lost to follow-up, withdrawal of ICF, the start of other antitumor therapy or termination of the study by the sponsor.

During the study, safety data will be collected continually. Safety and tolerability assessment: The occurrence and severity of AEs will be determined according to CTC AE v5.0. During the study, subjects will be continually evaluated by laboratory tests, vital signs measurements, ECOG score, physical examination, ECG examinations and AEs.

All subjects will be collected PK, immunogenicity, and tumor tissue samples for PK and immunogenicity assessment of SHR-1701 and analysis of biomarkers.

3.2. Clinically significant toxicity

Severity of adverse events will be graded according to NCI CTCAE Ver. 5.0. At phase I study stage, the occurrence of any of the following toxic and side reactions in the tolerability observation period (the 21 days after the first dose) will be considered as clinically significant toxicity if they are possibly or definitely related to SHR-1701:

1) Non-hematological toxicity of Grade ≥3, with the exception of the following:
   a) Nausea, vomiting, diarrhea, or asthenia of Grade ≥3, which ameliorates to Grade ≤2 within 7 days of supportive treatment;
   b) Grade 3 ALT/AST increased, which ameliorates to Grade ≤2 within 7 days of treatment;
   c) Grade 3 infusion reaction or Grade 3 pyrexia lasting for NMT 6h after supportive treatment;
   d) Grade 3 skin toxicity, which ameliorates to Grade ≤2 within 7 days of symptomatic/supportive treatment;
   e) Any symptomatic lab abnormality of Grade ≥ 3, which ameliorates to Grade ≤2 within 7 days of symptomatic/supportive treatment;
   f) Any blood amylase or lipase laboratory abnormality of Grade ≥ 3 that has no clinical manifestation, with the exception of pancreatitis.
2) Hematotoxicities of Grade ≥ 3, including any of the following:
   a) Platelet count decreased of Grade 3, lasting for ≥7 days or with significant clinical hemorrhage symptom(s);
   b) Neutropenia of Grade 3 or febrile neutropenia of Grade 3 with infection;

c) Grade 4 neutrophils reduced, lasting for ≥3 days;

d) Any other hematotoxicity of Grade ≥4.

3) Other unexpected, persistent, intolerable toxicities of Grade ≥2, which, in the opinion of SMC, justify the discontinuation of the SHR-1701 treatment.

3.3. Safety Monitoring Committee

An SMC consisting of external investigators and the sponsor's safety review team will be established for this study. In line with the study objectives specified in the protocol, the investigators and the sponsor will have their internal safety review team to regularly carry out safety review by virtue of their medical and statistical capability. The safety reviews will cover the individual and pooled data in the safety and clinical databases. The review actions include:

- Monitoring of SAEs in line with regulatory guidance.
- Regular teleconferences and/or meetings, in which the investigators and the sponsor will discuss on treatment-emergent adverse events (TEAEs) and abnormal changes in laboratory investigation results to determine the treatment's safety data and risk/benefit ratio.
- Prior to proceeding to phase II study, the investigator and the sponsor should discuss on the available subject safety data and RP2D by meeting (including teleconferences or webmeeting) and/or emails as far as possible.

3.4. PK, immunogenicity and biomarker studies

3.4.1. PK blood sampling time points

PK blood samples will be collected from all subjects at the following time points: within 0.5h before dosing and within 10min after dosing of SHR-1701 on C1D1, C2D1, C4D1; within 0.5h before dosing of SHR-1701 on C7D1 and on D1 after every 6 cycles beyond, on the date of the subject's end of treatment, and after 30 days (±7 days) of the last dose. The blood sampling time windows after the end of dosing do not include the time needed for tube cleaning. At each time point, 3.5 mL of venous blood will be collected into a serum separator tube for PK study of SHR-1701.

PK blood sampling should be carried out per the study schedule; if necessary, however, it is acceptable to collect unscheduled PK blood samples for PK analysis.

3.4.2. Immunogenicity blood sampling time points

Immunogenicity blood samples will be collected from all subjects. One (1) sampling is to be performed within 0.5h before dosing of SHR-1701 on C1D1, C2D1, C4D1, C7D1, and on D1 of cycles beyond C7 at an interval of 6 cycles, on the subject's end of treatment day, and after 30 days (±7 days) of the last dose. At each time point, 5 mL of venous blood will be collected into a serum separator tube for ADA and Nab detection.

Immunogenicity blood sampling should be carried out per the study schedule; if necessary, however, it is acceptable to collect unscheduled immunogenicity blood samples for immunogenicity analysis.

3.4.3. Collection of tumor tissue samples

Subjects should provide their tumor tissue samples. Requirements: neutral formalin-fixed, paraffin-embedded unstained tumor sections. It is recommended that 5-10 sections of 3~5 μm thick (or more samples if the tissue samples are small or are aspirate samples) be collected and submitted to the study's designated central laboratory for biomarkers (including PD-L1

expression, TILs, TGFβ/pSmad pathway-related protein, tumor micro-environment gene expression, etc.) assays to explore the relationship between the biomarkers and the drug's efficacy. The tumor tissue samples may be archived ones (prior to the treatment) or freshly collected ones (freshly collected ones are preferred). For the harvesting/collection and disposal procedures of tumor specimens, refer to Laboratory Manual.

For a few subjects who are unable to provide adequate tissue samples, it is up to the investigator and the sponsor to determine the eligibility of subject patients for the study by discussion.

4. Selection and withdrawal of subjects

4.1. Enrollment Criteria

Subjects have to meet all of the following criteria for inclusion

1. Histologically or cytologically diagnosed with PC, with evidence(s) of unresectable locally advanced or distant metastasis disease, which is histologically or cytologically verified to be duct adenoma or acinic cell carcinoma.
2. Naive to systemic treatment for advanced/metastatic PC; disease progression after more than 6 months of past neoadjunctive treatment or postoperative adjunctive treatment. Palliative radiotherapy is permitted; however, the study medication cannot be started in less than 2 weeks of the completion of the palliative radiotherapy. Previous treatment with anti-tumor TCM preparation(s) is permitted; however, such treatment has to be discontinued at least 2 weeks prior to the start of the study medication.
3. 18 - 70 year-old, male or female.
4. ECOG PS score 0 - 1.
5. Life expectancy ≥ 12 weeks.
6. All toxicities from previous anti-tumor therapy or surgical procedures have ameliorated to Grade 0 - Grade 1 (per NCI CTCAE 5.0) or to levels stipulated in eligibility criteria. Excluding other toxicities (e.g., alopecia, lassitude, hearing injury, etc.) which, in the opinion of investigator, pose no safety risks to the patient.
7. With at least 1 measurable disease that meets RECIST 1.1 criteria.
8. Adequate organ and bone marrow functions, meeting the following criteria(patients need to avoid corrective treatment within the 14 days prior to routine hematology):
   a) WBC≥ 3,000/mm^3 (3 × 10^9/L);
   b) ANC≥ 1,500/mm^3 (1.5 × 10^9/L);
   c) Lymphocyte count ≥ 500/mm^3 (0.5 × 10^9/L);
   d) PLT ≥ 100,000/mm^3(100 × 10^9/L);
   e) Hb ≥ 10 g/dL (100 g/L);
   f) Serum ALB ≥ 2.9 g/dL
   g) Serum creatinine ≤ 1.5×ULN or creatinine clearance ≥ 50 ml/min;
   h) TBIL ≤ 1.5×ULN;
   i) AST/SGOT or ALT/SGPT ≤ 3×ULN, or ≤ 5×ULN for patients with metastases to liver;
   j) INR ≤ 1.5, PT and APTT ≤ 1.5×ULN;

k) Protein urine <2+; if protein urine is ≥2+, then the patient's 24-h urine protein has to be ≤1g to be eligible.

9. Availability of fresh or archived tumor tissue samples, preferably fresh ones. Formalin-fixed, paraffin-embedded tumor tissue blocks or unstained tumor specimens, 5-10 sections.

   Note: if the above-mentioned tumor tissue specimens are unavailable, the sponsor's medical monitor has to be consulted to determine the patient's eligibility. For the harvesting/collection and disposal procedures of tumor specimens, refer to Laboratory Manual.

10. Female subjects of childbearing age have to be tested negative in a serum pregnancy test within 7 days prior to the start of study medications and willing to take a medically recognized effective contraceptive measure (e.g., intra-uterine device, contraceptives or condom) during the study and within 3 months after the last dose of the study medications to be eligible for the study; male subjects who have female partners of childbearing age should be surgically sterilized or willing to take effective contraceptive measure(s) during the study and within 3 months after the last dose of the study medications.

11. A signed ICF, stating that the subject is willing and able to abide by the scheduled follow-up visits, study treatment, laboratory investigations and other study procedures.

4.2. Exclusion Criteria

A subject will be excluded from the study if he/she meets any of the following criteria:

1. Previous use of TGFβinhibitor, anti-PD-1/PD-L1 antibody, anti-PD-L2 antibody, anti-CD137 antibody, anti-CTLA-4 antibody or other drug/antibody that acts on T-cell costimulatory signaling or checkpoint pathway.

2. Previous allergy to study medications or any excipient of them, or previous serious anaphylactic reaction to other mAb.

3. Any of the following circumstances:

   a) Any major operations (with the exception of diagnostic biopsy) within 28 days prior to the first dose of the study medications.

   b) Use of any immunosuppressants (with the exception of corticosteroids nasal spray and inhaler, or systemic steroids at physiological dose (i.e., prednisone at NMT 10 mg/d, or other corticosteroids at equivalent physiological dose) within 7 days prior to the fist dose of the study medications.

   c) Use of any immunomodulators within 14 days prior to the first dose of the study medications.

   d) Inoculation with any live attenuated vaccines within 28 days prior to the first dose of the study medications or within 60 days of the end of treatment with the study medications.

4. Previous or present metastasis to CNS.

5. Risk of any symptomatic, life-threatening complications (including uncontrolled effusion of moderate or above amount into pleural space, pericardium, abdominal cavity) of advanced disease that have disseminated to viscera.

6. Presence of any active autoimmune diseases or expected relapse of such diseases

(including but not limited to: autoimmune hepatitis, interstitial pneumonitis, uveitis, enteritis, hepatitis, hypophysitis, vasculitis, nephritis, hyperthyroidism, hypothyroidism[with the exception of those whose condition can be controlled by hormone replacement therapy]; (patients who have dermatoses(e.g., vitiligo, psoriasis, etc.) alopecia, type I diabetes mellitus that require no systemic treatment, or in their childhood suffered from asthma which has completely resolved in their adulthood and requires no intervention can be included; however, patients who have asthma that requires bronchodilator for medical intervention should not be included).

7. Presence/history of other active malignant tumors within 5 years prior to the study, with the exception of basal cell carcinoma or squamous cell carcinoma, superficial bladder cancer, carcinoma in situ of cervix, carcinoma in situ of breast ductal and thyroid papillary carcinoma that have been cured by topical therapy.
8. HIV infection or AIDS, treatment naïve active hepatitis (hepatitis B, defined as: HBsAg+, HBV-DNA ≥ 500 IU/ml, plus hepatic function abnormal; hepatitis C, defined as: HCV-Ab +, HCV-RNA > LLOD of analytical method, plus hepatic function abnormal) or coinfection of HBV and HCV concurrently.
9. Presence of clinically significant acute or chronic pancreatitis.
10. Presence of other clinically significant acute or chronic infections (determined by the investigator according to patient’s history of exposure to source of infection, etiology test positive results, clinical symptom(s), sign(s), imaging finding(s), and the need for clinical intervention).
11. Presence/history of any of the following clinically significant cardio-/cerebro-vascular diseases within 6 months prior to the study: myocardial infarction/brain infarction/hematencephalon, serious/unstable angina, congestive heart failure (cardiac dysfunction of severity ≥ NYHA Class 2) and clinically significant supraventricular or ventricular arrhythmia that requires clinical intervention.
12. Systemic use of any antibiotics for ≥ 7 days within 4 weeks prior to the first dose; pyrexia >38.5°C of unknown cause during the screening period/prior to the first dose (patients with pyrexia attributable to tumor may be included at the investigator’s discretion).
13. Known history of homologous organ transplant or history of homologous hematopoiesis stem cell transplant.
14. Participation in clinical trial of any other drug within 4 weeks or 5 times the half-life of the drug of the previous clinical trial after the last dose of the said drug, whichever is longer, prior to the fist dose of the current study.
15. Female patients in lactation period. Breast feeding have to be discontinued after the administration of SHR-1701 for treatment due to the unknown risk of the drug to baby.
16. Known history of abuse of psychotropics abuse or doping.
17. Other serious physical or psychiatric disorders or lab abnormalities that may increase the risk of the patient’s participation in the study, or interfere with the study results, and other conditions which, in the opinion of the investigator, render the patient unsuitable for the study.

4.3. Criteria for re-screening

Re-screening is permitted in this study; in other words, subjects who have signed the ICF but haven’t started the study treatment due to their failure to meet the eligibility criteria during the

screening can be re-screened. Investigation results obtained in the specified time windows during the first screening period can be used in place of corresponding overlapped screening investigations if such results meet the eligibility criteria. At re-screening, it is required that new Subject Number be assigned.

4.4. Withdrawal or discontinuation by subjects

Subjects may withdraw their ICF at any time and exit the study at will. Subjects may also be withdrawn from the study by investigator depending on the AE(s) they experience. Furthermore, subjects may be withdrawn from the study by investigator or the sponsor for their failure to meet the eligibility criteria, violation of the study protocol, or out of management and/or other safety considerations.

4.4.1. Criteria for study withdrawal

Reasons for withdrawal from the study may include:

- Withdrawal of ICF and/or refusal to receive further follow-up visits by subject;
- Other conditions which, in the opinion of the investigator, justify the withdrawal of the subject from the study; for example, the subject is imprisoned, quarantined, or incapacitated from free expressing his/her will, or significantly violates the protocol;
- Lost to follow-up;
- Death of subject;
- The study is terminated by the sponsor.

The reason(s) for subject withdrawal should be recorded in CRF and the subject's medical record.

It is noteworthy that the withdrawal of ICF includes subject's withdrawal of consent to the investigator's further contact or withdrawal of previous authorization to someone who provides further information of the subject. If possible, subjects who decide to withdraw from the follow-up should inform the investigator of their decision in writing. The investigator should explain and record the occurrence of withdrawal of ICF and clarify whether it is a withdrawal from treatment with the investigation drug alone or a withdrawal from treatment with the investigation drug and the follow-up visits specified in the study protocol.

4.4.2. Criteria for discontinuation of study treatment

Discontinuation of study treatment does not mean withdrawal from the study. Subjects who discontinue study treatment must continue to complete the remaining study visits as required by the protocol. Study medication has to be discontinued in subject in any of the following cases:

- The subject asks to discontinue his/her treatment with study medication;
- The subject's response evaluation results meet the criteria for disease progression, and the subject fails the criteria for remain on treatment after disease progression (refer to 4.5);
- Pregnancy in female subjects during the study;
- Intolerable toxicity in subject despite dose modification, or any AE, laboratory abnormality or other medical condition that makes the investigator believe further treatment is not in line with subject's best interest;
- General deterioration of health status that prevents continued participation in the study;
- Any significant protocol deviation (e.g., enrollment of ineligible subject) found during

the study and confirmed by the sponsor;

- Termination of the study by the sponsor;
- Other reasons which, in the opinion of th investigator, render it impossible to carry on with the study treatment.

4.4.3. Procedures for withdrawal or discontinuation

Every effort must be made to complete the protocol-specified efficacy and safety examination at the time of discontinuation of study treatment, complete safety follow-up, and record adverse events (AEs) and outcomes in full. The investigator can recommend or provide new or alternative treatments to the subject based on his/her actual condition. Non-PD subjects should be followed up as far as possible for imaging evaluation until their start of new antitumor therapy or PD.

Unlike subjects who withdraw ICF and exit the study, subjects who withdraw consent to discontinue the study treatment only will be retained in the study and should follow the protocol specified study procedures for follow-up visits.

If a subject refuses further visits, his/her survival status should be followed up continually if possible unless the subject withdraws consent to the further disclosure of information or further contact. In this case, no further study evaluations will be performed and no additional data will be collected.

4.5. Criteria for remain on treatment after disease progression

Some subjects might still benefit from treatment with the immunotherapy even after imaging evidence of PD. For example, a subject might have significant necrosis or denaturation of tumor tissue in spite of increased tumor volume; and his/her tumor CT image density might decrease; in such case, it is generally believed that the subject might benefit from his/her “remain on treatment after PD”. A subject who meets the following criteria will be eligible for remain on treatment with SHR-1701 alone or in combination with chemotherapy after PD per the definition in RECIST 1.1:

- The investigator believes that remain on treatment is in the best interest of the patient for whom it is unnecessary to start other antitumor therapy immediately;
- The subject can tolerate the study treatment;
- The subject shows no significant decrease in PS score and no significant deterioration of tumor related symptoms;
- The subject is free of signs (e.g., spinal cord compression, etc.) of rapid disease progression, especially in important topography;
- Prior to continuing with the study treatment, the subject has to sign an ICF for Remain on Treatment after PD, in which the potential risks, discomforts and other available treatment options are clearly described;
- The subject’s remain on treatment after PD must be reviewed and approved by the sponsor.

For subjects who remain on the study treatment after PD, the treatment has to be discontinued if their PD (Table 12) has been confirmed in the next tumor assessment; if the investigator still believes that a subject can benefit from his/her remain on treatment, the investigator should discuss with the sponsor for approval; if the sponsor approves, the investigator should have the subject to sing another ICF for Remain on Treatment after PD for continuing the treatment until

the subject can no longer benefit from the treatment in the opinion of the investigator. If a subject has non-confirmed PD in the next tumor assessment, he/she should continue to follow the study protocol for treatment, evaluation and follow-up visits.

Table 12 Definition of re-confirmed PD after first disease progression

	**Conditions in which a re-confirmed PD is justifiable (Meeting any of the following criteria)**	**Conditions in which a re-confirmed PD is unjustifiable (Meeting all of the following criteria)**
**Target lesion**	An increase ≥20% in tumor burden and an increase ≥5mm in absolute size from the values after the first PD	An increase <20% in tumor burden from the value after the first PD
**Non-target lesions**	Presence of definite and persistent progression of non-target lesion (qualitative) relative to the first PD	Absence of definite and persistent progression (qualitative) relative to the first PD
**New lesions**	a) Emergence of new lesion(s) since the fist PD;<br>b) Increase of previously emerging new lesion*, or emergence of other new lesion(s);	No emergence of new lesion since the fist PD;

* Criteria for determination of increase in size of new lesion: if the new lesion meets the criteria for measurable disease, the descriptions of criteria for determination of increase in size of target lesion should be followed; otherwise, the descriptions of criteria for determination of increase in size of non-target lesion should be followed.

The data of first PD as assessed by the investigator will be used for all statistical analyses involving progression information, regardless of the subject's study treatment status after the PD.

If a subject discontinues his/her treatment for reduced general condition without any objective evidence of PD, the progression status of the subject will be reported as "clinical tumor progression". Objective evidences of PD (e.g., radiologically confirmed evidences) should be collected from such subjects after their discontinuation of study treatment.

4.6. Early Termination or Suspension of the Study

The study may be terminated early or suspended if there are sufficient reasons. This may be due to decisions from the regulatory authority, opinion changes by the ethics committee, efficacy or safety issues of the investigational drug, or based on the sponsor's judgment. Furthermore, the sponsor reserves the right to terminate the development of SHR-1701 at any time. The party who decides to suspend/terminate the study will give a written notice documenting the reason for study termination or suspension to the investigator, the sponsor, and the regulatory authority. The investigator should immediately inform the EC and the sponsor and provide relevant reasons.

Reasons for early termination or suspension of the study may include:

- Identified unexpected, significant, or unacceptable risk to subjects;
- Available efficacy results in support of early termination of the study;
- Low compliance to protocol requirements;

Once the above-mentioned drug safety, protocol compliance, data quality and other issues that led to suspension of the study have been resolved, the study can be resumed with approvals from the sponsor, EC or regulatory authority.

4.7. Definition of End of Study

End of Study: End of study is defined as the termination of the study after the death of 70% of the subjects at Stage 2 of the study or upon the decision of the sponsor.

5. study treatment

5.1. Overview of the investigational drug

5.1.1. Acquisition of the drug

All study drugs are supplied by the sponsor. They are uniformly packed and have been inspected against their specifications (see the corresponding certificates of analysis (CoAs)). The management, dispensing and recovery of clinical medications in this study are in the charge of a designated person. The investigator must ensure that all investigational medications are only used for the subjects participating in this clinical trial, and their dosage and administration should follow the study protocol. The remaining drug should be returned to the sponsor. No investigational drug for this study is allowed to be used for other purpose.

The medical monitor should be responsible for monitoring the supply, use and storage of the clinical investigational medications and the handling process of the remaining drugs.

5.1.2. Pharmaceutical information

Investigational Drug: SHR-1701 Injection

Manufacturer: Shanghai Hengrui Pharmaceuticals Co., Ltd.

Dosage form: injection

Strength: the strength of the product provisionally proposed for this clinical trial is 6 ml: 0.3 g

Batch Number: Refer to the CoA

Route of administration: intravenous instillation

Storage conditions: this product should be stored and transported in its original package at 2°C ~ 8°C and protected from light. Do not freeze or shake

Study Drug: Paclitaxel for Injection (Albumin Bound)

Manufacturer: Jiangsu Hengrui Pharmaceuticals Co., Ltd.

Dosage Form: Lyophilized powder

Strength:100mg in glass bottle, 1 bottle/box.

Batch Number: Refer to the CoA

Route of administration: intravenous instillation

Storage conditions: Store at 20-30°C away from light

Study Drug: Gemcitabine

Manufacturer: Jiangsu Hansoh Pharmaceutical Co., Ltd.

Dosage Form: Lyophilized powder

Strength: 1.0g

Batch Number: Refer to the CoA

Route of administration: intravenous instillation

Storage conditions: Store sealed in dry place

5.1.3. Preservation of drugs

The management, dispensing and recovery of clinical medications in this study are in the charge of a designated person. The investigator must ensure that all investigational medications are only

used for the subjects participating in this clinical trial, and their dosage and administration should follow the study protocol. The remaining drug should be returned to the sponsor. It is not allowed to transfer the study drugs to any one other than those participating in this clinical trial.

The study drugs should be stored under the storage conditions specified under 5.1.2; they should be packed in their original containers and properly labeled. Where the storage conditions indicated on drug label differ from the conditions specified in other documents (e.g., IB), the conditions on the label should be followed.

The investigator or his/her authorized person (e.g., a pharmacist) should see to that all study drugs are stored in an eligible, access-controlled, secure area in accordance with applicable regulatory requirements.

The study site needs to record the daily maximum and minimum temperature of all storage locations (e.g., freezer, refrigerator, or storage room). The recording of storage temperature should start from the receiving of the study drugs to the end of last visit of last subject (LVLS). Even study sites equipped with continuous monitoring system should keep a temperature log to record the correct storage temperature. The investigators should regularly check the operation state of temperature monitoring devices and storage devices.

Any deviation from the product labeling conditions should be promptly reported upon discovery. The study site should take active measures to return the product under the labeled storage conditions as soon as possible, and report any storage temperature deviations and the actions taken correspondingly to the sponsor.

Study drugs affected by storage temperature deviation should be quarantined and should not be used until the sponsor determines that the temperature deviations are not protocol deviations. The use of study drugs affected by temperature deviation without prior permission from the sponsor will be deemed protocol deviation. The sponsor will provide the site with specific steps to report temperature excursions.

5.1.4. Mode of drug administration

It is recommended that SHR-1701 be administered at 30 mg/kg or 20 mg/kg (depending on the specific stage of the phase Ib study) by intravenous instillation in 30-60 minutes with infusion pump. Intravenous bolus or rapid bolus are not allowed. At the end of infusion, the infusion pipeline should be flushed with sufficient amount of normal saline or 5% glucose solution (in accordance with the standard medical practice of the study site). For the mixing procedures and (preparation) concentrations of drug solutions and the mode of administration of the drug solutions, refer to Pharmacy Manual.

Gemcitabine, administer at 1000 mg/m^2 (for the drug's initial dose, subsequent dose modification, refer to 5.2.3), q3w, i.v., on D1 and D8 of each cycle; for more details, refer to the IFU of the drug.

Albumin-bound paclitaxel, administer at 125mg/m^2 (for the drug's initial dose, subsequent dose modification, refer to 5.2.3), q3w, i.v., on D1 and D8 of each cycle; for more details, refer to the IFU of the drug.

The study medications are administered on D1 of each of the 3-week cycles. Dosing sequence: first, SHR-1701 is administered intravenously; after an interval of at least 30min, albumin-bound paclitaxel is administered; finally, gemcitabine is administered.

The administration of SHR-1701 and the chemotherapy regimen should be harmonized, i.e., if the administration of SHR-1701 is delayed due to SHR-1701 related AE, the administration of the chemotherpay regimen should also be postponed (if the dosing of SHR-1701 needs to be

suspended for a long time and the investigator believes the harmonized suspension of chemotherapy is bad for the patient, it is acceptable to continue treatment with the AG regimen alone); vice versa, if the administration of AG regimen postponed due to the toxicity of chemotherpay, the dosing of SHR-1701 should also be postponed (if, in the opinion of the investigator, it is detrimental to suspend the SHR-1701 treatment in a patient whose chemotherapy is suspended; the subject can be treated with SHR-1701 monotherapy as scheduled); if the staggering of the administration time of the chemotherapy and SHR-1701 is inconsistent with relevant requirements, then the investigator should make adjustment of the dose regimen according to the latest dosing time (of chemotherapy of SHR-1701) in order to harmonize the dosing time of the chemotherapy and SHR-1701 (on the premise that the toxicity has relieved to such an extent that all drugs can be administered together).

During treatment with the AG regimen, if the investigator determines that the observed toxicity is caused by one component drug of the regimen, he/she can reduce the dose of the drug; if the cause of the toxicity is indeterminate, then the doses of all drugs should be reduced. If the dosing of a drug (albumin-bound paclitaxel or gemcitabine) needs to be discontinued due to AE(s), the use of another component drug of the chemotherapy can be continued. It is up to the investigator to decide the necessity to administer the chemotherapy and adjust the dosage of the chemotherapy depending on the subject's actual situation.

If a subject discontinues his/her chemotherapy, the application of SHR-1701 monotherapy can be continued on the premise that the subject has no PD or meets the criteria for remain on treatment after PD.

Postpone of the administration of a drug to 3 days after the scheduled dosing date will be deemed as a dose delay. Subsequent dosing time should be calculated on the basis of the actual date of the previous dose. The subjects will be administered the study drugs until the protocol specified study endpoints have been achieved.

5.2. Dose modification of study drugs

5.2.1. Dose modification of SHR-1701

In principle, it is not allowed to reduce the dose level of SHR-1701.

5.2.2. Discontinuation and delay of SHR-1701 dose

It is not allowed to increase or decrease the dose level of SHR-1701; if treatment with SHR-1701 is temporarily suspended because of toxicity, the treatment can be resumed on the subject at the original dose level when the toxicity has been relieved.

During the study, the dosing in a subject should be delayed in case any of the following occurs:

- Non-skin drug-related AEs of Grade ≥2 (excluding Grade 2 lassitude);
- Drug-related creatinine, AST/ALT or TBIL abnormality of Grade 2;
- Drug-related skin reaction of Grade 3;
- Drug-related lab abnormalities of Grade 3 (excluding lymphocyte decreased, asymptomatic blood amylase or lipase abnormality):
  - Grade 3 lymphocyte decrease that requires no dose delay;
  - Blood amylase or lipase abnormality of Grade 3; however, dose delay is unnecessary if the subject is asymptomatic or shows no pancreatitis related symptom.

Study treatment can be resumed when drug-related toxicity has regressed to grade ≤1 or baseline

level. It is recommended that safety assessment/examination be conducted once weekly or at higher frequency in subjects whose treatment is discontinued until the study treatment is resumed. If the toxicity fails to regress to baseline level or Grade 0-1 after 12 weeks of withdrawal, permanent treatment withdrawal should be considered.

Resumption of treatment could be considered for subjects who are recovering from the toxic effect after 12 weeks of dose discontinuation, provided that the investigator believes the subject will benefit from the resumption. However, the decision to resume treatment needs to be made by the investigator and the sponsor together after discussion.

5.2.3. Criteria for dose modification of AG regimen

The dose of chemotherapy should be calculated according to subject's baseline body weight. If, during the study, the subject's body weight change from baseline value is ≥10%, the dose level of chemotherapy needs to be modified; on the contrary, if the subject's body weight change from baseline value is <10%, then no dose modification is required. If necessary, modification should be made (refer to 5.2.3.1 and 5.2.3.2) for the doses on D1 and D8 of each cycle depending on the subject's situation (clinical symptoms, physical signs, and laboratory investigation results, etc.). In this study, the doses of chemotherapy drugs can be adjusted to any of the 3 predefined levels (Table 13). If a subject has dose reduction because of toxic reaction, then no dose reduction is allowed for him/her in subsequent treatment; subjects who cannot tolerate the treatment after two dose reduction should be permanently withdrawn from treatment with the corresponding chemotherapy drug. In case of the simultaneous occurrence of more than one toxicities of different grade in different categories, dose modification should be done according to the toxicity of highest grade. Dose regimen should be determined according to subject's body surface area, Body Surface Area (m^2) =0.0061×Body Height (cm) + 0.0128×Body Weight (kg) - 0.1529.

Table 13 Dose levels of AG regimen available for dose modification

Dose level	Gemcitabine.	Albumin paclitaxel
1	1000 mg/m^2 D1, D8 q3w,	125 mg/m^2 D1, D8 q3w,
-1	800 mg/m^2 D1, D8 q3w,	100 mg/m^2 D1, D8 q3w,
-2	600 mg/m^2 D1, D8 q3w,	75 mg/m^2 D1, D8 q3w,

5.2.3.1. Rules for dose modification of chemotherapy on D1 of a cycle

Table 14 Rules for dose modification of the AG regimen on D1 of each cycle

**Hematologic toxicity**			
**Absolute neutrophil count (ANC)**		**Platelet count (PLT)**	
≥ 1.5 × 10^9/L	&	≥ 100 × 10^9/L	Initial therapeutic dose or the same as previous dose
< 1.5 × 10^9/L	and/or	< 100 × 10^9/L	One week of dose delay, until ANC ≥ 1.5 × 10^9/L and ≥ 100 × 10^9/L
**Non-hematological toxicities**			
Grade ≤2 (except for Grade 2 skin toxicity*)			Initial therapeutic dose or the same as previous dose
Grade 3 (except for Grade 3 catarrh or diarrhea%)			Dose reduction of albumin-bound paclitaxel and/or gemcitabine (depending on relatedness) to the next lower level#
Level 4			Permanent discontinuation (with the exception of medication for treatment of pulmonary embolism&)

*Skin toxicity of Grade 2-3: Dose of the AG regimen should be adjusted to the next lower level; if the toxicity recurs or does not resolve, then permanent withdrawal should be considered;

% Catarrh or diarrhea of Grade 3: the AG regimen should be suspended until the toxicity regresses to grade 1 or normal; the treatment in the next cycle should be down regulated to the next lower level;

Peripheral neuropathy of grade ≥ 3: The administration of albumin-bound paclitaxel should be suspended until the toxicity regresses to grade 1 or normal, then the next cycle of treatment will be carried out at the next lower level; if albumin-bound paclitaxel has been suspended for the toxicity for more than 21 days, then permanent withdrawal should be considered; the administration of gemcitabine is not affected by this;

& Pulmonary embolism: Symptomatic treatment with low molecular weight heparin can be administered to asymptomatic or mild symptomatic pulmonary embolism subjects without discontinuation of the chemotherapy; if moderate or severe symptomatic pulmonary embolism occurs, permanent discontinuation of chemotherapy should be considered.

5.2.3.2. Rules for dose modification of chemotherapy on D8 of a cycle

The occurrence of treatment-related toxicity in the next cycle will affect the administration of chemotherapy on D8; for detailed rules for the modification, refer to Table 15.

Table 15 Rules for dose modification of the AG regimen on D8 of each cycle

**Hematologic toxicity**			
**Absolute neutrophil count (ANC)**		**Platelet count (PLT)**	
$\geq 1.0 \times 10^9$/L	&	$\geq 75 \times 10^9$/L	The same as previous dose
$\geq 0.5 \times 10^9$/L, $< 1.0 \times 10^9$/L	and/or	$\geq 50 \times 10^9$/L, $< 75 \times 10^9$/L	AG regimen, at the next lower dose level
$< 0.5 \times 10^9$/L	and/or	$< 50 \times 10^9$/L	Temporary suspension of chemotherapy
Grade 3-4 febrile neutropenia$			The AG regimen should be suspended until the toxicity regresses to grade 1 or normal; the treatment in the next cycle should be down regulated to the next lower level;
Recurrence of Grade 3-4 febrile neutropenia			Downregulation of dose level of AG regimen again
**Non-hematological toxicities**			
Grade≤2, and nausea/vomiting or alopecia of Grade 3			The same as previous dose
Grade 3, except for nausea/vomiting or alopecia			Albumin-bound paclitaxel and/or gemcitabine (depending on relatedness) should be suspended until the non-hematotoxicity is reversed to Grade 1 or normal, then the next cycle of treatment will be carried out at the next lower level
Grade 4, except for catarrh and diarrhea@			The treatment should be suspended; subsequent treatment should be determined by the investigator after a comprehensive evaluation of the subject's condition.

$ If neutrophil decrease lasts for more than 21 days without remission after symptomatic treatment, permanent discontinuation of chemotherapy should be considered.

@ Permanent discontinuation of chemotherpay should be considered for subjects who experience Grade 4 catarrh or diarrhea,

5.3. Duration of treatment with the investigational drug

Subjects should continually receive study treatment until PD, intolerable toxicity, their active

discontinuation of study treatment or withdrawal from the study, or their passive withdrawal from the study by the investigator. In consideration of the cumulative toxicity of long-course chemotherapy, it is recommended that the entire chemotherapy lasts for NMT 6 cycles.

One mechanism of the antitumor action of SHR-1701 lies in the drug's suppression of the PD1/PD-L1 pathway; in light of applicable experience with similar drugs, some subjects after receiving the immunotherapy drug may experience tumor pseudoprogression. Subjects who have the first radiologically-confirmed PD may be eligible for remain on treatment with SHR-1701 alone or in combination with chemotherpay if they meet the criteria in 4.5 Criteria for remain on treatment after disease progression.

5.4. Management, dispensing, recovery and destruction of drugs

The management, dispensing and recovery of clinical medications in this study are in the charge of a designated person. The investigator must ensure that all investigational medications are only used for the subjects participating in this clinical trial, and their dosage and administration should follow the requirements in 5.1.4. Remaining drugs or expired drugs should be returned to the sponsor or destroyed at the study site according to specified SOPs. None of the drugs shall be transferred to any one other than participants of the clinical study.

At drug dispensing, a drug receipt in duplicate should be signed by the dispenser and the receiver; one will be kept at the study site and the other will be kept by the sponsor. For recovery of the remaining drugs and empty boxes, both parties should sign the Drug Recovery Form. The dispersing and recovery of each drug should be recorded on a special sheet in a timely manner.

The medical monitor should be responsible for monitoring the supply, use and storage of the clinical investigational medications and the handling process of the remaining drugs.

- **Destruction of investigational drug**

The sponsor or its authorized personnel should destroy the investigational drug and keep a record of all destructions.

5.5. Concomitant medications and concomitant therapies

Concomitant medications/concomitant therapies are medications/therapies administered at the investigator's discretion and in consideration of the subject's interest.

All concomitant medications, blood products, non-drug interventions (e.g., aspiration) administered to the subjects in the period from 30 days prior to the first dose of study medications to the end of the safety follow-up visits should be recorded in CRF in strict accordance with GCP.

Medications or vaccines specifically prohibited in the study protocol are not allowed throughout the study. If a subject develops a comorbidity that necessitates the use of prohibited medications, and may require discontinuation of study drug treatment or receipt of prohibited medications, the investigator will need to discuss with the sponsor, and the decision for the subject to continue study treatment or receive prohibited medications is ultimately at the discretion of the investigator, the sponsor, and the subject.

5.5.1. Other antitumor therapies or investigational drugs

During the study treatment of subjects, no antitumor therapies other than those specified in the study protocol are allowed, including modern TCM preparations that have been granted marketing authorizations(for more details, refer to Annex 5 Prohibited TCM Preparations during the Study), and immunomodulators (including but not limited to: interferon, interleukin-2, thymic peptide, etc.).

The subjects are not allowed to take part in any clinical trials of other drugs/medical devices.

The subject are also not allowed to receive other systemic antitumor therapies, such as chemotherapy, molecular targeted therapy, hormone therapy, immunotherapy, biological therapy and radiotherapy, etc.

The subjects are allowed to receive diphosphonate for the treatment of bone metastasis. It is acceptable to use small area palliative radiotherapy (the coverage area of radiotherapy shall be NMT 5% of the bone marrow region; for bone marrow content in human body, refer to Annex 6 bone and bone marrow content (%) in human body) in subjects whose pain in bone metastasis lesion cannot be effectively controlled by systemic treatment or local analgesics.

It is acceptable to administered palliative therapy on local lesions that have significant symptoms, such as bone pain lesions; local radiotherapy or surgery may also be considered, provided the following criteria are met. Moreover, it is recommended that the sponsor be consulted prior to the start of the local palliative therapy.

1) The investigator must determine whether those subjects who need local therapy due to deterioration of symptoms during the study have PD or not;
2) Criteria for patients with PD to remain on treatment after disease progression;
3) The lesion for local therapy should not be the target lesion.

5.5.2. Treatment of diarrhea and vomiting

Primary prevention of diarrhea, nausea and vomiting is not allowed prior to the first study treatment. It is up to the investigator to determine the acceptability of primary prevention in subsequent cycles and chose the prophylactic drug(s). A precondition is that the prophylactic drugs are not listed in previous sections as prohibited medications, and free of known or predictable drug-drug interactions during the treatment.

5.5.3. Vaccine

No live attenuated vaccine shall be used in the period from 4 weeks prior the first dose to 60 days after the end of the study treatment. It is advised that subjects who plan to receive live attenuated vaccine during the study be excluded from the study.

It is acceptable to use vaccines, such as, pneumonia vaccine and influenza vaccine, to prevent infectious diseases. However, such vaccines must be inactivated vaccines. The investigator must consult the sponsor before the use of other vaccines.

5.5.4. Immunomodulators and corticosteroids

Subjects are not allowed to receive immunosuppressant therapy concurrently with the study therapy (unless the immunosuppressant therapy is for the treatment of drug-related AEs).

Concurrent treatment with immunostimulants (except for the management of drug-related adverse events) is not permitted.

Subjects are also not allowed to use systemic corticosteroids for a prolonged period. It is acceptable to use corticosteroids in accordance with their IFUs for NMT 1 week as preventive medication of allergy to chemotherapy or contrast agents. Systemic treatment with corticosteroids to individual subjects after discussion with the sponsor is permitted. It is acceptable to use corticosteroids for a short period (NMT 3 weeks) to treat auto-immune diseases (e.g., delayed hypersensitivity reaction arising from contact with allergens).

However, emergency use, topical application, inhalation by spray, eye drops or local injection of corticosteroids are permitted. Systemic use of corticosteroids (≤ 10 mg/day prednisone or

equivalent) at physiologic replacement doses (e.g., adrenal replacement steroid dose) is permitted.

5.5.5. Hematopoietic growth factors and blood transfusion

It is not permitted to use hematopoietic stimulating factors such as granulocyte colony stimulating factor (GCSF), erythropoiesis stimulating factor (ESF), and thrombopoietin and to transfuse blood or blood products (e.g., albumin) as primary prevention measures before the study treatment; however, it is acceptable to use the afore-mentioned for the treatment of AEs.

5.5.6. Anti-inflammatory therapy

It is permitted to use anti-inflammatory or narcotic analgesics if they have no known or predictable drug-drug interaction with the study treatment and are not listed as prohibited medications in the protocol.

5.5.7. Surgery

Any surgery performed during the study should have its theoretical basis and necessity. The time interval between the surgery and the administration of the investigational drug should not interfere with the healing of wounds and the identification of bleeding of undetermined origin. It is advised that the administration of the investigational drug be discontinued within 1 week prior to the surgery. The postoperative resumption of the study treatment depends on the clinical evaluation results of wound healing and post-operative recovery.

5.5.8. Supportive care

Palliative and supportive care for disease-related symptoms will depend on the investigator's judgment and relevant guidelines (e.g., ASCO guidelines).

During the treatment period, the subjects should be administered the best supportive treatment.

It is inadvisable to include subjects with controlled tumor-related pain. For subjects who need analgesics, stable pain-relieving scheme should be established prior to their enrollment; subjects with symptomatic lesions (e.g., metastasis to bone or invading nerves) that are suitable for palliative radiotherapy should be completely treated within at least 4 weeks prior to their enrollment; topical/local treatment of asymptomatic metastases (e.g., epidural metastases that have no spinal cord compression manifestation) should be considered before the start of the study treatment if appropriate lest their further growth may lead to functional disorders or intractable pain.

It is not permitted to use RANKL inhibitors (Nuclear Factor κ B Receptor Activator Ligand, such as denosumab). It is inadvisable to include subjects who have uncontrolled hypercalcaemia (calcium ion: >1.5 mmol/L; or, calcium: >12 mg/dl; or, corrected serum calcium:>ULN), or symptomatic hypercalcemic subjects who need persistent treatment with diphosphonate therapy or denosumab therapy. It is acceptable to include patients who are on diphosphonate therapy for prevention of skeletal events because of their history of clinically insignificant hypercalcinemia; subjects who are on treatment with denosumab prior to enrollment have to agree to discontinue their denosumab therapy and shift to diphosphonate therapy after their enrollment.

Existing hormone replacement therapy is permitted. For example, subjects who are currently receiving gonadotropin-releasing hormone (GnRH agonist) can enter this study as long as they have tolerated GnRH agonists well for at least 3 months prior to enrollment. Likewise, patients with a history of autoimmune-mediated hypothyroidism who are receiving a stable dose of thyroid replacement hormone can be enrolled in this study; Type 1 diabetes patients who are receiving a stable insulin regimen and whose blood glucose is controlled can be enrolled in this study.

5.6. Recommended symptomatic treatment of common AEs

5.6.1. Rules for Safety Management of Immuno-oncology Agents

For their special severity and duration characteristics, immuno-oncology (I-O) agent induced AEs are somewhat different from other antitumor agent induced ones. SHR-1701 belongs to this type of agents. Therefore, it is necessary to identify and treat its I-O agent induced AEs and to reduce the occurrence of serious toxicity events. Investigators can refer to relevant guidelines on the toxicity management of immunotherapies to assist their evaluation and treatment of AEs in the following systems: gastrointestinal tract, kidneys, lungs, liver, endocrine, skin and nerve. Below are some recommendations on the treatment of immune-related toxicities.

- Immune-related skin toxicity

Patients with Grade 1 ~ 2 skin AEs are eligible for remain on the study treatment (for at least 1 week). Subjects with pruritus cutaneous can be treated with topical skin moisturizing cream, oral antihistamines and/or topical glucocorticoids ointments. The study treatment can be resumed when the skin AEs have resolved to below Grade 1.

When a subject experienced Grade 3 skin AEs, he/she should discontinue the study treatment and start treatment with topical skin moisturizing cream, oral antihistamines and large dose glucocorticoids ointments.

Subjects who experience Grade 4 skin AEs should be permanently withdrawn from the study treatment and immediately hospitalized for treatment; during their hospitalization, dermatologist should be consulted and glucocorticoid (methylprednisolone 1 ~ 2 mg/kg) should be administered by intravenous infusion. The dosage of the glucocorticoid should be reduced gradually depending on the conditions of the AEs.

- Immune-related pneumonitis

In the clinical study of SHR-1701, patients will be placed under intensive monitoring for signs and symptoms of immune-related pneumonitis such as coughing, chest distress, etc.

Patients who experience Grade 2 pneumonia should discontinue their study treatment and, after excluding the possibility of infectious pneumonitis, receive oral methylprednisolone at 1 ~ 2 mg/kg for treatment.

Patients who experience Grade 3 ~ Grade 4 pneumonia should be permanently withdrawn from the study treatment. Moreover, they should be intravenously administered methylprednisolone at high dose (2 ~ 4 mg/kg). If their conditions aggravated during the hormone therapy, infliximab, mycophenolate-mofetil (MMF) or cyclophosphoramide may be added for treatment and the dose levels of these therapies should be reduced after 4-6 weeks of treatment.

- Immune-related gastrointestinal toxicity

In the clinical study of SHR-1701, patients will be placed under intensive monitoring for signs and symptoms of immune-related enteritis, such as abdominal pain, diarrhea and hematochezia, etc.

Patients with non-serious diarrhea (Grade 1) can continue their study treatment and should be administered antipropulsives (e.g., loperamide).

Patient with Grade 2 diarrhea should discontinue their study treatment and, depending on the severity of their diarrhea and other concomitant symptoms, start to receive glucocorticoid therapy(budesonide or an oral glucocorticoid, 1 mg/kg). If the patients' conditions have not been improved after 3-5 days of treatment, they should undergo colonoscopy.

Patients who experience serious diarrhea (Grade 3 ~ Grade 4) should be permanently withdrawn

from the study treatment and intravenously administered methylprednisolone at 2 mg/kg for treatment.

- Immune-related hepatotoxicity

In the clinical study of SHR-1701, patients will be placed under intensive monitoring for signs and symptoms of immune-related hepatitis, such as liver discomfort, abnormal increase of transaminases, etc. Patients with hepatotoxicity of grade ≥ 2 should be put on high-dose hormone therapy. Patients with Grade 2 immune-related hepatitis should discontinue their SHR-1701 temporarily and receive treatment; patients with Grade 3 or Grade 4 immune-related hepatitis should be permanently withdrawn from SHR-1701 therapy.

Patients who experience Grade 2 hepatitis AE should be temporarily withdrawn from the study treatment and closely monitored for their AST and ALT levels (1 ~ 2 tests per week). If their transaminases levels show no signs of improvement more than 1 week after discontinuation, they should be treated with methylprednisolone (0.5 ~ 1 mg/kg) and their AST, ALT and bilirubin levels should be closely monitored; the dose level of their methylprednisolone therapy should be gradually reduced after several weeks.

Patients who have Grade 3 hepatitis AE should discontinue their study treatment and start methylprednisolone therapy (1 ~ 2mg/kg); if their AE have not been improved after 2-3 days of treatment, mycophenolate-mofetil (MMF) may be added.

Patients who experience Grade 4 hepatitis AE should be permanently withdrawn from the study treatment, hospitalized, and intravenously administered methylprednisolone at 2 mg/kg for treatment. If the AE fails to resolve after 2-3 days of treatment, mycophenolate-mofetil (MMF) therapy may be added. If a patient fails two immunosuppressants, hepatologists should be consulted.

- Immune-related abnormal thyroid function

Abnormal thyroid function can occur at any time period of the study. Therefore, patients will be regularly tested for their thyroid function during the SHR-1701 study and closely monitored for clinical symptoms of abnormal thyroid function. Patients who develop immune-related hyperthyrosis should be treated with high-dose cortisone/prednisone. Patients with hypothyroidism AE should be treated with hormone replacement therapy but should not be treated with glucocorticoids.

In the clinical study of SHR-1701, patients will be placed under intensive monitoring for signs and symptoms of immune-related abnormal thyroid function. Patients with AEs of grade ≥ 3 should receive high-dose hormone therapy; patients with Grade 4 AEs should be permanently withdrawn from SHR-1701 therapy.

- Immune-related nephritis and renal failure

Patients with nephritis AE should be examined to exclude renal failure caused by other reasons. Afterwards, the study treatment should be interrupted or permanently discontinued depending on the severity of renal insufficiency; moreover, other nephrotoxic agents should also be discontinued and methylprednisolone therapy (1 ~ 2 mg/kg) should be started.

- Rheumatological toxicity

Patient with mild arthralgia AE can be treated with non-steroidal anti-inflammatory drug (NSAIDs); if the symptom is not relieved after the treatment, low-dose hormone therapy (10 ~ 20 mg prednisone) may be considered. Patients with serious polyarthritis are advised to go to department of rheumatology or consult rheumatologist; 1 mg/kg prednisone therapy should be started. Sometimes it may be necessary to use infliximab or other anti-TNFα drugs to treat their

arthritis.

- Immune-related cardiotoxicity

In the clinical study of SHR-1701, patients will be placed under intensive monitoring for signs and symptoms of immune-related myocarditis, such as dyspnea, palpitations, chest pain and precordial discomfort, etc. It is up to the investigator to consider the need for ECG, cardiac enzyme spectrum and cardiac ultrasound examinations. If a patient is diagnosed with immune-related myocarditis, he/she should be treated with large dose methylprednisolone (1 ~ 2 mg/kg) and the treatment with the investigational drug should be permanently discontinued.

- Principles for the treatment of other immune-related adverse reactions

In principle, SHR-1701 therapy should be suspended depending on the severity of immune-related adverse reactions; when the AR is reversed to grade ≤1 or baseline level, resumption of SHR-1701 therapy may be considered. In patients who experience serious Grade 3 or life-threatening Grade 4 ARs, SHR-1701 therapy should be discontinued permanently.

In this study, it is recommended that patients who need glucocorticoid therapy be closely monitored for glucocorticoid induced ARs (the severity of which is generally in direct proportion to dose level and duration) and, if such ARs are identified, preventive and therapeutic measures should be taken in accordance with the study site's medical practices and applicable guidelines.Table 16 The recommendations listed in are for reference only (covering but not limited to the following conditions)

Table 16 Glucocorticoid induced ARs and recommended preventive and therapeutic measures

Glucocorticoid induced adverse reaction	Preventive and therapeutic measures
GI ulceration and/or bleeding	Gastric mucosa protectants, H2 receptor antagonists, proton pump inhibitors, etc.
Electrolyte imbalance (e.g.:hypopotassemia, hypernatremia, etc.)	Monitoring of electrolyte, active correction of electrolyte imbalance (e.g.: low sodium high potassium diet and, if necessary, oral or intravenous supplementation of potassium, etc.)
Hyperglycemia, hypertension, hyperlipidemia, etc.	Monitoring of blood sugar, blood pressure, lipids and, if necessary, administration of hypoglycemics, antihypertensives, lipid lowering drugs
Osteoporosis, spontaneous fracture or osteonecrosis	Supplementing active vitamin D3 and calcium and, if necessary, administration of anti-osteoporotics
Bacterial, viral and fungal infections	Active anti-infection treatment
Congestive heart failure, water-sodium retention	Administration of appropriate diuretics that has minimal impact on electrolyte balance
Psychiatric symptoms, such as, anxiety, excitation, euphoria or depression, insomnia, etc.	Administration of corresponding anxiolytic, antidepressant and hypnotic agents under the direction of psychiatrists
Muscular weakness, muscle atrophy, slow wound healing	high protein diet

(Refer to "DOH Principles for Clinical Application of Glucocorticoids" and "Consensus on the Drug Treatment of Peritumoral Brain Edema (The 1st Edition)")

5.6.2. Management of infusion reactions

Throughout the study process, the investigators need to pay close attention to any and all potential infusion reactions and/or allergic reactions, especially acute immune-mediated adverse reactions (including cytokine storm).

SHR-1701 as a full humanized monoclonal antibody has little potential for triggering infusion reactions or allergic reactions. Therefore, generally no prophylactic medication is required before the drip infusion of SHR-1701. Relevant information revealed that allergic reaction/allergy events are most likely to occur within 24h of the infusion. If such allergic reaction/allergy events do occur, they should be treated by slowing down or discontinuing the infusion, administrating clinical supportive treatment, and the application of prophylactic medication prior to subsequent dosing. The potential allergic reaction manifestations include: pyrexia, cold intolerance, chills, headache, skin rash, arthralgia, hypotension or hypertension, or bronchial spasm.

For the treatment of allergic reactions, the study site's medical practices and guidelines should be followed. Below are some recommended therapies for the treatment of infusion reactions (Table 17) for the reference of the investigators.

Table 17 Recommended treatment for SHR-1701 infusion reaction

CTCAE Grade	Clinical symptoms	Recommended treatment	SHR-1701 therapy
Level 1	Mild transient reaction	Observe at the bedside, and monitor closely until recovery. Prophylactic medication prior to infusion is recommended: diphenhydramine 50 mg, or equivalent and/or acetaminophen 325-1000 mg at least 30 min before the administration of SHR-1701.	Continuing
Level 2	Moderate reaction: It requires treatment or dose suspension and can resolve rapidly after symptomatic treatment (e.g., antihistamines, nonsteroidal anti-inflammatory drugs, anesthetics, bronchodilators, intravenous infusion)	Normal saline intravenous infusion, diphenhydramine 50 mg IV or equivalent and/or acetaminophen 325-1000 mg; bedside observation and closely monitoring until the resolution of reaction<br>Corticosteroids or bronchodilators may be considered if clinically indicated;<br>The amount of the investigational drug infused should be recorded in the original medical records;<br>Prophylactic medication prior to infusion is recommended: diphenhydramine 50 mg, or equivalent and/or acetaminophen 325-1000 mg at least 30 min before the administration of SHR-1701. Cortisols (equivalent to a hydrocortisone dose of 25 mg), if necessary	Suspend. 50% of the initial infusion rate will be used when the drug is re-administered after resolution of symptoms. The infusion rate can be adjusted back to the original value if no complication was observed within 30 min.<br>Monitor closely. The infusion should be discontinued if the symptoms recur.
Grade ≥ 3	Grade 3: serious reaction, with no rapid response to treatment and/or drug interruption; or recurrence of symptoms after response; with sequelae requiring hospitalization.<br>Grade 4: Life-threatening.	The drip infusion of SHR-1701 should be stopped immediately;<br>The intravenous infusion of normal saline should be started.<br>Bronchodilators are recommended: 0.2-1 mg of 1:1000 epinephrine solution, s.c, or 0.1-0.25 mg of 1:10000 epinephrine solution, i.v. If necessary, 50 mg of diphenhydramine and 100 mg methylprednisone (or equivalent dose) can be administered intravenously.<br>The study site's medical practices and guidelines for the treatment of anaphylactic reactions should be followed. Observe at the bedside, and monitor closely until recovery.	Termination of medications

5.6.3. Rules for treatment of common AEs of the AG chemotherapy regimen

The AG chemotherapy regimen has been approved by CFDA and DOH for the treatment of advanced/metastatic PC patients; abundant experience has been accumulated during the clinical application of the regimen. In light of this, it is stipulated in the study protocol that the treatment of AG chemotherapy regimen related ARs can be done in line with clinical practices; below are some advices for the reference of the investigators.

1. Neuropathies: Neuropathies caused by albumin-bound paclitaxel mainly include sensory neuropathies and peripheral neuropathies; for the detailed treatment, refer to 5.2.3.1;
2. Allergic reactions: mainly manifested as: pruritus cutaneous, skin rash, etc.; in case of the occurrence of Grade 1/Grade 2 allergic reactions, the affected subjects should be administered dexamethasone (20 mg, i.v.,) and/or diphenhydramine (50 mg, i.v.) prophylactically 30 minutes prior to the next dose of the chemotherapy.
3. Pyrexia and infections: common infections include candida infection, injection site infection, respiratory tract infection and pneumonia, etc. In previous studies, septicemia occurred in some subjects (<1%); if a subject manifests pyrexia (≥38.5°C), then, regardless of the ANC results, he/she should contact the investigator immediately and start to take oral antibiotics (ciprofloxacin 500mg, b.i.d., or levofloxacin 500mg, q.d., if the subject has a history of allergy to fluoroquinolones antibacterial agents, then oral amoxicillin/caratclavulanate potassium 500mg, b.i.d., or t.i.d) is recommended. It is advised that every subject has made appropriate antibiotics available for timely use whenever pyrexia occurs.
4. Respiratory system: main manifestations include: coughing, dyspnea, interstitial pneumonitis, pleural effusion, pulmonary embolism, etc.; the study medication should be discontinued immediately in patients diagnosed with interstitial pneumonitis, and the patients should be administered corresponding treatment after clinical evaluation.
5. Skin reactions: mainly include: generalized skin rash, nail changes, nail pigmentation and itching; for detailed treatment, refer to 5.2.3.1;
6. GI reactions: mainly include: nausea/vomiting, diarrhea and catarrh; for detailed treatment, refer to 5.2.3.1;

6. Study Procedures

6.1. Screening Phase

The screening period starts from the signing of ICF and ends at the start of the first dose of the investigational drug, or the failure of screening.

Subjects have to sign the ICF before proceeding to the screening procedures of the study. If a subject has taken laboratory tests and imaging evaluations in the specified time windows prior to his/her signing of the ICF, it is acceptable to use relevant data from such laboratory tests and imaging evaluations as the result data of the corresponding screening laboratory tests and imaging evaluations.

Unless otherwise specified, the following screening should be completed within 28 days prior to the start of the investigational drug therapy.

- Obtaining signed and dated ICFs from subjects.
- Collecting demographic information: gender, date of birth and ethnicity, etc.

- Collecting AEs: Recording the occurrence of AEs after the signing of ICF.
- Tumor diagnosis: Pathologically-confirmed diagnosis date, pathological classification, pathological staging (TNM), clinical staging and primary lesion and metastases sites.
- History of tumor treatment:
  - ✓ Surgical history of tumor: name of surgical procedures, date of surgery;
  - ✓ History of radiotherapy: irradiation site, dose, start and end dates;
  - ✓ History of neoadjuvant chemotherapy: chemotherapy regimen, cycle, start and end time;
  - ✓ History of adjuvant chemotherapy: chemotherapy regimen, cycle, start and end time;
  - ✓ History of concomitant diseases, history of previous medications, drug allergic history, etc.;
- Virology tests: HBsAg, HBsAb, HBeAg, HBeAb, HBcAb and HBV DNA quantitative assay (if “test of (five) hepatitis B serologic markers” reveal active HBV infection [HBsAg+] or history of previous HBV infection, HBV DNA quantitative assay has to be performed), HCV-Ab (if HCV-Ab test result is positive, then HCV-RNA quantitative assay has to be performed) and HIV-Ab.
- Imaging examination: The examination should be done by chest, abdominal, and pelvic CT or MRI. Subjects with suspected or diagnosed metastasis to brain should receive brain MRI (or CT if MRI is impossible). Bone scan is to be done only when clinically indicated; screening bone scan has to be scan done within 42 days prior to the first dose. In the screening period, tumor assessment can be done with imaging examination results obtained within 4 weeks prior to the first dose of the study medications even before the subject’s signing of ICF and the results are usable so long as they are up to the requirements.
- Collection of tumor tissue samples: Subjects should provide their tumor tissue samples. Requirements: neutral formalin-fixed, paraffin-embedded unstained tumor sections. It is recommended that 5-10 sections of 3~5 μm thick (or more samples if the tissue samples are small or are aspirate samples) be collected and submitted to the study’s designated central laboratory for biomarkers (including PD-L1 expression, TILs, TGFβ/pSmad pathway-related protein, tumor micro-environment gene expression, etc.) assays to explore the relationship between the biomarkers and the drug’s efficacy. The tumor tissue samples may be archived ones (prior to the treatment) or freshly collected ones (freshly collected ones are preferred). For the harvesting/collection and disposal procedures of tumor specimens, refer to Laboratory Manual. For a few subjects who are unable to provide adequate tissue samples, it is up to the investigator and the sponsor to determine the eligibility of subject patients for the study by discussion.
- Concomitant medications/concomitant therapies: any and all concomitant medications/concomitant therapies within 30 days prior to the first dose of the investigational drug must be recorded.

The following screening should be done within 7 days prior to the start of investigational drug treatment; (serum) pregnancy test should be done within 7 days prior to the first dose of the investigational drug.

- Body height and body weight.

- ECOG score.
- Vital signs: pulse rate, respiratory rate, body temperature and blood pressure.
- Full physical examination: general conditions, head and face, skin, lymph nodes, eyes, ears, nose and throat, oral cavity, respiratory system, cardiovascular system, abdomen, reproductive-urinary system, musculoskeletal, nervous system and mental state, etc.
- Routine hematology: RBC, hemoglobin (Hb), platelet count (PLT), WBC, absolute neutrophil count (ANC) and lymphocyte count.
- Routine urinalysis: WBC, RBC, protein urine. If protein urine is ≥2＋, 24-h urine protein quantitative assay must be performed additionally.
- Fecal occult blood (OB): if fecal occult blood is positive, a retest must be performed; if the retest result is still positive, the subject might have active hemorrhage of digestive tract and, at the investigator's discretion, the subject might have to receive gastrointestinal endoscopy.
- Blood chemistry panel: ALT, AST, γ-GT, TBIL, DBIL, AKP, BUN or urea (BUN preferred), TP, albumin (ALB), creatinine (Cr), blood sugar (GLU), K^+, Na^+, Ca^{2+} and $Mg2^+$, Cl^-.
- Blood amylase and lipase: Blood amylase and lipase test should be performed once within 7 days prior to the first dose, and at times of clinically indicated during the study.
- Coagulation function: APTT, PT, TT, Fibrinogen (FIB), INR.
- Thyroid function: serum TSH, free triiodothyronine (FT3), free thyroxine (FT4); if FT3, FT4 are unavailable, it is acceptable to use (T)T3, (T)T4 in place of them.
- 12-lead ECG: QT, QTc and P-R interval should be scrutinized during ECG examinations. Should there be any abnormality, other applicable examinations/tests may be arranged at the investigator's discretion.
- Echocardiography: left ventricular ejection fraction (LVEF).
- Pregnancy test: For female subjects of childbearing age, a serum pregnancy test is scheduled to be done within 7 days prior to the first dose
- Serum CA19-9 assay

6.2. Treatment period

The treatment period starts from the start of the first dose in subjects and terminates at the end of the study treatment. The first dose of the investigational drug should be administered at a time as close as possible to the time when the subject's eligibility has been confirmed in the screening period. A treatment cycle consists of 21 days.

All investigations and evaluations (except for imaging examinations) should be completed within 3 days before dosing. The laboratory tests (routine hematology, routine urinalysis, fecal occult blood, blood chemistry panel, coagulation function and thyroid function, serum CA19-9 assay) and ECG examination schedule to be done on C1D1 can be omitted if corresponding baseline laboratory tests done within 7 days prior to the first dose are available.

- The following assessments have to be completed before dosing on D1 and D8 of each cycle:
    - Body weight
    - ECOG score standard

- Vital signs
- Physical examination: physical examination(s) should be performed when clinically indicated.
- Hematology
- Routine urinalysis (if protein urine is ≥2＋ during the study period, it is up to the investigator to determine the need for 24h urine protein quantitative assay or other tests in light of the subject's actual situations.
- Blood biochemistry
- Fecal occult blood (D1 only)
- Coagulation function (D1 only)
- Thyroid function (D1 only)
- 12-lead ECG (D1 only)
- Serum CA19-9 test: To be performed every other treatment cycle (C1D1, C3D1, C5D1…)

Furthermore, the following operations have to be completed on D1 and D8 of each cycle:

- SHR-1701 dosing (D1 only)
- Administration of AG regimen
- Recording AEs
- Recording concomitant medications and concomitant therapies

- Collection of immunogenicity and PK blood samples: Refer to the flow chart of the clinical trial
- Imaging examinations: Imaging examinations are performed once every 6 weeks in the first 12 months and once every 9 weeks afterwards in the study treatment period and as appropriate in case a new lesion is suspected; if a subject withdraws/is withdrawn from the study for any reason, he/she needs to receive imaging examination in a timely manner (in ±4 weeks of his/her withdrawal; if the subject's previous examination is no more than 4 weeks away from his/her discontinuation of treatment, then no re-examination is required at his/her withdrawal). The conditions for the imaging examinations should be the same with those for baseline examination (including scan slice thickness, contrast agent, etc.). The acceptable time window for an imaging examination is the scheduled date±7 days; it is acceptable to perform unscheduled imaging examination at times of suspected disease progression (e.g., symptomatic deterioration). Time data are based on calendar day and will not be adjusted in response to the delay of the start of cycle.

Subjects who have achieved CR and PR should receive a re-assessment of their tumors by imaging examination for efficacy confirmation. The re-assessment should be done no later than 4 weeks after the first response; it is acceptable to carry out the re-assessment 4 weeks after the first response or at the next scheduled time for imaging examination. If the time interval between an imaging examination of subject and the next scheduled examination is less than 4 weeks, then the next scheduled imaging examination can be omitted on the premise that subsequent imaging examinations will be resumed at their scheduled time points.

If a schedule visit date in the protocol is in violation with a holiday or other special circumstances, the visit date can be re-scheduled to a workday that is closest to the originally

schedule date.

6.3. End-of-treatment/Withdrawal visit

The following safety assessments and operations have to be done at subject's decision of treatment withdrawal and/or study withdrawal ±3days. If safety assessment related examinations have been completed within 7 days prior to the discontinuation of study treatment, then none of such examinations has to be repeated at this stage.

- Body weight
- ECOG score standard
- Vital signs
- Full physical examination: general conditions, head and face, skin, lymph nodes, eyes, ears, nose and throat, oral cavity, respiratory system, cardiovascular system, abdomen, reproductive-urinary system, musculoskeletal, nervous system and mental state, etc.
- Hematology
- Urine routine
- Fecal occult blood
- Blood biochemistry
- Coagulation function
- Thyroid function
- 12-lead ECG
- Echocardiography
- Pregnancy test
- Serum CA19-9 assay
- Imaging examinations: An imaging examination has to be performed in a timely manner when a subject withdraws/is withdrawn from the study for any reason; if the subject's previous examination is no more than 4 weeks away from his/her discontinuation of treatment, then no re-examination is required at his/her withdrawal). Except for those who have radiologically confirmed disease progression, all subjects who discontinue the study treatment for other reasons should receive imaging examinations at protocol specified frequency until disease progression, the start of new antitumor therapy, lost to follow-up or death is registered.
- Collection of immunogenicity and PK blood samples: Refer to the flow chart of the clinical trial
- Recording AEs
- Recording of concomitant medications/concomitant therapies

6.4. Follow-up period

6.4.1. Safety follow-up period

The 1st safety follow-up visit is scheduled to be done 30 days (±7 days) after the last dose; at that visit, subjects should go to the study site for the following evaluations (if a subject starts new antitumor therapy before the scheduled 1st safety follow-up visit, then this visit should be

done before the start of the new antitumor therapy):

- Body weight
- ECOG score standard
- Vital signs
- Full physical examination: general conditions, head and face, skin, lymph nodes, eyes, ears, nose and throat, oral cavity, respiratory system, cardiovascular system, abdomen, reproductive-urinary system, musculoskeletal, nervous system and mental state, etc.
- Hematology
- Urine routine
- Blood biochemistry
- Fecal occult blood
- Coagulation function
- Thyroid function
- Serum A19-9
- Electrocardiogram
- Recording AEs
- Recording of concomitant medications/concomitant therapies

If laboratory tests (routine hematology, routine urinalysis, blood chemistry panel, coagulation function, thyroid function) and ECG examination corresponding to those scheduled to be done at the 1st safety follow-up visit have been done within 3 days prior to the visit, the scheduled tests and examinations can be omitted.

The 2nd safety follow-up visit (60 days±7 days after the last dose) and the 3rd safety follow-up visit (90 days±7 days after the last dose) can be done by phone call or other valid method. Information to be collected includes survival, concomitant medications/concomitant therapies and AEs.

During the follow-up period, PK and immunogenicity blood samples should be collected per the protocol if possible.

Except for those who have radiologically confirmed disease progression, all subjects who discontinue the study treatment for other reasons should receive imaging examinations at protocol specified frequency until disease progression, the start of new antitumor therapy, lost to follow-up or death is registered.

6.4.2. Survival follow-up period

At the end of the safety follow-up period, the subjects proceed to the survival follow-up period. The survival follow-up period lasts until subject's death, lost to follow-up, withdrawal of ICF and refusal to provide further information or until termination of the study by the sponsor. During this period, 1 follow-up visit will be carried out every 1 month for collection of survival information and subsequent antitumor treatment information (if the subject has started a new antitumor therapy, the regimen and start/end date of the treatment should be recorded).

Subjects who have no radiologically-confirmed disease progression before the end of treatment should receive imaging evaluations per the efficacy evaluation frequency specified for the current study until disease progression, death, lost to follow-up, withdrawal of consent and

refusal to provide further information, the start of other antitumor therapy, or until the termination of the study by the sponsor; radiological evidences of PD in such subjects should be collected in possible.

6.5. Unscheduled Visits

If any subject is in need of unscheduled follow-up visit for AE(s) during the study, the following should be recorded:

- Concomitant medications/concomitant therapies
- Recording AEs;
- Relevant examinations performed (including imaging examinations, if any);

7. Study evaluation:

7.1. Safety Evaluation

7.1.1. Safety parameter

In this study, safety parameters include clinical symptoms, vital signs, physical examination, laboratory tests (routine hematology, routine urinalysis, blood chemistry panel, thyroid function, coagulation function, etc.). AEs observed should be assessed for their type, incidence, severity, start and end time, eligibility for SAE, relatedness to the investigational drug, and outcome per CI CTCAE 5.0.

7.1.2. Definition of AE

Adverse Event (AE) refers to any untoward medical occurrence in a patient or a clinical study subject administered with a study drug, but it does not necessarily have a causal relationship with the treatment. AEs may be any unexpected untoward symptoms, signs, lab abnormalities or diseases, including at least the following situations:

1) Worsening of pre-existing (before entering the clinical study) medical conditions/diseases (including worsening of symptoms, signs and laboratory abnormalities);
2) Any new adverse medical condition (including symptoms, signs and newly diagnosed diseases);
3) Any abnormal laboratory values or results of clinical significance.

"Disease progression" is bound to occur in the population of this study and should not be reported as a PT for AEs. Whenever disease progression is observed, the event that justifies the disease progression should be reported as an AE. Example: for a subject who experiences a seizure that is determined to be related to brain metastases, the recorded PT for the AE should be Seizure rather than Disease progression or Brain metastases.

The investigators should record any and all AEs occurred in the subjects. The recorded information should include: descriptions of AE and all of its related symptoms, date of occurrence, severity and relatedness to the investigational drug, duration, measures taken, and final results and outcome.

7.1.3. Definition of serious adverse events (SAEs)

An SAE refers to any of the following untoward medical events in subjects after the administration of the investigational drug at any dose level.

- An event leading to death;

- Life-threatening event (it is defined that the subject is in danger of immediate death at the time of the event);
- An event requiring hospitalization or prolonged hospitalization;
- Events leading to permanent or serious disability/incompetence/work capacity impairment;
- Congenital anomaly or birth defect;
- Other important medical event (it is defined as an event that jeopardizes the subject or requires intervention to prevent any of the above situations.).

AEs leading to hospitalization or prolonged hospitalization in clinical study should be considered as SAEs. Hospitalization does not include the following:

- ✓ Rehabilitation facility
- ✓ Sanatorium
- ✓ Admission to conventional emergency room
- ✓ Day surgery (e.g. outpatient/day/ambulatory surgery)
- ✓ Social reasons (e.g., medical insurance reimbursement)
- ✓ Hospitalization or prolonged hospitalization not associated with worsening of AEs was not a SAE. Example:
  - Admission to hospital because of the original disease and without new AE or aggravation of the original disease (for example, in order to examine the laboratory examination abnormality persisting before the examination until now);
  - Hospitalization for management reason (for example, annual routine physical examination);
  - Hospitalization specified in the trial protocol during clinical trial (for example, operation as required in the trial protocol);
  - Hospitalization unrelated to the worsening of AEs (for example, elective cosmetic surgery);
  - Scheduled treatment or surgery should be recorded in the whole trial protocol and/or the baseline data of the subject;
  - Hospitalization solely for use of blood products.

Diagnostic or therapeutic invasive (e.g. surgery), non-invasive procedures should not be reported as an AE. However, when the disease condition leading to these procedures meets the definition of AE, it should be reported. For example, acute appendicitis occurring during the reporting of AE should be reported as an AE, and appendectomy should be recorded as the treatment method of this AE.

7.1.4. Disease progression and death

Disease progression is defined as the deterioration of the subject's condition caused by the indications of the study, including radiographic progression and progression of clinical symptoms and signs. New metastases from the primary tumor, or progression of existing metastases, are considered as disease progression. Events that are life-threatening, require hospitalization or prolonged hospitalization, or result in permanent or significant disability/functional insufficiency/work capacity impairment, congenial abnormaly or birth

defects will not be reported as SAEs. If there is any uncertainty as to whether an SAE is due to disease progression, it should be reported as an SAE.

Any and all subject deaths, regardless of the involvement state of new antitumor therapy, should be reported as SAEs (see 7.3 Follow-up and report of AEs). Any and all deaths potentially attributable to PD symptoms and signs in the opinion of the investigator should be recorded in eCRF and reported as SAEs. The term "death" should not be used as an AE or SAE term, but as an outcome of an event, and an event leading to death should be recorded as an AE or SAE. If the cause of death can not be determined at the time of reporting, the AE or SAE term will be recorded as "unexplained death". Disease progression can be used for reporting an SAE only when the PD has led to subject death and the investigator fails to determine the specific event that result in the death.

7.1.5. Immune-mediated AEs

Immune-mediated AEs (IMAEs) refer to specific events (including: pneumonia, diarrhea/colitis, hepatitis, nephritis/renal dysfunction, rash and endocrinopathy, etc.) found in subjects on immunosuppressant therapies. Endocrine events (hypothyroidism/thyroiditis, hyperthyroidism, hypophysitis, diabetes mellitus, adrenal gland insufficiency) are generally excluded because these events can be treated without medical intervention with immunosuppressants. Presented in Table 18 are PTs included in IMAE analysis for supporting warnings and precautions.

Table 18 PTs included in IMAE analysis for supporting warnings and precautions

IMAE category	PTs included IMAE category (coded per MedDRA)
Pneumonia	Pneumonia, interstitial pneumonitis
Diarrhoea/colitis	Diarrhea, colitis, enterocolitis
Hepatitis	Hepatotoxicity, hepatitis, acute hepatitis, autoimmune hepatitis, AST increased, ALT increased, bilirubin increased, ALP increased
Adrenal insufficiency	Adrenal insufficiency
Hypothyroidism/thyroiditis	Acute thyroiditis (prostration for frequent thyroiditis), autoimmune thyroiditis (prostration for frequent thyroiditis)
Hyperthyroidism	Hyperthyroidism
Hypophysitis	Hypophysitis
Diabetes	Diabetes mellitus, diabetic ketoacidosis
Nephritis and kidney dysfunction	Nephritis, acute nephritis, tubulointerstitial nephritis, acute kidney failure, kidney failure, creatinine increased
Rash	Skin rash, maculopapular rash

7.1.6. Adverse events of special interest

For the reporting of AEs of special interest (SIEs) as defined in the clinical study protocol, Hengrui's Clinical Study Serious Adverse Event/Adverse Event of Special Interest Report Form should be completed within 24 hours of the investigator's awareness and reported to the sponsor. If an SIE is also an SAE, two Henrui Clinical Trial SAEs/SIEs Report Forms should be filled out and submitted to relevant departments in accordance with SAE reporting procedures.

Abnormal liver function: Abnormal AST and/or ALT levels concurrent with an abnormal elevation in total bilirubin level that meet criteria (1) (2) (3) in Table 19 with no other causes leading to abnormality, should always be reported as an SIE; if it conforms to the SAE definition, it should also be reported following the SAE reporting procedures.

Table 19 Evaluation of abnormal liver function tests

Conditions met	Judgment criteria
(1) Abnormal ALT or AST	Normal at baseline: ALT or AST during the treatment period > 3×ULN;<br>Abnormal at baseline: ALT or AST during the treatment period > 2×baseline level, with value > 3× ULN; or value > 8× ULN.
(2) Abnormal TBIL	Normal at baseline: TBIL during the treatment period> 2×ULN;<br>Abnormal at baseline: TBIL increased during the treatment period> 1×ULN or its value> 3×ULN.
(3) No hemolysis and alkaline phosphatase < 2 × ULN (or no information available)	

If a subject has abnormal increases of AST and/or ALT levels and abnormal elevation in total bilirubin level during the treatment or follow-up period, the subject should return to the site for assessment as soon as possible (preferably within 48 hours) after learning of the abnormal results. The assessment should include laboratory tests, detailed medical history, physical examination, and the possibility of (primary or secondary) liver tumor.

In addition to AST and ALT retests, laboratory investigations need to do include albumin, creatine kinase, TBIL, direct and indirect bilirubin, γ-glutamyltranspeptidase, PT/INR, AKP. Detailed medical history collection recommendations include: history of alcohol consumption, acetaminophen, soft drugs, various supplements, family medical history, occupational exposure, sexual behavior history, travel history, history of contact with jaundiced patients, surgery, blood transfusion, history of liver disease or allergic diseases. Further tests may also include the testing for acute hepatitis A, B, C, D, and E and liver imaging examination (e.g., biliary tract).

7.2. Classification of AEs and SAEs

7.2.1. Criteria for judging the severity of adverse events

Refer to the grading criteria in NCI-CTC AE 5.0. In case of AEs not listed in NCI-CTCAE 5.0, the following criteria can be referred to (Table 20):

Table 20 Severity criteria of AEs

Grade	Clinical description of severity
1	Mild; asymptomatic or minimal symptoms; clinical or laboratory abnormalities only; intervention not indicated
2	Moderate; minimal, local, or noninvasive intervention indicated; limited age-appropriate instrumental activities of daily living (ADL); instrumental activities of daily living (ADL) refer to preparing meals, shopping, using the telephone, managing money, etc.
3	Severe or medically significant but not immediately life-threatening; hospitalization or prolonged hospitalization indicated; disabling; limiting self-care ADL. Self-care refers to bathing, dressing, undressing, eating, using toilet, taking medications, etc., not being bedridden
4	Life-threatening; emergency treatment indicated
5	Results in death

7.2.2. Determination of the correlation between an AE and the investigational drug

The investigator should determine the relatedness of AEs to the investigational drug comprehensively from, for example, the reasonable time sequence of the occurrence of AE to the administration of the drug, the characteristics of the investigational drug, the pharmacology/toxicology actions of the investigational drug, the use of other concomitant medications, the underlying disease, previous medical history, family history of the subject, and response to challenge and re-challenge, etc. The investigator should assess the potential relatedness between AEs and the investigational drug as “definitely related, possibly related,

possibly not related, definitely not related, and indeterminate".

7.3. Follow-up and report of AEs

7.3.1. Follow-up of AEs/SAEs/SIEs

All AEs/SAEs/SIEs should be followed up until resolution and the regression of relevant laboratory investigation results to baseline level, the degradation to Grade ≤1, the attainment of stable state, or the availability of a reasonable explanation (e.g., lost to follow-up, death); or final determination of that they are not related to the investigational drug/study process at the end of the safety follow-up period; if possible, best outcome and definite determination of relatedness of AEs to the investigational drug should be achieved for the subjects.

At each follow-up visit, the investigator should inquire about the occurrence of AEs/SAEs/SIEs and provide follow-up information timely according to the sponsor's requirements. For the principles on the collection of AEs/SAEs/SIEs from subjects at the end of the study after the last dose, refer to Table 21

Table 21 Principles for AEs/SAEs/SIEs collection and follow-up period

Collection period	Category
The 30 days after last dose (including postdose D30)	All AEs/SAEs will be collected
30 days to 90 days after the last dose	All SHR-1701-related AEs will be collected
	All SAEs/SIEs will be collected if no new antitumor therapy has been started;<br>If new antitumor therapy has been started, then only study medication related SAEs/SIEs and all fatal SAEs will be collected after the start of the new antitumor therapy.
90 days after the last dose	All investigational drug related SAEs/SIEs will be collected.

Safety follow-up period: The subjects should be followed up to 90±7 days after the last dose of SHR-1701, or 30 days after the last dose of gemcitabine/albumin-bound paclitaxel, whichever occurs later.

7.3.2. Report of serious adverse events

The collection of SAEs should start from subject's signing of ICF and terminate at the end of the safety follow-up period. For the specific collection and follow-up principles, refer to Table 21 Principles for collection and follow-up of AEs/SAEs/SIEs. In case of an SAE, whether it is an initial report or a follow-up report, the investigator must immediately complete, sign and date a Henrui Clinical Trial SAEs/SIEs Report Form; moreover, the investigator should report the SAE to the sponsor within 24h of his/her awareness of the SAE, and report to relevant authorities according to local regulatory requirements.

Safety information in this study should be reported to the sponsor via the e-mail address

hengrui_drug_safety@hrglobe.cn

After the safety follow-up period, SAEs suspectedly related to the investigational drug will be collected. SAE information to be recorded in detail include the symptoms, severity, relatedness to the investigational drug, occurrence time, treatment time, measures taken, follow-up time and method, and outcome. If the investigator considers an SAE not related to the investigational drug but potentially related to study conditions (such as termination of original treatment, or comorbidities during the study), this relationship should be described in detail in the narrative

section of the SAE report form. If the intensity of an ongoing SAE or its relationship with the investigational drug changed, a follow-up report should be submitted promptly. If the investigator identifies any erroneously reported SAE information, he/she can correct, cancel or degrade the SAE in follow-up report and explain, and following the SAE reporting procedures to submit a report on the issue.

7.3.3. Pregnancy reporting

If a female subject becomes pregnant during the clinical study, the subject must discontinue her study treatment and withdraw from the study. If the partner of a male subject becomes pregnant during the clinical study, the subject can stay in the clinical study. The investigator should complete the Hengrui's Clinical Study Pregnancy Report/Follow-up Form and report it to the sponsor within 24 hours after learning of the pregnancy event.

The investigator needs to follow up the pregnancy event until 1 month after delivery, and report the result to the sponsor.

Still birth, spontaneous abortion, or fetal anomaly out of a pregnancy is considered as an SAE and required to be reported within the time limit for SAE reporting.

If a subject experiences a concurrent SAE during pregnancy, then a copy of Henrui Clinical Trial SAEs/SIEs Report Form should be filled out and the SAE should be reported in accordance with the SOP for reporting SAEs.

7.4. Assessment of Biomarker(s)

- Whole blood samples will be collected regularly and used for analysis of the immunogenicity f SHR-1701 in serum (anti-SHR-1701 antibodies).
- Tumor tissue samples will be exploratorily analyzed for biomarkers including but not limited to PD-L1 expression, TILs, TGFβ/pSmad pathway-related protein, tumor microenvironment gene expression and their correlation with the drug's efficacy.

7.5. Efficacy Evaluation

7.5.1. Efficacy parameters

The efficacy indicators in this study include ORR, DCR, PFS, DoR and OS.

Objective Response Rate (ORR): The proportion of subjects who receive the study treatment and the best overall response (BOR) is evaluated as complete response (CR) or partial response (PR) according to RECIST 1.1.

Duration of response (DoR): defined as the time from the date of first recorded tumor response (evaluated according to RECIST v1.1 criteria) to the date of first recorded objective tumor progression (evaluated according to RECIST v1.1 criteria) or death from any cause, whichever occurs first.

Best Overall Response (BOR) refers to the best response evaluated by the investigator, which is recorded during the period from the date of enrollment to the date of objectively recording the progression according to the RECIST 1.1 or the date of starting the follow-up anti-tumor therapy, whichever comes first. For subjects without PD record or subsequent antitumor therapy, the BOR will be determined according to all the response evaluation results.

Disease Control Rate (DCR): The proportion of subjects who receive the study treatment and the best overall response (BOR) is evaluated as complete response (CR), partial response (PR), and stable disease (SD) according to RECIST 1.1.

Progression Free Survival (PFS): defined as the period from the date of the first dose to the date

of first recorded tumor progression or all-cause death, whichever occurs first, per RECIST 1.1.

Overall Survive (OS): OS refers to the period from the date of first dose to the date of all-cause death.

7.5.2. Criteria for efficacy assessment

Indicators such as ORR, DCR, PFS and DoR are assessed by imaging techniques per RECIST 1.1 (Annex 4 Response Evaluation Criteria in Solid Tumors).

Survival recording and evaluation: Discharged subjects will be followed up by phone or other valid method once per month for their survival until their death, lost to follow-up, withdrawal of ICF, or until the termination of the study by the sponsor.

Tumor response evaluation will cover all known or suspected disease sites. Imaging studies include computed tomography (CT) or magnetic resonance imaging (MRI) scans of the chest, abdomen, or pelvis; brain MRI for subjects with known or suspected brain metastases; bone scans and/or bone x-rays for subjects with known or suspected bone metastases.

Radiological evaluation in screening period will be conducted within 28 days prior to the first dose of the investigational drug, first imaging evaluation of tumor will be carried out 6 weeks (±7 days) after the start of the study treatment. During the study treatment, radiological evaluation will be carried out once every 2 cycles (6 weeks±7 days) in the first 12 month, once every 3 cycles (9 weeks±7 days) afterwards, until subject's PD, lost to follow-up, death, withdrawal of ICF, start of other antitumor therapy or until termination of the study by the sponsor.

The same imaging technique should be used to evaluate the same type of lesions at subsequent tumor assessments as at screening. Assessment of anti-tumor activity will be performed during the screening period and during treatment by radiographic imaging according to the trial flow chart; it should also be performed when disease progression is suspected (e.g., symptomatic deterioration) and the subject withdraws from treatment (if the assessment has not been completed in the previous 4 weeks). Once PD is observed, the affected subject(s) should receive physical examination and radiographic confirmation right away rather than at the next scheduled imaging examination. If the unscheduled imaging examination reveal clinical progression that does not meet the criteria for PD in RECIST 1.1, the next imaging examination will be carried out on the originally scheduled date unless the next scheduled examination is less than 14 days away from the unscheduled one.

Subjects should be followed up continually in accordance with the study protocol and the follow-up is not affected by dose interruption or dose delay. If a subject wants to withdraw from the study treatment because of deterioration of his/her general health but no objective evidences are available for the time being, the clinical progression should be reported as Clinical progression of tumor. Best efforts should be exercised to record the objective progression of the disease (e.g, by imaging examination) even after withdrawal from the treatment.

All subjects' documentation and images data must be verified for their sources and reviewed by peers.

7.6. Pharmacokinetic Evaluation

In PK studies, serum drug concentration will be determined at the following time points: 0.5h before dosing and 10min after dosing of SHR-1701 on C1D1, C2D1, C4D1; within 0.5h before dosing of SHR-1701 on D1 of Cycle 7 and after every 6 cycles thereafter; on the day of subject's withdrawal from the treatment, and 30 days (±7 days) after the last dose. At each time point, 3.5 mL of venous blood will be collected into a serum separator tube for PK study of SHR-1701.

The major PK parameters to be investigated include but are not limited to: (1) C_{trough}; (2) C_{max}.

7.7. Immunogenicity assessment

In immunogenicity assessment, 1 blood sample will be collected 0.5h before dosing of SHR-1701 on C1D1, C2D1, C4D1, C7D1, on D1 every 6 cycles thereafter, on EOT day, and 30days (± 7 days) after the last dose, respectively, for determination of anti-SHR-1701 antibodies (including ADA and Nab). At each time point, 5mL of venous blood will be collected into a serum separator tube for immunogenicity assay. The immunogenicity of SHR-1701 during the treatment period will be assessed by comparing subject's determination data vs. subject's baseline data. The immunogenicity of SHR-1701 will be comprehensively analyzed in light of the drug's plasma concentration, safety and effectiveness day.

8. Data analysis/statistical method

Data analysis will be completed by Henrui and/or an CRO. For the analysis, the data from all sites were pooled.

Subjects who fail the screening will not be administered any study treatment in spite of their signing of the ICF and will be excluded from all analyses; data from these subjects will be presented in tabular listing and reported.

Reasons for withdrawal from the trial will be summarized and listed. The list should include the following information: date of first dose and date of last dose, exposure duration of the investigational drug, and subject’s date of withdrawal.

8.1. Statistical Analysis Plan

The detailed summary and statistical analysis methods of the data collected by this study will be included in the statistical analysis plan (SAP), finalized and filed by the sponsor before database lock. In response to any and all protocol modifications deemed to have considerable impact on the SAP in the opinions of the sponsor or the principal investigator, the SAP needs to be revised to remain in agreement with the study protocol. In the SAP, relevant information in this protocol might be revised; however, any and all revisions involving the major and/or critical factors of the protocol (e.g., definitions and analyses of endpoints) need to be reflected in the protocol revisions.

8.2. Sample Size

Phase Ib: Dose-finding stage

Sample size: about 6-12 subjects.

Phase II: Efficacy extension stage

When sample size is 49 subjects, exact test at the significance level(α) of 0.05 will be able to provide a 95% CI [16%,44%] for the ORR of the Combination Therapy Group (SHR-1701 + gemcitabine + albumin-bound paclitaxel); the accuracy of ORR (half width of the CI is 14%) is 30%±14%, taken into consideration a dropout rate of 10%, it is estimated that 54 subjects will be enrolled.

For the key secondary endpoint (OS rates at 9-month), this sample size is also able to detect a difference of 20% with 80% power, i.e. 50% vs. 70%.

The 6 subjects who are enrolled at stage 1 will proceed to stage 2 of the study. Therefore, the total sample size of this study will be about 54-60 subjects.

8.3. Populations for analysis

The following analysis sets will be involved in this study:

- Full analysis set (FAS): All subjects who are enrolled and administered at least a dose of the investigational drug are included. This set will be used for effectiveness analysis.
- Safety set (SS): the same with FAS. This set is the primary analysis population for safety analysis of this study.
- Clinically significant toxicity analysis set: By definition, this set includes all subjects who receive the co-medication to the end of Cycle 1(21 days in total) or discontinue their treatment because of the occurrence of clinically significant toxicity are included in this set.
- Per protocol set (PPS): PPS is a subset of FAS; all subjects with significant protocol deviations that have been determined to have significant impact on efficacy will be excluded from this set. The list of subjects to be included in or excluded from PPS should be reviewed and determined by the sponsor and investigator before locking of database.
- Evaluable Set (ES): ES is a subset of FAS; by definition, it includes all enrolled subjects who have been administered at least 1 dose of the investigational drug and subjected to at least 1 post-baseline tumor assessment.
- PK analysis set (PKAS): All enrolled subjects who have been administered at least 1 dose of the investigational drug and have post-dose PK evaluation data constitute the PKAS of this study.
- Immunogenicity Analysis Set: All subjects who are enrolled in the study and receive the investigational drug at least once, and have baseline and at least one post-baseline immunogenicity evaluation data.

8.4. Statistical Methods

8.4.1. General Analysis

In this study, unless otherwise specified, the data will be summarized using descriptive statistics according to the following general principles.

Measurement data are summarized by mean, standard deviation (SD), median, maximum, and minimum; count data are summarized by frequency number and percentage; time-event data are subjected to Kaplan-Meier estimation of survival rate and plotting of survival curve; plasma concentration data are summarized by geometric mean, geometric SD, geometric coefficient of variation (CV), mean, SD, CV, median, maximum, and minimum. If necessary, corresponding 95% CIs are provided for the above-mentioned analyses.

Baseline is defined as the latest test data prior to the first dose.

In this study, missing data of efficacy indicators will not be processed specifically. In safety assessment, missing data will not be estimated.

8.4.2. Safety analysis

Safety analysis will be based on SS; clinically significant toxicity analysis is based on clinically significant toxicity analysis set.

Treatment Groups will be subjected to descriptive summary of the incidence of clinically significant toxicity events by SOC and PT. The start time, type, name, disposable measures, duration and outcome of clinically significant toxicities will be listed.

According to Henrui's reporting SOPs, safety analysis is limited to summary of descriptive statistics, including but not limited to the following. For detailed analyses, refer to SAP.

- Analysis of patient discontinuation, dose reduction or suspension due to AEs;
- Summary of (all-cause and treatment-related) adverse events (AEs);
- Occurrence and severity of (all-cause and treatment-related) AEs.
- Summary of details of SAEs;
- Analysis of relatedness of AEs;
- Occurrences of abnormal laboratory indexes, vital signs, ECG data.

8.4.3. Pharmacokinetic analysis

Pharmacokinetic analysis is based on PK analysis set. PK parameters such as C_{trough} and C_{max} will be subjected to statistical description (number of subjects, mean, SD, median, minimum and maximum) by dose group and scheduled blood sampling time point. Drug concentration-time profiles will be plotted for individual subjects based on their actual sampling time; mean and/or median drug concentration-time curves (linear scale and logarithmic scale) will be plotted for all subjects by dose group and scheduled sampling time.

8.4.4. Efficacy analysis

Analysis of effectiveness will be based o FAS, PPS and ES.

ORR: Clopper-Pearson method will be used to estimate the 95% CI of the estimated value of this indicator.

DCR: Clopper-Pearson method will be used to estimate the 95% CI of the estimated value of this indicator.

BOR: CR, PR, SD, PD will be subjected to descriptive statistical analysis.

DoR: median DoR will be estimated by Kaplan-Meier (KM) method, and the two-sided 95% CI of the indicator will be calculated.

TTP: median TTP will be estimated by Kaplan-Meier (KM) method, and the two-sided 95% CI of the indicator will be calculated.

PFS: median PFS will be estimated by Kaplan-Meier (KM) method, and the two-sided 95% CI of the indicator will be calculated. Survival curve will be plotted by Kaplan-Meier (KM) method.

OS: median OS, OS rates at 6-month, OS rates at 9-month, OS rates at 12-month will be estimated by KM method, and the two-sided 95% CIs of the indicators will be calculated. Survival curve will be plotted by Kaplan-Meier (KM) method.

8.4.5. Immunogenicity study

Immunogenicity analysis will be based on immunogenicity analysis set. Positive rate of anti-SHR-1701 antibodies, proportion of anti-SHR-1701 antibodies with neutralizing activity, and correlation between anti-SHR-1701 antibody and the drug's Ctrough, safety and effectiveness will be summarized and analyzed.

8.4.6. Other analyses

SHR-1701-related solid tumor markers, such as PD-L1 expression in tumor tissue, TILs, TGFβ/pSmad pathway-related protein level, tumor microenvironment gene expression, etc., and the relationship between these biomarkers and clinical efficacy will be summarized by descriptive statistics.

9. Data Management Method

The sponsor is responsible for the clinical trial data management of this study, and uses an

electronic data capture (EDC) system to collect and manage the study data.

9.1. Data acquisition

9.1.1. Completion of Electronic Case Report Form (eCRF)

The eCRF will be completed by the investigator or data entry personnel via the EDC system. The ECRF should be completed in a timely manner to ensure that the completed data are traceable from the original records. When data modification is performed in EDC, it is required to fill in the reason for data modification according to the system prompts. The modification history and reasons for modification will be documented in the audit trail of the EDC system. The investigator or his/her authorized person needs to confirm the authenticity, completeness, and timeliness of the eCRF data and electronically sign in the EDC system.

9.1.2. Use of Electronic Data Capture (EDC) system

The data manager will build the eCRF and logic verification program in the EDC system according to the study protocol, and complete the user acceptance test and go-live use before the first subject is enrolled. All EDC users will be required to complete the relevant training and archive the training records in order to gain access to the study eCRF. When a user attaches his/her electronic signature to the eCRF, he/she needs to confirm and agree with the statement of use of the electronic signature. The account is limited to the user's own use, and the password needs to be kept properly and changed regularly. When there is a change of personnel in the study team, the authority needs to be cancelled in time.

9.2. Data Management

9.2.1. eCRF data review

The logic verification program of EDC system will check the integrity and logic of the entered data and raise queries for data that may have problems. The investigator or data entry personnel may correct the data or interpret and confirm it by answering queries. The medical monitor, the data manager, and the medical reviewer will also review the eCRF data and raise queries for questionable data if necessary. The investigator should answer queries from the system and the data reviewer in a timely manner, and may raise queries several times until the data issue is resolved if necessary.

9.2.2. Data review meeting and database lock

Prior to database lock, the study team needs to complete data cleaning, summarize all protocol deviation events that occur during the study, and hold a data review meeting to determine the analysis population. Decisions made during the data review meeting will be documented. After the data review meeting is approved, the study team will confirm that the study database in the EDC system is locked, and no changes can be made to the data after locking.

9.2.3. Data archiving

After the study is completed, the EDC system needs to generate the eCRF of subjects in PDF format, which will be stored on CD-ROMs and archived by the sponsor and each institution for audit. The study data should be stored and managed according to GCP requirements, and the investigator should inform the sponsor in advance when destroying any documents or records related to the study. The sponsor should keep the clinical study data for at least 15 years after the investigational drug is approved for marketing, or even if the clinical study is terminated early.

9.3. Protocol deviations

Protocol deviations denotes any and all noncompliances to the clinical trial protocol, GCP or SOPs. The non-compliances in practice may occur in subjects, investigators, or other researchers.

Study sites must prepare corresponding corrective measures that are immediately implementable in case of the occurrence of deviations.

Study sites are under the obligations to remain continuously cautious in order to timely recognize protocol deviations and complete the actions required in the scheduled protocol for prompt identification and reporting of protocol deviations. All deviations must be recorded in original documents of the study and reported to the sponsor. Protocol deviations must be submitted to local IRB in accordance with local ethical regulatory requirements. The PIs or investigators at study sites should be responsible for understanding of and abiding to local ethical codes.

10. Original documents and acquisition of source data/files.

In accordance with ICH E6, relevant regulations, and the research institution's requirements for the protection of the subject's personal information, each site must properly maintain the treatment and scientific research records related to this study. As part of Hengrui's sponsorship or participation in the study, each site shall allow the sponsor or its authorized representatives and the regulatory authority to inspect (and, if permitted by law, copy) clinical records for quality review, audit, and evaluation of safety, study progress, and data validity.

Original data are all information necessary for reconstruction and evaluation of the clinical study and are the original records of clinical findings, observations, or other activities. Examples of these original documents and data records include, but are not limited to: hospital records, laboratory records, memos, subject diary cards, pharmacy dispensing records, recordings of consultation meetings, recorded data from automated instruments, copies or transcriptions verified to be accurate and complete, microfiches, photographic negatives, microfilm or disks, radiographs, and documents and records of subjects kept in participating pharmacies, laboratories, and medico-technical departments.

The investigators will see to that all source data, whether written ones or electronically entered ones, are accurate, precise, synchronous, and accountable. In routine clinical trial activities, if computer systems (and/or any other type of electronic devices) are used to create (enter for the first time), modify, maintain, archive, retrieve or transmit source data by electronic means, such systems have to meet all applicable laws and regulations governing the use of electronic records and/or electronic signatures. Such systems might include but are not limited to electronic medical/health files, AE follow-up/reporting, assessment per protocol requirements, and/or reconciliation drug counting.

When written records obtained with such systems are used in place of electronic records for specified activities, such written records should have certified duplicates. The certified duplicates, which contain verified original information, date, and signature, are precise duplicates that have the same attributes and information with the original.

11. Quality Assurance and Quality Control

In order to ensure the quality of the study, the sponsor and the investigator will jointly discuss and develop the clinical study plan before the study officially begins, and will confirm whether the relevant study personnel participating in the study have received appropriate GCP training.

Study medications must be managed by each site in accordance with SOPs, including receipt, storage, dispensing, recovery, and destruction (if applicable).

According to the GCP guidelines, necessary steps shall be adopted in design and implementation phases of the study to ensure the data collected is accurate, consistent, complete and credible. All observed results and abnormal findings in the clinical study should be timely verified and recorded to ensure the reliability of data. The instruments, equipment, reagents and standards used for various inspection items in the clinical study should have strict quality standards and

ensure that they can work in normal state.

The investigator will enter the information required by the protocol into the eCRF, and the medical monitor will verify whether it is completed completely and accurately, and guide the staff of the site to make necessary corrections and additions.

The drug regulatory authority, the institutional review board (IRB)/the independent ethics committee (IEC), the medical monitor and/or the auditor of the sponsor may conduct a systematic inspection on study-related activities and documents to evaluate whether the study is conducted in accordance with the study protocol, SOPs, and relevant regulatory requirements (e.g., GLP and GMP), and whether the study data are recorded in a timely, true, accurate, and complete manner. Audits should be performed by personnel not directly involved in the clinical study.

12. Regulations, ethics, informed consent and subject protection

12.1. Regulatory considerations

According to applicable Chinese regulatory requirements, the clinical trial of a drug can be legitimately carried out only after relevant clinical trial application (CTA) dossiers have been submitted to CFDA for review and approval and CFDA has granted the letter of approval. The CTA approval document for SHR-1701 drug product is numbered 2018L02627.

The regulatory basis for the design of this clinical trial protocol (CTP):

1) Drug Registration Regulation
2) Good Clinical Practice
3) Consensus ethical principles of international standards, including the Declaration of Helsinki and the Council for International Organizations of Medical Sciences (CIOMS) International Ethics Code.
4) ICH guidelines
5) Other applicable laws and regulations

12.2. Ethical Norms

This trial protocol should be reviewed and approved in writing by the Ethics Committee of the hospital before implementation. The study protocol, protocol amendment, informed consent form and other relevant documents, such as recruitment advertisement, should be provided to the ethics committee. This clinical trial must comply with the Declaration of Helsinki, CFDA Good Clinical Practice (GCP) and applicable regulations. The study can be launched only after it is approved by the hospital’s EC.

Without the consent of both the sponsor and the investigator, neither party shall unilaterally modify the protocol of this study. The investigators may, prior to the acquisition of an approval from the EC/IRB, made modification(s) to the CTP in order to protect subjects against direct and immediate hazards. Moreover, the investigators should submit the modifications/changes and their rationales along with the proposed protocol amendment to the EC/IRB as soon as possible for review. The investigators must provide explanations for any and all protocol deviations and record such deviations.

During the clinical study, any modification to this study protocol should be submitted to the ethics committee, and other study documents should be modified accordingly if necessary, and submitted and/or reviewed in accordance with the requirements of the ethics committee. The investigator should be responsible for submitting the interim report regularly in accordance with the relevant requirements of the ethics committee, and should notify the ethics committee that

the study has ended after the end of the study.

12.3. institutional review board/independent ethics committee

Prior to the launch of the study, the CTP, ICF, subject enrollment ads and other documents for subjects must be reviewed and approved by the IRB/IEC. Subject enrollment cannot be started before IRB/IEC's approval of the CTP and ICF. Any and all amendments made to the CTP must be reviewed and approved by IRB/IEC. The investigator or the sponsor needs to submit the Investigator's Brochure (IB) or Instructions for Use (IFU) of the products and any and all updates of the documents to IRB/IEC for review and approval.

The investigator or the sponsor should follow the competent authority's requirements or the institution's article of incorporation to submit relevant reports, updates, and other documents (e.g., urgent safety reports, revisions, and management letters) to IRT/IEC.

12.4. Informed Consent

12.4.1. Informed consent form and other written information required by subjects

The ICF incorporates all elements specified in ICH guidelines and GCP and regulatory authority's requirements, meeting the ethical principles proposed in Declaration of Helsinki.

The informed consent form describes the study medication and study process in detail, and fully explains the risks of the study to the subjects. Written documentation of informed consent must be obtained before the subjects perform any study-related procedures.

All amendments to the informed consent form must also be approved by the ethics committee, and the ethics committee will decide whether the new version needs to be signed again for subjects who have signed the previous version of the informed consent form.

The ICF should state that subjects' personal ID records must be kept confidential, but the sponsor's authorized representative and regulatory authorities can have access to the subjects' information.

12.4.2. Informed consent process and records

Informed consent begins before the subjects agree to participate in the clinical study and continues throughout the clinical study. The investigator will discuss the risks and possible benefits of participating in the study in detail and fully with the subject or his/her legal representative. Subjects will be required to read the informed consent form approved by the ethics committee. The investigator will explain the clinical study to the subject and answer any questions that the subject may raise. Subjects may not start participating in the study until informed consent is obtained. Subjects can withdraw consent at any time during the course of the clinical study. A copy of the informed consent form will be retained by the subject. Even if the patient consulted refuses to participate in the study, his/her rights and interests will be fully protected and the quality of his/her medical care will not be affected in any way.

12.5. Confidentiality of Subject Information

Confidentiality of subject information shall be strictly enforced by the investigator, personnel participating in the study, the sponsor and its representative. Confidentiality also covers biological samples and genetic testing in addition to the clinical information of subjects. Therefore, the study protocol, documents, data and all other information generated therefrom will be kept strictly confidential. All relevant study or data information may not be disclosed to any unauthorized third party without the prior written approval from the sponsor.

Other authorized representatives of the sponsor, the IRB, regulatory authorities, and representatives of manufacturers of the study medications can inspect all documents and records

required to be maintained by the investigator, including, but not limited to, medical records and medication records of subjects. The site should allow access to these records.

Subject contact information will be kept securely at each site and will be used only internally during the study. At the end of the study, all records will continue to be kept in a safe place according to the timelines specified by the local IRB and regulations.

The study data of subjects collected for statistical analysis and science report should not include subjects' contact information or identity information. Instead, subjects and their study data will have a separate study identification number. The study data entry system and study management system used by study personnel at each clinical study site are secured and protected by pass words. At the end of the study, all identity information from the study database will be eliminated and archived.

13. Publication of Study Results

The study results are proprietary to Jiangsu Hengrui Pharmaceutical Co., Ltd. If the investigators plan to publish any study-related data and information, they should ask for Henrui's approval and, at least 30 days prior to submission for publication or other form of publication, provide Hengrui with the original manuscript, abstract or full text of the planned publication (posters, invited presentations or guest lectures). If patent applications have been submitted for protecting the study related intellectual property rights, the investigators should agree to put off the publication for NMT 60 days. Prior to the publication, Hengrui may require the investigators to remove any previously unpublished confidential information (except for study results). If the study is part of a multicenter study, the investigator needs to agree that the first publication is the overall result for all sites. However, if the original manuscript of the overall analysis has not been submitted within 12 months of completion or termination of the study at all sites, the investigator may request to publish the results separately in accordance with other requirements under this section.

14. Reference

1. Mohsen Naghavi, The global, regional, and national burden of pancreatic cancer and its attributable risk factors in 195 countries and territories, 1990–2017: a systematic analysis for the Global Burden of Disease Study 2017, Lancet Gastroenterology & Hepatology, 2019, 4(12): 934–947.
2. Siegel RL, Miller KD, Jemal A. Cancer statistics, 2019. CA Cancer J Clin, 2019, 69(1):7-34.
3. Zheng Rongshou, Sun Kexin, Zhang Siwei, et al.; Report of cancer epidemiology in China, 2015, Chinese Journal of Oncology, 2019, 41(1): 19-28
4. Feng RM, Zong YN, Cao SM, et al. Current cancer situation in China: good or bad news from the 2018 Global Cancer Statistics?. Cancer Commun (Lond), 2019, 39(1):22.
5. Roser, M., Ritchie, H. Cancer. Our World in Data. https://ourworldindata.org/cancer (2018).
6. Luo JF, Xiao LH, Wu CX, et al. The incidence and survival rate of population-based pancreatic cancer patients: shanghai cancer registry 2004-2009. PLoS One. 2013, 8(10): e76052.
7. Ben QW, Wang KX, Yuan YZ, et al. Pancreatic cancer incidence and outcome in relation to ABO blood groups among Han Chinese patients: a case-control study. 2011, 128(5):1179-86.
8. Burris HA, Moore MJ, Andersen J, et al. Improvements in Survival and Clinical Benefit With Gemcitabine as First-Line Therapy for Patients With Advanced Pancreas Cancer: A Randomized Trial. J Clin Oncol. 1997;15: 2403-2413.
9. Moore MJ, Goldstein D, Hamm J, et al. Erlotinib Plus Gemcitabine Compared With Gemcitabine Alone in Patients With Advanced Pancreatic Cancer: A Phase III Trial of the National Cancer Institute of Canada Clinical Trials Group. J Clin Oncol. 2007; 25:1960-1966.
10. Conroy T， Desseigne F, Ychou M, et al. FOLFIRINOX versus Gemcitabine for Metastatic Pancreatic Cancer, 2011 N Engl J Med. 2011, 364(19):1817-25.
11. Daniel D. Von Hoff, M.D. et al. Increased Survival in Pancreatic Cancer with nab-Paclitaxel plus Gemcitabine. N Engl J Med. 2013, 369:1691-1703
12. Ruihua Xu, Xianjun Yu, Jihui Hao. et al. Efficacy and safety of weekly nab-paclitaxel plus gemcitabine in Chinese patients with metastatic adenocarcinoma of the pancreas: a phase II study Xu et al. BMC Cancer. 2017, 17:885
13. Talia Golan, Pascal Hammel, Michele Reni, Maintenance Olaparib for Germline BRCA-Mutated Metastatic Pancreatic Cancer N Engl J Med. 2019, 381(4): 317-327.
14. Holter S, Borgida A, Dodd A, et al. Germline BRCA mutations in a large clinic-based cohort of patients with pancreatic adenocarcinoma. J Clin Oncol 2015; 33: 3124–9.
15. Skelton RA, Javed A, Zheng L, He J. Overcoming the resistance of pancreatic cancer to immune checkpoint inhibitors. J Surg Oncol 2017, 116:55–62.
16. Galluzzi L, Senovilla L, Zitvogel L, et al. The secret ally: immunostimulation by anticancer drugs. Nat Rev Drug Discov. 2012, 11:215–233.
17. Glen J. Weiss1, Lisa Blaydorn, Julia Beck, et al. Phase Ib/II study of gemcitabine, nab-paclitaxel, and pembrolizumab in metastaticpancreatic adenocarcinoma. Invest New Drugs; 2018, 36(1):96-102.

18. Phase I Study of Nivolumab + nab-Paclitaxel + Gemcitabine in Advanced Pancreatic Cancer. 2019 ASCO GI.

19. Julius Strauss, Christopher R. Heery, Jeffrey Schlom et al. Phase I Trial of M7824 (MSB0011359C), a Bifunctional Fusion Protein Targeting PD-L1 and TGFb, in Advanced Solid Tumors. Clin Cancer Res 2018, 24:1287-1295

Annex 1 ECOG PS score

Level	EOCG scale of performance status
0	Fully active, able to carry on all pre-disease performance without restriction
1	Can move around freely and engage in light physical activities, including general housework or office work, but cannot engage in heavier physical activities.
2	Ambulatory and capable of all selfcare but unable to carry out any work activities. Up and about more than 50% of waking hours
3	Can only take care of himself partly, and spend more than half of the time in bed or wheelchair.
4	Bedridden, unable to take care of himself.
5	Dead

Annex2 Cockroft-Gault equation (for calculation of creatinine clearance)

Serum creatinine concentration, in mg/dL

$$\text{Male creatinine clearance rate}\left(\frac{\text{ml}}{\text{min}}\right)=\frac{(140-\text{Age})\times\text{Body weight}}{72\times\text{Serum creatinine}}$$

$$\text{Female creatinine clearance}\left(\frac{\text{ml}}{\text{min}}\right)=\frac{0.85\times(140-\text{Age})\times\text{Body weight}}{72\times\text{Serum creatinine}}$$

Serum creatinine concentration, in μmol/L

$$\text{Male creatinine clearance rate}\left(\frac{\text{ml}}{\text{min}}\right)=\frac{(140-\text{Age})\times\text{Body weight}}{0.818\times\text{Serum creatinine}}$$

$$\text{Female creatinine clearance}\left(\frac{\text{ml}}{\text{min}}\right)=\frac{0.85\times(140-\text{Age})\times\text{Body weight}}{0.818\times\text{Serum creatinine}}$$

Unit of age: year-old; Unit of body weight: kg.

Annex 3 New York Heart Association (NYHA) Functional Classification

Heart failure symptom severity is poorly correlated with ventricular function, but clearly correlates with survival, and patients with mild symptoms may still have a higher absolute risk of hospitalization and death.

**Classification**	**Symptom**
I	No limitation of physical activity. Ordinary physical activity doesn't cause tiredness, heart palpitations, or shortness of breath.
II	Slight limitation of physical activity. Comfortable at rest, but ordinary physical activity results in tiredness, heart palpitations, or shortness of breath
III	Marked or noticeable limitations of physical activity. Comfortable at rest, but less than ordinary physical activity causes tiredness, heart palpitations, or shortness of breath.
IV	Unable to carry out any physical activity without discomfort. Symptoms also present at rest. If any physical activity is undertaken, discomfort increases. If intravenous administration is not required, Grade Iva is for those who can move indoors or at the bedside, and Grade IVh is for those who cannot get out of bed and require intravenous administration support

Annex 4 Response Evaluation Criteria in Solid Tumors

Response Evaluation Criteria in Solid Tumors Version 1.1 (excerpt)

(New Response Evaluation Criteria in Solid Tumors: Revised RECIST Version 1.1)

Note: This annex is an in-house translated document for reference only. In actual practices, the English version shall prevail.

1. Background

Omitted

2. Purpose

Omitted

3. Measurability of tumor at baseline

3.1 Definitions

At baseline, tumor lesions/lymph nodes will be categorized measurable or non-measurable as follows:

3.1.1 Measurable lesions

Tumor lesions: must be accurately measured in at least one dimension (longest diameter in the plane of measurement is to be recorded) with a minimum size of:

- CT scan 10 mm (CT scan slice thickness no greater than 5 mm)
- 10 mm caliper measurement by clinical exam (lesions which cannot be accurately measured with calipers should be recorded as non-measurable)
- Chest X-ray 20 mm
- Malignant lymph nodes: Pathologically enlarged and measurable. lymph nodes must be ≥ 15 mm in short diameter when assessed by CT scan (CT scan slice thickness recommended to be no greater than 5 mm). At baseline and follow-up, only the short axis will be measured and followed.

3.1.2 Unmeasurable lesions

All other lesions, including small lesions (longest diameter < 10 mm or pathological lymph node with ≥ 10 mm to < 15 mm short axis) and non-measurable lesions. Lesions considered truly non-measurable include: leptomeningeal disease, ascites, pleural or pericardial effusion, inflammatory breast disease, lymphangitis carcinomatosa of the skin/lung, abdominal mass not confirmed and followed by imaging, and cystic lesions.

3.1.3 Special considerations regarding lesion measurement

Bone lesions, cystic lesions, and lesions previously treated with local therapy require particular comment:

Bone lesions:

- Bone scans, PET scans, or photographs are not suitable for measuring bone lesions, but can be used to confirm the presence or absence of bone lesions;
- Lytic lesions or mixed lytic/osteoblastic lesions, with identifiable soft tissue components, that can be evaluated by cross-sectional imaging techniques such as CT or MRI can be considered as measurable lesions if the soft tissue component meets the definition of

measurability described above;

- Blastic bone lesions are non-measurable.

Cystic lesions:

- A lesion that meets the definition of a simple cyst in radiography should not be considered a malignant lesion because it is a simple cyst in the definition, and it is neither a measurable lesion nor an unmeasurable lesion;
- If it is a cystic metastatic lesion and meets the above definition of measurability, it can be treated as a measurable lesion. However, if there are non-cystic lesions in the same patient, non-cystic lesions should be preferred as target lesions.
- Locally treated lesions;
- Lesions situated in a previously irradiated area, or in an area subjected to other topical therapy, are usually considered non-measurable unless it has demonstrated clear progression in the lesion. The study protocol should detail the conditions under which such lesions can be considered measurable.

3.2 Description of measurement method

3.2.1 Lesion measurements

During clinical evaluation, all tumor measurements shall be recorded in metric system. All baseline assessments of tumor lesion size should be completed before the start of treatment, and must be completed within 28 days (4 weeks) before the start of treatment.

3.2.2 Evaluation method

The same techniques and methods should be used for baseline assessment and subsequent measurement of lesions. Except for lesions that cannot be evaluated by imaging but can only be evaluated by clinical examination, all lesions must be evaluated by imaging.

Clinical lesions: Clinical lesions will only be considered measurable when they are superficial and diameter ≥ 10 mm as assessed using calipers (e.g., skin nodules). For subjects with skin lesions, documentation by color photography with the size of the lesion measured by a ruler is recommended. When lesions can be evaluated by both clinical exam and imaging, imaging evaluation should be undertaken since it is more objective and may also be reviewed at the end of the study.

Chest X-ray: When tumor progression is an important endpoint, chest CT is preferred because CT is more sensitive than X-ray, especially for new lesions. Chest X-ray detection is only applicable when the boundary of the measured lesion is clear and the lungs are well ventilated.

CT, MRI: CT is currently the available, reproducible, best method for response assessment. The definition of measurability in this guideline is based on the CT scan thickness ≤ 5 mm. If the CT slice thickness is greater than 5 mm, the measurable lesion should be at least twice the slice thickness. MRI is also acceptable in some cases (such as a full-body scan).

Ultrasound: Ultrasound should not be used as a method to measure lesion size. Ultrasound inspection is not repeatable after the measurement due to its operational dependence, and cannot guarantee the sameness of technique and measurement between different measurements. If new lesions are identified by ultrasound in the course of the trial, confirmation by CT or MRI is advised. If there is concern about radiation exposure at CT, MRI may be used instead of CT.

Endoscopy, laparoscopy: The utilization of these techniques for objective tumor evaluation is not advised. However, they can be useful to confirm CR when biopsies are obtained or to determine

relapse in trials where relapse following CR or surgical resection is an endpoint.

Tumor markers: Tumor markers alone cannot be used to assess objective tumor response. However, if markers are present at baseline above the upper limit of normal, they must be normalized for a subject to be considered in complete response. Because tumor markers are disease specific, instructions for their measurement should be incorporated into protocols on a disease specific basis. Specific guidelines for both CA-125 response (in recurrent ovarian cancer) and PSA response (in recurrent prostate cancer), have been published. In addition, the International Gynecological Cancer Organization has formulated the CA-125 progress standard, which will soon be added to the objective tumor evaluation standard of the first-line treatment plan for ovarian cancer.

Cytological/histological techniques: These techniques can be used to differentiate between PR and CR in certain situations defined by the protocol (e.g. residual benign tumor tissue in lesions of germ cell tumors). When effusions are known to be a potential adverse effect of treatment (e.g. with certain taxane compounds or angiogenesis inhibitors), the cytological confirmation of the neoplastic origin of any effusion that appears or worsens during treatment can be considered if the measurable tumor has met criteria for response or stable disease in order to differentiate between response (or stable disease) and progressive disease.

4. Tumor response evaluation

4.1 Assessment of overall tumor burden and measurable disease

To assess objective response or future progression, it is necessary to estimate the overall tumor burden at baseline and use this as a comparator for subsequent measurements. Only patients with measurable disease at baseline should be included in protocols where objective tumor response is the primary endpoint. Measurable lesion is defined by the presence of at least one measurable lesion. In studies where the primary endpoint is tumor progression (either time to progression or proportion with progression at a fixed date), the protocol must specify if entry is restricted to those with measurable disease or whether patients having non-measurable disease only are also eligible.

4.2 Baseline Documentation of ‘Target’ and ‘Non-target’ Lesions

When more than one measurable lesion is present at baseline, all lesions up to a maximum of five lesions total (and a maximum of two lesions per organ) representative of all involved organs should be identified as target lesions and will be recorded and measured (this means in instances where subjects have only one or two organ sites involved, a maximum of two and four lesions respectively will be recorded).

Target lesions should be selected on the basis of their size (lesions with the longest diameter), be representative of all involved organs, but in addition should be those that lend themselves to reproducible repeated measurements. It may be the case that, on occasion, the largest lesion does not lend itself to reproducible measurement in which circumstance the next largest lesion which can be measured reproducibly should be selected.

Lymph nodes merit special mention since they are normal anatomical structures which may be visible by imaging even if not involved by tumor. Pathological nodes which are defined as measurable and may be identified as target lesions must meet the criterion of a short axis of ≥15mm by CT scan. The baseline only needs to detect the short axis. The short axis of the node is the diameter normally used by radiologists to judge if a node is involved by solid tumor. Nodal size is normally reported as two dimensions in the plane in which the image is obtained (for CT scan this is almost always the axial plane; for MRI the plane of acquisition may be axial, saggital or coronal). The minimum value is taken as the short diameter. For example, an abdominal node

which is reported as being 20mm×30mm has a short axis of 20mm and qualifies as a malignant, measurable node. In this example, 20 mm is the measured value of the module. All other pathological nodes (those with short axis ≥10mm but <15 mm) should be considered non-target lesions. Nodes that have a short axis <10mm are considered non-pathological and should not be recorded or followed.

A sum of the diameters (longest for non-nodal lesions, short axis for nodal lesions) for all target lesions will be calculated and reported as the baseline sum diameters. If the lymph node diameter is included, as mentioned above, only the short diameter is counted. The sum of baseline diameters will be used as a reference value for the baseline level of disease.

All other lesions, including pathological lymph nodes, can be regarded as non-target lesions and do not need to be measured, but should be recorded during the baseline assessment. Measurements are not required and these lesions should be followed as 'present', 'absent', or in rare cases 'unequivocal progression'. In addition, it is possible to record multiple target lesions involving the same organ as a single item (e.g. 'multiple enlarged pelvic lymph nodes' or 'multiple liver metastases').

4.3 Response criteria

4.3.1 Target lesion assessment

Complete response (CR): Disappearance of all target lesions. Any pathological lymph nodes (whether target or non-target) must have short axis <10 mm.

Partial remission (PR): The sum of the diameters of the target lesions is reduced by at least 30% from the baseline level.

Progressive Disease (PD): At least a 20% increase in the sum of diameters of target lesions, taking as reference the smallest sum on study (this includes the baseline sum if that is the smallest on study). In addition to the relative increase of 20%, the sum must also demonstrate an absolute increase of at least 5 mm. (the appearance of one or more new lesions is also considered progression).

Stable disease (SD): The degree of reduction of the target lesion did not reach the PR, and the degree of increase did not reach the PD level, between the two. The minimum sum of the diameters can be used as a reference during the study.

4.3.2 Special notes on target lesion assessment

Lymph nodes: Even if the lymph nodes identified as target lesions are reduced to less than 10 mm, the actual short diameter value corresponding to the baseline must be recorded for each measurement (consistent with the anatomical plane of the baseline measurement). This means that when lymph nodes are included as target lesions, the 'sum' of lesions may not be zero even if complete response criteria are met, since a normal lymph node is defined as having a short axis of <10 mm. Case report forms or other data collection methods may therefore be designed to have target nodal lesions recorded in a separate section where, in order to qualify for CR, each node must achieve a short axis <10 mm. For PR, SD and PD, the actual short axis measurement of the nodes is to be included in the sum of target lesions.

Target lesions that are too small to measure: While on study, all lesions (nodal and non-nodal) recorded at baseline should have their actual measurements recorded at each subsequent evaluation, even when very small (e.g. 2 mm). Sometimes, however, it may be too small, resulting in the images becoming so faint on CT scan. The radiologist may not feel comfortable assigning an exact measure and may report them as being 'too small to measure'. When this occurs it is important that a value be recorded on the case report form. If it is the opinion of the

radiologist that the lesion has likely disappeared, the measurement should be recorded as 0 mm. If the lesion is believed to be present and is faintly seen but too small to measure, a default value of 5 mm should be assigned. (Note: Lymph nodes are unlikely to have this condition, because they generally have a measurable size under normal conditions, or are often surrounded by fatty tissue as they are in the retroperitoneal cavity; However, if this kind of measurement value cannot be given, the default value is 5 mm). This default value is derived from the 5 mm CT slice thickness (but should not be changed with varying CT slice thickness). Since there is little chance that the same measurement value will recur, providing this default value will reduce the risk of erroneous evaluation. To reiterate, however, if the radiologist is able to provide an actual measure, that should be recorded, even if it is below 5 mm.

Separate or combined lesions: When non-nodular lesions are divided into fragments, add the longest diameters of the separated parts to calculate the sum of the diameters of the lesions. Similarly, for combined lesions, the planes between the combined parts can be distinguished, and then the maximum diameter of each can be calculated. However, if the combination is inseparable, the longest diameter should be the longest diameter of the entire fusion lesion.

4.3.3 Assessment of non-Target lesions

This section defines the criteria for tumor response in non-target lesions. While some non-target lesions may actually be measurable, they need not be measured and only qualitatively assessed at the time points specified in the protocol.

Complete Response (CR): Disappearance of all non-target lesions and normalisation of tumor marker level. All lymph nodes must be non-pathological in size (<10mm short axis).

Non-CR/Non-PD: Persistence of one or more non-target lesion(s) and/or maintenance of tumor marker level above the normal limits.

Progressive disease: Unequivocal progression of existing non-target lesions. Note: The appearance of one or more new lesions is also considered progression.

4.3.4 Special notes on assessment of progression of non-target disease

The concept of progression of non-target disease requires additional explanation as follows: When the patient also has measurable disease, to achieve ‘unequivocal progression’ on the basis of the non-target disease, there must be an overall level of substantial worsening in non-target disease such that, even in presence of SD or PR in target disease, the overall tumor burden has increased sufficiently to merit discontinuation of therapy. A modest 'increase' in the size of one or more non-target lesions is usually not sufficient to qualify for progression; therefore, it may be rare for changes in non-target lesions alone to define overall tumor progression in the face of SD or PR of target lesions.

When the patient has only non-measurable non-target disease: This circumstance arises in some phase III trials when it is not a criterion of study entry to have measurable disease. The overall assessment still refers to the above criteria, but because there is no measurable data of the lesion in this case. The deterioration of non-target lesions is not easy to evaluate (by definition: all non-target lesions must be truly unmeasurable). Therefore, when changes in non-target lesions lead to an increase in the overall disease burden that is equivalent to disease progression in the target lesions, a clear definition of progression based on non-target lesions requires the establishment of an effective detection method for evaluation. As described, an increase in tumor burden is equivalent to an additional 73% increase in volume (equivalent to a 20% increase in the diameter of a measurable lesion). Examples include an increase in a pleural effusion from 'trace' to 'large', an increase in lymphangitic disease from 'topical' to 'widespread', or may be described in protocols as 'sufficient to require a change in therapy'. Examples include pleural

effusions ranging from trace to large, lymphatic involvement spreading from the primary site to distant, or may be described in the protocol as 'necessitating a change in therapy'. If clear progression is found, the patient should be regarded as disease progression overall at that point in time. While it is preferable to have objective criteria to be applied in the assessment of non-measurable disease, increased criteria must be reliable.

4.3.5 New lesions

The appearance of new malignant lesions denotes disease progression; therefore, some comments on new lesions are important. There are currently no specific standards for imaging detection of lesions, but the discovery of a new lesion should be clear. I.e. not attributable to differences in scanning technique, change in imaging modality or findings thought to represent something other than tumor (for example, some 'new' bone lesions may be simply healing or flare of pre-existing lesions). This is important when the subject's baseline lesions show partial or complete response. For example, necrosis of a liver lesion may be reported on a CT scan report as a new cystic lesion, which it is not.

The lesions that have been detected during follow-up but not found in the baseline examination will be regarded as new lesions and indicate disease progression. An example of this is the subject who has visceral disease at baseline and while on study has a CT or MRI brain scan which reveals metastases. The subject's brain metastases are considered to be evidence of PD even if he/she did not have brain imaging at baseline.

If a new lesion is equivocal, for example because of its small size, continued therapy and follow-up evaluation will clarify if it represents truly new disease. If repeat scans confirm there is definitely a new lesion, then time for progression should be the date of the initial scan.

While FDG-PET response assessments need additional testing for confirmation, it is sometimes reasonable to incorporate the use of FDG-PET scanning and complement CT scanning in assessment of progression (particularly possible 'new' disease). New lesions via FDG-PET can be identified according to the following procedure:

- A negative FDG-PET at baseline and a positive FDG-PET at follow-up is a sign of PD.
- No FDG-PET at baseline and a positive FDG-PET result at follow-up:
- If the positive FDG-PET at follow-up corresponds to a new site of disease confirmed by CT, this is PD.
- If the positive FDG-PET at follow-up is not confirmed as a new site of disease on CT, additional CT scans are needed for confirmation (if so, the date of PD will be the date of the initial abnormal FDG-PET scan).
- If the positive result of the follow-up FDG-PET examination is consistent with the existing lesion by CT examination, and the lesion does not progress on imaging tests, then the disease has not progressed.

4.4 Evaluation of Best Overall Response

The best overall response is the best response recorded from the start of the study treatment until the end of treatment taking into account any requirement for confirmation. On occasion a response may not be documented until after the end of therapy so protocols should be clear if post-treatment assessments are to be considered in determination of best overall response. The plan must specify how any new treatments before progression affect the best response. The patient’s best overall response assignment will depend on the findings of both target and non-target disease and will also take into consideration the appearance of new lesions. In addition, it also depends on the nature of the experiment, program requirements, and results

measurement standards. Specifically, in non-randomised trials where response is the primary endpoint, confirmation of PR or CR is needed to deem either one the 'best overall response'.

4.4.1 Time Point Response

It is assumed that at each protocol-specified time point a response occurs. Table 1 provides a summary of overall response at each time point for subjects who have measurable disease at baseline.

Table 1 Response of time point: subjects with target lesion (including or excluding non-target lesion)

Target lesions	Non-target lesions	New lesions	Overall response
CR	CR	Non	CR
CR	Non-CR/non-PD	Non	PR
CR	Not evaluable	Non	PR
PR	Non-progressive or not fully evaluated	Non	PR
SD	Non-progressive or not fully evaluated	Non	SD
Not fully evaluated	Non-progressive	Non	NE
PD	Any condition	Yes or No	PD
Any condition	PD	Yes or No	PD
Any condition	Any condition	Yes	PD

CR = complete response, PR = partial response, SD = stable disease, PD = progressive disease, NE = not evaluable

If the subject has no measurable disease (no target disease), Table 2 is to be used for assessment.

Table 2 Time point response: subjects with non-target disease only

Non-target lesions	New lesions	Overall response
CR	Non	CR
Non-CR or Non-PD	Non	Non-CR or non-PDa
Not fully evaluated	Non	Not evaluable
Unequivocal PD	Yes or No	PD
Any condition	Yes	PD

A: 'Non-CR/non-PD' is superior to 'stable disease' for non-target lesions. Since SD is increasingly used as endpoint for assessment of efficacy in some trials so to assign this category when no lesions can be measured is not advised.

4.4.2 Missing assessments and non-evaluable designation

If no imaging/measurement can be done at a particular time point, the subject is not evaluable (NE) at that time point. If only partial lesion measurements are made at an assessment, usually the case is also considered NE at that time point, unless a convincing argument can be made that the contribution of the individual missing lesion (s) would not change the assigned time point response. This is likely to occur in the disease progression. For example, if a patient had a baseline sum of 50mm with three measured lesions and at follow-up only two lesions were assessed, but those gave a sum of 80mm, the patient will have achieved PD status, regardless of the contribution of the mission lesion.

4.4.3 Best overall response: all time points

The best overall response is determined once all the data for the subject is known.

Evaluation of the best overall response when the study does not require confirmation of the complete or partial response: The best response in the trial is the best response at all time points (for example: a patient is evaluated as SD in the first cycle, PR in the second cycle, and PD in the last cycle, but the best overall response is evaluated as PR. When the best total remission evaluation is SD, it must meet the minimum time from the baseline level specified by the plan. If the standard for the shortest time is not met, even the best overall response evaluation is not recognized as SD, and the patient's best overall response will be determined by subsequent evaluations. For example: a patient is evaluated as SD in the first cycle and PD in the second cycle, but it does not meet the minimum time requirement for SD, and the best overall response is evaluated as PD. The same patients who are lost to follow-up after being evaluated as SD in the first cycle will be considered unevaluable.

Best response determination in trials where confirmation of complete or partial response is required: Complete or partial responses may be claimed only when the criteria for each are met at a subsequent time point as specified in the protocol (generally 4 weeks later). In this case, please refer to Table 3 for the description of the best overall response.

Table 3 Best overall response when confirmation of CR and PR required

Overall response at first time point	Overall response at later time points	Best overall response
CR	CR	CR
CR	PR	SD, PD or PR[a]
CR	SD	SD provided that the minimum criteria for SD duration is met, otherwise PD
CR	PD	SD provided that the minimum criteria for SD duration is met, otherwise PD
CR	NE	SD provided that the minimum criteria for SD duration is met, otherwise NE
PR	CR	PR
PR	PR	PR
PR	SD	SD
PR	PD	SD provided that the minimum criteria for SD duration is met, otherwise PD
PR	NE	SD provided that the minimum criteria for SD duration is met, otherwise NE
NE	NE	NE

CR = complete response, PR = partial response, SD = stable disease, PD = progressive disease, NE = not evaluable.

A: If a CR is truly met at first time point, then any disease seen at a subsequent time point, even disease meeting PR criteria relative to baseline, will have a efficacy evaluation as PD at that point (since the disease have reappeared after CR). The best response depends on whether SD occurs at the shortest treatment interval. However, sometimes' CR 'may be claimed first while subsequent scans suggest small lesions are likely still present. Hence, in fact the subject has PR, not CR at the first time point. In this case, the original CR should be changed to PR and the best response is PR.

4.4.4 Special notes on response assessment

When nodular lesions are included in the overall target lesion evaluation and the nodule size is reduced to a "normal" size (< 10 mm), there is still a lesion size scan report. To avoid overestimation the situation on the increase in the size of the nodule, the measurement result should be recorded even if the nodule is normal. As mentioned earlier, this means that subjects with CR will not have a total sum of 'zero’ in the CRF.

If efficacy confirmation is required during the trial, repeated 'unmeasurable' time points will complicate the best efficacy evaluation. The analysis plan of the trial must state that these missing data/assessments can be explained clearly when determining efficacy. For example, in most trials, the response of a subject’s PR-NE-PR can be regarded as a confirmation of efficacy.

That subjects with a systematic deterioration of health status require discontinuation of treatment without objective evidence of disease progression should be reported as symptomatic progression. Every effort should be made to assess objective progression even after treatment discontinuation. Assessment description on symptomatic deterioration is not an objective response: it is the reason for stopping treatment. The objective response status of such subjects will be determined by evaluation of target and non-target lesions as shown in Tables 1 – 3.

Defined as early progression, early death and non-evaluable conditions are special cases of the study and should be clearly described in each protocol (depending on the treatment interval and treatment period).

In some cases, it is difficult to distinguish local lesions from normal tissues. When the evaluation of complete response is conducted under this situation, it is recommended that to perform biopsy before assigning a status of complete response to the focal lesion. When the abnormal imaging test results of some subjects' focal lesions are considered to represent fibrosis or scar formation, FDG-PET is used as an evaluation standard similar to biopsy to confirm the efficacy of complete response. In this case, the application of FDG-PET should be described prospectively in the plan, and the report of the specialist medical literature for this situation should be used as support. However, it must be acknowledged that both approaches may lead to false positive CR due to limitations of FDG-PET and biopsy resolution/sensitivity.

For equivocal findings of progression (e.g., very small and uncertain new lesions; cystic changes or necrosis in existing lesions), treatment may continue until the next scheduled assessment. If at the next scheduled assessment, progression is confirmed, the date of progression should be the earlier date when progression was suspected.

4.5 Frequency of Tumor Re-evaluation

The frequency of tumor re-evaluation during treatment depends on the treatment regimen and should be consistent with the type and schedule of treatment. However, in the context of phase II studies where the beneficial effect of therapy is not known, follow-up every 6–8 weeks (timed to coincide with the end of a cycle) is reasonable. Smaller or greater time intervals than these could be justified in specific regimens or circumstances. The protocol should specify which tissue sites are to be assessed at baseline (usually sites most likely to be involved with metastatic disease for the tumor type under study) and how often assessments are repeated. Normally, target and non-target lesions are evaluated at each assessment. In selected circumstances, certain non-target lesions may be evaluated less frequently. For example, bone scans may need to be repeated only when CR is identified in target disease or when progression in bone is suspected.

After the end of treatment, re-evaluation of tumors depends on whether the response rate or the time to an event (progression/death) is used as the clinical trial endpoint. Eg. time to an event (e.g. TTP/DFS/PFS) requires routine repeat evaluation as specified in the protocol. In randomized comparative trials in particular, the scheduled assessments should be performed as outlined in the schedule (eg, 6 to 8 weeks on treatment or 3 to 4 months after treatment) and should not be affected by delays in therapy, dosing intervals, or any other events that might lead to imbalances in treatment arms in the timing of disease assessments.

4.6 Confirmation of response assessment/duration of response

4.6.1 Qualification

In non-randomized trials where response is the primary endpoint, confirmation of PR and CR is required to ensure responses identified are not the result of measurement error. This also allows reasonable interpretation of the results when historical data is available, but the efficacy of the historical data of these trials should also be confirmed. However, in all other circumstances, i.e., in randomized trials (Phase II or III) or studies where stable disease or progression are the primary endpoints, confirmation of response is not required since it will not add value to the interpretation of the trial results. However, the removal of the requirement for efficacy confirmation will make the central review to prevent the effect of offset even more important, especially in non-blinded experimental studies.

In the case of SD, within the shortest time interval after the start of the trial (generally no less than 6 to 8 weeks), at least one measurement meets the SD standard specified in the protocol.

4.6.2 Duration of overall response

Overall response period is measured from the time when measurement criterias for CR/PR are first met (whichever is first recorded) to the first date when relapse or progressive disease is objectively documented (taking as reference the smallest measurements for progressive disease recorded on study). The duration of overall complete response is measured from the time when measurement criteria for CR are first met to the first date when relapse or progressive disease is objectively documented.

4.6.3 Stable disease

Stable disease is measured from the start of the treatment (in randomized trials, from date of randomization) to the time when the criteria for progression are met, taking as reference the smallest sum on study (if the baseline sum is the smallest, this is the reference for calculation of PD). The clinical relevance of stable disease varies with different studies and different diseases. If the proportion of subjects achieving stable disease for a minimum period of time is an endpoint of importance in a particular trial, the protocol should specify the minimum time interval required between two measurements for determination of SD.

Note: The duration of response and stable disease as well as the progression-free survival are influenced by the frequency of follow-up after baseline evaluation. It is not within the scope of this guideline to define a standard follow-up frequency. The frequency of follow-up should take into account many factors, such as disease type and stage, treatment cycle and standard practice. However, these limitations in the accuracy of the measured endpoints should be taken into account if comparisons between trials are needed.

4.7 PFS/TTP

4.7.1 Phase 2 clinical trial

This guideline is focused primarily on the application of objective response as endpoints in phase 2 trials. In some cases, response rate may not be optimal for evaluating the potential anticancer activity of new drugs/regimens. In these cases, PFS/TTP at demarcated time points may be considered a suitable surrogate to provide an initial signal of biologic activity of a new drug. However, it is clear that in an uncontrolled trial, these assessments can be questioned because an apparently valuable observation may be related to biological factors such as subject screening rather than the effect of the drug intervention. Thus, phase II screening trials utilizing these endpoints are best designed with a randomized control. However, since certain tumours behave consistently (and often consistently in poor state), non-randomised trials are justified to some

extent. However, in these cases, due to the lack of an active control, careful attention should be paid to document evidence of efficacy when assessing expected PFS or TTP.

Annex 5 Prohibited TCM Preparations during the Study

TCM preparations that are not allowed during the study include but are not limited to:

- ✓ Huatan Huisheng Tablets
- ✓ Brucea javanica oil soft capsule
- ✓ Zhe Mu Syrup
- ✓ Cantharidin
- ✓ Cinobufagin
- ✓ Toad venom
- ✓ Kangai Injection
- ✓ Kanglaite
- ✓ Zhongjiefeng Injection
- ✓ Aidi Injection
- ✓ Awei Huapi Plaster
- ✓ Kangaiping Wan
- ✓ Fukang Capsules
- ✓ XiaoAiPing
- ✓ Pingxiao Capsules
- ✓ Pingxiao Tablet
- ✓ Shendansanjie Capsule
- ✓ Ankangxin Capsules
- ✓ Bosheng Aining
- ✓ Zedoary Turmeric Oil and Glucose Injection
- ✓ Kanglixin Capsules
- ✓ Cidan Capsules

Annex6 Bone marrow content (%) of human skeleton

Percent Bone Marrow in the Adult Skeleton

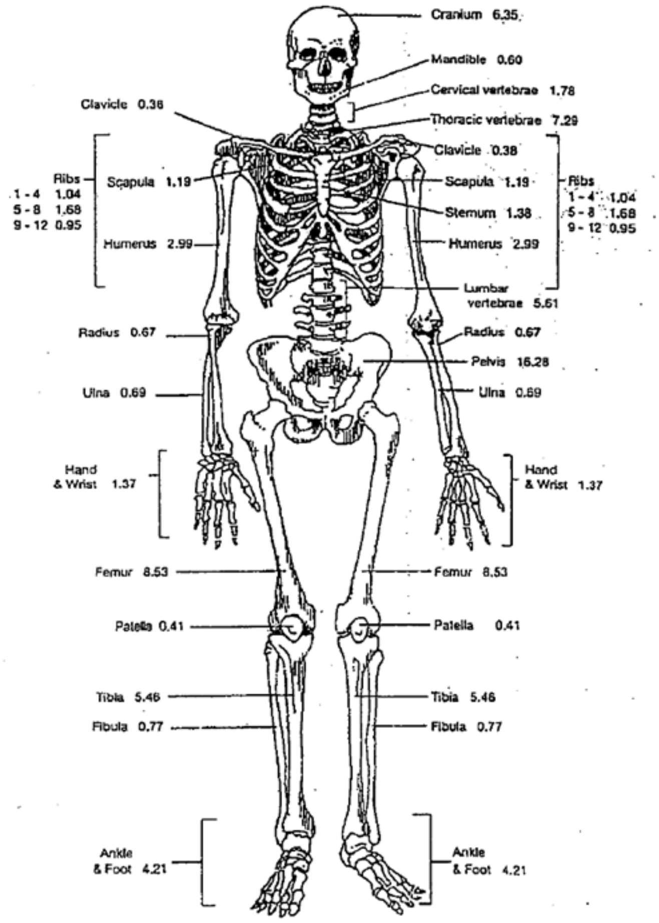

Woodward Holoday E. A summary of the data of Mechanik on the distribution of human bone marrow. Phys Med Biol. 1960;5:57-59
