# Supplementary material for: A phase 1b/2 study of first-line anti-PD-L1/ TGF-βRII fusion protein SHR-1701 combined with nab-paclitaxel and gemcitabine for advanced pancreatic ductal adenocarcinoma
Source: Signal Transduct Target Ther. 2025 Dec 20;10:415. doi: 10.1038/s41392-025-02530-2 (PMC12718313; doi:10.1038/s41392-025-02530-2)
Supplement: Supplementary file 3 — Statistical Analysis Plan [file 41392_2025_2530_MOESM3_ESM.pdf]

A Phase Ib/II Clinical Study to Evaluate PD-L1/TGF-βRII Antibody (SHR-1701) Combined with Gemcitabine and Albumin-bound Paclitaxel in First-line Treatment of Advanced/Metastatic Pancreatic Cancer

Statistical Analysis Plan

Version No.: 1.0

Finalized on: 07-Apr-2023

Author: Wu Yiwen

Affiliation: Jiangsu Hengrui Pharmaceuticals Co., Ltd.

This statistical analysis plan (SAP) has been reviewed by the following functional personnel before finalization

Function	Reviewed by/Title	Working E-mail
Medicine	Wang Linna / Medical Vice General Manager	linna.wang@hengrui.com
Clinical Pharmacology	Zhang Yanyan / Assistant Director	Yanyan.zhang@hengrui.com
Translational Medicine	Gao Shanshan / Manager	shanshan.gao.sg1@hengrui.com
Statistics	Chen Chunxia / Clinical Statistics Director	Chunxia.chen@hengrui.com
Programming	Li Xiaojuan / Manager	Xiaojuan.li@hengrui.com

CONFIDENTIALITY STATEMENT

The information contained in this document are confidential and is owned by Jiangsu Hengrui Pharmaceuticals Co., Ltd. or its subsidiaries, and should not be disclosed. It must not be reproduced or distributed to any person unless he/she has signed a non-disclosure agreement with Jiangsu Hengrui Pharmaceuticals Co., Ltd. or its subsidiaries.

TABLE OF CONTENTS

LIST OF ABBREVIATIONS 5
1. VERSION REVISION 6
2. STUDY OVERVIEW 6
2.1 Study Background 6
2.2 Study Objectives and Endpoints 7
2.3 Study Design 9
2.4 Randomization and Blinding 11
2.5 Study Sample Size 11
3. HYPOTHESIS TESTING AND DECISION RULES 11
3.1 Statistical Hypotheses 11
3.2 Decision Rules 11
4. INTERIM ANALYSIS 12
5. ANALYSIS SETS 12
5.1 Full Analysis Set 12
5.2 Safety Set 12
5.3 DLT Set 12
5.4 Per Protocol Set 13
5.5 Pharmacokinetic Set 13
5.6 Efficacy-evaluable Set 13
5.7 Immunogenicity Set 13
5.8 Biomarker Set 13
6. OUTCOME VARIABLES AND COVARIATES 13
6.1 Safety Endpoints 13
6.1.1 Drug Exposure 13
6.1.2 Adverse Events 15
6.1.3 Physical Examinations 15
6.1.4 Laboratory Tests 16
6.1.5 Vital Signs 16
6.1.6 12-Lead Electrocardiogram 16
6.1.7 Other Safety Endpoints 16
6.2 Pharmacokinetic Endpoints 16
6.3 Pharmacodynamic Endpoints 16
6.4 Immunogenicity Endpoints 16
6.5 Efficacy Endpoints 18
6.6 Biomarker Endpoints 19
6.7 Covariates and Subgroups 20
7. DATA PROCESSING 20

7.1 Safety Data ... 20
7.2 PK Concentrations ... 21
7.2.1 Below Limit of Quantification ... 21
7.2.2 Deviation, Missing and Outlier Values ... 21
7.3 PK Parameters ... 21
7.4 PD Data ... 21
7.5 Efficacy Data ... 21
7.6 Biomarker Data ... 23
7.7 Immunogenicity Data ... 23
7.8 Missing Data ... 23
7.8.1 Last Survival Date ... 23
7.8.2 Other Dates ... 24
8. STATISTICAL METHODS AND STATISTICAL ANALYSES ... 24
8.1 Statistical Methods ... 24
8.2 Statistical Analyses ... 25
8.2.1 Description of Subjects ... 25
8.2.1.1 Subject enrollment and disposition ... 25
8.2.1.2 Demographic and baseline characteristics ... 26
8.2.1.3 Tumor diagnosis ... 26
8.2.1.4 Past medical history ... 26
8.2.1.5 Prior and concomitant medications ... 26
8.2.1.6 Subsequent anticancer therapy ... 27
8.2.1.7 Protocol deviations ... 27
8.2.2 Safety Analysis ... 27
8.2.2.1 DLTs ... 27
8.2.2.2 Adverse events ... 27
8.2.2.3 Laboratory tests ... 28
8.2.2.4 Vital signs ... 28
8.2.2.5 12-lead electrocardiogram ... 29
8.2.2.6 Physical examinations ... 29
8.2.2.7 Other safety endpoints ... 29
8.2.3 Drug Exposure ... 29
8.2.4 Pharmacokinetic Analysis ... 29
8.2.5 Pharmacodynamic Analysis ... 30
8.2.6 Efficacy Analysis ... 30
8.2.6.1 Objective response rate ... 30
8.2.6.2 Disease control rate ... 30
8.2.6.3 Best overall response ... 30
8.2.6.4 Progression free survival ... 31
8.2.6.5 Time to progression ... 31
8.2.6.6 Duration of response ... 31

8.2.6.7 OS rate at 6/9/12-month 31
8.2.6.8 Overall survival 31
8.2.7 Immunogenicity Analysis 32
8.2.8 Exploratory Analysis 33
9. REFERENCES 33
10. APPENDICES 34
10.1 Classification Criteria for Electrocardiogram 34

LIST OF ABBREVIATIONS

Abbreviation	Full Name
ADA	Anti-Drug Antibody
AE	Adverse Event
BOR	Best Overall Response
BLQ	Below Limit of Quantification
IMAE	Immune Mediated Adverse Event
irAE	Immune-related Adverse Event
LLQ	Lower Limit of Quantification
CV	Coefficient of Variation
CR	Complete Response
DCR	Disease Control Rate
dMMR	Deficient Mismatch Repair
DLT	Dose Limiting Toxicity
DoR	Duration of Response
ECG	Electrocardiogram
ECOG	Eastern Cooperative Oncology Group
MedDRA	Medical Dictionary for Regulatory Activities
MSI-H	Microsatellite Instability-High
MTD	Maximum Tolerated Dose
NAb	Neutralizing Antibody
ORR	Objective Response Rate
OS	Overall Survival
PD	Progression Disease
PD-1	Programmed Death Receptor 1
PD-L1	Programmed Death Receptor-Ligand 1
PFS	Progression Free Survival
PK	Pharmacokinetics
PR	Pulse Rate
PR	Partial Response
RP2D	Recommended Phase II Dose
Q3W	Every 3 weeks
QT	Ventricular contraction time
QTc	QT interval corrected
QTcF	QT interval corrected using the Fridericia formula
SAE	Serious Adverse Event
SD	Stable Disease
SS	Safety Set
SOC	System Organ Class
TEAE	Treatment Emergent Adverse Event
TTP	Time to Progression

1. VERSION REVISION

This version of Statistical Analysis Plan was prepared according to the Study Protocol version 1.0 (dated 26-May-2020). This version is the initial version and has no revision history at the moment.

2. STUDY OVERVIEW

2.1 Study Background

Pancreatic cancer is a common clinical malignant tumor of digestive system, which has been showing an upward trend globally.

At present, FOLFIRINOX (fluorouracil + calcium folinate + irinotecan + oxaliplatin) regimen is the first-line chemotherapy regimen recommended by NCCN Guidelines. However, FOLFIRINOX is a combination of four chemotherapy drugs with significant clinical toxicity and side effects. It is indicated only for treatment of patients with good performance status as defined by ECOG score of 0–1, and its application is very restricted in Chinese patients. Gemcitabine plus albumin-bound paclitaxel regimen (AG regimen) is another category IA first-line standard chemotherapy regimen recommended by NCCN Guidelines. This combination therapy has better drug safety and patient tolerance than the FOLFIRINOX regimen, and its efficacy and safety have been demonstrated in Chinese patients. Therefore, this regimen is widely used in clinical practice. At present, NCCN Guidelines recommend pembrolizumab, as a category 2 second-line treatment for patients with MSI-H or dMMR advanced/metastatic pancreatic cancer, but pancreatic cancer usually has a low tumor mutation burden and a prevalence rate of dMMR less than 1%. As a result, the combination therapy of immune checkpoint inhibitors and chemotherapy has become a new direction of exploration, the theoretical basis of which primarily lies in the immunomodulatory effects of cytotoxic chemotherapy drugs such as gemcitabine and paclitaxel. Chemotherapy drugs not only kill tumor cells, but also increase the exposure and presentation of tumor antigens, and enhance the activity of immune killer cells and T cells, thereby achieving the goal of synergistic effect.

In the tumor microenvironment, the PD-L1/PD-1 pathway and TGF-β signaling pathway play a crucial role in tumor immune escape. PD-L1 is mainly expressed on T cells, B cells, macrophages and dendritic cells (DCs), and can be up-regulated in activated cells. PD-L1 expressed on the surface of tumor cells binds to PD-1 on the surface of tumor infiltrating lymphocytes and inhibits lymphocyte function, which is one of the important reasons for immune escape in tumors. TGF-β is a dimer composed of two structurally identical or similar

subunits with a molecular weight of 12.5 kDa, linked by a disulfide bond. The TGF-β signaling pathway has a dual nature in the process of tumor occurrence and development: it inhibits tumor growth in the early stages and promotes tumor growth in the late stages. TGF-β can up-regulate PD-L1 on antigen-presenting cells (APCs) which can release soluble PD-L1 into the tumor microenvironment, directly inhibiting cytotoxic T lymphocytes through the interaction between PD-L1 and its receptors, or inducing cytotoxic T lymphocytes to release inhibitory soluble molecules. Therefore, on the basis of inhibiting the PD-1/PD-L1 pathway, targeted neutralization of TGF-β in the tumor microenvironment can restore the activity of T cells, enhance immune response, and more effectively improve the effect of inhibiting tumor occurrence and development, becoming a new approach for tumor treatment.

Bifunctional fusion protein refers to a fusion protein containing two functional domains. Due to its specificity and dual functionality, it has become a research hotspot in the field of bioengineered therapeutic drugs and has broad application prospects in tumor immunotherapy and autoimmune diseases.

SHR-1701 is an anti-PD-L1/TGF-β RII bifunctional fusion protein developed by Hengrui and molecularly includes two parts: anti-PD-L1 monoclonal antibody and TGF-β RII extracellular domain truncated form. Specifically, it is composed of anti-PD-L1 antibody linked at the C-terminal amino acid of its heavy chain to TGF-β RII at the N-terminal amino acid of the extracellular domain through a linker protein fragment (G4S) 4G. SHR-1701 can block the PD-1/PD-L1 pathway and neutralize TGF-β in the tumor microenvironment. The combined inhibition of PD-1 and TGF-β negative signals can lead to a more effective anti-tumor immune response than inhibition of either pathway alone, thereby achieving the goal of improving anti-tumor efficacy.

2.2 Study Objectives and Endpoints

Primary study objectives

Primary study objectives of the Phase Ib study:

- To evaluate the safety and tolerability of SHR-1701 combined with gemcitabine and albumin-bound paclitaxel in first-line treatment of patients with advanced/metastatic pancreatic cancer, and determine the recommended Phase II dose of SHR-1701 in the combined regimen.
- To evaluate the efficacy of SHR-1701 combined with gemcitabine and albumin-bound paclitaxel in first-line treatment of patients with advanced/metastatic pancreatic cancer, as measured by objective response rate (ORR).

Primary study objective of the Phase II study:

- To evaluate the efficacy of SHR-1701 combined with gemcitabine and albumin-bound paclitaxel in first-line treatment of patients with advanced/metastatic pancreatic cancer, as measured by objective response rate (ORR).

Secondary study objectives

Secondary study objectives of the Phase Ib study:

- To preliminarily evaluate the efficacy of SHR-1701 combined with gemcitabine and albumin-bound paclitaxel in first-line treatment of advanced/metastatic pancreatic cancer.
- To evaluate the pharmacokinetic (PK) profile of SHR-1701.

Secondary study objectives of the Phase II study:

- Key secondary study objective: To evaluate the efficacy of SHR-1701 combined with gemcitabine and albumin-bound paclitaxel in first-line treatment of patients with advanced/metastatic pancreatic cancer, as measured by OS rate at 9-month.
- To evaluate the efficacy of SHR-1701 combined with gemcitabine and albumin-bound paclitaxel in first-line treatment of patients with advanced/metastatic pancreatic cancer, as measured by other efficacy variables.
- To further evaluate the safety and tolerability of SHR-1701 combined with gemcitabine and albumin-bound paclitaxel in first-line treatment of patients with advanced/metastatic pancreatic cancer.
- To evaluate the pharmacokinetic (PK) profile of SHR-1701.

Exploratory study objectives

- To evaluate the immunogenicity of SHR-1701.
- To collect tumor tissue samples for biomarker analysis in order to explore the relationship between biomarkers and clinical efficacy, including the expression of PD-L1 in tumor tissue, tumor infiltrating lymphocytes, levels of TGFβ/pSmad pathway-associated proteins, and gene expression in the tumor microenvironment.

Primary study endpoints

Primary study endpoint of the Phase Ib study:

- Recommended Phase II dose (RP2D) of SHR-1701 in the combined regimen.

Primary study endpoint of the Phase II study:

- Objective response rate (ORR) assessed by the investigator per RECIST v1.1 criteria.

Secondary study endpoints

Secondary study endpoints of the Phase Ib study:

- Incidence of dose limiting toxicity (DLT), incidence and severity of adverse events (AEs) and serious adverse events (SAEs) assessed as per NCI-CTCAE 5.0, as well as safety variables such as vital signs, electrocardiograms, and abnormal laboratory tests;
- Preliminary efficacy endpoints: Efficacy variables assessed by the investigator per RECIST v1.1 criteria, including objective response rate (ORR), disease control rate (DCR), best overall response (BOR), progression free survival (PFS), time to progression (TTP), duration of response (DoR), overall survival (OS) rate at 6-month, OS rate at 9-month, OS rate at 12-month, and OS;
- Pharmacokinetic parameters of SHR-1701, including C_{trough} and C_{max}.

Secondary study endpoints of the Phase II study:

- Key secondary study endpoint: OS rate at 9-month;
- Other secondary efficacy endpoints: Efficacy variables assessed by the investigator per RECIST v1.1 criteria, including disease control rate (DCR), best overall response (BOR), progression free survival (PFS), time to progression (TTP), duration of response (DoR), OS rate at 6-month, OS rate at 12-month, and OS;
- Pharmacokinetic parameters of SHR-1701, including C_{trough} and C_{max}.

Exploratory study endpoints of Phase Ib and II studies:

- Immunogenicity of SHR-1701: The presence of anti-SHR-1701 antibodies (including ADA and NAb) formed during the study period will be analyzed compared to baseline, combined with plasma SHR-1701 concentration, safety, and efficacy data.
- The relationship between biomarkers, including expression of PD-L1 in tumor tissue, tumor infiltrating lymphocytes, levels of TGFβ/pSmad pathway-associated proteins, and gene expression in the tumor microenvironment, and clinical efficacy will be explored.

2.3 Study Design

This is a Phase Ib/II, open-label, multicenter clinical study, consisting of two phases: Phase Ib, dose-finding of combination therapy, and Phase II, efficacy expansion. A total of 54–60 patients who have not received systematic treatment for advanced/metastatic pancreatic cancer are planned to be enrolled.

Phase Ib dose-finding study

This phase is designed to observe and evaluate the safety and tolerability of SHR-1701 combined with gemcitabine and albumin-bound paclitaxel in first-line treatment of patients with advanced/metastatic pancreatic cancer, and determine the recommended Phase II dose of SHR-1701 in the combined regimen.

In this study phase, a modified “3+3” algorithm will be used for dose finding. The pre-specified dose of SHR-1701 is 30 mg/kg, D1, q3w (every 3 weeks/21 days) and 20 mg/kg, q3w. The dose level of 30 mg/kg is the recommended Phase II dose (RP2D) determined based on the Phase I clinical trial of SHR-1701 monotherapy. In this study, dose finding will start from SHR-1701 30 mg/kg, D1, q3w. If it cannot be tolerated, the dose level of SHR-1701 will be reduced to 20 mg/kg, D1, q3w to continue the dose-finding.

The 6 subjects initially enrolled will receive SHR-1701 30 mg/kg, D1, q3w plus chemotherapy (gemcitabine 1000 mg/m^2, D1 and D8, q3w; albumin-bound paclitaxel 125 mg/m^2, D1 and D8, q3w). If a DLT is observed in no more than 1 of the 6 subjects (proportion <0.33), the efficacy expansion phase (Phase II) will start at this dose level;

If a DLT is observed in 2 or more of the 6 subjects (proportion ≥0.33) at this dose level, additional 6 subjects will be enrolled to receive SHR-1701 20 mg/kg, D1, q3w plus chemotherapy (gemcitabine 1000 mg/m^2, D1 and D8, q3w; albumin-bound paclitaxel 125 mg/m^2, D1 and D8, q3w). If a DLT is still observed in 2 or more of the subjects (proportion ≥0.33), the Safety Monitoring Committee (SMC) will discuss and decide on the dosage/administration method to be used for subsequent studies.

The first cycle of combination therapy (21 days) serves as the DLT observation period, and after completing one cycle of combination therapy, subjects will undergo safety assessments during the DLT observation period.

If the dose of the investigational product received by a subject in Cycle 1 is less than 90% of the prescribed dose (e.g., dose interruption due to infusion reactions, resulting in an overall intravenous dosage lower than 90% of the prescribed dose) and no DLT is observed, the subject will not be included in the calculation of the incidence of DLTs at given dose level, and a new subject needs to be added for replacement at the current dose level.

If a subject terminates the study treatment during the DLT observation period after the first dose due to reasons other than DLTs, the subject needs to be replaced by a new subject. Increasing or reducing the dose of SHR-1701 is not allowed during the study. During the DLT observation period, it is also not allowed to adjust the dose of any combination therapy.

Before starting the study at the next dose level, all subjects (n=6) at the current dose level must complete the DLT observation period. The interval between subjects entering the study at the same dose level is determined by the SMC based on the available safety/tolerability data.

Phase II efficacy expansion study

The efficacy expansion phase study will be conducted at the recommended Phase II dose of SHR-1701 plus chemotherapy determined based on the preliminary safety, PK, and efficacy

data of Phase Ib of the study. Additional subjects will be enrolled to receive the treatment at the same dose level (n=54). The efficacy and safety will be further explored in patients with advanced/metastatic pancreatic cancer.

In this study, end of study is defined as the time when 70% of the subjects have died in the Phase II of the study or the Sponsor decides to terminate the study.

2.4 Randomization and Blinding

This is a single-arm, dose-escalation, sequential, efficacy expansion study, without control, randomization, or blinding.

2.5 Study Sample Size

Phase Ib dose-finding study

The sample size is approximately 6–12 subjects.

Phase II efficacy expansion study

At the significance level α=0.05, when the sample size is 49 subjects and the point estimate of ORR is 30%, the exact test method can provide a 95% confidence interval with a half width (precision) of 14% for the ORR in the SHR-1701 combined with gemcitabine and albumin-bound paclitaxel treatment group. Considering a dropout rate of about 10%, it is expected that 54 subjects will be enrolled.

For the key secondary endpoint (OS rate at 9-month), this sample size is also adequate to afford an 80% power to detect a 20% difference, i.e. (50% vs. 70%).

The 6 subjects enrolled in Phase I can continue to enter Phase II, so the total sample size of this study is approximately 54–60 subjects.

3. HYPOTHESIS TESTING AND DECISION RULES

3.1 Statistical Hypotheses

There is no routine statistical hypothesis.

3.2 Decision Rules

Phase Ib dose-finding study

A modified “3+3” algorithm will be used for dose finding. The pre-specified dose of SHR-1701 is 30 mg/kg, D1, q3w (every 3 weeks/21 days) and 20 mg/kg, q3w. In this study, dose finding will start from SHR-1701 30 mg/kg, D1, q3w. If it cannot be tolerated, the dose level of SHR-1701 will be reduced to 20 mg/kg, D1, q3w to continue the dose-finding.

The 6 subjects initially enrolled will receive SHR-1701 30 mg/kg, D1, q3w plus chemotherapy (gemcitabine 1000 mg/m^2, D1 and D8, q3w; albumin-bound paclitaxel 125 mg/m^2, D1 and D8, q3w). If a DLT is observed in no more than 1 of the 6 subjects (proportion <0.33), the efficacy expansion phase (Phase II) will start at this dose level;

If a DLT is observed in 2 or more of the 6 subjects (proportion ≥0.33) at this dose level, additional 6 subjects will be enrolled to receive SHR-1701 20 mg/kg, D1, q3w plus chemotherapy (gemcitabine 1000 mg/m^2, D1 and D8, q3w; albumin-bound paclitaxel 125 mg/m^2, D1 and D8, q3w). If a DLT is still observed in 2 or more of the subjects (proportion ≥0.33), the SMC will discuss and decide on the dosage/administration method to be used for subsequent studies.

Phase II efficacy expansion study

The efficacy expansion phase study will be conducted at the recommended Phase II dose of SHR-1701 plus chemotherapy determined based on the preliminary safety, PK, and efficacy data of Phase Ib of the study. Additional subjects will be enrolled to receive the treatment at the same dose level (n=54).

4. INTERIM ANALYSIS

There is no routine interim analysis in this study. Phased analyses may be conducted based on study progress and practical needs.

5. ANALYSIS SETS

5.1 Full Analysis Set

Including all the enrolled subjects who have received at least one dose of the investigational product.

5.2 Safety Set

Including all the subjects who have received at least one dose of the investigational product.

5.3 DLT Set

Including all the subjects enrolled in the dose-finding phase of the study who have received the combination therapy (the dose of any investigational drug in Cycle 1 should be no less than 90% of the prescribed dose) up to the end of Cycle 1 (a total of 21 days) or have discontinued the treatment due to DLTs observed during this period.

DLT observation period is defined as the period from the first dose to Day 21.

5.4 Per Protocol Set

A subset of the Full Analysis Set. The subjects with important protocol deviations which have important influence on efficacy will be excluded from this set. The list of subjects who are included or excluded from the Per Protocol Set needs to be determined through review by the Sponsor and investigators prior to lock of database.

5.5 Pharmacokinetic Set

Including all the enrolled subjects who have received at least one dose of the investigational product, and have postdose evaluable pharmacokinetic data.

5.6 Efficacy-evaluable Set

Including all the enrolled subjects who have received at least one dose of the investigational product, and have baseline and at least one post-baseline tumor response assessment.

5.7 Immunogenicity Set

Including all the enrolled subjects who have received at least one dose of the investigational product, and have baseline and at least one post-baseline immunogenicity assessment data.

5.8 Biomarker Set

Including all the enrolled subjects who have received the investigational product, and have at least one evaluable biomarker data.

6. OUTCOME VARIABLES AND COVARIATES

6.1 Safety Endpoints

The statistical analyses of this study will involve the following categories of safety endpoints:

- Drug exposure
- Adverse events
- Physical examinations
- Laboratory tests
- Vital signs
- 12-lead electrocardiogram
- Other safety endpoints

In addition, DLT events will be tabulated according to the DLT definition in the protocol.

6.1.1 Drug Exposure

For Cycle X, the actual start date of the cycle for each subject is:

- If the subject received the investigational product at a visit (dose administered >0 at that visit), the earliest medication date for that visit cycle on the Drug Exposure page of eCRF is the actual start date of that cycle;
- If the subject did not receive the investigational product at a visit (dose administered =0 at that visit), the earliest examination date for that visit cycle is the actual start date of that cycle; If there is no examination date for the visit cycle, the visit date of the cycle is the actual start date of the cycle.

For Cycle X, the actual end date of the cycle for each subject is:

- For all cycles X (except for the last cycle), actual end date of the cycle = actual start date of the cycle (X+1) -1 day;
- For the last cycle, the actual end date of the cycle is the actual start date of the cycle + 21 - 1 days.

For the derived variables of the investigational product SHR-1701, albumin-bound paclitaxel, and gemcitabine, see the specific definition rules in Table 1

Table 1. Derived Variables of SHR-1701, Albumin-bound Paclitaxel, and Gemcitabine

Variable Name	SHR-1701	Albumin-bound paclitaxel	Gemcitabine
Administration method[1]	30 mg/kg, intravenous infusion, every 3 weeks.	125 mg/m^2, intravenous drip, on Day 1 and Day 8 in 3-week cycles	1000 mg/m^2, intravenous drip, on Day 1 and Day 8 in 3-week cycles
Duration of exposure (weeks)	(Date of last dose – date of first dose + 21) / 7	(Date of last dose – date of first dose + n) / 7;<br>If the date of last dose is Day 1, then n=7;<br>If the date of last dose is Day 8, then n=14.	
Duration of exposure (months)	(Date of last dose – date of first dose + 21) / 30.4375	(Date of last dose – date of first dose + n) / 30.4375;<br>If the date of last dose is Day 1, then n=7;<br>If the date of last dose is Day 8, then n=14.	
Actual dose level	Actual dose level = Actual dosage / body weight = (mg/kg)	Actual dose level = Actual dosage / body surface area = (mg/m^2)	
Drug exposure (mg)	Sum of actual dosage in all cycles		
Actual cumulative dose level	Sum of actual dose levels in all cycles (SHR1701: mg/kg; albumin-bound paclitaxel: mg/m^2; gemcitabine: mg/m^2)		
Planned dose level	30 mg/kg/cycle	2*125 mg/m^2/cycle	2*1000 mg/m^2/cycle
Planned duration of dosing (weeks)	(end date – date of first dose + 1) / 7<br>End date: Start date of the last non-zero actual dosing cycle + 21 - 1		
Planned dose intensity	Planned dose level / 1 (cycle) = (mg/kg/cycle)	Planned dose level / 1 (cycle) = (mg/m^2/cycle)	Planned dose level / 1 (cycle) = (mg/m^2/cycle)

Variable Name	SHR-1701	Albumin-bound paclitaxel	Gemcitabine
Actual dose intensity	Actual cumulative dose level / (Planned duration of dosing / 3) = (mg/kg/cycle)	Actual cumulative dose level / (Planned duration of dosing / 3) = (mg/m^2/cycle)	Actual cumulative dose level / (Planned duration of dosing / 3) = (mg/m^2/cycle)
Relative dose intensity (%)	100 × Actual dose intensity / Planned dose intensity		

The recommended formula for body surface area is as follows: Body surface area (m^2) = 0.0061 × height (cm) + 0.0128 × weight (kg) - 0.1529

6.1.2 Adverse Events

For the subjects in this study, only adverse events (AEs) related to study procedures will be collected from signature of the informed consent form (ICF) to the first dose. All adverse clinical events that (newly) occur during dosing/treatment or those that occur before dosing/treatment but increase in severity after dosing/treatment are referred to as treatment emergent adverse events (TEAEs), regardless of their causal relationship with administration/treatment. TEAEs that occur during the off dosing/treatment period (e.g., washout period, dose interruption period) in the study are classified as products/therapies received prior to that off dosing/treatment period.

All AEs will be coded using MedDRA (25.0), and graded for severity based on NCI-CTCAE (V5.0).

The association between an AE and the investigational product will be assessed by the 5-point scale of “definitely related, possibly related, unlikely related, not related, and unassessable”, and AEs assessed as “definitely related, possibly related, and unassessable” are defined as treatment-related adverse events.

In the study, adverse events recorded as immune related adverse events in a case report form (CRF) are defined as immune-related adverse events (irAEs).

In the study, adverse events recorded as adverse events of special interest in CRF are defined as special interest events (SIEs).

6.1.3 Physical Examinations

Physical examination data will be collected at the time points pre-specified in the protocol. A full physical examination (including general condition, head and face, skin, lymph nodes, eyes, ears/nose/throat, mouth, respiratory system, cardiovascular system, abdomen, reproductive-urinary system, musculoskeletal system, nervous system, and mental state) will be conducted within 7 days prior to the first dose and at the end of treatment/withdrawal from the study; and

targeted physical examinations will be conducted predose on Day 1 and Day 8 of each cycle, as well as at the first visit of the safety follow-up period if clinically indicated.

6.1.4 Laboratory Tests

Laboratory tests data, including hematology, urinalysis, serum chemistry, coagulation function and thyroid function, will be collected at the time points pre-specified in the protocol. Laboratory tests data will be judged by the investigators for the presence of clinically significant abnormal findings.

6.1.5 Vital Signs

Vital signs, including blood pressure, temperature, pulse and respiratory frequency, will be collected at the time points pre-specified in the protocol.

6.1.6 12-Lead Electrocardiogram

12-lead electrocardiogram data, including heart rate, PR, QT and RR, will be collected at the time points pre-specified in the protocol. Among these, $QTcF=QT/(RR)^{1/3}$.

6.1.7 Other Safety Endpoints

Serum CA19-9, ECOG score, virological examination, fecal occult blood, blood amylase and lipase, and echocardiography will be collected at the time points pre-specified in the protocol.

6.2 Pharmacokinetic Endpoints

The PK blood samples of SHR-1701 will be collected at the time points pre-specified in the protocol.

Table 3. Names, Units, and Calculation Statements of PK Parameters

Parameter	Unit	Calculation Statement
Maximum concentration (C_{max})	ug/mL	Observed concentration (for postdose points)
Trough concentration (C_{trough})	ug/mL	Observed concentration (excluding the predose point on C1D1, and predose points in other cycles)

6.3 Pharmacodynamic Endpoints

Not applicable.

6.4 Immunogenicity Endpoints

Immunogenicity samples will be collected at the time points pre-specified in the protocol, and evaluated for immunogenicity as measured by anti-SHR-1701 antibodies (including ADA and NAb) in serum.

Subjects can be classified into the following categories based on the ADA test results:

- Subjects with baseline ADA-positive samples: defined as those who are positive for the last ADA observation prior to the first dose.
- ADA-negative subjects: ADA-negative subjects refer to subjects who are not positive for ADA samples collected after the start of treatment compared to baseline, including two categories: a. Subjects who have a baseline ADA-negative sample and are ADA negative for all post-baseline samples; b. Subjects who have a baseline ADA-positive sample and are ADA negative for all post-baseline samples or have a titer of less than 9 times the baseline level for post-baseline ADA-positive samples.
- ADA-positive subjects include two categories:
  - Treatment-boosted ADA positive: Subjects who have a baseline ADA-positive sample and at least one ADA-positive sample after baseline with a titer ≥9 times the baseline.
  - Treatment-induced ADA positive: Subjects who have a baseline ADA-negative sample and at least one ADA-positive sample after baseline.

For treatment-induced ADA positive subjects, they can be classified into transient positivity, persistent positivity, and other positive statuses based on the duration of ADA positivity.

- Transient positivity: including 2 categories: a. Subjects who have a baseline ADA-negative sample, have only one ADA-positive sample before the last post-baseline test, and are ADA negative for the last ADA test; b. Subjects who have a baseline ADA-negative sample, have an interval of <16 weeks between the first and last ADA-positive samples, and are ADA negative for the last ADA test.
- Subjects with persistent positivity: Refers to subjects who have a baseline ADA-negative sample, at least 2 ADA-positive samples after baseline, and an interval of ≥16 weeks between the first and last ADA-positive samples.
- Subjects with other positive statuses: Refers to subjects who have a baseline ADA-negative sample, are ADA positive for the last post-baseline sample, and are not those with persistent positivity.

Based on the detection results of neutralizing antibodies for different targets, subjects can be classified as follows:

- Neutralizing antibody (NAb)-positive subjects: Subjects who are negative for Nab at baseline and positive for Nab after baseline
- NAb-negative subjects: Subjects who are negative for Nab after baseline
- NAb unassessable: Others

Time to first detection of ADA positivity (days): The time to first detection of ADA positivity in treatment-induced ADA positive subjects, calculated as: (time to first detection of ADA positivity in treatment-induced ADA positive subjects - time of first dose + 1)

Duration of ADA positivity (days): The duration of ADA positivity in treatment-induced ADA positive subjects (only for subjects who are ADA negative for the last sample after baseline), calculated as: (collection time of last positive sample - collection time of first positive sample + 1).

6.5 Efficacy Endpoints

Based on the RECIST 1.1 criteria, the efficacy endpoints of the study include objective response rate (ORR), disease control rate (DCR), best overall response (BOR), progression free survival (PFS), time to progression (TTP), duration of response (DoR), OS rate at 6-month, OS rate at 9-month, OS rate at 12-month, and OS.

Objective response rate (ORR): The proportion of subjects who have received the study treatment and have a best overall response (BOR) of complete response (CR) or partial response (PR) as assessed per RECIST 1.1 criteria.

Disease control rate (DCR): The proportion of subjects who have received the study treatment and have a best overall response (BOR) of complete response (CR) or partial response (PR) or stable disease (SD) as assessed per RECIST 1.1 criteria.

Best overall response (BOR) refers to the best imaging response during the study, i.e., the best response recorded between the date of the first dose to the date of objectively documented progression in accordance with RECIST 1.1 criteria or the date of start of subsequent anticancer therapy, whichever occurs first. For subjects with no documented progression or subsequent anticancer therapy, BOR will be determined based on all response assessment results. The BOR in this study is based on confirmed response, with confirmation criteria following the RECIST v1.1 criteria. For the first achievement of CR or PR, subjects must undergo confirmation no less than 4 weeks (28 days) after the first assessment. If stable disease (SD) is achieved, the duration of SD must be no less than 6 weeks (42 days).

Progression free survival (PFS): Defined as the time from the date of the first dose to the date of first documented tumor progression as assessed per RECIST 1.1 criteria (regardless of whether the treatment is continued or not) or death for any cause, whichever occurs first.

Table 4. Censoring Rules for Progression Free Survival

#	Observation	Progression or Censoring Date
1	No appropriate baseline assessment	Censor to start date (Day 1)

#	Observation	Progression or Censoring Date
2	No appropriate post-baseline assessment, no death	Censor to start date (Day 1)
3	Any death (except for #5 and #6)	Date of death
4	PD during the study (including that after missing one assessment)	PD date (date of the earliest imaging scan during that visit)
5	Start of a new anticancer therapy and no documented PD	Censor to the date of the last imaging assessment before the use of a new anticancer therapy (if the last imaging date before the use of a new anticancer therapy is the imaging date before administration, censor to Day 1)
6	Occurrence of death or PD after 2* or more consecutive missed assessments	Censor to the date of the last imaging assessment before death or PD
7	Withdrawal from the study due to toxicity or other reasons and no documented PD	Censor to the date of the last imaging assessment before withdrawal from the study
8	No death, no PD, remain in the study	Censor to the date of the last imaging assessment before the data cutoff date

PD: Progression disease.
Two* consecutive missed assessments: Before week 48: interval between PD or death and previous tumor assessment ≥98 days; After week 48: interval between PD or death and previous tumor assessment ≥140 days; Spanning week 48: interval between PD or death and previous tumor assessment >119 days.

Time to progression (TTP): Defined as the time from the date of the first dose to the date of first documented tumor progression as assessed per RECIST 1.1 criteria (regardless of whether the treatment is continued or not).

Duration of response (DoR): Defined as the time from the date of the first documented tumor response (CR or PR as assessed per RECIST 1.1 criteria) to the date of the first documented objective tumor progression or death for any cause, whichever occurs first.

OS rate at 6/9/12-month: Defined as the probability of survival from the first dose of the investigational product to 6/9/12 months, calculated using the Kaplan-Meier method.

Overall survival (OS): Defined as the time from the date of the first dose to the date of death for any cause.

6.6 Biomarker Endpoints

Tumor tissue samples will be collected according to the protocol requirements, and tested for PD-L1 expression (CPS, TPS), tumor infiltrating lymphocytes (percentage of CD8 positive cells in immune cells, percentage of CD8 positive cells in total cells), TGFβ/pSmad pathway-associated proteins (Hscore score of pSMAD2/3 tumor cells, Hscore score of pSMAD2/3 stromal immune cells), gene expression in the tumor microenvironment, etc.

6.7 Covariates and Subgroups

Subgroups:

PD-L1 expression - category 1 (CPS): (<1, ≥1, not evaluable);

PD-L1 expression - category 2 (CPS): (<5, ≥5, not evaluable);

PD-L1 expression - category 3 (CPS): (<10, ≥10, not evaluable);

PD-L1 expression - category 4 (TPS): (<1%, 1-49%, ≥50%, not evaluable);

Maximum decrease in CA19-9 from baseline (<80%, ≥80%);

Age (<65, ≥65);

Gender, BMI (<23, ≥23);

ECOG PS score at baseline (0, 1);

Site of metastasis (liver, lung, peritoneum, lymph nodes);

Number of metastasis sites (1, 2, ≥3);

Baseline CA19-9 (≤1000, >1000);

Site of pancreatic tumor (pancreatic head, non-pancreatic head).

Prior therapies (adjuvant chemotherapy, radical surgery)

In exploratory analysis, subgroup analysis of objective response rate will be conducted and a forest plot presented. Summary of PFS and OS will be presented only for subgroups of PD-L1 expression and CA19-9, and survival curves plotted. Summary of median PFS and median OS will be presented for the following subgroups (age, gender, ECOG PS score at baseline, baseline CA19-9, site of metastasis, site of pancreatic tumor, and prior therapies).

7. DATA PROCESSING

7.1 Safety Data

Unless otherwise specified, missing safety data will not be handled.

If the test result is “< value A”, then the corresponding value in the statistical summary is “value A”. If it is “> value B”, then the corresponding value in the statistical summary is “value B”. Original values will be presented for tabulation of test results (i.e. “< value A” or “> value B”).

QTcF(ms) will be calculated according to the formula ($QTcF=QT/(RR)^{1/3}=ms/(s)^{1/3}$), rounded to integers.

7.2 PK Concentrations

7.2.1 Below Limit of Quantification

Unless otherwise specified, all BLQ PK concentrations are set as the LLQ value during statistical analysis, presented as “<LLQ” for data tabulation, and processed as missing when plotting individual drug concentration-time curves.

7.2.2 Deviation, Missing and Outlier Values

When conducting statistical analysis and plotting overall drug concentration-time curves, if any of the following situations occur, the PK concentration will be processed as missing:

- The concentration value is recorded as not measured or not collected;
- The actual PK concentration sampling time is out of window, or clinical pharmacologists believe that there is a deviation in a PK concentration. Such data will be noted with comments in the summary tabulation.

When plotting individual drug concentration-time curves or tabulating, actual collected data will be used.

7.3 PK Parameters

The study involves no calculation of PK parameters, all of which are measured values.

7.4 PD Data

Not applicable.

7.5 Efficacy Data

The time to event data among efficacy data, such as DoR and PFS, are defined as the length of time from the starting time point to the occurrence of the event of interest (progression disease, death, etc.). In studies, it is often the case that not every subject will experience an event. Due to adverse events, use of prohibited drugs, and other reasons, there are frequently subjects who withdraw from the study prematurely before an event is observed, resulting in missing time endpoints. During statistical analysis, the time to event data of subjects who prematurely withdraw from the study will be censored. The commonly used censoring technique is right censoring, where DoR or PFS of the subjects will be defined as the (censored) duration of response or progression free survival, i.e., the time from the first dose to the date of the imaging visit with tumor assessment of progression free before withdrawal from the study or use of a new anticancer therapy. The specific censoring rules can be found in the corresponding sections of each variable in Section 8.2.6.

Date of a new anticancer therapy

The start date of the new anticancer therapy (drug therapy excluding traditional Chinese medicine, surgery, and radiotherapy) is used for censoring for efficacy analysis.

If the start date of the new anticancer therapy is missing, the PD date, the date of the last dose of study treatment, and the end date of the new anticancer therapy will be used for imputation. These dates are defined as follows:

- PD date is the date of PD assessed by the investigator.
- Date of the last dose of study treatment.
- End date of the new anticancer therapy is derived as follows:

- If the end date is completely missing, disregard it;
- If only the year is known for the end date, set it to “31DECYYYY”;
- If the month and the year are known for the end date, set it as the last day of that month, expressed as “MMMYYYY”.

For the start date of a new anticancer therapy, the imputing rules are as follows:

- If the start date of a new anticancer therapy is completely missing:

Start date = the minimum of [the maximum of (PD date + 1, date of the last dose of study treatment + 1), end date of new anticancer therapy];

- If only the year is known for the start date of a new anticancer therapy:

- If the year is < the minimum of [the maximum of (PD date + 1, date of the last dose of study treatment + 1), end date of new anticancer therapy], then the start date = 31DECYYYY;
- If the year is = the minimum of [the maximum of (PD date + 1, date of the last dose of study treatment + 1), end date of new anticancer therapy], then the start date = the minimum of [the maximum of (PD date + 1, date of the last dose of study treatment + 1), end date of new anticancer therapy]
- If the year is > the minimum of [the maximum of (PD date + 1, date of the last dose of study treatment + 1), end date of new anticancer therapy], then the start date = 01JANYYYY

- If the year and the month are known for the start date of a new anticancer therapy:

- If the year is < the minimum of [the maximum of (PD date + 1, date of the last dose of study treatment + 1), end date of new anticancer therapy],

or

If the year is = the minimum of [the maximum of (PD date + 1, date of the last dose of study treatment + 1), end date of new anticancer therapy], and the month is < the minimum of [the

maximum of (PD date + 1 day, date of the last dose of study treatment + 1 day), end date of new anticancer therapy], then the start date = the last day of MMM YYYY;

- If the year is = the minimum of [the maximum of (PD date + 1, date of the last dose of study treatment + 1), end date of new anticancer therapy], and the month is = the minimum of [the maximum of (PD date + 1 day, date of the last dose of study treatment + 1 day), end date of new anticancer therapy], then start date = the minimum of [the maximum of (PD date + 1 day, date of the last dose of study treatment + 1 day), end date of new anticancer therapy];
- If the year is = the minimum of [the maximum of (PD date + 1, date of the last dose of study treatment + 1), end date of new anticancer therapy], and the month is > the minimum of [the maximum of (PD date + 1 day, date of the last dose of study treatment + 1 day), end date of new anticancer therapy]

or

If the year is > the minimum of [the maximum of (PD date + 1, date of the last dose of study treatment + 1), end date of new anticancer therapy], then the start date = 01 MMM YYYY.

7.6 Biomarker Data

Unless otherwise specified, missing biomarker data will not be handled.

When the test result of a biomarker is "< value X", set it to half of that value before analysis and present it as "< value X" for data tabulation.

7.7 Immunogenicity Data

Unless otherwise specified, missing immunogenicity data will not be handled.

When the ADA titer result is "< value X", the test result will be set to that value during statistical analysis, and is presented as "< value X" for data tabulation.

7.8 Missing Data

7.8.1 Last Survival Date

At the cutoff date for analysis, if the subject's death has not been reported, the latest date among the complete dates of the following data will be taken as the last survival date:

- Dates of all examinations and assessments for the subject (tumor response assessment, laboratory tests, vital signs, 12-lead electrocardiogram, physical examination, ECOG score, pregnancy test, etc.)
- Start and end dates of the new anticancer therapy (subsequent systematic new anticancer therapy page, subsequent radiotherapy page, subsequent surgery page)

- Onset and end dates of adverse events
- Date of the last known survival status on the survival follow-up page
- Start and end dates of treatment with the investigational product
- Dates of concomitant medications or therapies
- Date of withdrawal of informed consent form

Of the dates on the termination of the study page (if the reason for termination is "loss to follow-up", the date of loss to follow-up is not calculated), only the dates of actual examinations can be used to infer the last survival date. The dates of examinations and assessments after the cutoff date for analysis will not be used to infer the last survival date.

7.8.2 Other Dates

In the study, for the processing rules for missing date data in terms of date of death, adverse events, past medical history, drug exposure, prior and concomitant medications (including new anticancer therapy), demographics, etc., refer to Hengrui's *Guidelines for Imputation of Date-based Missing Data in Clinical Trials*.

All the imputed dates must precede the date of withdrawal of informed consent form, loss to follow-up and death.

8. STATISTICAL METHODS AND STATISTICAL ANALYSES

8.1 Statistical Methods

Descriptive statistical summary of data from the study will be performed by treatment group (SHR-1701 30 mg/kg + AG regimen), unless otherwise specified. For binary variables, statistical analysis will provide the number and percentage of subjects by category, and if necessary, provide the 95% confidence interval (Clopper-Pearson exact method) for the percentage of the category of interest. For continuous variables, statistical analysis will provide mean, standard deviation, median, quartile, minimum, and maximum. For time to event data, Kaplan-Meier method (also known as product limit method) will be used to describe the survival distribution and estimate the median time. If necessary, the 95% confidence interval for the median time (Brookmeyer-Crowley method) will be provided, and survival curves plotted.

In this study, baseline is defined as the last measurement or diagnosis prior to use of the investigational product/therapy.

Calculation of the study day:

For dates prior to the first dose, study day = current date - date of the first dose.

For dates after the first dose (inclusive), study day = current date - date of the first dose + 1 (day).

The date of the first dose is defined as Day 1.

For statistical analysis, a summary will be presented by treatment group SHR-1701 30 mg/kg, D1, q3w plus chemotherapy regimen (gemcitabine 1000 mg/m^2, D1 and D8, q3w; albumin-bound paclitaxel 125 mg/m^2, D1 and D8, q3w) (SHR-1710 30 mg/kg + AG regimen).

Number of decimal places:

Unless otherwise specified, the number of decimal places in the analysis report will be presented using the following rules:

The number of decimal places of the minimum and maximum will be consistent with that of the original data to be collected, means and medians will have one more decimal place than the original data, standard deviations will have two more decimal places than the original data. However, at most 4 decimal places will be retained.

Percentages will be rounded to one decimal place. In case the frequency is 0, no percentage will be shown.

P-values will be rounded to four decimal places. If the P value is <0.0001, it will be represented as "<0.0001", and if the P value is >0.9999, it will be represented as ">0.9999".

If there are decimals for 95% CIs, they will be rounded to at least two and at most three decimal places. Specifically: A 95% confidence interval will have one more decimal place than the original data. If there are no decimal places in the original data, the 95% confidence interval will be rounded to two decimal places; If there are 3 or more decimal places for the original data, the 95% confidence interval will be rounded to a maximum of 3 decimal places.

Time to event (months) will be rounded to one decimal place.

Hazard ratios will be rounded to three decimal places.

SAS Version 9.4 or above will be used for all statistical analyses.

8.2 Statistical Analyses

8.2.1 Description of Subjects

8.2.1.1 Subject enrollment and disposition

The total number of subjects screened, number of subjects enrolled in the study/receiving the treatment, number of subjects terminated from the study (by reason for termination), number of subjects discontinued from the treatment (by reason for discontinuation), number of subjects included in different phases (Phase Ib dose-finding and Phase II efficacy expansion), and number of subjects included in each analysis set will be presented.

Subject enrollment and disposition will be tabulated.

8.2.1.2 Demographic and baseline characteristics

Demographic characteristics

A descriptive statistical summary will be presented by treatment group for the demographic characteristics of the treated subjects, including age, gender, ethnicity, height, weight, BMI, ECOG score, and drinking history.

Baseline characteristics

The following baseline information of the subjects will be summarized by:

- Number and percentage of subjects by tumor site and stage;
- Number and percentage of subjects by treatment;
- Number and percentage of subjects by CA19-9 level;
- Number and percentage of subjects by prior therapy;
- Number and percentage of subjects by number of metastasis sites;
- Number and percentage of subjects by metastasis site/organ.

8.2.1.3 Tumor diagnosis

A descriptive statistical summary will be presented for tumor parameters including pathological grade, metastasis status, recurrence status, TNM stage, initial clinical stage, current clinical stage, and pathological type.

Descriptive statistics, including mean, standard deviation, median, minimum and maximum, will be used to summarize the duration of disease (months).

Duration of disease (months) is defined as the time (in months) from the date of initial diagnosis to the date of the first dose, calculated as: (date of the first dose - date of initial diagnosis + 1) / 30.4375.

A detailed subject listing for tumor diagnosis will be presented.

8.2.1.4 Past medical history

Data will be coded using the Chinese version of ICH Medical Dictionary for Regulatory Activities (MedDRA) 25.0, and the treatment history of subjects before entering this study will be summarized by treatment group (number and percentage of subjects), and tabulated.

8.2.1.5 Prior and concomitant medications

Prior and concomitant medications will be coded using the Anatomical Therapeutic Chemical (ATC) system of World Health Organization's Drug Dictionary (WHODD Version 2020Q1 or higher).

Prior medication is defined as any drug that has been discontinued prior to the first dose of the investigational product during treatment period. Concomitant medication is defined as any drug that is initiated only after the first dose of the investigational product during treatment period, or any drug that has been initiated prior to the first dose of the investigational product during treatment period but is still ongoing.

Prior and concomitant medications will be summarized and described by Anatomical Therapeutic Chemical (ATC) Level II and Preferred Term, respectively. In the summary description, a subject who has received the same drug (classified by ATC) on more than one occasion should be counted as once.

All prior and concomitant medications will be summarized and tabulated.

8.2.1.6 Subsequent anticancer therapy

The types and outcomes of subsequent systemic anticancer therapies (therapies after the last dose), the purpose of subsequent surgery, and the purpose of subsequent radiotherapy will be tabulated by treatment group.

8.2.1.7 Protocol deviations

The determination of any important protocol deviation should be completed before database lock. Important protocol deviations will be summarized.

All protocol deviations will be presented in a listing.

8.2.2 Safety Analysis

In all the following sections except for Section 8.2.2.1 DLTs, which is based on the DLT Set, all other safety analyses will be based on the Safety Set.

8.2.2.1 DLTs

DLT events will be summarized and tabulated based on the DLT Set only. The tabulation includes but is not limited to the time to onset of DLT, DLT type and name, actions taken, duration, and outcome.

8.2.2.2 Adverse events

MedDRA 25.0 will be used to code adverse events before statistical analysis. If a subject experiences an adverse event under the same System Organ Class (SOC) and Preferred Term (PT) on multiple occasions, the event will be counted only once for that subject.

In addition, adverse events will be graded per CTCAE V5.0. For multiple occurrences of the same adverse event, the highest/most severe grade will be taken.

The statistical analysis of adverse events will include but is not limited to the following:

- Summary of adverse events (regardless of the causality with treatment);
- Summary of adverse events (treatment-related);
- Incidence and severity of adverse events (regardless of the causality with treatment);
- Incidence and severity of adverse events (treatment-related);
- Incidence of Grade 3 or higher adverse events (regardless of the causality with treatment);
- Incidence of Grade 3 or higher adverse events (treatment-related);
- Adverse events leading to dose interruption or discontinuation or reduction of any therapy/drug;
- Adverse events leading to death;
- Incidence of serious adverse events (regardless of the causality with treatment)
- Incidence of serious adverse events (treatment-related);
- Incidence of special interest events;
- Immune-related adverse events related to SHR-1701;
- Immune-related adverse events treated with systemic hormone therapy.

The following adverse event endpoints will be tabulated separately:

- Adverse events leading to dose modification;
- Adverse events leading to death;
- Serious adverse events (SAEs);
- Special interest events.

8.2.2.3 Laboratory tests

The changes in post-baseline laboratory test results from baseline (shift table) for some parameters will be summarized (by the most severe CTCAE grade) (CTCAE V5.0) (Grade 1, Grade 2, Grade 3, Grade 4, not tested). This summary will include all scheduled and unscheduled visits.

In addition, this tabulation will present the laboratory test data. Specifically, abnormal values of laboratory tests during the study will be tabulated with presence of clinical significance indicated.

8.2.2.4 Vital signs

Descriptive summary will be presented for vital signs data in the following manner:

Descriptive summary of vital signs data and the changes from baseline by scheduled visit as specified in the protocol (including baseline and post-baseline);

In addition, all vital signs data will be tabulated.

8.2.2.5 12-lead electrocardiogram

12-lead electrocardiogram data will be descriptively summarized in the following manner or by classification criteria as specified in Section 10.1:

- (Normal/abnormal) changes from baseline (normal, abnormal and not clinically significant, abnormal, not tested) in post-baseline 12-lead electrocardiogram test results (by the most severe clinical significance assessment). This summary will include all scheduled and unscheduled visits;
- Descriptive summary of 12-lead electrocardiogram data and the changes from baseline by scheduled visit as specified in the protocol (including baseline and post-baseline); Summary of QTcF data by classification criteria (see details in Section 10.1).

In addition, all electrocardiogram data will be tabulated with clinically abnormal data noted.

8.2.2.6 Physical examinations

Changes from baseline (shift table) in post-baseline physical examination results will be summarized. This summary will include all scheduled and unscheduled visits.

In addition, all the physical examination data will be tabulated.

8.2.2.7 Other safety endpoints

Test results of serum CA19-9 and their percent changes from baseline will be summarized and tabulated by treatment group and nominal sampling time. A curve (individual curve) showing percent changes from baseline in CA19-9 over time will be plotted.

Changes in the worst post-baseline ECOG score from baseline will be summarized, and this summary will include all scheduled and unscheduled visits.

ECOG score, virological examination, fecal occult blood, blood amylase and lipase, and echocardiography will be tabulated.

8.2.3 Drug Exposure

Variables such as duration of exposure, drug exposure, actual dose intensity, and relative dose intensity of the investigational products (SHR-1701, albumin-bound paclitaxel for injection, gemcitabine) will be summarized using mean, standard deviation, median, Q1, Q3, minimum and maximum based on the Safety Set.

Overall dose intensity of individual subjects will be tabulated.

8.2.4 Pharmacokinetic Analysis

Format description for PK concentration data are presented in Table 5.

Table 5. Statistics and Format Description for PK Concentration

Parameter/Concentration	Statistics	Display format
Concentration data[1]	Individual subject data	Original data
	n, n of BLQ	0 decimal places
	Min, Median, Max, Mean, S.D., GeoMean	3 significant digits
	CV%, GeoCV%	1 decimal place

[1] If there are more than 50% BLQ concentration data at a sampling time point, then only n, n of BLQ, and Max will be summarized for the concentration data at that time point.

Concentration of serum SHR-1701 will be analyzed based on the PK Set as follows:

- PK concentrations will be summarized by sampling time point and treatment group. The summary statistics include n, n of BLQ, arithmetic mean, standard deviation, coefficient of variation (cv%), median, minimum, and maximum.
- The mean and median PK concentration curves will be plotted as a function of sampling time (on both linear and natural logarithmic scales).
- PK concentration data of individual subjects will be tabulated.

For summary statistical figures, the sampling time is the nominal sampling time.

8.2.5 Pharmacodynamic Analysis

Not applicable.

8.2.6 Efficacy Analysis

The efficacy analysis will be performed based on the Full Analysis Set (FAS).

8.2.6.1 Objective response rate

For objective response rate, see Section 6.5.

ORR will be calculated based on the Full Analysis Set, Per Protocol Set and Efficacy-evaluable Set, and its 95% confidence interval will be estimated using the Clopper-Pearson method. If the Per Protocol Set and the Full Analysis Set are identical, no analysis will be performed based on the Per Protocol Set.

8.2.6.2 Disease control rate

For disease control rate, see Section 6.5.

DCR will be calculated, and its 95% confidence interval will be estimated using the Clopper Pearson method.

8.2.6.3 Best overall response

For best overall response, see Section 6.5.

Descriptive statistical analysis of best overall response (CR, PR, SD, PD, NE) will be performed by treatment group.

8.2.6.4 Progression free survival

For progression free survival, see Section 6.5.

Median PFS will be estimated using the Kaplan-Meier method, survival curve plotted, and its 95% confidence interval estimated using the Brookmeyer-Crowley method.

The censoring rules for PFS are presented in Table 4.

8.2.6.5 Time to progression

For time to progression, see Section 6.5.

Kaplan-Meier method will be used to estimate median TTP and calculate its 95% confidence interval;

The censoring rules for TTP are basically the same as those for PFS, except for #3 in Table 4, which is to censor to the date of the last imaging assessment before death (if the date of the last imaging assessment before death is the date of imaging assessment before administration, then censor to Day 1).

8.2.6.6 Duration of response

For duration of response, see Section 6.5.

The Kaplan-Meier method will be used to estimate median DoR and calculate its 95% confidence interval;

The censoring rules for DoR are basically the same as those for PFS, except for removal of #1 and #2 in Table 4.

8.2.6.7 OS rate at 6/9/12-month

For OS rate at 6/9/12-month, see Section 6.5.

The Kaplan-Meier method will be used to estimate the OS rate at 6/9/12-month. The 95% confidence interval will be calculated using the normal approximation method, where the log-transformed 95% CI of this probability will be calculated first, and then back-transformed.

8.2.6.8 Overall survival

The Kaplan-Meier method will be used to estimate the median survival, and survival curve will be plotted. The Brookmeyer-Crowley method will be used to estimate its 95% confidence interval.

The censoring rules for OS are as follows:

- In case of loss to follow-up/premature withdrawal other than loss to follow-up/no death, the censoring date shall be the last survival date.

8.2.7 Immunogenicity Analysis

Immunogenicity analysis will be based on the immunogenicity set, and the number of subjects with baseline ADA-positive samples, ADA positive subjects, and ADA negative subjects will be summarized based on frequency and percentage by ADA confirmatory result and by treatment group.

The time to first detection of ADA positivity and duration of ADA positivity will be calculated, and a descriptive summary will be presented.

Subjects will be summarized by type of neutralizing antibody (NAb), among which NAb positive subjects should also be summarized by its target.

The titer results for ADA positive subjects will be summarized:

A descriptive summary of the titer results for ADA positive subjects will be presented by the nominal blood sampling time using statistics including number, median, 25th percentile (Q1), and 75th percentile (Q3), and the corresponding titer-blood sampling distribution plot (box-and-whisker plot) of ADA positive subjects will be presented.

The relationships between immunogenicity and drug trough concentration (C_{trough}) will be summarized:

A descriptive summary of C_{trough} for ADA positive subjects (treatment-boosted ADA positive, transient positive after baseline, persistent positive after baseline, other positive after baseline) and ADA negative subjects will be presented by the nominal blood sampling time using statistics including number, arithmetic mean, standard deviation, geometric mean, median, minimum and maximum, and a corresponding plot showing the relationship between immunogenicity and drug trough concentration will be presented.

The relationships between immunogenicity and primary efficacy endpoint (ORR) will be summarized:

A descriptive summary of ORR results for ADA positive subjects (treatment-boosted ADA positive, transient positive after baseline, persistent positive after baseline, other positive after baseline) and ADA negative subjects will be presented.

The relationships between immunogenicity and safety variables will be summarized:

Summary of safety variables (occurrence of Grade ≥3 treatment-related adverse events [TRAEs], including at least one immune-related adverse event [irAE]) of ADA positive

subjects (treatment-boosted ADA positive, transient positive after baseline, persistent positive after baseline, other positive after baseline) and ADA negative subjects will be presented. Immunogenicity relevant data will be reported in a listing.

8.2.8 Exploratory Analysis

Exploratory analysis will be based on the full analysis set (data from subjects without biomarker test results will not be assigned values).

A box-and-whisker plot of percentage of CD8 positive cells in immune cells and percentage of CD8 positive cells in total cells versus efficacy assessment will be presented. A scatter plot of Hscore score of pSMAD2/3 tumor cells and Hscore score of pSMAD2/3 stromal immune cells versus PFS will be presented.

A subgroup analysis of ORR will be performed in order to calculate point estimates of ORR for each subgroup and estimate the 95% confidence intervals for each subgroup using the Clopper-Pearson method.

If applicable, the Kaplan-Meier method will be used to perform summary analysis and plot survival curves for PFS and OS by PD-L1 and CA19-9 subgroups. Summary of median PFS and median OS will be presented for the following subgroups (age, gender, ECOG PS score at baseline, baseline CA19-9, site of metastasis, site of pancreatic tumor, and prior therapies).

9. REFERENCES

None

10. APPENDICES

10.1 Classification Criteria for Electrocardiogram

Classification Criteria for QTcF

QTcF(ms)	450≤ max. <480	480≤ max.<500	max. ≥500
Increase from baseline in QTcF(ms)	30≤ max. <60	max. ≥60	
